# Initiating and Continuing Behaviour Change within a Weight Gain Prevention Trial: A Qualitative Investigation

**DOI:** 10.1371/journal.pone.0119773

**Published:** 2015-04-15

**Authors:** Samantha Kozica, Catherine Lombard, Helena Teede, Dragan Ilic, Kerry Murphy, Cheryce Harrison

**Affiliations:** 1 Monash Centre for Health Research and Implementation (MCHRI), School of Public Health & Preventive Medicine, Monash University, Clayton, 3168, Victoria, Australia; 2 Diabetes and Vascular Medicine Unit, Monash Health, Clayton, 3168, Victoria, Australia; 3 Department of Epidemiology and Preventive Medicine, School of Public Health & Preventive Medicine, Monash University, Melbourne, 3004, Victoria, Australia; University of St Andrews, UNITED KINGDOM

## Abstract

**Background:**

Preventing obesity is an international health priority. In Australia, young women who live in rural communities are at high risk of unhealthy weight gain. Interventions which engage young women and support sustainable behaviour change are needed and comprehensive evaluation of such interventions generates knowledge for population scale-up. This qualitative sub-study aims to identify enablers and barriers to behaviour change initiation and continuation within a community weight gain prevention program.

**Methods:**

In-depth semi-structured interviews were conducted with program participants 6 months after baseline. All interviews were audio-taped and transcribed verbatim. Transcripts were analysed independently by two investigators via thematic analysis.

**Participants:**

A total of 28 women with a mean age of 39.9±6.2years and a BMI of 28.6±5.2kg/m^2^ were purposively recruited from the larger cohort (n = 649) that participated in the prevention trial.

**Results:**

Four behaviour change groups emerged were identified from participant interviews: (i) no change, (ii) relapse, (iii) intermittent and (iv) continued change. Factors influencing behaviour change initiation and continuation included realistic program expectations and the participant’s ability to apply the core program elements including: setting small, achievable behaviour change goals, problem solving and using self-management techniques. Personal knowledge, skills, motivation, self-efficacy, accountability and perceived social and environmental barriers also affected behaviour change. Satisfaction with personal program progress and the perceived amount of program supports required to achieve ongoing behaviour change varied amongst participants. Women who relapsed expressed a desire for more intensive and regular support from health professionals, identified more barriers unrelated to the program, anticipated significant weight loss and had lower satisfaction with their progress.

**Conclusion:**

Initiating and continuing behaviour change is a complex process. Our findings elucidate that initiation and continuation of behaviour change in women undertaking a weight gain prevention program may be facilitated by accurate and realistic understanding of program expectation, the ability to apply the core program messages, higher internal motivation, self-efficacy and minimal social and environmental barriers.

**Trial Registration:**

Australia & New Zealand Clinical Trial Registry ACTRN12612000115831 www.anzctr.org.au

## Introduction

Obesity prevalence has increased at an alarming rate in recent years and is one of the most pressing global health challenges. Presently, efforts to address the obesity epidemic have predominantly focused on costly, high intensity weight loss interventions with varied success and sustainability [1, 2]. In this context, the rationale for the prevention of excess weight gain remains clear [2, 3] and is an international health priority [4, 5]. Research has demonstrated and quantified the efficacy of weight gain prevention programs [6, 7], which are likely to be easier to implement, cost effective and potentially more valuable in the long term compared with treatments targeting established obesity [8–10]. Further research is needed to inform scale-up of efficacy trials, as a component of an integrated approach to address the obesity crisis [11–13].

Although evaluation and research are strongly related they serve different purposes. As described by Stufflebeam, evaluation seeks to “improve, not prove”, whereas research aims to generate new knowledge and “prove” hypothesises [14]. Thus, program evaluation informs progress beyond isolated research studies in an attempt to broaden implementation and scale-up phases, while simultaneously informing program adaption for diverse target groups [2, 15, 16]. Best practice principles for community-based obesity interventions describe the integral role of program evaluation [12], yet limited well designed robust evaluations have been conducted in this setting [3, 17]. Whilst enablers of successful weight loss factors in overweight and obese individuals have been investigated quantitatively and include heightened self-efficacy and intrinsic motivation [18], few studies have applied robust qualitative methods to explore potential short term enablers and barriers to behaviour change in tandem with program delivery [19]. In addition, the majority of studies have explored influences of behaviour change post program completion, rather than at the time when new behaviours are being adopted [20]. As lifestyle management remains the cornerstone to obesity prevention and management, further knowledge of factors influencing the processes of making and continuing behaviour changes is needed.

In the present study we therefore aimed to understand the barriers and enablers of behaviour change initiation and continuation in rural women, participating in an active weight gain prevention trial, the Healthy Lifestyle Program (HeLP-her rural) [21]. Understanding the personal and program related factors which affect participants’ abilities to modify their behaviour inclusive of healthy eating and physical activity will likely improve the overall effectiveness of this novel program and prevention programs more broadly.

## Methods

### HeLP-her Rural study design

HeLP-her Rural is a pragmatic cluster randomised controlled trial (RCT) involving 649 reproductive aged women across 41 rural towns(population sizes of 2000 to 10,000) randomised to intervention (n = 21) and control (n = 20) [21]. The program is designed to be a low intensity, low cost and non-prescriptive program, underpinned by the social cognitive model [22] and self-determination theory [23]. This program focuses on building self-management capacity in order to achieve long-term sustainable behaviour change.

Intervention participants of this RCT receivied one group session delivered by a trained facilitator, a program specific manual (participant manual with simple lifestyle messages and self-directed activities), ongoing support via text messages and one telephone coaching session. Broadly the intervention focused on: 1) improving healthy eating and physical activity knowledge, 2) behavioural self-awaness (identifying personal barriers and enablers), 3) skill development (goal setting, action planning, addressing barriers, problem solving, and relapse prevention skills) and 4) setting small feasible behavioural change goals such as minor caloric restrictions and small increases in physical activity (i.e. increasing fruit and vegetable consumption, reducing soft drink intake, walking for 30 minutes per day). Control participants received only a single group health information session consistent with standard national dietary and exercise recommendations. The primary outcome of the HeLP-her Rural program was the difference in weight gain between the control and intervention communities at 12 months post program initation.

### Evaluation approach and theoretical framework

A mixed-method and multi-level evaluation of the HeLP-her Rural program was employed in order to capture both process and summative evaluation outcomes. The multi-level evaluation was conducted at both the participant (individual) and community (organisational) level. A theory-based evaluation approach underpinned by the social cognitive model [22] and the self-determination theory [23] was also applied. The social cognitive model suggests that health behaviour may be influenced through multiple avenues such as goal-setting, self-monitoring, self-efficacy, problem solving and relapse prevention training [22]. The program evaluation was guided by the RE-AIM framework, which addresses program Reach, Efficacy, Adoption, Implementation and Maintenance [24].

### Sampling and recruiting

Purposeful sampling techniques were applied for the semi-structured interviews to obtain a representative sub-sample from the larger RCT cohort. A criteria based, convenience sampling approach was performed according to the following criteria; 1) towns allocated to receive the intervention, 2) local government region (equal representation across all five local government regions involved) 3) town population size (2000–7500) and 4) socioeconomic status (SES) informed by Socio-Economic Indexes for Areas (SEIFA) and index of social advantage derived from Australian Census data [25]. We evaluated the SEIFA index of each town involved in the RCT and then selected a representative sub-sample of towns based on the average SEIFA index. Twelve towns were eligible for participation in the qualitative sub-study and six were randomly selected for participation. Participants were representative of all intervention communities and interviews were conducted until data saturation was reached, determined when no new ideas emerged from the interviews, as per standard methods [26]. Written consent was provided and a follow-up letter and phone call delivered to consenting participants. All participants received information regarding their involvement prior to participation and incentive gift vouchers were provided. This study was approved by the Monash Health Research Ethics Committee for research involving humans.

### Data collection

In-depth semi-structured telephone interviews of 25–50 minutes duration were conducted between March and August 2013, approximately 6 months post program commencement. A single researcher conducted all participant interviews with an interview schedule informed by a review of the literature. The interview schedule focused on 5 broad topics; 1) Motivation for program attendance, 2) Program expectations, 3) Behaviours change (enablers and barriers to both initiation and continuation of behaviour change), 4) Exploration of program engagement and utilisation and 5) perceived personal achievements (S1 Table).

All interviews were audio-taped using a digital recorder, de-identified upon conclusion of the interview and transcribed verbatim by an independent transcribing service (S1 Data Set). The interviews were conducted until a theoretical point of data saturation, determined when no new ideas emerged from the interviews, as per standard methods [26, 27].

### Data analyses

De-identified transcripts were analysed using thematic and inductive analysis, enabling the identification, coding and categorisation of primary patterns within the data. All transcripts were independently analysed and coded by two investigators (SK and KM) in accordance with the predetermined constructs. In depth discussion of emerging themes took place before a final iteration of the results was developed and agreed upon across investigators. An independent qualitative researcher was included to counteract the dual role of the researcher delivering and evaluating the program. Thematic coding of data and development of models were assisted by the NVivo Software program (QSR International Pty Ltd. Version 10, 2012, Victoria, Melbourne). Verbatim quotes from interviews that best represented the key findings for each theme were highlighted for subsequent reporting purposes.

## Results

A total of 28 women participated in the qualitative semi-structured interviews from 6 rural communities. Table 1 provides an overview of participants’ demographic and anthropometric characteristics.

**Table 1:** Demographic and anthropometric characteristics of participants.

Baseline characteristics	Participants (n = 28)
**Age: mean (SD)**	39.94 (6.23)
**BMI: mean (SD)**	28.65 (23)
**BMI categories: n (%)**	
**Within the healthy weight range**	6 (21)
**Overweight**	12 (43)
**Obese**	10 (36)
**Years living in rural Victoria: n (%)**	
**1–5 years**	6 (22)
**6–10 years**	3 (11)
**>10 years**	19 (67)
**Highest level of schooling: n (%)**	
**Year 12 not complete**	7(25)
**Yeard12 completed**	21(75)
**Highest level of education: n (%)**	
**No qualification**	5 (18)
**Certificate/Diploma**	11 (39)
**Bachelor**	12 (43)
**Work status n: (%)**	
**Full time**	6 (21)
**Part time/casual**	14 (50)
**No paid work**	8 (29)
**Household income: n (%)**	
**<$40,000**	6 (21)
**$41,000–64,000**	7 (25)
**$65,000–80,000**	7 (25)
**>$80,000**	8 (29)
**Marital status: n (%)**	
**Married/defacto**	26 (92)
**Never married/divorced**	2 (8)
**Household: n (%)**	
**Living with children under <16**	24 (86)
**Not living with children <16**	4 (14)

Results are presented as mean ± SD or relative frequencies (%). Abbreviations: BMI: body mass index.

### Behaviour change

Of the 28 women interviewed 27 reported initiation of some behaviour change post program commencement. Participants reported behaviour changes relating to diet (i.e. increasing fruit and vegetable consumption, reducing intake of soft drinks and fast foods), exercise (i.e. increasing walking and incidental exercise), or self-management (i.e. joined a local commercial weight loss program or sought additional nutrition information) all of which reflected the overall messages and aims of the program.

Four behaviour change groups were identified at the conclusion of the 6-month follow-up period including:

1. Continued change: change continued over 6 months
2. Intermittent change: inconsistent change over 6 months with alternation between modified behaviour and relapse.
3. Relapse: behavioural changes made initially but not continued over the 6 month period
4. No change: no behaviour change initiated post baseline.

Within these four groups, seven prominent themes emerged which differentiated the groups including:

1. Motivators for program participation,
2. Program expectations,
3. Program message application and utilisation,
4. Personal knowledge, skills and self-efficacy,
5. Accountability,
6. Social and environmental barriers,
7. Personal achievements and program support

Table 2 lists the similarities and differences between these four groups with respect to the seven key themes outlined above.

**Table 2:** Behaviour change and the associated enablers and barriers to behaviour change initiation and continuation post progracommencement.

	Continued behaviour change	Intermittent behaviour change	Relapse	No change
**Motivators for participation**	Weight and health	Weight and health	Weight and health	Health and university reputation
**Program Expectations**	Realistic and accurate	Unrealistic yet modifiable	Unrealistic	Nil
**Program message application and utilisation**	Enhanced self-awareness, utilisation of self-management strategies and found program support valuable	Enhanced self-awareness, utilisation of self-management strategies and found program support valuable	Not evident	Not evident
**Personal knowledge, skills and self-efficacy**	High motivation, self-efficacy, and self-discipline	Moderate motivation, self-management and confidence level	Poor motivation, self-efficacy, self-management and confidence	High motivation, self-efficacy, and confidence
**Accountability Orientation**	Internal	External	External	Internal
**Social and environmental barriers**	Not described	Fluctuating motivation, injuries, commitments and limited resources	Reduced motivation, lack of support, injuries, and commitments	Not described
**Personal achievements and program support**	Satisfied with progress and level of program support provided	Moderate satisfaction with progress, requesting additional support	Perceived progress poor and requesting additional support	Satisfied with progress and level of program support

### 1) Motivation for program participation

Wanting to lose weight and improve health and well-being was a recurrent theme across the interviews. This theme was consistent amongst all women interviewed, regardless of behaviour change grouping. Notably, the desire for substantial weight loss was described more commonly in the ‘intermittent change’ and ‘relapse’ groups. All groups exhibited similar motivation for program participation. However, the personal drive to commit to the program was often affected by individual and environmental barriers—be it perceived or actual.

“We all know what we need to do, but it’s just a matter of doing it. Being driven enough to, you know, what it and all that sort of thing.”–[Intermittent change group].

### 2) Program expectations

Behaviour change was facilitated by realistic program expectations at the commencement of the intervention with participants in the ‘continued change’ group and ‘intermittent change’ groups. Women in the ‘continued change’ group reported joining the program with more realistic program expectations.

“I went with an open mind I didn’t really know what was going to be involved”—[continue change group].

Conversely, women in the ‘relapse’ groups reported less realistic expectations, with majority seeking goals that were outside the scope of the program such as stress management or significant weight loss, with one woman reporting that she was seeking “the motivation pill”. Women who were less successful in changing their behaviour had enrolled with greater and therefore less feasible expectations, which were also reflective of previous unsuccessful attempts at weight loss.

### 3) Program message application and utilisation

Participants in the ‘continued change’ and ‘intermittent change’ groups highlighted that behaviour change was facilitated by the application and utilisation of program messages. Program messages detailing the importance of self-management, goal setting and the value of making small cumulative changes to caloric intake and physical activity resonated most with participants.

“It wasn’t a case you must lose 20kg and you have to stop eating and diet…and deprive yourself. It was all about small changes that were manageable without feeling you were a failure if you didn’t do the whole lot at once”–[continued change group].This message strategy did not resonate with women in the ‘relapse’ and ‘no change’ groups, with majority unable to repeat the core program messages. For example women who reported relapsing at 6 months attributed their initial behaviour change to heightened self-awareness and elevated motivation; however, these effects were short term.

“That [the program] was good to start off with, but I could sort of push [the program messages] by the wayside” [Relapse group].

Interestingly, the program was able to modify the expectations of the ‘intermittent group’ to appreciate the benefits of weight gain prevention and to value the importance of making smaller, cumulative lifestyle modifications, however, this was not the case for the ‘relapse’ group.

“It was all about making small changes that make a big difference in your life”- [intermittent change group].

“Just the whole start small, you don’t have to do it all at once because small changes in the end have the better outcome, they’re more longer lasting”—[Intermittent change].

### 4) Personal knowledge, skills and self-efficacy

Participants from all behaviour change groups agreed that, initial lifestyle change was enabled by improved self-awareness of lifestyle choices and improved motivation immediately following program commencement. However, enhanced self-monitoring capacity, harnessing self-management techniques, improved knowledge, and prioritisation of personal health emerged as enablers to continued behaviour change, for women in both the ‘continued change’ and ‘intermittent change’ groups only.

“It’s probably made me more aware of saying, you know, you can have that (biscuit) but it might contribute to that one kilo extra a year”- [continued change group].

Displays of motivation, self-efficacy and self-management tended to be on a decreasing path from the ‘continued’ change group to the ‘relapse’ group. Notably, women in the ‘continued change’ group described a heightened level of motivation, self-efficacy, self-worth and self-management prompting lifestyle changes.

“I’m actually worth this. I’m actually going to find time today to do something [exercise]”- [continued change group]

In comparison to the ‘continued change’ group, women in the ‘intermittent’ group expressed lower confidence, motivation and self-discipline, as well as being influenced greatly by described barriers to behaviour change.

“Motivation fluctuates quite a lot… depend[ing] like, on how busy I am at work, or what's going on in the household—even down to you know what time of the month it is”- [intermittent change group].

The women in the ‘relapse’ group displayed the lowest levels of motivation and self-management capacity. Furthermore, some revealed a strong sense of apathy and an ongoing battle with confidence.

“When you lose motivation or interest you don’t seem to take an interest in it”-[Relapse group]

Participants in the ‘no change’ group described having high levels of motivation and self-confidence to maintain a healthy lifestyle

“I'm just more proactive, if you're going to do it, do it”- [no change group.]

### 5) Accountability

Accountability arose as an important factor influencing behaviour change. ‘Internal accountability’ was defined as the ability to take personal responsibility for behaviour change and to motivate oneself to undertake change. ‘External accountability’ was defined as the desire or need for external feedback and motivation to undertake behaviour changes. The ‘continued change’ group reported internal accountability, partly demonstrated by joining other weight management program or physical activity programs which met their needs.

“It all comes down to you, you’ve got to do it, you’ve got to make the plan, you’ve got to buy the food”–[continued change group]

“When you get to where you want to be [ideal weight] you can feel proud that it’s you that has done it and it hasn’t been someone telling you what to eat, what to do, how much to eat. So it just feels like a bigger victory”–[continued change group]

In contrast, women in the ‘intermittent change’ and ‘relapse’ group commonly appeared to be seeking external accountability, looking for additional support and contact. However, the need for seeking external accountability differed between these two groups, with women in the ‘intermittent change’ group viewing it as a motivator for change, whilst women in the ‘relapse group’ viewing it as an integral need for behaviour change. Any inability to access external accountability was perceived to be a barrier in achieving a change in behaviour in this group reporting, “I can’t do it alone—[relapse group]. However, the women in the ‘intermittent change’ group also reported benefiting from the program accountability provided.

“Knowing that there would be checks later on down the track, that kept me motivated”-–[intermittent change].

### 6) Social and environmental barriers

Personal barriers to behaviour change were explored in all interviews and as expected greater barriers were reported by participants unable to maintain behaviour change. Women in both the ‘intermittent’ and ‘relapse’ groups reported that multiple barriers contributed to their inability for continued behaviour change. Women in the ‘relapse group’ expressed a reduced capacity to overcome these barriers. Commonly described personal barriers included injuries, multiple work and family commitments, financial stress and minimal support from partners to be able to find time for physical activity. In addition environmental barriers reported included lack of child care opportunities and geographical isolation to purchasing healthy foods and participating in regular physical activity such as joining a local gym.

“My situation is lack of access because we're right in the bush, young children, running a home business and having weight issues”–[intermittent change.]

There was also a connection between personal perceived barriers and environmental barriers. Decreased personal confidence, coupled with the perceived social impact of participating in a public trial about a physical condition that also has inherent social and emotional drivers was a significant barrier for many women.

“It’s going publicly and going, I need this help to lose weight… I’m a bit of a private person”- [Relapse group].

### 7) Personal perceived progress and support

Participants’ ability to reflect upon their personal progress throughout the program influenced their ability to facilitate or maintain behaviour change. A strong sense of pride with their achievements was commonly reported amongst participants in the ‘continued change’ group.

“I’m healthier now than what I was, definitely healthier, definitely fitter than when I started the program”- [continued change group].

This similar sense of achievement declined in the ‘intermittent change’ group, many indicating moderate satisfaction with their progress. Whilst they had made changes within their own limitations, they commonly reported requiring additional behaviour changes to achieve personal goals.

“I may not have lost a lot of weight, I have lost weight and I am continuing to lose weight only through the slight changes that we’ve made”- [intermittent change group].

The women in the ‘relapse’ group discussed that they were disappointed with their lack of progress and many underplayed successes and doubted their progress.

“I just hope the scales will be down by next December and not up”–[relapse group]).

The need for intensive support to achieve ongoing behaviour change varied across the groups. Women in the ‘relapse’ group expressed the greatest need for intensive and frequent support from health professionals. Participants in the ‘intermittent’ and ‘relapse’ groups requested prescriptive health information particularly regarding nutrition information, “this [amount of food] is appropriate, this is adequate”. Many women in the ‘relapse’ group appeared to be seeking an intensive weight loss programs, stating “you need the reminder every day don’t you?” and “one session doesn’t motivate me”.

## Discussion

This qualitative enquiry elucidates the enablers and barriers to initiating and continuing lifestyle change in women participating in a community weight gain prevention program. We revealed that the HeLP-her Rural program was successful in supporting women to initiate behaviour change in almost all participants. Based on our analysis four behaviour change groups emerged, varying from those who reported continued behaviour change to the few who reported no change. Within these four groups, influences of behaviour change emerged, helping to explain how and why continued behaviour change was or was not achieved. Continued behaviour change was prompted through the application of core program messages, specifically the utilisation of a small behavioural change approach to improving lifestyle choices and the development of realistic program goals. Personal factors (motivation, self-efficacy and self-management capacity) and orientation of accountability additionally explained participants’ behavioural change. Participants reporting continued behaviour change described fewer personal and environmental barriers and a higher sense of personal achievement.

Our findings suggest that program message application and utilisation likely promoted continued behavior change in women within this weight gain prevention trial. One program message that was frequently reported as highly valuable by participants was the application of a cumulative small behavioural change approach to improving lifestyle choices. Based on our semi-structured interviews, we ascertain that this approach contributed to behaviour change because it: 1) enabled women to feel confident and empowered that they could achieve important lifestyle changes, 2) assisted women to modify their expectations in order to understand the importance of weight gain prevention, 3) reduced pressure to succeed, and 4) broke down the notion that weight management must involve significant modifications to nutrition and exercise patterns. Our findings are consistent with the literature suggesting, that the acceptability of intensive weight loss programs with larger goals are generally poorly received in this population, who typically have limited time and have multiple competing demands [28]. The value and feasibility of a small change approach to lifestyle modification may contribute to informing the design and execution of future healthy lifestyle programs.

For many participants the development of realistic and feasible program goals led to improved ability to make lifestyle changes. This finding was consistent with a qualitative study conducted in obese women, which reported that individuals with more modest weight loss goals and less ‘fantasizing’ about ideal weights, were more likely to be successful in managing their weight [29]. According to our participants an improved sense of self-worth, confidence, motivation and prioritization of their own health contributed to continued behaviour change. This finding is also congruent with the results of two previous literature reviews exploring the interplay between psychosocial influences and weight management. These reviews highlight that behaviour change is more likely to be achieved in women with increased self-management capacity, self-efficacy, self-monitoring and internal motivation influences[18, 30]. This may have been reflected within the ‘no change’ group who, similar to the ‘continued change’ participants, reported high motivation and increased self-efficacy, yet did not engage with our healthy lifestyle program. This was likely due to the belief that their current health behaviours were consistent with the information provided during the program, therefor requiring no further modification.

According to our participant’s accountability orientation and the intensity of support provided from health professionals influences behaviour change. Our study shares common findings with a previous qualitative weight management trials, supporting the notion that internal accountability orientation can improve behaviour changes success [31]. Furthermore, Thomas et al investigated accountability and attitudes to weight loss in obese women, exploring what they believed “would work” to reduce their weight. They found that many women expressed difficulties losing weight independently without sufficient support and were seeking someone external to take responsibility for their weight management [32]. Similarly, here the women who were unable to continue behaviour change expressed the desire for more intensive programs to achieve ongoing behaviour change. These women appeared to require greater supports than those typically provided by low intensity weight gain prevention programs. However, the long term effectiveness of intensive programs is poor with high probabilities of weight regain and elevated program attrition rates [33]. Thus, it is unclear if intensive weight loss programs would be effective in rural women over the long term.

Whilst the HeLP-her program supported behavior change in many participants, barriers to behaviour change were discussed in-depth by women reporting relapses. Consistent with prior literature, the women unable to continue behaviour change reported barriers including financial stress, multiple commitments, limited access to exercise opportunities and fresh produce, and a lack of social support [32, 34] all of which are external factors to the program aims. Despite verbal and written messages clearly articulating that the aim of the HeLP-her Rural program was to prevent weight gain in women, many of the women unable to continue behaviour change reported seeking weight loss rather than maintenance. This may explain why there was a lack of engagement with the HeLP-her messages of weight gain prevention. This work also potentially suggests that despite a focus on moderation of expectations at the commencement and throughout the intervention, women may cling to the hope of weight loss. As such personal disappointment in their achievements may result, despite meeting the program aims to make some change in health behaviours. Exploration of strategies to better align program goals and participant expectations is an area requiring further investigation.

### Future directions

The development of a tailored approach to healthy lifestyle change and weight management ideally should be incorporated into future prevention programs. The exploration of enablers and barriers to behaviour change in women pre-intervention would be an important step in informing the type, quantity and intensity of support required at an individual level. This is supported by studies suggesting that researchers may need to tailor interventions to individual needs to improve success rates and maximise cost effectiveness [35, 36].

### Strengths and limitations

Strengths of the current study include the application of an in-depth qualitative research method to a large, rigorously designed weight gain prevention RCT. In addition, exploring behaviour change patterns and influences during an active intervention has potentially allowed us to identify behaviour change initiation, irrespective of whether behaviour change is continued throughout the entire length of the intervention. Such information would likely be missed if data was to be collected only at the commencement and completion phases of an intervention, which is common practice [20]. We also applied robust qualitative analysis methods using a theoretical framework and two independent researchers were involved in data analysis. Moreover, the purposeful sample increases the generalizability of the qualitative results to the wider RCT cohort. In future, as the results of this RCT become available we plan to conduct data triangulation with quantitative outcomes (weight, nutrition and exercise patterns).

Limitations include the dual role of one of the researchers (SK), who delivered and evaluated the program as described here. However, to address potential bias, we engaged an independent and experienced external qualitative researcher in the analysis process who was not involved in either study delivery or data collection. We acknowledge the inherent weakness of qualitative research including potential for researcher and participant bias and smaller sample size [37].

## Conclusion

Behaviour change is complex and an enriched understanding of the enablers and barriers to behaviour change will likely improve the effectiveness of future obesity prevention efforts. Our findings suggest that low intensity weight gain prevention programs can support positive behaviour change in women. Here, behaviour change initiation and continuation were influenced by realistic program expectations, ready application of program messages, higher internal motivation, self-efficacy, self-awareness, and minimal perceived environmental and social barriers. Women, who commonly relapse report a desire for more intensive program supports, identify more barriers to behaviour change unrelated to the program, anticipate significant weight loss and are likely to be less satisfied with their progress. Lifestyle program design should include simple achievable health messages, small changes to behaviour and reinforcement of program objectives including realistic weight objectives, as well as supporting internal accountability.

## Supporting Information

S1 Table Participant semi-structured interview schedule. (DOCX)

S1 Data Set Participant semi-structured interview transcripts. (DOC)
