# Supplementary material for: Initiating and Continuing Behaviour Change within a Weight Gain Prevention Trial: A Qualitative Investigation
Source: PLoS One. 2015 Apr 15;10(4):e0119773. doi: 10.1371/journal.pone.0119773 (PMC4398548; doi:10.1371/journal.pone.0119773)
Supplement: S1 Table — (DOCX) [file pone.0119773.s001.docx]

| Community profiling   - Overview of community characteristics, demographics, environment and social dynamics |
| --- |
| 1. Motivators, enablers and barriers for program participation  - Recruitment strategies utilised - Participants motivators for attendance - Reported potential local barriers to program participation |
| 1. Program expectations  - Exploration of participant’s initial expectations of the program - Participants perceptions of the program purpose i.e. weight prevention, healthy eating and improving lifestyle |
| 1. Program engagement and utilisation  - Evidence of participants knowledge gains post program - Evidence and examples of utilisation of the key program messages i.e. goal settings, small behaviour change goals, relapse prevention |
| 1. Program effectiveness  - Perceived effectiveness of the program demonstrated through knowledge gains, behaviour changes and shifts in attitudes - Exploration of the length of behaviour change i.e. short term change only, intermittent or continued change post program commencement - Investigation of the enablers and barriers to behaviour change continuation   i.e. lack of personal motivation, confidence, personal factors |
| 1. Perceived personal achievements during the program |

# S1 Table

# Participant semi-structured interview schedule
