# Supplementary material for: Initiating and Continuing Behaviour Change within a Weight Gain Prevention Trial: A Qualitative Investigation
Source: PLoS One. 2015 Apr 15;10(4):e0119773. doi: 10.1371/journal.pone.0119773 (PMC4398548; doi:10.1371/journal.pone.0119773)
Supplement: S1 Data Set — (DOC) [file pone.0119773.s002.doc]

# S2 Data Set

# Participant semi-structured interview transcripts.

Participant #MM

Full transcript

Facilitator: Okay, perfect. So just to start off with a pretty easy question, can you first off just talk to me a little bit about whether there's many opportunities to exercise in Yarram or opportunities to buy healthy food?

Interviewee: I don’t know about the exercise part but yes there's a gym here. Healthy food, you mean vitamins and things?

Facilitator: Yes or just fruit and vegetables, anything fresh.

Interviewee: Yes. There's the supermarket.

Facilitator: Yes, got that. What about exercising? Are there good walking paths, good walking tracks?

Interviewee: Yes.

Facilitator: Do you often see many women exercising?

Interviewee: Yes, I do.

Facilitator: What about yourself? Are you an active person?

Interviewee: Very much so. I've got to do the opposite, I've got to stop, slow down, stop for a while.

Facilitator: Stop and slow down, yes.

Interviewee: I probably do too much.

Facilitator: Yes. You said that you often see people walking. Is it groups of women normally or just women by themselves do you think?

Interviewee: Just probably one or two. I've never seen groups walking.

Facilitator: Yes. Overall as a...

Interviewee: ...there isn't.

Facilitator: Yes, of course. As a community overall would you think that people living in Yarram it's quite a health conscious community or not a health conscious community?

Interviewee: It's very sporty. Yes, I'd say it's healthy.

Facilitator: Yes. When you say very sporty what do you mean by that?

Interviewee: Well with the football, there's junior footy, there's netball, badminton, there's AusKick. We've got everything here.

Facilitator: So plenty of opportunities to be active.

Interviewee: Yes.

Facilitator: Would you consider the community to be quite social? If you wanted to socialise are there many opportunities to do that?

Interviewee: That's the only downside to this place. No, they're not very social here.

Facilitator: Not very social. What do you mean, it's just hard to meet people or - can you expand on that a little bit?

Interviewee: It's hard to fit in if you're not from here.

Facilitator: Yes. Are you from there?

Interviewee: No.

Facilitator: No. So it's not very welcoming?

Interviewee: Been here for five years and it's sort of you always feel like you're on the outer a little bit. I don’t know whether it's a small town thing or what.

Facilitator: Yes. So it's quite a, would you say, cliquey community?

Interviewee: Very much so.

Facilitator: Yes, so hard to come in from an outsider.

Interviewee: Yes, very much so.

Facilitator: That's interesting. Now I want you to think back to when we first came to Yarram. Do you remember what motivated you to join the healthy lifestyle program?

Interviewee: Well to meet people.

Facilitator: No, that's a good reason and you're certainly not the only one.

Interviewee: I mean some of them I knew anyway.

Facilitator: You're not the only one that's said that. Any other reasons? Was it to receive health information or...

Interviewee: Well no, not really, just overall health really.

Facilitator: Yes, just to get some insight into overall health.

Interviewee: Yes.

Facilitator: Yes.

Interviewee: Yes.

Facilitator: Were there any barriers or anything that made it difficult for you to attend that program or easy to attend?

Interviewee: I fitted it around work because I work part-time so it actually worked in that I wasn't working that day you were here so that was all right.

Facilitator: Yes. As you probably say in that room in Yarram when we had the session at the school we had quite a few women but not quite as many as we'd have liked. Can you think of any reasons as to why that was the case?

Interviewee: The ones that were there are like on the school committees or they do fundraising. They were those sorts of people. Yes, I don’t know, they probably thought they were a bit better than that.

Facilitator: Yes. When you say those sorts of people...

Interviewee: Yes.

Facilitator: ...do you mean the people that always sign up for everything?

Interviewee: Yes.

Facilitator: Yes. The other people you think didn’t attend you said a second ago "because they thought they were better than that."

Interviewee: Well yes, I mean they probably were but if it's getting out and mixing with new people they wouldn’t want to do that.

Facilitator: Yes, they don’t like to mix with new people. Now thinking about the actual program was there any health information that you took away or any new information that you learnt?

Interviewee: No, not really.

Facilitator: Because we had two sections. We had the first part was about just general healthy lifestyle advice about diet and exercise and the second part focused on more behaviour change techniques like problem solving, goal setting, developing this action plans.

Interviewee: Because I'm a social smoker. I wanted to give that away. But as far as weight loss I don’t need to lose any weight and I don’t need to do any exercise either.

Facilitator: Yes, you're already very active. So that information was quite familiar to you would you say?

Interviewee: Yes.

Facilitator: Yes. What about the part about the behaviour change techniques like goal setting, problem solving that can be applied to any part of your life such as smoking?

Interviewee: What do I need to change?

Facilitator: No, was that new to you?

Interviewee: No.

Facilitator: No.

Interviewee: Getting out of the habit.

Facilitator: Yes. But had you heard before about setting small goals and about barriers and problem solving?

Interviewee: No.

Facilitator: No. So that information was new. That's good.

Interviewee: Yes.

Facilitator: Yes. So I'm glad to hear you've taken away something. The information you mentioned - so you said that the behaviour change information was new to you but the nutrition stuff was quite familiar. Did you find it helpful though to have a bit of a refresher or was that helpful to you?

Interviewee: Well what I just said, I can eat what I want and I don’t put on weight. [audio cuts out] help because I've got a grown-up kid living back at home and he's a little bit overweight.

Facilitator: Yes. So you think it still could be valuable even to hear information that you already know.

Interviewee: Yes. He's not very active either, you know, he’s playing footy but...

Facilitator: Yes. So if you were to describe this healthy lifestyle program to someone else, a friend or a family member, how would you describe it? So if you were to finish my sentence "the program was about" what would you say?

Interviewee: It's about documenting what you eat and what bad habits you've got and how you can change them. I'd show them the book.

Facilitator: Yes. Do you think it focused on weight?

Interviewee: Yes, I thought it probably was.

Facilitator: Yes. Is there any information that you would have liked extra that may have been helpful to you?

Interviewee: No, I don’t think so. It was focused on weight which is a good thing because there's so many overweight people, some of the people that were there that day.

Facilitator: Yes. So there's a lot of overweight people in Yarram?

Interviewee: Yes.

Facilitator: Yes. In your opinion do you think that since you started partaking in this program you've thought more about your weight, about how much you're exercising and about what you eat?

Interviewee: Yes, well I have cut down on eating but I've changed jobs too. That's probably why I lost a bit of weight.

Facilitator: Yes. So it's not a result of the program, it's a result of changing jobs.

Interviewee: Yes.

Facilitator: Have you made any - since being involved in the program - changes to what you're eating, how much you exercise, smoking? Have you made any changes to your lifestyle?

Interviewee: I'm just working so yes I've tried. But I have cut back heaps.

Facilitator: Yes. So you've cut back. Has that been since starting the program or just in general?

Interviewee: No, it's been since starting the program.

Facilitator: Yes, good. So you've cut back heaps.

Interviewee: Yes.

Facilitator: How have you...

Interviewee: I'm only a social smoker and I don’t smoke all the time.

Facilitator: Yes. Have you done anything else in terms of changed what you eat or how much you exercise since starting the program?

Interviewee: No.

Facilitator: No. What about...

Interviewee: I changed jobs which made me...

Facilitator: Made you do a bit more.

Interviewee: Yes, not nibbling on food.

Facilitator: Yes. What about access to services? Since being involved have you accessed any other services that perhaps you may not have done previously like a GP or got a health check, anything like that?

Interviewee: No. I just monitor my blood pressure at the chemist or when I go to my neurologist every month.

Facilitator: So has that been new or that's just been long term?

Interviewee: No that's been ongoing.

Facilitator: Ongoing, yes. So you said you'd only changed smoking, is that correct?

Interviewee: Yes.

Facilitator: Is that because you said you were already active and already eating...

Interviewee: Yes.

Facilitator: Yes. So you didn’t need to change anything.

Interviewee: No.

Facilitator: Yes. You did mention - which is great - that you're trying to cut back on smoking since the program started. What's helped you do that do you think?

Interviewee: I've probably been a little bit more busier.

Facilitator: So the program in the session we talked about goal setting and about looking at barriers and obstacles to improving our health. Have you thought of smoking in terms of that?

Interviewee: Yes.

Facilitator: Yes, good.

Interviewee: I've kept myself busier so I don’t need to do that and I don’t probably mix with those sorts of people as much.

Facilitator: When you say those sorts of people what do you mean by that sorry?

Interviewee: Smokers.

Facilitator: Smokers, yes.

Interviewee: Smokers.

Facilitator: Yes. So you've said you've done a bit of goal setting so that's helped you?

Interviewee: Yes.

Facilitator: Have you used any of those action plans that we discussed in that first session and you also spoke to somebody on the phone not long ago?

Interviewee: No I haven’t done that.

Facilitator: That's all right.

Interviewee: I can’t find the book.

Facilitator: Yes, that's okay, no problem. So was the goal setting a new skill for you since joining this program?

Interviewee: Yes.

Facilitator: You've found that valuable and helpful?

Interviewee: Yes.

Facilitator: Good. I'm glad to hear that you've been able to apply it.

Interviewee: …to get that book.

Facilitator: Yes, we've got the book. What about those SMSs? Has that support helped you to achieve your goal of reducing your smoking?

Interviewee: About the eating.

Facilitator: Yes, they're more about eating.

Interviewee: Yes.

Facilitator: If you think more broadly about what's - so you've said you've stopped smoking. You said the goal setting helped you. Any other factors that have helped you achieve your goal?

Interviewee: No, just the price of them.

Facilitator: Yes, the price. What about your family? Have they helped support you?

Interviewee: The kids have. Hubby smokes so it's hard there going down that path.

Facilitator: Yes. What about the kids, so how have they helped?

Interviewee: Well they don’t sort of say anything but that might have helped I think. The fact they don’t say anything.

Facilitator: Yes, okay. Now if we think about the different components of the program. So far we saw you in Yarram for that group session then we've been sending your SMSs. We've also had that website, the program website and the manual, the book that you talked about.

Interviewee: Yes.

Facilitator: Have you found any of those parts of the program more helpful than others?

Interviewee: Probably the book.

Facilitator: You found the book helpful?

Interviewee: I very rarely get on the computer.

Facilitator: You don’t use the computer.

Interviewee: No because it means you've got to sit there and...

Facilitator: Yes. So the book - and that's...

Interviewee: Sit in one spot. I just read the book at night.

Facilitator: Yes. Why do you think the book helped you more than the messages or the phone call? Any reason?

Interviewee: Well I try and read anyway because that’s helping my daughter who doesn’t like reading. That's the only reason I'll read books.

Facilitator: Yes, to help her.

Interviewee: Yes.

Facilitator: Yes. Did you find that phone call helpful?

Interviewee: Which phone call?

Facilitator: No, not this one. You got one a couple of weeks ago.

Interviewee: Yes.

Facilitator: Someone talked to you about an action plan and set some health goals with you.

Interviewee: Yes. I'll just find the book.

Facilitator: No, that's all right, you don’t need the book. Was that helpful, that phone call?

Interviewee: Yes.

Facilitator: What about the text messages? Some people have found them helpful and other people have found them not so useful. What about you?

Interviewee: No, not the text messages.

Facilitator: Yes. So when you get one what do you do?

Interviewee: I just read it and the delete it.

Facilitator: Yes, good, thanks for being honest. So it's not very helpful for you. So if we were to rerun this program in order to get you as motivated as you are if we kept the book and we kept the phone call but we cut out the text messages would you be at the same level?

Interviewee: Yes. I'm not up with the whole technology thing.

Facilitator: Yes, not good with technology. But if we cut that book - just to clarify - you wouldn't be as motivated, is that correct?

Interviewee: No.

Facilitator: No.

Interviewee: Just from getting texts, no.

Facilitator: But if we removed that book that wouldn't be good for you?

Interviewee: No.

Facilitator: Yes, so you like the book. Okay, good. Now the other thing that I wanted to look at is whether you're somebody that - again there's no right or wrong answers - talks about your health with your friends, with your family? We're trying to look at whether this program affected anybody else outside the program or just the people that came to that session in Yarram in that classroom. So have you talked about the program with anybody?

Interviewee: No.

Facilitator: Is that in character for you, to not talk about your health?

Interviewee: Well no because I don’t have a problem with my health as such, only I've got to go to a neurologist but everyone knows about that. That's probably more of an issue for me than...

Facilitator: Yes, anything.

Interviewee: Yes.

Facilitator: So would you say that the main reason that you haven't talked about it is just because you don’t really have a health problem?

Interviewee: Yes.

Facilitator: Yes. Any other reasons why you don’t talk about - why you haven't mentioned this program to anybody else?

Interviewee: Well if I was to say to some large person "you should have a look at this" they might get offended by that.

Facilitator: Yes, I agree. What about to any of your friends? Do you talk about health with them or what you're eating, about your weight?

Interviewee: Just in general chitchat at work, yes.

Facilitator: Yes. Your family, did you mention - I know that for you a lot of the information was a refresher so you haven't actually changed anything about diet or your exercise.

Interviewee: No.

Facilitator: But have you mentioned anything else extra to your family that you may not have mentioned had you not have been in the program?

Interviewee: No.

Facilitator: No. That's all right, no problem.

Interviewee: These kids need to cut back on their sugar and stuff.

Facilitator: Yes. Do you talk to them about that?

Interviewee: Yes.

Facilitator: Yes. Have you made any changes to what you buy, what's in the pantry?

Interviewee: Well I don’t buy it, yes. But I frown upon everyone when there's soft drink around but...

Facilitator: Yes. But that's been ongoing, is that right? That's not new since you started the program?

Interviewee: Yes.

Facilitator: Okay. The last thing I want to ask you about is just about your satisfaction with the program. So again I want you to be completely honest because the only benefit for us is to find out what you really thought so don’t sugar coat anything. But based on your experience do you think you'd recommend this program to any of your friends that live in the community or any of your friends that live in another town?

Interviewee: Yes, I would recommend if they needed to lose some weight but I mean I wouldn’t go and - yes.

Facilitator: So you wouldn't go and...

Interviewee: No. People might get offended by that.

Facilitator: No, you wouldn’t just go up to people. No, fair enough, that wouldn't be probably too socially acceptable. When you think of this program do you think of it as a weight loss program, a weight prevention or just a healthy lifestyle program if you had to pick one of those terms?

Interviewee: A healthy lifestyle program.

Facilitator: Yes. Would you think of it as a weight loss or weight maintenance?

Interviewee: Well I'm hoping it's a weight loss for some people but - weight maintenance.

Facilitator: Like keeping weight off or more weight loss program. Like keeping weight the same or weight loss.

Interviewee: Keeping weight the same I guess.

Facilitator: Yes, you got that. Okay. In terms of the lady that ran the program would you have preferred if she was - do you have any preference about whether she was from Melbourne or whether she was from Yarram or somewhere local?

Interviewee: No.

Facilitator: Does it bother you?

Interviewee: No, it didn’t bother me. Only that they got lost coming over here.

Facilitator: Yes, I remember that. That was bad, wasn’t it.

Interviewee: Yes, the GPS. A lot of people have done that.

Facilitator: I know, it's very difficult. I was in that car, it was awful and we could not work out where we were. It really was really not great. But do you think that would bother other people?

Interviewee: Well I don't know. It doesn't bother me.

Facilitator: Yes. What about in terms of to get more people into the program do you think it would have helped if we had someone local on board?

Interviewee: It might have. I don’t know what the hospital run up there. They've got a dietician and things up there. There was a Weight Watchers...

Facilitator: Group.

Interviewee: ...group in Yarram. I don’t even know whether that's still going or not. I haven't a clue.

Facilitator: Have you ever been in any other health programs like this?

Interviewee: No.

Facilitator: No.

Interviewee: No.

Facilitator: What motivated you to join this one particularly?

Interviewee: I just went along to see what it's all about.

Facilitator: Yes, good. The venue, were you happy with where we hosted the session?

Interviewee: Yes.

Facilitator: Yes. Last question, would you prefer a group or an individual session to receive health information?

Interviewee: It doesn't bother me.

Facilitator: It doesn’t bother you.

Interviewee: No.

Facilitator: But if you had a preference?

Interviewee: Probably group.

Facilitator: Yes. Why do you think a group's more appealing for you?

Interviewee: Well again I don’t have a problem with health and such so I don’t know.

Facilitator: Yes. Some people liked their group because they liked the group discussions and they liked the support that they get from the group.

Interviewee: Okay.

Facilitator: So does that apply to you or not really?

Interviewee: I feel a bit awkward talking about my weight...

Facilitator: In a group.

Interviewee: Yes. See I didn’t say anything much that get-together we had here because I'm not overweight like 90 per cent of the other ones were so...

Facilitator: You didn’t want to say anything.

Interviewee: No. I just wanted to go and have a look, go and see what it's all about.

Facilitator: Yes, good. Did you think the information we provided was adequate? Was it enough?

Interviewee: Yes.

Facilitator: So there's nothing else you would like to have heard about?

Interviewee: No.

Facilitator: If we could change the program to make it better any recommendations of how we could improve it?

Interviewee: No.

Facilitator: Would you prefer more contact, less contact, more group sessions, no text messages?

Interviewee: More group sessions would be good.

Facilitator: Yes. Anything else? Take away anything, add anything?

Interviewee: No.

Facilitator: No. Okay, good. Is there anything else you want to add about your experience of being in the program? Anything you think's important for us to know when we're trying to evaluate the success of this program?

Interviewee: No. When are you coming back again?

Facilitator: We're coming back in - I think we came about October/September last year to Yarram so we'll be back then.

Interviewee: Yes. That'll be good.

Facilitator: Good. But there's nothing else you want to add?

Interviewee: No.

Facilitator: No. All right, great. Thank you again so much for your time. I'll send you that gift voucher and the card in the mail tomorrow and hopefully that should come to you by the end of the week but please give us a call if you don’t receive it. But that's good to hear from you and I'm glad - it sounds like everything's going well.

Interviewee: No worries. Thank you.

Facilitator: Perfect. Thanks for your time.

Interviewee: Thanks. Bye.

Facilitator: Bye.

End of transcript

Participant# BA

Full transcript

[Interviewee off microphone at the beginning]

Interviewee: As long as I’m careful and don’t get the ball on my eye, I should be fine.

Facilitator: Well that’s motivated. So the interview just covers a couple of brief topics. The first thing is – it covers motivation. What we’re trying to do is just work out what people liked about the program. Why they decided to join and about the satisfaction with the program. Do you have 20 minutes now, is that okay?

Interviewee: Yes, that’s fine. As long as you’ve got 20 minutes.

Facilitator: Yes, I’m fine, I’ve got nothing too exciting planned.

Interviewee: Okay. Can I make a suggestion, can I just – I’m having a bit of trouble with reception here. Can I just give you my home phone number and can you ring that? Is that okay?

Facilitator: Yes, no problem.

[Facilitator redials home phone number]

Interviewee: Yes that’s better; I can hear now.

Facilitator: So can you hear me now? Sorry.

Interviewee: No that’s fine.

Facilitator: I’ve just got a whining dog in the background.

Interviewee: Oh, have you? [Laughs] Okay.

Facilitator: The first part of the evaluation, the first question I had for you is if you can just describe your community in Yarram; about the size of the community, the average age you think of the people in the community, and any comments you have about major health problems in the community or…

Interviewee: Okay. Of course I’ll answer you from my perspective.

Facilitator: Yes.

Interviewee: My perspective okay, I’ve got a bit of a soapbox about this so that’s fine.

Facilitator: Yes.

Interviewee: Okay, small, country, little town where people are very involved in sport until about 30 years old. After that people just aren’t as active as they should be. I’m lucky enough to be part of a basketball club of older players in Melbourne who sort of understand the benefits of good health. I get a little bit frustrated in my local town that a lot of people my age don’t play sport.

A very active football club for six months of the year. A little bit of other sport played, a little bit of badminton and squash and some basketball, but people just don’t tend to do sustained active sports in Yarram, especially over about, I don’t know, 30 I suppose. We have a small gym in Yarram which I was a member of for a while. Very expensive, not much to offer really and quite restricted in hours of operation, so I sort of started that for a while and thought, ‘No, this doesn’t work for me’.

Look, it’s availability of venues. I play netball with the local club but I have to travel 25 kilometres to train and then it’s another hour’s drive most games unless we play at home. We have a basketball court which is accessible but only available say one or two nights a week because others – you know, it’s got to be shared around. We only have one court, if that makes sense, for our whole town. We have squash courts and badminton courts and tennis courts and that’s about it.

I mean, no things such as Pilates clubs and things that you might have access to in Melbourne. No Curves Fitness or anything like that that sort of caters for people my age, I suppose.

Facilitator: What about – so would you consider overall the town to be active then?

Interviewee: No. I wouldn’t. I consider the children to be fairly active. I mean I’m a teacher at a primary school; I taught Phys Ed last year. There’s quite a big push for the young people in our town to be active. I wouldn’t even say the over 18 year olds are overly active, and certainly not the next age group, you know the 30s to 50s, and definitely not the 50s and overs.

Facilitator: Would you consider the town social connected? So would there be lots of opportunities to socialise, or…?

Interviewee: Yes and no. I think it’s what people make of it. I’m a very social person so I’m out three or four nights a week with it, playing sport or doing social activities. Of course, in my workplace I have friends and peers who don’t socialise at all. They sort of say there’s not a lot of social opportunities. I suppose it doesn’t really cater for them. I mean, I’m into sport I suppose and catching up with friends. This is sort of – in my school there’s, I don’t know, between 25 and 50 but – perhaps not really. Like there are not a lot of restaurants that people can just go out to socialise. Yes, not a great deal. I don’t think anywhere near as much as Melbourne – as many opportunities as Melbourne or Sydney or big places.

Facilitator: And any health problems that stand out to you? Would you call the community one that obesity and….is a problem?

Interviewee: Yes, yes. Especially – don’t get on until my age group I suppose. That’s I guess – that’s where my opinion comes from.

Yes certainly lifestyle; it’s obesity, is the main one. A few of my friends are diabetic. Not so much the smoking, like my social group there’s no-one really that smokes, so that’s a good thing. We don’t have that associated problem, but diabetes, I have friends with diabetes, obesity, and look, just lack of wanting to get up and do anything. It really frustrates me. We’ve got on our staff some women that I guess are menopausal, about the same age as me, and as they reach for the second Tim Tam they say, “Oh, I just can’t lose weight because I’m menopausal”, and they just don’t help themselves I don’t think.

It’s really disappointing because I really do love sports and being active. I look around and think, ‘These people are wasting so much of their time’, but that’s my opinion. As I said, I just love my sporting pursuits and I think it’s really sad that they keep blaming the menopause, the dreaded menopause, or lack of opportunities when really it’s their choice, the sedentary lifestyle and the eating too much and things like that. They are lifestyle choices – rather sitting in front of the television rather than going for a walk.

And I notice that, certainly in my school environment, that’s I guess I’m most entitled to speak about, but on a staff of let’s say 15 women, there’s only perhaps myself and one or two others that are even involved in sport and activities.

Facilitator: So inactivity is a problem?

Interviewee: Yes, it certainly is. Yes, the people that I know.

Facilitator: Yes, yes.

Interviewee: But then I’ve got lots of sporting friends, but they tend to be much younger. I’d say the netball team and – I play in two netball teams and Monday night one they’re all young teachers; I think I’m the oldest player by 25 years. It’s sort of 25 years is the next age in my netball team. Follow the football. I’m certainly the oldest. My basketball I’m the oldest by far.

Facilitator: What keeps you active and so motivated?

Interviewee: I love it. I’m lucky, I play in a club called the Missing Legends in Melbourne, a basketball club, and our oldest player is 75 and they know how to keep fit and they want to keep fit.

Look it’s also the socioeconomic thing down here. My friends in Melbourne – I’m not saying, but they’ve sort of all been doctors, lawyers, accountants and had opportunities and some money, being able to pursue their lifestyle of playing sport. A lot of the people I’m talking about down here sort of go to work – also too, perhaps our integration aides and some of the teachers and then they feel they don’t have time for anything else or the money to do anything else.

Facilitator: Yes, so the money to exercise and be active?

Interviewee: Yes. You know, I’m lucky, I’m going to Italy in two months’ time to play basketball.

Facilitator: Oh, wow!

Interviewee: Yes, the World Masters so I’m lucky to be able to afford that and a lot of the people I work with couldn’t ever afford that anyway.

Facilitator: But still they could go for a walk or…?

Interviewee: Oh, they could but – you know, I really get frustrated that they just tend not to want to do that. I just don’t get it because I just – look, I must admit it has been school holidays and I’ve been slack but I can’t wait to start netball again, you know. I can’t wait for basketball to start. I don’t understand how people don’t have the same drive and energy.

Facilitator: Is it for you, is it about the health benefit? Or what’s it just…?

Interviewee: I don’t know. I just really enjoy playing sport. I guess so, yes. Yes, I like to think I’m very healthy but I just really love it. I mean I play sport four nights a week. Drives my husband crazy. I do try to be home to cook. My boys have left home now so that makes it easier. I like to – my boys, yes, I’ve done lots of coaching. It sounds like I’m a paragon of exercise. I’m not. I could always do a lot more but I just really enjoy doing it.

Facilitator: That’s great, yes. And keeping on with that, you said a lot of other people in the town aren’t active and don’t make good health choices.

Interviewee: I don’t think so. Look, I’m talking about the ones that I’ve been in contact with, yes.

Facilitator: And that, yes – we’re interviewing a few people so it’s good to get your opinions, so what you think and your experiences. But why do you think, in Yarram – we had a pretty good response overall – a big thanks to you for pushing our teacher session – what do you think would be the barriers for women attending? So why do you think we probably didn’t get quite as many women involved in the program as we would have liked? Any insights?

Interviewee: Well, look, I sort of asked lots of our staff to do it. I just think lack of interest, you know? That’s what really frustrates me. I often ask the people at school, “Do you want to join my netball team. Would you like to play basketball?” And they’ve always got an excuse. I just don’t understand it myself. If anyone asks me it’s like, “Yes, yes please. I’d like to play.” But they just – I don’t know if they haven’t been brought up with it. It’s a place for the young age to learn the skills I suppose and I guess that’s what I’m up against. So people my age now I say, “Do you want to play on my…?” “Oh, I’ve never played before. I can’t play.”

And perhaps that’s small town; that they never learned when they were young. I mean, it’s big here now – don’t get me wrong – but I didn’t grow up in this town but perhaps Yarram 30 years ago didn’t have junior basketballers, and they perhaps didn’t learn how to play. I don’t really know. I think you have to have some skills. When you get to a certain age you have to have some skills otherwise you won’t ever play it.

Facilitator: And maybe they don’t feel confident?

Interviewee: No, that’s it. It’s a confidence thing. And I sort of bounce around a bit, you know like this advocate stuff, “You’ve got to play. You’ve got to play”. I don’t think I intimidate anyone but I never get a, “Oh yes, I’ll come and play.” Like…

Facilitator: And the interest, do you think that’s more of like health being a priority? Is that what you mean? Or…

Interviewee: I think it’s they just like watching television and eating chocolate, to be honest. That sounds simply harsh I know, but I’m just thinking about the ladies at my school, not so much the general community but certainly – and a lot of the parents at our school. It’s just easier to go home and watch tellie and make unhealthy choices, is my opinion.

Facilitator: And do you think that comes down to maybe health isn’t a priority? Or…

Interviewee: I would say so. And look, they keep having excuses and you know, I had kids and yes, it was harder to exercise but I always found an opportunity to exercise. But by not watching television. You know what I mean, cutting out television time or cutting out other things. I guess you have to prioritise, don’t you always?

Facilitator: Yes. So you think maybe it’s not a priority?

Interviewee: No, it’s not a priority.

Facilitator: Yes.

Interviewee: And look, I think even this day and age, like we certainly aren’t a model family, but if we had Christmas presents we’d buy our kids a trampoline and a swimming pool rather than indoor games and, you know…

Facilitator: And laptops…

Interviewee: You know, they’ve got a big farm and they can kick the football. That’s what they are meant to do. So we lucky, we live out of town. I guess we were lucky that we were able to – I mean certainly not the early years – but able to afford that sort of thing for our kids. And my husband is very fit and active as well. So yes, motorbike riding, and all those sorts of things that kids do, we did it with them.

Facilitator: So you’re role modelling as well?

Interviewee: Yes, that would be it, certainly. You see I play – and when my boys come home from Uni I expect them to come in and play basketball with my team. We used to have a pick-up game on Wednesday nights, and I play netball with my boys with a mixed netball team, so – but you don’t see that happening very often around Yarram.

Facilitator: Do you think the session time would have been a problem? We had the teachers’ one after hours but we had the women’s one during working hours.

Interviewee: I don’t think so. Look, I just see it’s a general lack of apathy. I don’t know if it’s in small, rural towns or general. We have trouble getting parents anywhere at all. If we offer food it’s often incentive or you can come along with a feed or door prize, there’s food, but it’s just an apathy. I think it has a little to do with the socioeconomic status of a lot of our people in our town.

Facilitator: Yes, it’s to do…yes.

Interviewee: Like I sound elitist but they don’t get it, or it’s just easier not to, or they lack confidence perhaps.

Facilitator: So, yes, lack confidence…

Interviewee: Yes, and if their friends aren’t doing it they won’t do it. All those sorts of…

Facilitator: Yes, and that’s a common thing?

Interviewee: Yes. I think it’s peer group – I think it depends on their peer group. A lack of – see, my peer group don’t usually play sport but I’m confident enough to just go and – yes, like when I first started playing basketball at the World Masters there was no-one in Yarram to go so I had to put my name down on the internet and ended up playing with a team from Dalby and then a team from Melbourne. So you have to have that access I suppose, and the interest.

Facilitator: Yes, access and interest.

Interviewee: Yes.

Facilitator: And also it sounds like, from what you’re saying, friendship groups and peer support is important as well?

Interviewee: Absolutely. I don’t sort of play most of my peers. Like now it’s the younger kids I suppose. But yes, at the Masters’ Games it’s certainly peers and all that sort of thing, with like interest. But around Yarram it’s really hard to get anyone of my age, but I just go on and do it anyway. I don’t really care. I think that I’m the old lady who stills netball and basketball. I really don’t care.

Facilitator: No, as long as you’re enjoying it, who – it doesn’t matter.

Interviewee: Oh, yes. I guess you have to be having, not success, but you know I’m lucky that I’m still able to do it to a reasonable degree. I’d hate for people to be thinking – you know, if I wasn’t up to it I wouldn’t play. You know what I mean? I don’t want to embarrass myself or my kids though you have to have some sort of, I guess, ability if that makes sense.

Facilitator: Yes. So since you’ve been in the program - you probably remember when we were in Yarram – since then we had the group session at the school, we’ve done the SMS, the manuals, we’ve had access to the website. Are there any key messages or anything you’ve learned or retained that stands out to you about the program?

Interviewee: Look I guess I’m sort of lucky, it’s already sort of been at the back of my mind anyway, health and fitness. And I guess a good thing with the reminders are unfortunately I always fall off the wagon. Like over school holidays, because I lead a very healthy lifestyle throughout the year, over school holidays if I want to indulge in the cakes and the biscuits that’s when I do. That’s when sport is usually, certainly over the last two weeks, there hasn’t been a formal sporting commitment apart from netball which starts tomorrow. So I guess in the school holidays I certainly fall by the wayside, but not to that sort of standard.

Facilitator: No, not to what you said the rest of the town is doing. Are there any…?

Interviewee: Yes, like I said, there doesn’t seem to be any sustained sporting activities that go on all throughout the year anyway.

Facilitator: But did any of that information – was it more of a refresher for you? Or was some of that information new to you that we talked about in the sessions?

Interviewee: Look, most of it was a refresher. You know, because I have led a healthy lifestyle. You could always learn new things, don’t get me wrong, but the things like drink more water, eat the serves of fruit and veg. I guess being a teacher we’re – you know, the socioeconomic group that sorts of understands; we teach health to our kids at school so we have a fairly good information base ourselves, and we do like to role model for the kids as well.

Facilitator: Yes, good.

Interviewee: So I guess if you’re talking to teachers about that sort of thing they’re pretty much on the ball anyway as far as information and knowledge. Not everyone follows the information and knowledge but you certainly find that they are well informed.

Facilitator: So, as a rule most of the six messages we talked about, were familiar to you; about limiting soft drink and things like that?

Interviewee: Yes, yes.

Facilitator: Good. So if you were going to describe the program to someone, what would you have described your being in the program – how would you describe it? So if you were going to describe to someone that you’d been in this program, would you call it…?

Interviewee: Innovative. You know, like I’ve never been involved in something like that before. Well supported, informative, well run, well organised. Yes, I guess they’re probably the best words to describe it.

Facilitator: Yes, great. And since you joined the program – I know it’s a bit hard with you because you’re already very active and living a healthy lifestyle – but do you think it’s made you think more about your health and more about your weight and what you’re eating? So has it influenced…?

Interviewee: Look, as I said, because this has sort of been my mantra I guess, and as I said, everyone can always be healthier. Don’t be me wrong, I’m certainly not a lithe, you know, a little tiny person that runs every morning and things like that.

Probably for me it hasn’t. But it’s certainly maintained what I’ve been doing…which is good too because, look the older you get, and I’m 52 now, the older you get the bit harder it is every year. So in a way, for me, it was perfect timing again to revisit it all.

Facilitator: And when you say harder, you mean in terms of – what do you mean in terms of, sorry?

Interviewee: Sorry, in terms of…?

Facilitator: Well you said it gets harder. You mean to live a healthy lifestyle?

Interviewee: Well I guess when you’re 52 it gets that bit harder to get out of bed at 6 o’clock, go for your ride and you know? I just guess the body slows down a little as well. It’s just a fact of aging, isn’t it really?

Facilitator: Yes.

Interviewee: So I guess for my age, I know I’m a little bit outside what normally people do that are a bit older, it is that little bit – you know, so much in my body has been okay but it is, it gets that bit harder. You know? The morning and all that sort of thing and…

Facilitator: To keep at it.

Interviewee: I guess I have the time now because my kids are older as well, so it’s a double-edged sword isn’t it really? I guess the time where we had the time to exercise, do all those things, and can afford all the healthy food and things like that, our bodies are letting us down a little, so…

Facilitator: Yes. I get what you’re saying, yes. And in terms of the different aspects of the program, did you find some more valuable than others? Like the manual or the text messaging? What have you found the most useful? The group session, the manual, the text messages…?

Interviewee: The text messages are good, every so often you get, “Oh, yes, okay.” They are straight away a reminder, aren’t they, the text messages?

Facilitator: So you found them a good reminder?

Interviewee: Yes, they are great reminders.

Facilitator: Yes.

Interviewee: And I’ve had the odd ‘phone call here and there and that’s been really good as well; keeps you on track.

Facilitator: Yes. What about the group session?

Interviewee: Sorry?

Facilitator: What did you think of the group session?

Interviewee: Yes, it was good. It was good. Yes, yes. I don’t think there’s any other way you could really do it. And there’s that one-on-one first for the statistics and that sort of thing.

Facilitator: Yes, where we measured you and…yes.

Interviewee: [Overtalking] keep that private I suppose.

Facilitator: Yes. And have you used the website?

Interviewee: No I haven’t used the website.

Facilitator: Do you use the internet much?

Interviewee: Look for school I do. It’s one of those things, you know, it’s sort of the last thing I suppose, and because I feel I’ve got a handle on it, have the information, the knowledge, no I haven’t.

Facilitator: Yes.

Interviewee: If I was looking for information I’d probably use it.

Facilitator: Yes. And if we were going to change the program slightly, you’re pretty – it sounds like you said you’re very motivated, but if we took away the manual or the SMS messaging, do you think it would change where your level of motivation is to keep healthy?

Interviewee: I think so, yes. I think it needs those supports.

Facilitator: Yes. So you think all of them have been good?

Interviewee: Yes, I think so.

Facilitator: Did you find the manual useful?

Interviewee: Yes, I did. You know, it’s one of those things you read at the very start, though you don’t tend to read – like in your busy lifestyle you read it and think, ‘Oh yes, I knew that’, or ‘Oh, that’s a fact I’ve got to remember’. You do tend to put them away.

Facilitator: Yes. Okay good. And since you started the program, have you made any other additional changes to your lifestyle? Have you increased your exercise at all? Or it’s been the same?

Interviewee: Well, it probably hasn’t improved – you know what I mean? Like I’m bike riding most mornings and playing sport.

Facilitator: So you were already doing that, weren’t you?

Interviewee: Yes. Probably more fruit in my diet. As I said, apart from having a healthy diet, fruit wasn’t something I found that palatable. I have been making a real effort to have at least one piece of fruit a day. So lots of veggies and lots of salads in my diet, but I don’t know, fruit wasn’t something that I really enjoyed, but really making an effort to have a piece, at least one piece of fruit every day.

Facilitator: Perfect. That’s a good change.

Interviewee: Yes.

Facilitator: And what about – have you been able to maintain that since we saw you four or five months ago?

Interviewee: Yes, I have.

Facilitator: Great. And have you accessed any other services in the community since we saw you that maybe you thought – some women have come along and thought, ‘Oh, I’d better go and get my Vitamin D checked’, because we talked about different health issues. Have you made any other [overtalking]?

Interviewee: No. It’s quite interesting because the year before I had a really big Vitamin D episode and I actually was really very, very sick. Saw lots of doctors and had muscle tremors and yes, I was really low in Vitamin D so I was very aware of that. I don’t sort of take vitamins. I just, you know, healthy lifestyle and lots of exercise.

Facilitator: Yes, good.

Interviewee: I guess I have started bike riding since then. Like that wasn’t something that was part of my daily regime but it is now.

Facilitator: Good, yes.

Interviewee: So yes, that started about then. I reckon it was about October/November I bought my bike.

Facilitator: Oh good, that’s when we were there last time.

Interviewee: Yes, so that’s certainly been added from before.

Facilitator: That’s great.

Interviewee: Yes.

Facilitator: And when you said you’ve increase a bit with your exercise a little bit, you said you’ve been doing a bit more riding and you’ve been eating more fruit, did you use any of those behaviour strategies that we talked about? So there was the goal setting, the problem solving, the relapse prevention or the action…?

Interviewee: Probably sub-consciously. You know what I mean? Often don’t think about them, it’s just part of your sub-conscious. Yes, so I’d definitely say so.

Facilitator: Any example of how you’ve used fruit? So with the fruit, any example of how you could have put the goal setting in place or the problem solving? Or…?

Interviewee: Well look, every day in my classroom now, at 10 o’clock we have brain food.

Facilitator: Great.

Interviewee: It’s fruit. So, yes, the kids remind me as well. So we work together. I often forget so the kids say, “It’s 10 o’clock, it’s brain food fruit time”. We can only have fruit in class at that time.

Facilitator: Yes, so again that sort of rearranging of time…

Interviewee: Yes. And if you make it the same time every morning you tend to remember.

Facilitator: Yes. Has the fruit day being going for a while?

Interviewee: Yes, yes. And I always have a piece of fruit on my desk now, on my table. Not that I can just randomly kind of eat it in front of the kids but they know that it’s there for when it’s fruit time.

Facilitator: Yes. So again…

Interviewee: So it’s sort of on display for the kids. They have theirs in their bags. I just get a piece out…

Facilitator: Put it out so that they can see it. Yes.

Interviewee: Yes.

Facilitator: So it’s about that planning?

Interviewee: And it’s quite funny, the kids, being little country kids, they’ll often have something special grown on their tree. I’ll say, “That looks really nice”, and the next day I’ll have two or three of those in my fruit bowl.

Facilitator: Oh, they must love you.

Interviewee: Yes, I’d like to think we all just love each other and in our class – you know what I mean, but sort of that sharing of, if I’ve noticed something that they’re eating. It’s certainly not things that they’ve bought, I wouldn’t do that. But kids have had Nashis on their tree and they’ve brought – and I’ll say, “I love that Nashi” so of course for a couple of weeks the Nashi tree had fruit on I was kept in Nashis and things like that.

Facilitator: That’s nice. Nice to share.

Interviewee: Yes it is nice.

Facilitator: Just moving on again. In terms of the program reach, I know it sounds that you’re already very active and you’re already trying to motivate a lot of other staff, but have you changed any of your food habits so that it would have affected your husband since you started the program? So have…?

Interviewee: Look, when I say yo-yo dieter, I have a healthy eating diet. Like I said, in the school holidays I just made some cakes and some muffins, things that I don’t normally do. I don’t [audio cuts out] because organised sport isn’t going and I always put on over the January break a kilo or two every year, and I know I shouldn’t but it’s…

Facilitator: That’s okay.

Interviewee: We have a swimming pool, we have people over and I don’t drink alcohol but we buy chips and dips every so often. So I have to make a conscious effort but I don’t really call it a diet any more; it’s just really healthy eating.

Facilitator: Yes. And does that affect your husband?

Interviewee: He’s quite healthy as well so like he’s a very active man. We eat very healthy meals. We’re not over the top, macrobiotic anything like that. I mean, multigrain we eat in the fridge, and water all the time and just those sorts of things, fruit in the fruit bowl, lots of veggies and salads for dinner…

Facilitator: And has that changed in….

Interviewee: [Overtalking] out carbs.

Facilitator: Yes, good. Has that changed since starting in the program, or that’s been consistent?

Interviewee: Probably more conscious of it. Once again we sort of came from a base of – and I’ve got three boys who are extremely healthy, like junkie type kids that don’t eat carbs after 5pm, live on broccoli, carrots and every day. Like they’re amazing, our boys. They’ve been home over the Uni break and you know, there’s been no junk food in our house. I bought my boys Easter eggs, what a mistake! So they were on their beds, and they still sit on their beds, so…

Facilitator: So do you talk about health a lot with them?

Interviewee: Yes. It’s quite interesting; our second – our younger son was quite, not obese certainly not, but a thickset boy when he was 15 or so, and we tried all the subtle things because our other two boys were quite thin. We tried all the subtle stuff. I wouldn’t get home until 5 o’clock at night and he’d come home from school and like all kids do they’ll eat sort of what they could find. About 17 he sort of got the health message himself and you would not meet a healthier kid now. Nothing we could do really would help. I mean we offered sort – not bribe – let’s go for this together, let’s do this together. He’s always been active but now he’s just a fighting, little fit, lean machine, I can tell you…

Facilitator: Oh good.

Interviewee: So our three boys are all very, very health conscious.

Facilitator: Very health conscious, good.

Interviewee: Exercise every day and eat really healthy food.

Facilitator: That’s great. Did you tell the family that you were in this program?

Interviewee: No, probably not. So it does sort of pass on the generations. And that’s the other thing I see, like generational obesity. It really gets to me because, you know, I’m talking about my staff, I suppose, or our staff and our aides and you look at the larger people and their kids are all larger too.

Facilitator: And have any….?

Interviewee: And I know some of it they can’t help but I think some of it is what that they eat.

Facilitator: No it definitely is. I mean, some people – you have different starting points but if you’re willing to exercise and eat better you can change it.

Interviewee: I had to laugh. Like we’ve got three sons and the eldest one’s in the Navy and he comes home for a couple of weeks here and there. Well, the three of them all run down the road – and they can all do six kilometres easily – but if the three of them, they’ll do seven, and then eight, and they’ll come back absolutely dripping with sweat and then they’ll have their weights and because it’s like a big competition they’ll lift more and more. And I think, ‘You boys!” It’s all this testosterone and egging each other on I suppose, but it’s good.

Facilitator: It’s good. It’s good. They can set an example for the rest of the town.

Interviewee: It does.

Facilitator: And have the women in – so obviously we came to Yarram and we did the teacher session. Have you guys talked much about the health messages? Or have you been talking about any of the things you’ve learned to the other colleagues?

Interviewee: We do, I admit. But it’s more a group of us that, you know, I guess what we eat I suppose, more than anything else. I walked in the staff room and said, “What are you having there?” and “Can we have the recipe for that?” Two of the ladies are on the Michelle Bridges’ program. One lady has lost heaps of weight. And we sort of do, we encourage each other, we notice the health [audio cuts out]. We don’t ever comment on the unhealthy food, but we notice and we swap recipes I suppose.

Facilitator: That’s great, yes.

Interviewee: And we congratulate each other on goals. I was even thinking we’d go back – like a bit of a, as I said, I want to lose four or five kilos before I go to the Masters Games. You know, it’s going to be hot over there and I want to be absolute peak fitness so I’m sort of thinking about some sort of club when we get back just to lose a couple of kilos each, I suppose, and to increase our exercise. But I’m not sure how to do it. I’ve got a few ideas. We’ll see.

I don’t want to make it a big issue but there’s certainly on our staff there’s a few that could certainly benefit, myself included, as I said. I’m a healthy size; I’m 72 kilos and 172 centimetres, so I wouldn’t want to get any larger but the most healthy sort of body [overtalking].

Facilitator: Yes. Any specific examples of how you’ve influenced any of your staff, or any of your friends, to eat better?

Interviewee: Well I guess so. Like I said, we’ll take something healthy to school and share recipes. I suppose that’s the best way of doing it. Yes, I suppose so. I mean, I’ve got this couple of really healthy salad recipes and a couple of times I’ve taken them along to my friends and said, “Try this. It’s really nice”. Yes, it’s just all veggies and nuts and, yes, so we do; we’re quite good at that.

Facilitator: Good at sharing it. And sorry, has that been the same since starting the program? More, less or the same?

Interviewee: It’s hard to say. I guess I’m more aware of it now you’ve asked the question. But perhaps. Yes, I would say ‘yes’ to that.

Facilitator: Good. We’re just trying to see how far the program’s reached and…

Interviewee: Yes, I understand.

Facilitator: We’re just trying to measure the reach. So did it reach anyone - did the message that we talked about reach anyone outside that classroom?

Interviewee: I would like to think so too. Possibly the husbands and partners.

Facilitator: Yes, which is great because that’s what we want. If you’re going to get women involved….

Interviewee: My husband just had his meniscus operated on today. He’s a healthy man; yes it is, he runs; he’s a farmer, he runs everywhere and has had a lot of trouble the last six months. But today he’s had his meniscus operated on, so that’s why I couldn’t talk to you before.

Facilitator: Okay, yes. Hope he recovers quickly.

Interviewee: Yes, but he’s a healthy eater and a healthy lifestyle as well.

Facilitator: But, and you probably both influence each other…

Interviewee: And I guess that’s really good because the people I see at school that are not part of the program perhaps, their whole family is – it’s sort of like a family thing, if one’s unhealthy the whole family is.

Facilitator: Yes, and it’s often the same way…

Interviewee: Yes, you don’t – another girl that’s on the program, Kate, she’s just really embraced it and her partner – like they’re just fighting fit at the moment.

Facilitator: Yes, that’s great.

Interviewee: Yes. And Penny, our principal, yes I guess so. Some of us have really…

Facilitator: Very fit.

Interviewee: Absolutely.

Facilitator: And the last think I want to just cover is about your satisfaction with the program. So based on your experience and what you’ve been in the program, would you recommend it to any other members in your community?

Interviewee: Absolutely, absolutely, yes.

Facilitator: And some people – we’ve had quite a lot of different answers to this – but in terms of the facilitator, did you like or did you have any opinion about whether the facilitator – so when you came it was Kate who did it – was local versus somebody external, do you have any preference?

Interviewee: No, I thought Kate was wonderful. That was a while ago and so much as happened since then but, no, I thought she was wonderful. The whole – no, it was very well handled.

Facilitator: Yes. And you wouldn’t change – it wouldn’t encourage you or discourage you whether the person was local or…?

Interviewee: No, not at all.

Facilitator: No difference?

Interviewee: No. I guess it depends on the personality more than where they’ve come from. Like the presenters were all perfect personalities for it. And look, you get someone local, it might be perfect as well but then it might not. So I think it’s more personalities and connectedness than where they’ve come from.

Facilitator: Yes. Where they’ve come from and probably their qualifications.

Interviewee: Hmm.

Facilitator: And what about the venue? Obviously it was at your school. Do you think that’s a good venue to run a Healthy Lifestyle session at?

Interviewee: I think that ours is a good venue now we’ve got the new building as the venue.

Facilitator: And do you have any preference for a group versus individual program? What would you – what’s ideal for you?

Interviewee: Look it’s sometimes good to do some parts individual and some parts group. It’s sort of good to have that rah-rah group mentality and then some bits, I guess, a bit more private, and it’s nice to have that individual…

Facilitator: Yes, so from the group it’s about – what you think it’s about, like sort of the support and the….

Interviewee: Yes. And look, I guess there are a couple on our staff that are quite overweight, you know what I mean. There are some things I don’t think they’d like to share with the whole group. I guess you can meet your targets and your goals, you can celebrate and I guess shout it out loud from the top of the building, but you know what, I think it might be more sensitive to take an individual approach.

Facilitator: Yes, because that’s what we found, that some people didn’t feel very comfortable in the group and it was a big deal for them to even come to the group because they felt self-conscious.

Interviewee: Yes.

Facilitator: So you think individual for the people who are self-conscious?

Interviewee: Yes. But I’m certainly not. I’m lucky that I’m not one of the bigger – you know what I mean. It’s always hard.

Facilitator: You don’t need to be, you’re very active.

Interviewee: I’ve been into PDs where they’ve talked about obesity with obese people in the room and I think it must be so hard for them. It’s almost like they’re talking about their health issues and things associated with obesity and you think, ‘These people are living it and I’m sure they don’t want it…’ – you know? They know how bad it is but they can’t obviously do anything about it, it’s just the way they are. I think that must be really hard being in a session like that when you have that problem yourself.

Facilitator: Yes, and I remember, there were some of your staff, as you said, some that do have, you know – so it could be hard.

Interviewee: Yes. That’s right.

Facilitator: And last question: If we were going to run this program again – which Kate actually will be running it across Victoria in the next couple of months…

Interviewee: Yes.

Facilitator: Any suggestions of how we could make it better? Would you like more ‘phone calls, less contact, more contact?

Interviewee: Look I know for me it’s not the issue but I received something in the mail that was a gift voucher, okay?

Facilitator: Oh, yes, yes.

Interviewee: A random one, like “Oh wow!” you know? People like rewards. I know it’s all about money and things like that, and I was one of the lucky ones but when I went to spend that, it was like, “Oh, wow, it was because of…” It doesn’t have to be a lot but the drink bottles we got, all those little things and unfortunately these days that’s what people like; they like rewards and things. It doesn’t have to be monetary, it can be anything, but those little rewards along the way I think are really important.

Facilitator: And there’s been – I might tell you but being part of this interview you actually do get a reward. It’s an incentive gift card…

Interviewee: That wasn’t part – you know what I mean? Like I remember spending it and either way I got, “Oh look at this. You can win door prizes and…” Bought you though some clothes, I hope some nice clothes. It’s not all about that but these days that’s what people – those random surprises or – even the drink bottles, they’re great drink bottles.

Facilitator: Yes, yes.

Interviewee: Those little things. People like something tangible really.

Facilitator: But you’d keep it the same way? You’d keep the text messages. Would you keep the…?

Interviewee: I think you’d have to. I think that’s the best way because, as I said, everyone’s got a ‘phone and that’s instant. Straight away it’s like, “Oh, yes, I must remember to do that”. The website I admit I haven’t accessed because it sort of the last, you know, with work. I’m doing work, so it’s the last thing I think of, and I think I had a fairly good basis as well to work from. You know, it would be nice to meet face to face again with the trainers.

Facilitator: Yes.

Interviewee: Is that going to happen at the end of the program?

Facilitator: Yes, that happens at 12 months, but a lot of – some women have also said that they would like it – with their feedback they said a six month one would have been ideal.

Interviewee: Yes, I’d [overtalking] let them know personally how we’ve gone.

Facilitator: Because they said it would help them with their contact.

Interviewee: Yes.

Facilitator: But okay, that’s good. Anything you didn’t like about the program?

Interviewee: No, not really. I thought it was very well run, by women, which is always a well-run project, isn’t it? I shouldn’t say that, but no, it was well run, professional, personal.

Facilitator: Great. Perfect.

Interviewee: Yes.

Facilitator: Well thank you so much for your time and your help. Much appreciated.

Interviewee: Oh, much – I hope it’s not all about me, me, me.

Facilitator: But that’s the whole point, it is about you because we want to know about your experiences, so we can deliver the program all we like but we want to know how people found the program, if they liked it. So it’s really valuable.

Interviewee: I just can’t understand obviously the people who let themselves go and not like sport. That’s what I really find – it’s just my mindset. I look at some people and I think, ‘I can’t believe you’re eating that’, or ‘I can’t….’ Look, there’s always treats and I can eat it too but, ‘I can’t believe you don’t want to come and play basketball with me tonight. I can’t believe it.’ And I know everyone’s different and different – yes.

Facilitator: Different priorities unfortunately and some people…

Interviewee: It’s even just, I don’t know, motivation, what they think is important in life but I don’t think they realise until they get to 60 or my age even, unless you look after yourself it’s a pretty awful you know. All those health issues that you can get if you’re overweight, if you’re obese at my age, so especially around the menopausal years, you’re so much better if you’re healthy.

Facilitator: Of course, yes. And it delays any further health problems and…

Interviewee: And it’s okay when you’re 20 or 30 and think, ‘Oh, it won’t happen to me. It won’t happen to me.’ Well it does when you get to 50.

Facilitator: And it can when you’re at 25.

Interviewee: That’s exactly right but it can make your old age quite miserable, I think. Or it could make it exciting…

Facilitator: More bearable.

Interviewee: I keep saying, I play basketball with 75 year old men. There’s this 70 year old men’s team and I think, ‘Wow, that’s just amazing.’

Facilitator: And when you say marathons and you say 85…

Interviewee: I know. You just see people and you think, ‘That’s the way it should be.’ I mean, look, you can’t – I don’t have….

end of transcript

Participant #AW

full transcript

Interviewee: ...most amused by her on our phone.

Facilitator: That's okay, no problem.

Interviewee: Hello. Then I realised what I'd done.

Facilitator: Oh that's okay no problem. Sorry, in terms of other factors that motivated you is there anything else that you can think of?

Interviewee: No, like you said, just a general interest and looking for information and yes...

Facilitator: In terms of – as you would have seen in Wonthaggi we had a pretty good response but not quite the response we were hoping for. Was there anything that you think stands out as to why other women wouldn't have attended?

Interviewee: Perhaps it's being busy. Yes, and perhaps because it was sort of in the day time that I came to that session and I sort of looked around and I thought gee I thought there'd be a lot more mums like me and then I thought well, yeah, I was just lucky 'cause I could ask my partner to be home on that day whereas others mightn't have that flexibility. That's the only thing I could really think of.

Facilitator: In terms of what you've learned at that program, in that Aboriginal – that healthy lifestyle session at the school, the lady talked to you both about some general health information and then focussed on behaviour techniques like goal setting and action plans and we all wrote an action plan together. Was any of that information new to you?

Interviewee: Yes, definitely. A lot of it actually. The whole idea of the small steps can really intervene in that weight gain type thing. And how little it can kind of take to just to sort of at least maintain the weight you're at.

Facilitator: And the messages that we talked about, was any of that new to you about the soft drink or about...

Interviewee: Yes there was lots of little bits of information, definitely. You know a glass of champagne is equivalent to a bowl of ice-cream and something like that and oh my god!

Facilitator: And what about in terms of like the goal setting and problem solving, is that information new or something you were already doing?

Interviewee: It was probably just more of a good reminder I think. It's something that it kind of all makes sense but when you see it all in the book like that, it's really useful.

Facilitator: In your impression, what do you think the take home messages were from that program?

Interviewee: To keep on top of it I think because it does require constant evaluation I think. You can kind of fall off the wagon really easily in life so I think just to make it a lifestyle change.

Facilitator: Since joining this program, do you think you've thought any more about what you eat, how much you exercise or about your weight?

Interviewee: Yes, definitely. It has – because I really like refer to the book and it will be good in the future just to have as a reference. Yes.

Facilitator: In terms of the components of the program we had the book as you've mentioned, we had the SMS messages, that initial health session and not long ago you got a session by the dietician that rang you – was any of that more useful to you than another part of it?

Interviewee: The messages were certainly good. They do kind of give you a kick up the bum. Yes, and they were helpful hints as well. So, yes, that was good.

Facilitator: What about – you said the manual – a lot of women said they used the manual initially but then didn't refer back to it. How have you gone?

Interviewee: Yes, I like it because maybe not always doing action plans or anything like that but just really the general information at the beginning, yes, I just find that really useful just to keep going back to and remind myself of healthy eating and physical activity and yes, it was good.

Facilitator: If we were to take away any of the components, is there any part that you'd recommend removing or do you think it all works well together.

Interviewee: Yes, I thought it was really good actually, I think it's really to the point. The book's not too huge or anything and it seems to flow really well so yes, I liked that.

Facilitator: What about the phone session when the girl rang you to discuss an action plan and to set some goals? Was that useful for you?

Interviewee: Yes, it was. Yeah, she was helpful with that and with the goal setting and stuff, yes.

Facilitator: Since starting, how do you think you've gone? Has your motivation been pretty good or has it gone up and down or how have you been going?

Interviewee: Definitely up and down but I do feel like it's on my mind a lot now like I don't think I'm going to forget about it and I know what to do to fix it sort of thing.

Facilitator: That's really good to hear.

Interviewee: Yes.

Facilitator: Is there any specific changes that you've made to your behaviour? To what you've been eating or your exercise? Any specific changes?

Interviewee: Yes, probably more the healthy eating because I know I do like lose motivation, especially in winter with the exercise stuff but with the healthy eating I feel like much more on top of that because it's something that I've got to do for the whole family so I'll obviously have their interests and I've got to put myself in there as well. So yeah.

Facilitator: Any specific changes that you can think of specifically like reducing junk food, more fruit, anything that stands out?

Interviewee: Yes, definitely. Probably the most specific is just buying less convenient sort of snack foods and junk foods in the house and just having more fresh produce.

Facilitator: In terms of motivation, you mention that that's fluctuated but has that been sustainable or sort of gone up and down do you think in terms of buying those healthier foods?

Interviewee: Yeah, I think the exercise motivation is probably what makes me kind of fluctuate, it sort of goes hand in hand doesn't it. [overtalking] so active then you tend to maybe go for the more comfort foods or whatever but yeah, I feel a lot more aware of it at least and as a whole a lot better.

Facilitator: Yes, because a lot of women said that they came away from that initial session quite motivated and then it dipped a bit but then it sort of has gone up and down.

Interviewee: Yeah.

Facilitator: Is that something that you've experienced?

Interviewee: Yes I have experienced that but I definitely have improved as a whole.

Facilitator: That's great. Can you think of any strategies that have helped you? What we're trying to work out is how you've done it and then relay that information other people that haven't been as successful. How have you made these changes do you think to your – you mentioned that the biggest one you've made is reducing convenience foods. How have you done that?

Interviewee: Sorry? How have I...?

Facilitator: How have you been able to reduce those convenience foods?

Interviewee: I guess just being committed when I go to the supermarket and just try and be a bit more wary of that when I'm shopping and maybe a bit more meal planning as well so that I don't get caught out for a meal and just revert to – these things or something like that.

Facilitator: In terms of you did mention that you've done the action plans and some of that goal setting, do you think you've done anything like that – maybe not necessarily written it down but have you used any strategies like that do you think? Or more just...?

Interviewee: Yes, well with the healthy eating definitely and probably like at first it was the exercise as well. I really focussed on that for a while as well.

Facilitator: Has that been achievable through goal setting or more just making it a priority?

Interviewee: I think so and also just the weight checks as well, like that has definitely become a part of my week now I'll get on the scales and just make sure that I'm monitoring that as well.

Facilitator: Do you find that motivating – that weighing yourself?

Interviewee: Yes, definitely. Yeah you note that, you know, I’m due for that walk or whatever, get out with the kids or whatever, yes.

Facilitator: It sounds like you've made some really good changes but do you think there's something that's helped you – have you ever tried to do any of this in the past?

Interviewee: Do any of it?

Facilitator: Yes, like change your diet or change your activity in the past?

Interviewee: Yes, I've always just thought that I needed to exercise more and I haven't really – like I've thought of junk as just being the treats or the takeaway but I haven't really had my head around you know it being the sugar in the coffee or alcohol or all of those hidden type things. So I've found that useful.

Facilitator: Is there something that's helped you make changes this time versus attempts in the past?

Interviewee: Yes, I think it's just an overall combination of everything like just realising that it's all those things – it's drinking the water, getting the exercise, watching what you drink and eat and yes, a bit of everything makes it as a whole.

Facilitator: Is it as you said that bit of everything – has that been through being involved in this program or is something else changed in your life that's made you more aware and be able to make these changes do you think?

Interviewee: Yes, I think it has really helped. It's probably things that have – like I said I've probably known it all but it has sort of prompted me I think and perhaps having a family now with having the kids has made me think that I've got to do it for them as well. Yes, so probably that combined with yes, the knowledge and the boost from you guys.

Facilitator: That's really interesting what you said about your family – that's your motivation and that's something we've heard a lot, is there anything else that motivates you to be healthy do you think?

Interviewee: Yes, I think I've just realised that being active helps with everything in life. Your motivation and it all makes you feel better, being healthy and active so I think that's my motivation. I just want to be the best that I can be sort of thing.

Facilitator: As you said, looking after your family – is role modelling for the children important to you do you think?

Interviewee: Yes, definitely.

Facilitator: And what about support? Some women have talked about also being able to make positive change because they felt supported by their families and friends – what's that been like for you?

Interviewee: Yes, really good. My partner's also sort of interested in his own health and for the family and stuff and so we've kind of worked together on that which has been really good because he used to be a real junk sort of fanatic and now he's really as a team we've sort of both improved. So it's good...

Facilitator: That's fantastic.

Interviewee: ...support from him.

Facilitator: The next thing I just wanted to have a quick look at is what we're trying to work out is whether by you being involved in this program it's influenced anyone's behaviour like your families or your friends. So you mentioned that since being in this program you've been eating more healthily, do you think that's had any effect on your family?

Interviewee: Yes, well my family just within the household definitely because I have an impact on what they eat and what they do but also like I've spoken to my mum and my sister, especially when I first did the very first session and I actually have talked to them about it a bit and I've probably passed on a little bit of positivity yeah, to them.

Facilitator: That's great and you said that you've affected your family's choices – any specific examples of how you've changed what you cook and buy for the family?

Interviewee: Yes, just the fresh produce and reminding myself that the kids need to eat you know five veg a day and having that in my mind and always having lots of water sitting around, bottles of water for them and yeah, just the meal plans I guess always.

Facilitator: Just to confirm you said that that's been pretty sustainable over – you said your motivation has, as most people would say has dipped but in terms of the changes to what you've been eating, that's been pretty consistent?

Interviewee: Yes, that's right, the eating part I feel like I've got control over fairly well for myself and my family, probably mostly it's just the exercise motivation where I fall down.

Facilitator: Of course and we can't all be perfect can we?

Interviewee: No.

Facilitator: You mentioned – sorry the other thing we're looking at is in terms of whether you've spoken about this program with any of your friends or anybody else outside your family? Some women said they have and others have not – how's it been for you?

Interviewee: Yes, I think I would probably talk about it more but because I'm rather tied to my kids I don't actually get much social life unfortunately. But within my family I certainly have.

Facilitator: That's great and the last thing I wanted to talk to you about is just about your satisfaction with the program and whether, based on your experience in this program, you'd recommend it to anybody else outside in your community or anybody?

Interviewee: Yes, I definitely would I think it's fantastic and just like the fact that you held it in the first place, I thought oh wow, there's people that are actually out there to help and yes, it felt like a privilege. Yes, it was good.

Facilitator: I'm glad to hear you say that. Is there any other reasons why you'd recommend it?

Interviewee: Yes I found just the facts in the book about it rather kind of dismal for women like [laughs over] and stuff like that and I just think yes, it's just so important to sort of take on things now or as early as possible and it gets too hard.

Facilitator: Would you say overall you'd be satisfied with the changes you've made with this program or do you think there's still room for...?

Interviewee: Definitely.

Facilitator: In terms of the facilitator that ran the program, do you prefer whether that's someone that's local or someone from Melbourne?

Interviewee: I don't think it would matter. No, I don't think it would matter at all.

Facilitator: What about the group session versus an individual one – do you have any preferences?

Interviewee: NO, I think the group thing is fine but then I haven't been in the area for a long long time. Maybe if I'd been here a long time and I was amongst a room of locals who I'd known – who I all knew it might be different but to me it was fine.

Facilitator: Did you find that – some people in the group session said they liked it because it felt like other people knew what they were going through and that they had someone they got to learn and get ideas from other women – did you find that useful?

Interviewee: Yes, that's right. I thought everyone opened up and contributed a lot and yes, it's good to hear other people's stories. Yes.

Facilitator: Do you think – again we've had really mixed responses about this but have you seen or spoken to anyone else in the program since that session back in October last year?

Interviewee: No I haven't but I've seen someone but I haven't spoken to them about it but I remember them in the program.

Facilitator: One woman suggested like you she hadn't seen anyone in the program since the initial group session that something like a Facebook forum or another visit to reconnect with the group would be useful.

Interviewee: Oh yeah.

Facilitator: What do you think or do you think you're quite happy with where you're at and you wouldn't need any further support?

Interviewee: Yeah, I personally would be happy with what there is but yes, maybe another form of media and connection would be good for some people, yes.

Facilitator: So overall, do you think you were satisfied with the amount of support provided or would you have liked more?

Interviewee: Yes, I thought it was enough. Yes, I was satisfied with that.

Facilitator: Good and the last thing is, what about the venue? A lot of women – some women really liked the venue being at the school and other women said that if they didn't have kids at the school they didn't think it was a very ideal venue. What's your opinion?

Interviewee: Yes, it worked fine for me but I guess – it was fine for me. I can't think of a downfall for it really.

Facilitator: Sorry, officially the last question is based on your experiences, is there anything you think we could do to improve this program in the future?

Interviewee: well I thought it was fantastic but like you said perhaps there weren't as many people as there perhaps could have been at the initial session. Maybe something along that line but I wouldn't be an expert to suggest any way to improve that but that's the only thing I could think of.

Facilitator: Are there any tools or anything else you think would have helped your motivation at all, that we could have done or anything that springs to mind?

Interviewee: No, I thought it was all really good. Yeah.

Facilitator: Anything else you think we need to know about Wonthaggi as part of our evaluation or...?

Interviewee: No I don't think so. Nothing springs to mind but...

Facilitator: well thank you so much for your time and if you think of anything else that you want to discuss or you need any further help please feel free to contact us. Everything you've said has been really valuable so...

End of transcript

Participant #SA

Full transcript

Facilitator: To start off with, what I’ve been doing is just asking women to talk a little bit about their town because we’re trying to compare all the different towns to each other, but in Yarram, what’s your perspective about the amount of fruit and vegetables available and exercise opportunities?

Interviewee: The fruit and veg is all good because we mainly – oh, we do go over to the Valley sometimes, but when we run out of course, we run into town and get it, so that’s pretty good. The exercise part, I suppose you can do quite a bit if you actually look around and think about what you want to do. The only thing is – like, I swim a lot – but I can’t swim during the Winter because the pool’s not open, unless I want to travel about 80 kms to the nearest pool.

Facilitator: Yeah, so that’s pretty far.

Interviewee: It is when you’ve got kangaroos jumping out at you.

Facilitator: And in terms of walking, is there good walking paths?

Interviewee: Yeah, there is now. They’ve built a railway track, on the old railway, and we can go pretty much to the next town – if you can call it a town.

Facilitator: And would you consider Yarram overall, to be quite an active town or not particularly active overall?

Interviewee: Pretty active. I think especially around this time with the football and the netball.

Facilitator: So quite a sport focused community?

Interviewee: Yeah.

Facilitator: And in terms of socialising, some towns we’ve been to have been quite connected – the women have been quite connected to each other – whereas, other towns have been a bit more isolated. I know you don’t live in Yarram as such, but what are your thoughts on Yarram?

Interviewee: I think it is. If you want to get out there and be social, there’s heaps of little things to do, but I’m not really; I’m a bit too busy to even bother about being social. I mean, our footy club, we do every Thursday night – we’ll go out for tea, that’s the football club, and that’s pretty social so that’s our main big thing, and then the football on the weekend.

Facilitator: Okay, cool. If you think back to when you started this program back in about September/October last year, do you remember what motivated you to join this program?

Interviewee: I just saw it in the “Chatterbox”, the school paper, and I thought “Oh yeah, why not?”. I was interested too because for the last few years I’ve been swimming, and then somebody at work said “Have you ever worked out how much you actually lose when you swim and what you put on when you’re not swimming?”, and I went “No, I haven’t even thought about it”. So it sort of coincided with that, and I thought “Well, I can then look into seeing what else I can do”.

Facilitator: So from what I’m hearing, was it more to learn a bit more?

Interviewee: Yeah.

Facilitator: What about support to maintain your weight or lose weight? Was that something that occurred to you?

Interviewee: No. For me, I’m not really that fussed on the weight side of things because that’s why obviously with the swimming I didn’t even think of it, but just out of interest. I was more interested in foods and what maybe I can do better there.

Facilitator: And at that group session in Yarram, we had a pretty good response, does anything stand out to you as to why other people in the community may not have been able to attend?

Interviewee: I think there were a few that couldn’t because of work. I know one came in afterwards, she said “Oh yeah, I’ll come in as soon as I finish work”.

Facilitator: But nothing going on around that time?

Interviewee: No, I don’t think so. I can’t think back that far.

Facilitator: I know. It was quite a while ago now.

Interviewee: Yeah.

Facilitator: And thinking back to that, when we first saw you and we’d also provided you with that manual, based on what we’ve given you, we talked about in that first session general health information about food and exercise, and then we also talked about behaviour change, strategies like goal setting, problem solving, and we all did an action plan together, was any of that information new to you?

Interviewee: No, I don’t think so. I suppose if I’d looked into it a bit, I would have found something, but no, I think I’ve seen some of that on tele with all that sort of stuff, so it wasn’t that new.

Facilitator: So most of the health information about the different messages, most of that was quite familiar to you?

Interviewee: Yeah.

Facilitator: Did you find that a useful refresher or you already kind of knew it?

Interviewee: It was good to refresh, and also when I went through the book, I thought some of my goals, I know I’m going to achieve anyway with swimming, but it would be after the swimming, that’s when I needed to refer back to the book to get the motivation to go, because at the moment I can only do three days a week of walking and stuff, but with my grandson at the moment, today’s gone so maybe tonight I can try and get an hour in or half an hour, so I’m just trying to aim for that. At the moment we’re also trying to buy a house and move house, so I’m not going to have a lot of time to be doing the walking, but then I suppose with lifting and moving, I’m going to get the exercise in then.

Facilitator: Yeah, good. So for you, the main thing you’ve been focusing on is exercise, is that right?

Interviewee: Yeah – well, part exercise. When I’m not swimming I try and work on other exercise, and then when I’m swimming, it’s more the foods and keeping healthier foods.

Facilitator: And in terms of increasing that exercise, you mentioned before that you were setting some goals, was that something you were doing before you started this program or as a result of this program do you think?

Interviewee: It was before, especially with the swimming. Yeah, I’d been doing it for, I think, about four or five years now.

Facilitator: And what kind of goals were you setting?

Interviewee: Well, first of all, the first season was 3000 and see how I go, and then the next was 4000 and then the next year I just sort of went “No, I’ll still just cruise at 4000”, and then for – not last season, the one before – one of the women said “Well, you’re nearly up to 4000, why don’t you keep going?”, and I went “Oh, I don’t know”, and then I went “Okay, fine, I’ll do 5000”, then did another 5000, and then I sort of struggled only because of the freezing cold water towards the end of the season, and I actually went up to Sale which would be 80 kms away, into a nice heated pool but I did 250 laps in one go.

Facilitator: Oh, my God, wow.

Interviewee: Yeah, a tiring exercise. But this last season, yeah, I was able to cruise in that 5000 no worries.

Facilitator: Fantastic.

Interviewee: Yeah, so it was good. I’ll see how I go for the end of the year whether I start up and do a bit more. I’ll be able to spend a bit more time at the pool because I won’t be travelling out of town, I’ll be living in town, so I might be able to get in an extra 10 or 20 laps before having to rush home and get kids organised.

Facilitator: That’s good, yeah.

Interviewee: One of the kids is in the swimming club in Sale and we’ll have to travel up there twice a week, so that’ll give me two days a week swimming…

Facilitator: To use the pool?

Interviewee: Yeah, to use the pool – that I probably wouldn’t have done before. I mean, when we do swimming, I’ll usually do one day a week up there with them for their lessons, but this time it’s two days.

Facilitator: So it’s good use of time. And since you started the program – again, we just want you to be honest as possible because there’s no right or wrong answers – but do you think it’s made you think a little bit more about how much you exercise, your weight or what you’re eating, or no change?

Interviewee: Oh, there was a change, yeah definitely. Well, I suppose, to get back to the swimming, when I’m not swimming, it’s exercise, it’s finding something else to do in the time that there is no swimming pool open.

Facilitator: So you mentioned you found other activities?

Interviewee: Yeah.

Facilitator: And again, was that behaviour change – did you use any of those things like that action plan, problem solving, or it was more you just found something else?

Interviewee: Well, I knew that what I could do out here was to walk and then I thought “Well, I’ve got the treadmill. We can do that”, but knowing when we move into town we’re going to try and save petrol, and walk or ride. So I know that when we get into town I’ll have the extra walking and riding so it won’t be such a struggle to go “Right, you’ve got to find the time”, because it will be happening already.

Facilitator: So it will be that incidental exercise?

Interviewee: Yeah.

Facilitator: And you also mentioned the changes to what you’ve been eating. What changes have you made do you think?

Interviewee: Well, a couple of years ago I found out that I had a gluten and lactose intolerance, so I’ve really got to – and with this program, I was starting to think “Well, maybe I shouldn’t be just going for the easy packaged options and start thinking about healthier vegies and salads and stuff”. We actually went down on the weekend and bought a heap of salad, but it’s just stuck in the fridge at the moment because I haven’t had a chance to have a look at it and make it up.

Facilitator: So it’s more about increasing fruit and vegetables?

Interviewee: Yeah. I get a lot of fruit because I make up smoothies and we’ve just found a little ice cube thing to go in the smoothie, so that has vegies in it.

Facilitator: Oh, great.

Interviewee: Yeah, it does help when – and, you know, three to four times a week we try and get the vegies in with sports and kids running around and…

Facilitator: Yeah, so with a busy lifestyle. And just to confirm, that’s been a bit more since starting the program?

Interviewee: Yeah, I think we’re just looking at it definitely a lot more. Before, it was like, yeah we know, but haven’t got…

Facilitator: Of course. And in terms of motivation – so you mentioned that you’ve been doing a bit more exercise and eating a bit better, has that motivation been sustained since starting this program or do you think it’s fluctuated a bit?

Interviewee: Depending on what’s happening, yeah, it’s sort of gone up and down a little bit, but because we’ve got the book and the little text messages, it’s in the back of my mind a bit more.

Facilitator: And that sort of keeps it on your mind?

Interviewee: Yeah, like “Okay, I’ve got to do that”.

Facilitator: And if we were to look at the specific components of the program – so we had the group session back in Yarram, we gave you the manual, the text messages, and not so long ago you also got a phone call by a girl just to give you a bit of support and you wrote an action plan with her, what’s been the most valuable or useful to you do you think?

Interviewee: The whole lot.

Facilitator: Yeah?

Interviewee: Yeah, especially the book and just the [overtalking] and stuff like that.

Facilitator: And that book, did you fill out the activities in that book or just more read it?

Interviewee: No, I did both. At one stage there I was able to do it all and go through the goals and stuff, but I have to now go through it a bit more and just go over everything just to remind myself and get back on track.

Facilitator: That’s fantastic. So it sounds like you’ve been using it sort of intermittently since starting?

Interviewee: Yeah.

Facilitator: And you said the text messages were good?

Interviewee: Yeah, because there are times when you just get caught up with life and then all of a sudden you go “Oh yeah. Okay, I’ll do that”.

Facilitator: And what about the phone call? Did that help sort of focus you again or give you a bit more support?

Interviewee: Yeah, both. I was able to sort of go through a few things I wasn’t quite sure about or whatever.

Facilitator: And just to clarify again, do you feel that you’ve been adequately supported in this program or do you think you needed more or less? What’s your opinion?

Interviewee: I think it was pretty good.

Facilitator: So support, you were quite happy with the level of support?

Interviewee: Yeah.

Facilitator: And I think you mentioned, just to clarify, your motivation, has that been quite good to keep eating healthy and exercise?

Interviewee: Yeah. Of course, you have your moments but yeah, I think I’ve done pretty well.

Facilitator: And is there anything that we could have helped you with, you know, providing more support or more information, that would have helped increase your motivation?

Interviewee: Mainly I suppose for the foods with the intolerances. I’ll sort of look at the other books and you think “Oh, it’s just too fancy”. I just want something really simple and easy to make up because when you get hungry, you think “I can’t be bothered spending the next half an hour or something trying to make something”; you just want something quick and easy in the fridge. But yeah, I’ve just got to try and think of other options to do and maybe make them up on the weekend if I have time to do some sort of salad that can sit in the fridge and I can just keep going back to it, and remember that I can just go and get a boiled egg or something.

Facilitator: Yeah, so something easy.

Interviewee: Yeah, some sort of little meal that’s just quick and easy because most people can just go and whip up a sandwich, but I can’t.

Facilitator: It sounds like you’ve made some really good changes, is there anything that’s sort of motivated you? We’ve had some people that have asked us for the motivation pill as such, what’s been your motivation do you think?

Interviewee: I don’t really know. I know, like, with swimming it’s because I really enjoy it and it’s just something that I like to look forward to and stuff; towards the end of the season you sort of get a bit sick of it. Yeah, I don’t know.

Facilitator: To be healthy or is it as a role model for your children? Anything that stands out?

Interviewee: Probably both of those because, as I said, I want to be healthy and be able to run around after the kids and keep up with my grandson at his crawling stage.

Facilitator: You said you’ve increased more fruit and vegetables and it sounds like you’ve upped your exercise a bit, if you were going to – we’re just trying to give some tips to other women involved in this – can you explain just a little bit about how you’ve done that? So how have you made the more vegetables, you mentioned you buy more from the market, anything else?

Interviewee: No, we’re just putting in a lot of other foods, like, we sort of add them to other things.

Facilitator: And again, just to clarify, a lot of the changes have happened since starting the program?

Interviewee: A few of them, like, with the exercise, trying to think about something else other than swimming. I know last year after I finished swimming, I pretty much just went “No, not doing anything. I’m sitting back and relaxing”, but I did have a crap year anyway before, which is probably why I just went “No, I’m not going to bother”, and I did put on about three or four kilos doing that, but then I knew I had swimming coming up eventually at the end of the year. But just knowing that the treadmill’s there and I can use that, and I thought “Well, it’s colder months, there’s less snakes out”, so I’m going to be more likely to walk down to the letterbox.

Facilitator: And in terms of the changes that you’ve made about what you’ve been eating, are there any reasons why it’s been easier to change it now versus in the past?

Interviewee: No, about seven years ago we changed because we knew both sides of our family has the diabetes and thought “Well no, we don’t want it, so let’s change it” which is then why I found out I was lactose and gluten intolerant because of a few little changes, and then I had to change that sort of thinking again. I think with the help of the book, I went “Okay, I really do need to think about less packaged stuff and more fresh”, and it makes me feel sort of better anyway. I know yesterday we had a family get together because it was Mum’s first anniversary after she passed away, and we put a plaque up and we had some food there, and I went “Stuff it, I’m just going to have a slice of cake”, which I can’t eat normally, and now I’m paying for it today because I’m in pain, so I’m just like “Well, that’s what happens”. So yeah, I do have to be a bit more aware and sometimes I think because I’ve been so good, I’ll just eat it because it won’t react, and then I react. So yeah, you know you’re going to react with it. But it has helped with my weight because I don’t go for pastries and stuff, but with some of the gluten free things, it seems to be more packaging with pizzas and stuff, but a lot of the stuff I put on the pizza is always a lot more healthier than actual other – yeah, I was starting to think packaging stuff biscuits gluten free might be a little bit healthier, but it’s still not the best and I’ve got to try and come up with some interesting snacks.

Facilitator: It sounds like it’s all going pretty well.

Interviewee: Yeah, I think so, although I don’t feel like I’ve lost a lot of weight, but I don’t think I’ve put on a heap either.

Facilitator: That’s great. Have you been monitoring your weight?

Interviewee: No, because the only scales we’ve got are on the Nintendo Wi and I haven’t really been able to get on it lately.

Facilitator: And the next thing I wanted a quick look at is the program reach. So what we’re interested in is whether, since starting this program, you’ve influenced anything that your family have eaten or any of your friends? Do you think this has happened?

Interviewee: More family I think, and with us sort of upping the sports with the kids and everything, we are sort of thinking more about what we’ve got to do with the diabetes in the family, and I suppose with cancers and stuff like that on my side, then we’ve got to think of eating more healthy and trying to think of that. And I think – like, my husband’s definitely “Right, we’ve really got to look at it a lot harder and start putting a lot better foods in the fridge”.

Facilitator: So your husband’s very supportive?

Interviewee: Yeah, and he motivates me a bit more too because he’ll get out and work all day and then come home and get on the treadmill, and I’m like “Oh no, I can’t be bothered”, and then he’ll go “Well, I’ll do half an hour and then you’re on”.

Facilitator: Oh, that’s good. So you work together as a family?

Interviewee: Yeah. And as I said, when we move into town, the aim is that we all walk or ride to work and school.

Facilitator: Yeah, so you’re trying to increase the exercise all together?

Interviewee: Yeah.

Facilitator: And you also mentioned that, I assume by you buying more vegetables, that’s going to increase the vegetables for the rest of the family?

Interviewee: Yeah, we’re hoping so. One of the kids refuses to eat vegies, and one of the older ones, when they were younger, we were able to hide all the vegies in the mashed potato which was really good, but the youngest one will not eat mashed potato, so we’re trying to come up with other things. A lot of stir fries we’ve managed to get in, but occasionally he’ll go “Okay, that’s a vegie, I’ll just push that aside”, which is strange because he’s the only one, and the rest of us all eat vegies and we’ve all explained that most people don’t like them but you eat them because they’re good. We even tried to explain to him that when I was pregnant with his sister, that the smell of vegies would set me off and I’d be throwing up, but I said I’d have to sit down and eat the vegies with all of you kids and then as soon as I’d finished, I had to run to the toilet and throw them back up. Occasionally now, the smell of the vegies being cooked will set me off and I’ll say “But I still have to sit here and eat them even though I feel like throwing them back up”.

Facilitator: Yeah, so you’re trying to set a good example for him?

Interviewee: Yeah. And we’ve said to him “Look, if you have to, smother sauce on them or something or push them into the mashed potato” but no, he doesn’t like any of that.

Facilitator: How old are your children again?

Interviewee: The only two that are left at home now are the two youngest ones, who are 12 and…

Facilitator: Do you talk to them much about healthy eating?

Interviewee: Yeah, all the time.

Facilitator: And do you think that’s been the same, or more or less since starting this program?

Interviewee: I think probably the same, because the youngest one, he has always been a real stocky little kid, but my daughter’s a bony thing, like, one of my brother-in-law’s said when they were younger and they were running around naked at Mum’s house and he goes “Oh my God, what have you done? You don’t feed that one, and you feed that one too much”, “Well no, my daughter eats heaps, and my son sort of doesn’t”.

Facilitator: Yeah, just the genetics.

Interviewee: My son wants to eat a bit more but what he’s eating is not the healthiest. I try and make them make their own lunches and let them know that they’ve got to make sure it’s healthy choices in there and if they get stuck we just say “Well, there’s a wrap in the fridge, you can make up whatever you want”. He sort of goes through the ups and downs – he does it really well, and then he’s like “No”, and shove something in that’s really easy. They’re not very big sandwich eaters so I said “Well, you’ve got to come up with something else that’s healthy”, so we go through all the stuff in the fridge.

Facilitator: Well, it sounds like they’re getting some good healthy eating lessons.

Interviewee: Yeah. Whether they do it or not’s another thing.

Facilitator: And what about any friends or anybody else outside your household, have you talked to them about anything you learnt in the program, or showed them any of the program resources?

Interviewee: No, I haven’t really, other than the other girls that were there, I’d sort of come up and say “Have you done anything on there?”, and a few of them were like “No, we’re getting there”. There was one I was speaking to the other day and she goes “Yeah, I’m trying to get in a bit of walking”, but that’s more because she’s now out on the farm and running around after cows. Every now and then you bump into one of them and you’ll say “How are you going?”, but other than that, I haven’t really.

Facilitator: But it sounds like you’ve got a bit of support from them which is positive?

Interviewee: Yeah.

Facilitator: And thinking about your satisfaction with the program, based on the program this far, do you think you’d recommend it to anybody else?

Interviewee: Yeah, I think so.

Facilitator: Any reasons?

Interviewee: I think if they’re really wanting that extra help, it would give them that little bit of a boost.

Facilitator: And in terms of the facilitator that ran the program at the day session in Yarram, do you prefer whether that person was someone local or someone from Melbourne?

Interviewee: I think Melbourne, because some people don’t like to have people around you that you know telling you what to do if it’s somebody from town. And I think because of being in Melbourne you think they’re a lot more knowledgeable, you know, closer to all the information and more information you get quicker.

Facilitator: And what about in terms of a group versus an individual session? Are there any thoughts on that?

Interviewee: I think a group was good, but then some people might prefer the individual one. I don’t know what other people think, but I thought it was all right.

Facilitator: Some people talked about the group session as being good because you could hear what other people were doing and you could see that other people had the same sort of problems. Was that helpful for you?

Interviewee: Yeah, and then with others doing different exercises that maybe I hadn’t thought about, and the same with them. Actually, there was one woman – I don’t think she was there and I don’t think she’s doing the program – but she saw me and the kids talk about my swimming and that, so she’s now starting to do it, and she was asking questions actually on the weekend about the exercises and what to do with swimming.

Facilitator: That’s good.

Interviewee: I don’t think she was part of the program, but yeah…

Facilitator: That’s good, so it’s still helping somebody else.

Interviewee: Yeah.

Facilitator: And is there anything else that we could have done? It sounds like from what I’m hearing, you were already living a pretty active and healthy lifestyle, is that correct?

Interviewee: Yeah.

Facilitator: But is there anything else that we could have – any more information? You mentioned maybe a bit more on wheat and dairy free snack ideas, but is there anything else you thought was missing or that could have been useful?

Interviewee: I don’t know – yeah, you’ve got all the websites and…

Facilitator: Yeah.

Interviewee: Yeah, maybe exercise programs, like a little basic one that you could sort of add to, although you’ve got Biggest Loser on tele, a bit on that.

Facilitator: You’ve been watching it?

Interviewee: Oh yeah.

Facilitator: A lot of people we’ve met have been on the Michelle Bridges’ diet. Have you been on that?

Interviewee: We’ve had a look at it, but after you get to a certain point you’ve got to pay and we can’t afford to be paying out a huge amount. We’ve also looked on the Jenny Craig sort of thing, trying to get some ideas there, but a lot of the foods just seem to be really fancy and you think “Can I really find all those ingredients in town and is it worth it?”. You just want something that’s really easy and simple. We’ve even looked at that Lite n' Easy, but again, it’s the money. We don’t have a lot of money because we don’t spend heaps on food compared to most people because we just don’t go for top of the range brands and everything, so we do try and keep everything…

Facilitator: …a bit affordable?

Interviewee: Yeah.

Facilitator: And do you think if this program had a cost to participate, would that be a barrier do you think?

Interviewee: I think so, yeah, depending on the cost and whether it was ongoing. Because it was free it just made it a little bit easier I think.

Facilitator: Of course. And if it was going to cost, what do you think would be a reasonable price?

Interviewee: I have no idea.

Facilitator: No idea?

Interviewee: No, not at the moment.

Facilitator: And the last thing is, do you have any other recommendations about how we could improve the program or how you’d like to see it change?

Interviewee: No, I think it was pretty good, other than maybe plans for menus and that for coeliacs or people with intolerances, or just somewhere else where they can get something.

Facilitator: Information?

Interviewee: Yeah, information and maybe a little exercise plan because that way they can sort of start on a little bit and if they’re not interested, well they can try and find other things, because I was starting to think about doing weights training and sort of build up the arms and legs with some work, but then I’ve done my arm at work anyway, so I can’t hold onto the weights…

Facilitator: That’s a problem, yeah.

Interviewee: …so I’m thinking “Well, It’s going to be a bit hard”, and I thought “Well, how am I going to go walking on the treadmill?”, but I did pretty well, but my arm ached afterwards so I thought “Well, that can’t be an excuse, because it aches all the time, so don’t turn around and get off the treadmill just because it’s aching”.

Facilitator: Some people also mentioned that they’d prefer more contact, you know, another phone call, or another face-to-face session, would you like that or you’re happy with it as it is?

Interviewee: I think another face-to-face might have been pretty good.

Facilitator: And one lady mentioned that she thought a Facebook group would be good, or some way that she could talk to other people in the program to get support from them, would that be useful to you do you think?

Interviewee: No. I’m on Facebook but I’m just not on it enough – I’m not near the computer enough to sort of sit down because even today, I’ve got to find some time and get on and email the swimming club, and I don’t think that’s going to happen today.

Facilitator: Yeah, so just time would limit you?

Interviewee: Yeah. I used to have a bit more time and I used to get on Facebook and play and talk to people and stuff, but now, yeah, it’s just there. I might have a quick flick if I’m waiting for the kids at the bus stop, and that’s about it.

Facilitator: Is there anything else you think it’s important for us to know about Yarram, or anything else that you want to add that we might need to know for this evaluation?

Interviewee: No, I think that would be about it, that I can think of at the moment.

Facilitator: Well thank you so much for your time, and everything you’ve said has been really valuable to us. I’ll send you that…

end of transcript

Participant #CJ

full transcript

Facilitator: So did you receive the letter about the evaluation?

Interviewee: I did actually.

Facilitator: Good, perfect. So the point of the evaluation is just to get your feedback about the program, about how you found it, what you’ve learnt. We’re just trying to look at how many people the program’s reached. So I was wondering if we could start off with – the interview takes about 20 minutes. I’m also – just to – we’ll be tape recording that. Is that okay?

Interviewee: Yeah, that’s fine.

Facilitator: Perfect. It’s just to make sure that I accurately report what you say. To start off with, I was hoping that you could please describe your community in Wonthaggi. Just describe the town that you live in.

Interviewee: Small country town.

Facilitator: What about access to health services? Is there any – can you comment on that?

Interviewee: If there’s gyms and things like that?

Facilitator: Yeah.

Interviewee: Yeah, there’s gyms and women’s gyms.

Facilitator: Do you think that many women are active in the town?

Interviewee: Yes, I do.

Facilitator: Do you see groups of women walking or is it hard to find someone to walk with?

Interviewee: Oh, I do – you do see lots of people walking.

Facilitator: What about the age of your community? Is it quite a young population or older?

Interviewee: More older.

Facilitator: In terms of health services, do you think there’s lots of opportunities to get access to doctors and health professionals?

Interviewee: Yes.

Facilitator: In terms of employment, is there many opportunities to be employed in the community, in your opinion?

Interviewee: No, not really. Pretty much who you know, not what you know.

Facilitator: Have you lived in the community for a long time?

Interviewee: No. I’ve been here probably two and a half years now.

Facilitator: Any other impressions about the town that you can think of?

Interviewee: It’s a nice lifestyle. I really enjoy it down here.

Facilitator: When we started – we came to Wonthaggi a couple of months ago now, do you remember why you decided to join the Healthy Lifestyle program? What motivated you to join the program?

Interviewee: I think because I’m just always conscious about looking after myself and things like that. So I thought “why not?”

Facilitator: Is there anything particularly that you’re conscious about?

Interviewee: Probably just maintaining weight.

Facilitator: Were there any other factors that helped make coming to the program easier for you?

Interviewee: Probably because it was an easy access. Like you guys came to school, I guess that made it easy.

Facilitator: Your kids go to the school?

Interviewee: They do. I have a daughter that goes to the school.

Facilitator: Did you hear about the program through the flyers? Or how did you find out about the program?

Interviewee: Just through the school and the flyers that they had.

Facilitator: We’re interested to see whether women came in pairs or with groups of friends. Did you come alone or did you come with some friends?

Interviewee: Oh, I came by myself.

Facilitator: Another thing that we’re interested in is what factors made it difficult for some people to attend. Were there any factors that stand out to you about what would make it difficult and how it would be difficult to come to the program?

Interviewee: I guess it just would be depending if you worked or not.

Facilitator: What about priority? Do you think that the women in Wonthaggi would view health as a priority in general?

Interviewee: Yeah, I’d say there’d be a big group that would. But there’s also a part that doesn’t seem to be phased.

Facilitator: So you think the timing. Anything else that may have prevented other women from not coming?

Interviewee: Probably just having young children, I guess having to keep them at home.

Facilitator: Childcare. Overall, as you would know, the program was made up of a health – the first Healthy Lifestyle program at the school, the manual, the SMS’ and the websites. Since starting the program, was there anything new that you’ve learnt particularly or any information that you’ve retained?

Interviewee: Just being conscious, I guess, would have – exercising and watching what you do eat.

Facilitator: Has this changed since you started the program or has that always been a – you’ve been conscious of that always?

Interviewee: Oh, I’d say I’ve always been conscious of it. But just some habits you can get into. Being able to just keep those in check.

Facilitator: That’s important to you?

Interviewee: It’s – very much so.

Facilitator: Great. If you were to finish my sentence – so if I said to you “if you were going to describe the Healthy Lifestyle program to a friend …” – so if I said to you “the Healthy Lifestyle program that you attended was about …” – what would you describe the program as being about? What do you think the main focus of the program was?

Interviewee: More about just … probably just more about organising – what you organise for food and stuff and just maintaining a, you know, well-rounded lifestyle. With activity plus healthy eating.

Facilitator: Was most of the information that we discussed new to you or was it a refresher or some of the information was quite new to you?

Interviewee: Oh no, I’d say a refresher. I think part of different things that sort of relate to health and exercise. So if it’s when it’s come to you and it’s someone talking to you about it. Sort of just more gets you to get back on track and stuff like that. So a refresher.

Facilitator: You mentioned that you’ve been more conscious of your health behaviours. Is that – did you say, sorry, it was since you started the program? Or it hasn’t changed since you started the program?

Interviewee: No. Since starting the program.

Facilitator: Do you think more about food or exercise, typically? Is anything more important to you?

Interviewee: I’d say probably they’re just – I put them together as the same thing. They’re both as important as each other.

Facilitator: Part of the evaluation is that – I'm trying to look at the specific components of the program and work out what you found most useful. So the program was – we had the group session, the manual, the SMS’ and the website. You recently had a phone call as well, to help motivate you. Was any specific component more valuable to you than any of the others?

Interviewee: I’d say the SMS’. Because they obviously keep you reminded of what you’re a part of. And the book, I guess, too. You can always refer back to it and the steps and stuff.

Facilitator: Have you been referring back to it often?

Interviewee: If I was to say yes, that would be lying.

Facilitator: That’s okay. But that helps you keep on track?

Interviewee: No, that said. But that seems more – when you just get the SMS’, it just makes you sort of aware, okay? Like someone’s out there watching you, being harder with you.

Facilitator: Do you find that motivating?

Interviewee: I do, actually.

Facilitator: What about the phone call? Was that helpful to you to talk with someone about your own health goals?

Interviewee: Yes. Yeah, I found that helpful. But then you don’t sort of feel like you’re doing it by yourself.

Facilitator: Do you use the website?

Interviewee: I haven’t been on there.

Facilitator: Do you normally use the internet to access health information?

Interviewee: Yeah, I don’t have – I do sometimes. But I don’t have a computer at home.

Facilitator: Makes it a bit harder.

Interviewee: Yeah.

Facilitator: So if we were going to change the program, in order to get you as motivated as you are now, do you think if we took away any elements of it, it would make a difference? So if we were to take away the phone call, would you be as motivated as you are now?

Interviewee: Possibly not.

Facilitator: So it all helps together?

Interviewee: It does. I think it is. It’s a complete thing. Because otherwise you just forget about it and just go about and, before you know it, you fall back into your usual routine.

Facilitator: What about the website? If we took away that, would it make a difference to your motivation?

Interviewee: Oh well, for myself, I would say no. Like I said, I’m not always on it.

Facilitator: Since you started the program, have you made any specific changes to your lifestyle? Anything that you’ve been focussing on?

Interviewee: Yes.

Facilitator: Yes? What’s been a priority for you?

Interviewee: I’ll just – just in regards to drinking. So yeah, just not having it be something you do all the time. But allowing myself to have a glass of wine on the weekend, because that was something – because moving to a small town and not knowing a lot of people, I started getting into a bad habit of having a glass of wine every day. So for me now, that’s something that I’ve definitely changed. When I went to the conference thing that we had at the school, that was one of the things they talked about.

Facilitator: About alcohol?

Interviewee: Yeah. But not that I’m an alcoholic. But it was just a habit of just “oh yeah, just unwind” you know? Before you know it, it’s definitely something that could – what is it? Put on the calories and stuff? So yeah, that’s been a conscious thing to not do that. Not falling into that sort of habit. Because obviously when you have a glass of wine, then you want to start nibbling on snacks and stuff. So yeah.

Facilitator: Have you used any of the – so in the session where you talked about behaviour change strategies like problem solving or goal setting and action plans, have you used any of those strategies to help you cut down the drinking?

Interviewee: Yes, well obviously not having any wine or anything in the house. Is always a strong thing. If you don’t have it, you’re not going to touch it. That’s what I’ve – it’s helped me to stay on track in that area. Yeah.

Facilitator: Great. So would you say that you’ve used the action plan? Or more problem solving?

Interviewee: I’d say more problem solving.

Facilitator: It’s interesting how what you’ve just said – and I think that would be important to other women – any other strategies about how you’ve reduced that – it’s just that we’re trying to help with what you say and use your ideas to help other women. Any other strategies you’ve used to help cut down alcohol?

Interviewee: Yeah, well obviously just purchasing it at the end of the week if I felt like that’s what I wanted to do for the weekend, have a glass of wine. But also having a focus on something else. Being a bit more creative and taking up a activity …

Facilitator: Have you done that?

Interviewee: … that you’re not conscious – yeah and I do. Because I do a bit of needlework and stuff. But if you can have a hobby, you’re not bored. Because I think you tend to be – it’s boredom that makes you set your ways in habits and stuff.

Facilitator: Yep, I agree.

Interviewee: And just being very conscious of it. Knowing that it’s something I wanted to change, I guess.

Facilitator: You’ve been able to sustain that change for the last couple of months?

Interviewee: Yes, I have actually.

Facilitator: Also with the program, we talked about accessing health services, you know, getting health checks. Have you done anything else different since joining the program? Seen a doctor or accessed any other services that perhaps you wouldn’t have otherwise?

Interviewee: No. Like I take myself to – like have a health check. It’s something that sort of prompt me.

Facilitator: Good. So it prompt you to do a health check?

Interviewee: Yeah. That was all good.

Facilitator: Great. So you’re healthy. That’s good. Anything else? Have you been weighing yourself since you started the program or been more conscious of that?

Interviewee: Well I do. Then sometimes then I stop because I can usually go by what my clothing is as to how that works.

Facilitator: Some people find that weighing can be a bit – cause them to be a bit negative.

Interviewee: Down on themselves. Yeah. Well that’s it. I can sometimes feel that way. Because you feel like you’ve been doing all the good things and nothing seems to be happening.

Facilitator: So it’s – sometimes you find that it …?

Interviewee: I do have a set of scales, but I have to say it’s something I don’t jump on all the time. I kind of just know in myself, oh I need to do something.

Facilitator: Great. So you’re conscious of that.

Interviewee: Yeah, that’s it.

Facilitator: Another thing that I’m particularly interested in is we’re trying to work out about the program about whether the messages in the program or what we talked about – so you said you’ve cut back on drinking – whether that’s affected anybody else in the town or whether it’s affected your family. So since participating in the program, have you talked about your health with your family or has that changed?

Interviewee: Well my husband does a lot of fly in-fly out so he works interstate. So it’s virtually my daughter and myself. But no, not really. I haven’t really discussed with anyone.

Facilitator: Have you talked about drinking less with anybody?

Interviewee: No, not really. Because, like I said, most of the girls that would’ve went to that group – they’re obviously girls that I know at school, but it’s not something that I’d discuss with them. Everyone’s sort of gone off and done their own sort of thing.

Facilitator: So as a town, the group, you haven’t really discussed health with much of the group.

Interviewee: No. Because I’d say after that, having that time together at the school, most of those ladies I wouldn’t see.

Facilitator: Do you talk about your health with any of your other friends?

Interviewee: Oh yeah. I have my friends – we do, always talking and they’re always sharing. They’re trying to lose weight and things like that.

Facilitator: What about – since you started the program, have you made any changes to what you eat and what you buy for you and your daughter?

Interviewee: Yeah, I do. I always just try to have a lot of fruit and vegetables in the house.

Facilitator: Has that been since the program or always?

Interviewee: No, I’d say that’s always. Unless take away food, I guess. Been more conscious of being – preparing our food rather than buying stuff.

Facilitator: So you’ve been more conscious of that?

Interviewee: Yes.

Facilitator: So that’s a good example of how you’ve changed your behaviour. That has influenced others. So that’s influencing your daughter. Sorry.

Interviewee: No, you’re right.

Facilitator: The last question I have for you is if we were going to come back and run this program again in Wonthaggi, based on your experience, would you recommend the program to anybody else in the town?

Interviewee: Oh yeah, because like I said, with the emails and that, it just keeps sort of a – it’s almost like an alarm to just keep us conscious of our lifestyle and what your goals are. Just keeping yourself living healthy. So yeah, I would.

Facilitator: The girl that came to run the program – would you prefer if it was someone local who ran the program or someone from Melbourne?

Interviewee: I guess if it was someone local, it would be more handy because obviously, that person would be about more. But other than that, no, I wouldn’t see any other reason why it would have to be someone different.

Facilitator: So otherwise no difference?

Interviewee: No. Unless they’re going to get – have more one-on-one, like more face time. More frequent. But if that wasn’t the case, well then no, there wouldn’t be.

Facilitator: Would you prefer – if you were going to receive health information, would you prefer it to be one-on-one or a group based session?

Interviewee: Either way. I would be happy with either. I’m happy for groups or one-on-one’s always more personal. But group are always fun too because you get to encourage each other.

Facilitator: Obviously you just get that support from each other in the group. If it helps make any sort of social – some people have sort of kept in touch through the program, through walking groups and things like that. So that’s been helpful for them. What about the venue? Is that convenient or was that a good venue for you?

Interviewee: Yeah, it was. Because at school, it’s always the most handy thing for most women.

Facilitator: If we were going to change this program in the future, how could we change it to make you more motivated or to help you further?

Interviewee: I think how you mentioned if somebody was to come down. I think if there was more – not such a long period. If somebody was to come down and just sort of meet with the group again, that might be more encouraging.

Facilitator: So that would help?

Interviewee: Like sometimes if I – once you’ve been to something then that’s – you’re not going to see those people or whatever again, you can kind of lose your motivation in that way. But yeah, I’d just say maybe more …

Facilitator: More sessions?

Interviewee: Yeah.

Facilitator: Anything else you’d like? More phone calls, less phone calls? Anything else you can comment on how we could improve it?

Interviewee: No, I’d say those were quite fine. The emails and all that sort of prompting and stuff.

Facilitator: Anything else you want to add? Anything else you think I need to know about your town?

Interviewee: No, only that it is just a very small town and – I’ve only been living here for a short time, so just now I'm getting to know people really. But other than that, it’s just, yeah.

Facilitator: Is it hard …?

Interviewee: Sorry?

Facilitator: Is it hard to meet people? Are there many options for socialising?

Interviewee: I wouldn’t say there’s many options for socialising. But because I do have a daughter at school, it sort of makes it easier to meet people. Obviously, then you can join a church and stuff like that.

Facilitator: So the church is a good option as well. What about sporting clubs for adults? Is that an option?

Interviewee: What was that?

Facilitator: Sporting clubs? Are there any adult sporting clubs?

Interviewee: Oh there’s like a golf course and things like that.

Facilitator: Netball or tennis or anything like that?

Interviewee: Oh yeah. Yeah there’s all those sorts of things. A lot of people down this way do surfing and all that sort of thing. Bike riding.

Facilitator: Would you consider it quieter – a town that’s quite social? Do you often see lots of women or people getting together to socialise? Or it’s a little bit less?

Interviewee: Oh no. Yeah, I do. Because I’ve obviously, when you rang earlier, I was having coffee with a friend. But yeah.

Facilitator: Plenty of opportunity…?

Interviewee: There’s always people out and about.

Facilitator: Okay, perfect. I don’t think I’ve got anything else to ask you. I think that’s about it, unless you’ve got anything else you want us to add about the evaluation of the program. Anything else that you think would be useful?

Interviewee: No. I’ve got nothing else to add.

Facilitator: Nothing else to add? Perfect. Well thank you so much again for your time. It’s much appreciated. Everything you say is very valuable to us. As a token of appreciation, we’ll send you a gift card in the mail this week. We hope that you’ve enjoyed the program this far and we look forward to seeing you in a couple of months at our 12 month visit.

Interviewee: No problem. Thank you so much.

Facilitator: Thank you so much for your time.

Interviewee: Thank you.

Facilitator: Thanks, bye now.

end of transcript

Participant #FS

full transcript

Facilitator: Perfect. No, because this is just about – hold on one moment, can you hear me?

Interviewee: Yeah.

Facilitator: This is just about the evaluation of the program, so it’s really asking about your opinion on how you thought the program went, what you liked about it and just about whether the program met your expectations. So it’s more about getting some insight from you, so it’s not a test or anything, you don’t need your book.

Interviewee: Yeah, no…

Facilitator: So thank you so much. So to start off with, I just want to do – the whole point of the evaluation is to look at what motivated you to join the program, what behaviour changes you’ve made and about how satisfied you were with the program. But to start off, I was hoping if you could just tell me a little bit about your community in Wonthaggi and just describe it in terms of size, the common health problems, you know, demographics… Anything you can think of that sort of you can identify with your community.

Interviewee: God.

Facilitator: What do you think, is it a big community, small community..?

Interviewee: It’s about 7,000 people. So you’d describe that as being little.

Facilitator: Yeah no problem, any common health problems? Do you think anyone often complains about weight or diabetes? Any common problems?

Interviewee: Not that I know of, I wouldn’t have a clue. No.

Facilitator: What about the engagement of social connections in the community? Do you find that people are isolated or well connected through different community groups or can you comment on anything like that?

Interviewee: I would think that there would be different community groups, that everyone can be socially connected, yeah.

Facilitator: Okay, how long have you been living in the community for?

Interviewee: Since ’86.

Facilitator: Great, no problem.

Interviewee: Yeah, nearly 20 years.

Facilitator: And what are the community groups? So you said there’s lots of community groups, are there any ones that stand out to you?

Interviewee: Are there any community groups that stand out for me?

Facilitator: Yeah.

Interviewee: Well, me having school aged kids, I just sort of revolve around the school and the kinder circle and yeah, soccer club and basketball club and all those things, yeah.

Facilitator: Anything else?

Interviewee: Well there’s the pool that needs a lot of loving, but there’s the pool. No, nothing particular.

Facilitator: But overall you’d say that people are quite connected with each other?

Interviewee: I would think so, yeah.

Facilitator: Can I ask you, I know it’s a while ago now, but if you think back what motivated you to join the program, was there anything that – what made you decide to join? Were you interested in the health or…?

Interviewee: I need to lose a lot of weight.

Facilitator: Is that important to you?

Interviewee: Yeah but I don’t seem to be doing too much about it, but yeah.

Facilitator: Yeah, and did you come to the session, how did you hear about it, did you come with friends or did you come alone?

Interviewee: I came through the school newsletter, is where I heard about it and yeah, come to it up to the school.

Facilitator: Yeah, did you go with any friends or just by yourself?

Interviewee: No, just by myself, I knew everyone in the room though.

Facilitator: Was there a lot of women from the school?

Interviewee: There wasn’t a huge group of us, I’d say half a dozen women. But yeah, we just all went off our own back. Well, I did anyway.

Facilitator: Okay, so as you mentioned, there weren’t a whole lot of women in that room. Can you think of any reasons as to why perhaps the program wasn’t attended by more members in your community. It was in October last year, any things that you think were going on that you think would have prevented other people for going or that would have made it hard for yourself to go?

Interviewee: I think people are always self-conscious when they’re going, if they want to lose weight. So I suppose it just sort of comes back to that, just you know, do you want to admit that you want to lose weight in front of a whole heap of other mums at school.

Facilitator: Yeah perfect.

Interviewee: I don’t know, that’s my opinion as to why I didn’t want to go or I don’t want Eliza to know that I’m going to lose weight, just self-consciousness I suppose.

Facilitator: Yeah, was that a problem for you?

Interviewee: No, I didn’t really give a shit. I’m not one to worry about what other people think.

Facilitator: Yeah good. Is there anything else you can think of, so the timing of the year or the venue? Anything else you can think of that maybe would have stopped people?

Interviewee: Perhaps, not actually hold it within the school community, although that’s a public building and everything else, Perhaps maybe hold it – it’s a bit hard to do it at your park or somewhere else…

Facilitator: So you think maybe somewhere a bit more mutual.

Interviewee: Yeah, where as you might get other people from other schools and that go, could increase your numbers maybe.

Facilitator: Okay, what about – do you think that people were aware of the program? Do you think there was enough advertising about the program?

Interviewee: Well I only got to hear about through the newsletter in the school, I didn’t see it, I don’t know whether you advertised in a local paper or not or anything else, but yeah, that was where I heard about it from.

Facilitator: Do you read the local paper?

Interviewee: I do read it but not everything.

Facilitator: Of course, yeah.

Interviewee: But yeah, that might have been the way to go, I don’t know. I don’t know if you did or you didn’t, so yeah?

Facilitator: No problem. Anything else you can think of about why other people may not have attended?

Interviewee: I don’t know, did you advertise it other school’s newsletters?

Facilitator: Yeah we did.

Interviewee: Yeah, maybe just because it was held at one school and each school, I don’t know.

Facilitator: No, no, I know, a bit hard, sorry.

Interviewee: Too hard.

Facilitator: And I just want to look at what changes you’ve actually made to your behaviour. I know you talked about this with the girls that rang you a couple of weeks ago for the phone coaching, but is there anything – so you attended the group session in Wonthaggi and you’ve got your manual. Was there any key messages that you learnt from the program? Anything that stands out to you that you remember specifically about what the session was about or..?

Interviewee: Well I was a little disappointed in perhaps maybe, when the lady rang me, that was the first phone call that I’ve had, and when she rang me that motivated me again. So I thought maybe perhaps a little more – that would work for me, a little more phone calls, you know, phone action. Rather than just a text message, because you get a text message and you go, ‘I can’t be bothered dealing with that now.’

Facilitator: Okay, but so going back, is there anything that, at the session that you’ve learnt. So were any of those six key messages, was there anything new that you learnt though at the session that you thought was helpful?

Interviewee: No, I already knew all those things. Not that they weren’t helpful. It was a nice fresh reminder, but yeah, I already knew all the sort of rules and regulations. It’s a matter of activating them that’s all.

Facilitator: And what about – sorry, when you said that you knew most of it and that you liked being refreshed, was there anything in particular that you liked the refresher on?

Interviewee: I can’t even remember them to tell you what they were? [laughs]

Facilitator: So there were the six messages about soft drink, about eating breakfast, about more fruit… Was there anything again that helped spark up your memory a little bit?

Interviewee: Well they all do, because it’s all just a refresher. It’s meant to be told again…

Facilitator: And that helps you?

Interviewee: Yeah. But yeah, you need that reminder everyday don’t you?

Facilitator: Yeah.

Interviewee: And I do.

Facilitator: Of course.

Interviewee: One of the other girls that did the course, she’s lost a stack of weight.

Facilitator: Really?

Interviewee: Really, yeah.

Facilitator: How did she do that, was it through the program or she..?

Interviewee: She stopped eating junk food and she’s walking everyday, and yeah she looks fantastic. So yeah, you look at her and you go, ‘Good on ya, well done!’

Facilitator: That is fantastic. What do you think would help you lose all the weight like her?

Interviewee: What do I think would help me? Probably another 30 hours in each day, and a pill that gives you motivation.

Facilitator: So would you say, is motivation a problem for you?

Interviewee: It is for me, yeah.

Facilitator: But since being in the program, being in the program and getting the text messages and you know, going to that first session, has it made you start to think a little bit more about what you’re eating or about your weight?

Interviewee: Yeah, no definitely, it has.

Facilitator: I know, a bit of a general question, but how often are you thinking about your weight or what you’re eating? Like, most days, every week or any..?

Interviewee: Think about what I should be doing? Yeah, thinking about it and actually activating it is two different things so which question are you asking me?

Facilitator: Sorry, just to get you – you’re thinking about it more since you joined the program, just to confirm?

Interviewee: Yeah.

Facilitator: Perfect.

Interviewee: No, I’m more conscious of what I’m doing and what I’m shoving in my mouth, so yeah, you can probably say most days to that.

Facilitator: Yeah perfect. Now moving on, so you mentioned before about the different components that you found useful. So you said that you found the phone coaching useful. So the components of the program that you’ve been involved with, you had the group session, you’ve had the manual, the SMS messages and the phone coaching. What I’m interested in is to find out what did you find the most valuable? So if we were going to run this program again, we just want to know what you liked best and what you didn’t find so helpful, what was most valuable?

Interviewee: Text messages, you sort of just ignore those.

Facilitator: So did it motivate you at all or not really?

Interviewee: No, I’d say no to that, the phone call made me go, rightio it’s time to get my shit together.

Facilitator: So the phone call was motivating.

Interviewee: Yeah the phone call was best for me.

Facilitator: Great. What about the group session, what did you think?

Interviewee: Yeah no it was good, it was well read, yeah. But yeah, you only get the one of them don’t you?

Facilitator: Yeah only one, and we’re coming back at the year time.

Interviewee: One doesn’t motivate me.

Facilitator: So you need more?

Interviewee: Yeah.

Facilitator: And one of them, when you say it doesn’t motivate you, any reason or just you find that you need?

Interviewee: Just because I need more reminders and more – yeah certainly more of a kick up the arse really.

Facilitator: Okay, what about the website? Did you access the website at all or have you been on the website?

Interviewee: No I think I did look up the site and then that was it, but no I haven’t gotten back onto it or gone into much at all.

Facilitator: Do you use the internet much?

Interviewee: Yeah I do yeah.

Facilitator: What would you like on the website to make it more useful?

Interviewee: I don’t know.

Facilitator: Not sure?

Interviewee: You know, since I just went on it and looked at it. I probably only did it the first week or something like that and then just, not doing it since then. Slackness really on my behalf, nothing wrong with how you guys are running it.

Facilitator: Thank you. So just to clarify something, if we were going to take out one of the components of the Help Her Program, if we didn’t have the text messages and we kept in the phone coaching and the initial group session, do you think you’d be just as motivated or do you think that would make any changes to your weight if we cut out the text messages, or you do think it’s helpful?

Interviewee: I don’t think that would affect it either way, no. I think it’s a good idea to text, but I just found that I didn’t take enough notice of them. It was a reminder but, yeah I just didn’t listen to them. That’s my fault, not your fault.

Facilitator: Of course, so it was a reminder, but not motivating enough?

Interviewee: Yeah, I found that when I had the phone call that was motivation again, I went right okay, refocus and get on with this.

Facilitator: Perfect, what about the manual? Be honest, it’s completely open, we won’t be offended.

Interviewee: That was good to start off with, but yeah I could sort of push that by the wayside.

Facilitator: If we didn’t have the manual do you think that would have influenced where your motivation is and about the end of the program, do you think that would have made a difference if we didn’t have that manual?

Interviewee: What, would it make it better?

Facilitator: Yeah, do you think the manual helps?

Interviewee: Yeah I think it does, yeah.

Facilitator: So if we were going to – so for you at the end of the year, if we took away the SMSs it probably wouldn’t change your weight, but if we took away the manual perhaps it would have some…?

Interviewee: If you took away your phone contact it definitely would have.

Facilitator: So the phone contact was more important?

Interviewee: I’m just more inclined to say, yeah you need that bit of extra phone contact. But some people would think the completely different – but that’s my opinion.

Facilitator: Yeah, but the whole point is, we want to get your feedback and then we’ll look at a whole lot of different people and what they’ve said. I’m sure you’re not going to be the only one – everyone likes things different but we’re just trying to get an idea of everyone’s opinion, collate it all and then work it out.

Interviewee: Get more ideas.

Facilitator: Yeah. So just moving on, so have you actually made any changes to your lifestyle since you’ve started the program, so anything you’ve changed to your diet and exercise?

Interviewee: Yeah, I’m out walking more, and yeah watching what I eat a little bit sometimes. Yeah I’m more conscious.

Facilitator: Are you weighing yourself?

Interviewee: Not very often.

Facilitator: And when you say you’re out more, how much more do you think you’ve increased by, like one walk a week, two walks a week or..?

Interviewee: Two walks, yeah.

Facilitator: And what about your eating, has that changed much?

Interviewee: Yeah less junk and more fruit.

Facilitator: And when you say junk, what…?

Interviewee: Chips and lollies and biscuits and all those bad horrible things.

Facilitator: Great, okay, and when you’ve made these changes, so you sad you’re walking more and eating more fruit and less junk food, do you remember using any of those behaviour strategies that we talked about in the manual and the sessions? So the goal setting, the problem solving, relapse prevention, the action plans… Did you use any of that or anything that comes to mind?

Interviewee: Nothing really comes to mind. I did earlier, I’ve just gotten slack with it all.

Facilitator: And when you said a bit earlier, what were you using, goal setting or problem solving or anything that stands out?

Interviewee: Just the problem solving idea and changing the alternative and instead of having that, have that…

Facilitator: Yeah, so any chance you can give me an example of how you used the problem solving please?

Interviewee: Yeah just go onto the alternate food rather than..?

Facilitator: So, for food?

Interviewee: Yeah.

Facilitator: So was that for getting rid of the junk food like you mentioned?

Interviewee: Yeah.

Facilitator: So deciding, getting rid of junk food and looking at alternatives?

Interviewee: Yeah, just get more fruit and having an apple instead of grabbing a biscuit.

Facilitator: And that was helpful?

Interviewee: Yeah.

Facilitator: Perfect. Just moving towards the end now sorry, the other thing I’m interested in looking at is about the reach of the program, so whether anything you learnt influenced anyone else in your life, so any of your family members or if you have a partner? So did anything you do change what you purchase for the house or anything like that? Any influence on anybody else?

Interviewee: Yeah it did, it improved it for us yeah. The whole family really.

Facilitator: And sorry, just to document that, what did you change to what you bought for the family?

Interviewee: I just less junk food.

Facilitator: In the house?

Interviewee: Yeah.

Facilitator: Anything else?

Interviewee: More fruit, less junk food.

Facilitator: Great, what about exercise?

Interviewee: Yeah the kids will come with me occasionally for a walk and that.

Facilitator: Great, and did you talk about anything you learnt with the kids or just changed what was in the house?

Interviewee: No I just changed it, yeah.

Facilitator: Did you talk about the messages with anybody else or anyone else outside or any friends, or anyone else in the program?

Interviewee: Not really, maybe a little bit, but not in great detail at all.

Facilitator: Do you talk about your health normally with people?

Interviewee: No.

Facilitator: Is that probably why you didn’t talk about it?

Interviewee: Yeah probably. That’s just me, I’m a private person.

Facilitator: That’s fair enough. And sorry, the kids, were they supportive or happy with the changes you made?

Interviewee: Yeah except the teenagers yeah.

Facilitator: Perfect, okay. And the last question that I have to ask you is, so we’re looking at satisfaction of the program with the participants, but based on your experiences, would you recommend the program to anyone else in the community?

Interviewee: Yeah, because I go on the grounds that it’s not going to hurt you and yeah, it may not have necessarily jump started me towards a 10 kilo loss or anything, but it does work, it can work.

Facilitator: Great. So the facilitator that came to talk to you, so when she ran the group session, what would you prefer, did you prefer someone from outside, or would you have preferred if it was someone from your community?

Interviewee: It doesn’t matter to me.

Facilitator: Doesn’t matter. Because some people have preferred it to be someone they know, someone like someone external…

Interviewee: No, either-either, not fussed.

Facilitator: What about the venue, was that okay?

Interviewee: Yeah it was fine for me, you know, we were within our school community, but I just thought that maybe perhaps others, they might feel better in – not that there’s anything wrong, don’t get me wrong. I’m just thinking for others it might have been more comfortable had it had been in a…

Facilitator: Like a community centre or something else?

Interviewee: Yeah, that’s the way, would be a way to go.

Facilitator: And what about a group session? So do you prefer, if you were going to get this health advice, would you prefer it in a group session or would you prefer it on an individual session?

Interviewee: That’s a bit of a – yeah individual would probably be good, but either-either really. If you’re going to activate you’re going to activate aren’t you?

Facilitator: Yeah, but some people just feel more comfortable talking, don’t feel comfortable talking about health issues in groups, so that’s awesome.

Interviewee: Yeah, I don’t know. I went, so I did well, I went.

Facilitator: Great, and we appreciate that and we thank you for that.

Interviewee: Yeah it was a bit of a hurdle, but I went.

Facilitator: When you say a bit of a hurdle, as in just to come out, what do you mean by that, sorry?

Interviewee: It is, it’s going publicly and going, I need this help to lose weight really. But yeah, like I said I’m a bit of a private person so it’s a bit of a hurdle, but yeah, you’ve got to try and get over it?

Facilitator: Have you done anything like this before?

Interviewee: No.

Facilitator: Has there been anything available like this before?

Interviewee: Probably.

Facilitator: But not that you went to?

Interviewee: Yeah I don’t know, probably, I don’t know.

Facilitator: Perfect, and the absolute last question I swear, if we were going to run this program again, you already mentioned to me that you’d like more phone contact, but is there anything else, is there any other way that you’d like the program to be run? So would you like more sessions or how would you like to see this run differently in the future?

Interviewee: Yeah I’d probably just stick with more phone calls. I don’t think there’s anything else that I’d particularly change about it. A bit more phone contact, because to me that gives me a foot up the arse and gets me going.

Facilitator: Any idea how many you’d want in a year roughly?

Interviewee: Say, four phone calls or something like that.

Facilitator: Great.

Interviewee: Just a foot up the arse.

Facilitator: And nothing else?

Interviewee: No I think it’s done well.

Facilitator: Perfect. So that’s all I wanted to ask you, unless you’ve got anything else you want to add in that you think is important? The point of the evaluations, just to get your feedback and see what you thought about the program and how we could improve it and just to get your insight into the program. Anything else that you think is important for me to know?

Interviewee: Yeah just one thing, you need to find the motivation pill for us.

Facilitator: Yes, I’d be a millionaire.

Interviewee: That’s right, you would be. That’s about the only thing, but no, it’s all good.

Facilitator: Thank you so much for your time, it’s really appreciated and everything you say is really valuable to us because we’re hoping only to improve this program in the future and I’m glad that you’ve enjoyed it so far and I’ll definitely get the feedback back to the head of the program about more phone calls. But thank you again so much, and if you have any questions or concerns just let us know. I’ll send you that gift certificate in the mail, so that should come next week, but if it doesn’t come just give me a call back, but I don’t see – I’ve got your address written down as 92 [White Road] Wonthaggi?

Interviewee: Yeah that’s correct.

Facilitator: So I’ll send that to you in the mail today.

Interviewee: Alright no worries.

Facilitator: Thank you so much again for your time.

Interviewee: Bye.

Facilitator: Bye.

end of transcript

Participant # WT

full transcript

Facilitator: It's really just an informal chat just to get your views about your progress in the program, whether you liked it and how you think we could change the program to make it better.

Interviewee: Yes.

Facilitator: Then as a token of appreciation we will send you a $20 gift card as well to Coles Myer.

Interviewee: Yes.

Facilitator: I also just wanted to check with you is it okay if I tape record the interview? It's just to make sure that we correctly understand everything you say and to make sure we...

Interviewee: Yes.

Facilitator: ...can use everything you say. Great. Everything will be kept completely confidential. Don’t worry, no one else will have access to what you say. So to start off with if you can we're just trying to compare each of the different 42 towns that we went to. I was just hoping that you could tell me a little bit about the opportunities to exercise and to buy fresh fruit and vegetables and fresh foods in Wonthaggi.

Interviewee: Yes, I guess it's pretty readily available. Mind you it's pretty much only from the supermarkets and things like that. Once or twice a month we have a market on which has home grown sort of vegetables and stuff like that. But other than that I mean I think the nearest organic shop is Inverloch which is 10 minutes away.

Facilitator: Yes. What about is there a gym or good walking tracks, sporting clubs to exercise?

Interviewee: Yes, definitely. There is. There's a YMCA gym, north Wonthaggi and there's also now a 24 hour Voyage which I'm a member of. There's a good six or seven kilometre track that a lot of people use for exercise as well.

Facilitator: That's great. Do you think as a whole that women in Wonthaggi - it's an active community?

Interviewee: Yes and no. I mean there's a lot of poorer people, single mothers and things like that. I'd say it's half and half really but that's just my opinion.

Facilitator: No, of course. That's what we're just trying to get, your opinion. Would you consider the community or yourself or any of your friends to be reasonably health conscious?

Interviewee: I would say so. But then again it's half and half as well because there's put here and a lot of people hang out there of a weekend and things like that.

Facilitator: Yes. In terms of a social community or one that there's many opportunities to socialise, to see friends, to meet people, what are your thoughts on that about Wonthaggi?

Interviewee: I don’t think there's a lot down here for the youth, like young women. I mean there are community groups [audio cuts out 0:02:46.0] and things like that but not as much as a bigger town I suppose.

Facilitator: Yes. What social activities are available? So if you were going to meet friends or catch up with people what would you...

Interviewee: A lot of the time we just go out for coffee or a meal or something like that. There's not really a lot of places to go. But my friends and I all have children, we tend to go to the park and have barbecues and salads and stuff like that and let the kids play and things like that.

Facilitator: Yes. So if you were...

Interviewee: Playgroup sort of stuff as well.

Facilitator: Yes. So would you say that if you wanted to be social there'd be enough opportunities to be social?

Interviewee: Yes. Yes I would say so. If you want to get out there and do it, yes.

Facilitator: You can do it. Now thinking back to when you started this program in about October last year do you remember what motivated you to join this healthy lifestyle program?

Interviewee: Well I'm a qualified fitness instructor so I saw it in the newsletter and I was interested on what it would be like and things like that.

Facilitator: Yes. So did you come to receive health information, is that correct?

Interviewee: Not really because I found a lot of the information I already knew but I also noticed that when I was there that not a lot of women did know all that.

Facilitator: Yes. So from you it was just more...

Interviewee: Just interested in what the program was going to be like and what other women thought and things like that.

Facilitator: Yes. When you said before that you knew a lot of the information did you find it still useful as a refresher or was it not overly helpful to you?

Interviewee: It wasn't but I don't think you had any other people that were actually already in the fitness industry there. But yes, there were a few things that were a refresher as well.

Facilitator: Was there anything that stood out to you that you learnt or that you found particularly useful that you can remember from either the session or from the manual or from anything that we've given you?

Interviewee: Not really that I can remember, no specifics. But as I said I knew most of it, well pretty much all of it, but it was just nice to get a refresher and bring those things to my mind again.

Facilitator: Yes. So if you were to describe the program to somebody else, so a friend or anyone else in the town, if you were to finish my sentence when I said the program was about what do you think you'd say?

Interviewee: Making healthier lifestyle choices and putting a lot more importance on getting moving and exercise.

Facilitator: Great. So from your point of view you thought it was about both exercise and diet or was it more about one or the other?

Interviewee: I think it was both and it really motivated me because I've lost quite a lot of weight as well, that sort of stuff helps.

Facilitator: That's great. You just mentioned that you've lost quite a bit of weight, has that been since starting the program or...

Interviewee: Yes, pretty much because it sort of just spurred me on a little bit. I was starting to lose a little bit of motivation at the gym and it just started me back up again.

Facilitator: Yes. As you're obviously involved in the program you'd know that the program is made up of that initial session in Wonthaggi then we've been having the text messages, the phone coaching and the website as well as the manual. Did anything in particular motivate you more than - sorry, has any part of that been more useful than the others, do you get what I mean?

Interviewee: I think with the manual reading through it a couple of times and then tracking my weight once a month. That part was really good, having that so I could track my progress.

Facilitator: That resource, yes. So you've been weighing yourself?

Interviewee: Yes.

Facilitator: Were you doing that before you started the program?

Interviewee: I was but sporadically.

Facilitator: Yes. So now a big more regular.

Interviewee: Yes. It's been once a month pretty much.

Facilitator: Great. Have you found the weighing yourself helpful?

Interviewee: I have but I also know that there's a real difference between my full fat water content so I don’t put all my eggs in the basket of weighing myself because I know that - because I do measurements as well - if you lose centimetres and not weight you've still lost.

Facilitator: Yes, of course. In terms of since you started the program do you think you've thought more about your exercise, your weight or your diet more since?

Interviewee: It was more brought to my attention, definitely.

Facilitator: Yes. Did you think more about weight or have you been thinking more about exercise, more about your food, anything more so than others?

Interviewee: A bit of both because with increasing protein and keeping hydrated and things like that really do help develop muscle, help muscle and drop unhealthy weight.

Facilitator: So just to clarify, you've been thinking about all of them equally.

Interviewee: Yes because they all work hand-in-hand.

Facilitator: Yes, of course. Sorry, just to go back to the components of the program, so all those messages, did you find the text messages or the website or the phone coaching helpful?

Interviewee: I think the text messages are a good idea because they keep it fresh in your mind.

Facilitator: I know it gets a bit confusing but some women talked about it being a reminder and others were talking about it being more of a motivator. What was it...

Interviewee: I think it's a bit of both. I mean sometimes having children and things like that you can get a little bit busy and you take care of them instead of yourself. But with the text messages reminding it was sort of like it just brings it back into your head. So I think it's just a reminder to stay motivated really.

Facilitator: Yes, good. If we were to take out any of those parts of the program which part do you think we could take out that would lead you to be as motivated as you are now? So if we took out that phone coaching or if we took out that website.

Interviewee: I didn't - are you there?

Facilitator: Yes, sorry.

Interviewee: I didn’t go to the website just because of how busy I am. Yes, I wouldn't take any of it out because different people respond to different things.

Facilitator: Good, it's all valuable. So you mentioned to me a minute ago that you said that the information you learnt wasn't new to you but was a refresher about the healthy lifestyle information. What about the information we talked about about the behaviour change techniques like that goal setting, problem solving, action plans, those strategies to help make change. Was that new to you?

Interviewee: No because, as I said, I'm a fitness instructor so I teach that to people every day.

Facilitator: You're teaching that already.

Interviewee: When I was working, yes. But as I said, it just sort of made me think about my own goals.

Facilitator: Yes. Since starting the program is there something in particular that you have - you mentioned that you've lost quite a bit of weight but what have you actually changed specifically do you think?

Interviewee: Just basically eating regularly and smaller portions and just really looking at what I eat in comparison to how much I move because eat less and move more basically. Or sorry, make the right food choices.

Facilitator: Yes, good. When you say make the right food choices was that some of the information you used from the session?

Interviewee: Well as I said...

Facilitator: You know it.

Interviewee: ...I knew it but it did bring it back and I was more aware of what I was putting in as expending.

Facilitator: Any other specific changes like more fruit and vegetables? You mentioned more water, less junk food. Anything...

Interviewee: Yes, definitely more water.

Facilitator: ...else like that?

Interviewee: More water was a big thing for me because being busy and stuff it does - when it's cold you don’t drink as much water and you've got to be more conscious of that.

Facilitator: Yes, of course, I agree. Sorry you said you've managed to - it sounds like you've made some great progress. Did you use those behaviour strategies, anything that stands out to you like goal setting?

Interviewee: Definitely the goal setting because setting smaller goals to get to the ultimate one in the end.

Facilitator: Yes. Has that been helpful?

Interviewee: Yes, definitely because I tend to put a lot of pressure on myself and splitting it up into smaller goals helps to get there without putting too much mental stress on yourself.

Facilitator: Yes. Has self-monitoring also helped you make the changes?

Interviewee: Yes, definitely. The graph in the back of the manual...

Facilitator: Has been helpful?

Interviewee: ...was a really good way of seeing the changes.

Facilitator: So you said you've self-monitored your weight, what about your food and your exercise? Do you use any self-monitoring of them?

Interviewee: I used to use a food diary just when I was in the first phase of losing the weight. Now I'm sort of in just keeping myself at the weight I am now. But yes, I definitely used a food diary when I was trying to lose the weight, now I'm just trying to maintain it.

Facilitator: Now you're trying to maintain it. So break that down into goals.

Interviewee: I pretty much eat the same sort of things but I don’t tend to need to write it down because I know how many calories are in this and that and things like that.

Facilitator: So just to clarify, what we're really interested in is, as you've said, you're a fitness coach, you already knew what to do. We're just trying to pinpoint what it is that's made you make these changes since you started the program. So what I'm hearing from you is the things that have helped you is that constant reminder, that motivation through the text messages.

Interviewee: Absolutely, yes and the manual.

Facilitator: You said the self-monitoring's been helpful.

Interviewee: Yes, definitely.

Facilitator: Has anything else specifically since you started this program helped you better improve your motivation and helped you achieve your goals, anything else that I'm missing?

Interviewee: No, I don't think so. I think I've covered it, yes.

Facilitator: Yes. What about relapse prevention, important to you?

Interviewee: Yes well I mean I can't say I haven't had any junk food. Of course the level has decreased. But with the relapse prevention it's just with the motivational things that you have provided it's kept it to a minimum.

Facilitator: That's fantastic, great to hear. Do you value or have you found you've had much support from your family or friends?

Interviewee: Not really, no. It's been more of a personal thing for me.

Facilitator: Yes and that's great, that's what a lot of women have said because we found some woman say that they really need that support from a partner or children but some women say that they like to do it more...

Interviewee: I've been for walks with friends and stuff and things like that and just getting out and taking the kids out and things like that. But that would probably be it I'd say.

Facilitator: Yes. Just to confirm, has this change been pretty consistent since starting that program in about October last year or has it gone in waves?

Interviewee: Absolutely. No, no, it's been consistent. So far.

Facilitator: Has it had any effect on your family? You said that you've been exercising more, drinking more water, more fresh foods. Any effect on your family?

Interviewee: I don’t know. Like taking the boys out for a bike ride and things like that and going across the road and playing basketball and things like that.

Facilitator: Is that quite new or is that something you were always doing?

Interviewee: I was always doing that. They're pretty high energy kids and need to be tired out.

Facilitator: Yes. So another thing that we're looking at is about the reach of the program, so trying to work out, as a result of you participating in this program, whether it's influenced only the participants or whether it's affected their families. Would you say since...

Interviewee: I'd probably say the diet has changed. The children are more likely to try new things.

Facilitator: Yes. What kind of things are you trying?

Interviewee: Well you know what children are like, they're not great on vegetables and things like that but you keep serving it up and eventually they'll try it.

Facilitator: Yes, good. So you've changed some of the foods that you've been cooking for your family.

Interviewee: Not overly but yes it has impacted it.

Facilitator: A little bit, yes. Any other examples in terms of food other changes you've made to the household? If you haven't that's no problem. But any other examples you can think of that come to mind of how you've influenced your children?

Interviewee: Not really because I was pretty on top of that already.

Facilitator: Already, yes.

Interviewee: Yes.

Facilitator: What about talking about health with your family or talking about this program with your kids, partner, have you spoken about it?

Interviewee: I haven't really spoken about it but I've always sort of been on their butts kind of about drinking water and not soft drink and things like that. So they know what dehydration is and they know that if they don’t eat well they're going to get sick more often and things like that.

Facilitator: Yes. So any change, just to clarify, since starting the program or not, that's just usual?

Interviewee: Well I think because of the way I've been eating and things like that they sort of have to follow suit as well so yes, in that respect.

Facilitator: So more action rather than talking.

Interviewee: Yes and there's less biscuits and chips and stuff in the house so they don’t have access to that stuff so the only other choice they have is things that would be good for them.

Facilitator: Yes. Do you normally talk about sort of health with the kids?

Interviewee: Yes, usually.

Facilitator: Yes, good. Because some women just said that they've changed things but they haven't talked about it because they felt sort of a bit self-conscious or they didn’t want to talk about it with their family. Is any of that familiar sort of...

Interviewee: Well it's just me and the kids so it's always sort of been around.

Facilitator: So they're happy to talk about it.

Interviewee: Yes.

Facilitator: What about friends? You did touch on that you said you've gone for a few walks with friends and you’ve had that support from your friends.

Interviewee: Yes.

Facilitator: Any examples of how you've influenced anyone else since starting this program or if you have or if not?

Interviewee: I just get them out to come for a walk, something to do. A bit of a chat...

Facilitator: Yes. Has that been more since starting the program or just in general?

Interviewee: It's probably a little bit more, yes.

Facilitator: Yes, so you're getting them to...

Interviewee: Because it's been more in my head it's...

Facilitator: Yes, makes you change.

Interviewee: Yes.

Facilitator: Again sorry, just touching on that again, do you talk about your health with your friends?

Interviewee: Yes.

Facilitator: Did you have any friends come with you to the program?

Interviewee: No.

Facilitator: No. Did you talk about the program with them? Sorry to be investigating.

Interviewee: No, you're right. Yes, I told them that I went and stuff like that.

Facilitator: Have you shared any of the resources? So you had the manual, did you share anything?

Interviewee: No, not really. But most of my friends are busy with their kids, it's not often we get to catch up without children around.

Facilitator: Yes, of course. Do you feel comfortable talking about your weight, your health, what you eat with your friends?

Interviewee: Yes.

Facilitator: Okay, good. Some people have said they felt really - they don’t talk about their health because they're not comfortable but I'm gathering...

Interviewee: No, as I said, I've lost...

Facilitator: Fitness instructor...

Interviewee: ...quite a bit of weight. I'm used to talking about it.

Facilitator: Good. The last thing I want to touch on is just about your satisfaction with the program and again I want you to be completely honest about this so I don’t want you to say anything - we won't be offended. But based on your experience would you recommend this program to anybody else?

Interviewee: Absolutely. Not enough people are conscious of it and know or will seek out information.

Facilitator: Yes. So you think it's more about - has any programs like this been available in Wonthaggi before?

Interviewee: I mean you've got to take that first step and go to the gym because they do have programs there.

Facilitator: Yes, but like...

Interviewee: A lot of women are too self-conscious about their weight and things like that to even set foot in there.

Facilitator: To set food in there, yes.

Interviewee: Yes. A lot of women I've spoken to want to lose weight before they go to the gym because they feel self-conscious going to the gym.

Facilitator: Yes. So do you think that could have been a barrier for us in terms of getting people into that room?

Interviewee: Yes, it would be but I mean I don’t know how you'd overcome that though.

Facilitator: Yes. What do you think the other barriers were? We didn’t have quite the response that we were hoping for in Wonthaggi.

Interviewee: Okay, yes.

Facilitator: Anything you think would have stopped women from coming? Any obvious barriers or anything you think we could have done better to get more people there?

Interviewee: I think because there's a lot of single parents in Wonthaggi I think one of the barriers would have been having kids looked after while they go to something like that.

Facilitator: Yes. So...

Interviewee: A lot of parents work as well.

Facilitator: Yes. So the timing, maybe an evening session would have been useful.

Interviewee: Yes or even having somebody there to take care of the kids but I mean that's a cost as well.

Facilitator: Of course. In terms of sort of social engagement do you think that Wonthaggi it's easy to get women to put their hand up for things?

Interviewee: No.

Facilitator: No.

Interviewee: No, probably not.

Facilitator: No. Because some people also suggested that perhaps we needed someone local, someone like yourself to promote the program locally and that would have resulted in a better response. What are your thoughts?

Interviewee: Yes, the thing is though this person that you would have had to get to have done it would have had to have a local of social ties in the town as well.

Facilitator: Yes. So that wouldn't...

Interviewee: I find that difficult because I have three children and I am on my own and I see them 24/7 sort of thing.

Facilitator: Yes. They'd need to have a lot of time as well.

Interviewee: Yes.

Facilitator: Do you think it made a difference that the facilitator that came was from Melbourne versus being somebody local?

Interviewee: No, I don’t think so.

Facilitator: Do you have any preference for that?

Interviewee: No, not at all.

Facilitator: The venue - it was hosted at the school as you'd probably remember - do you think that was an ideal venue or do you think that would have stopped other people from other schools not coming or any insight into that?

Interviewee: Well it was held at the North Wonthaggi School and my children go to a different school but I didn’t see anyone I knew. So most of the mothers that came were from the North Wonthaggi School and I would say that most of them would have. Maybe a few smaller sessions that the different schools.

Facilitator: Do you think that would have helped with the women feeling more comfortable?

Interviewee: I think so because then they'd get their friends and come.

Facilitator: Yes. In terms of a group versus an individual session I know you said that you're a fitness trainer and sort of you're happy with your weight. Does it make a difference to you whether it's a group or an individual session? Any preference on how you'd like to receive health information?

Interviewee: I think a lot of women that aren't happy with their weight and the way they look would probably appreciate a more private or smaller group. I mean women are more brave if they can go with a group of friends.

Facilitator: That's what we've had a lot of women say so it's interesting that you say that. We thought that some women, even the bigger women, would feel more comfortable going alone but a lot of them have actually said that they'd rather go with that support.

Interviewee: Yes, definitely.

Facilitator: Is that what you find as well, being a fitness...

Interviewee: I mean it's all personal choice really though but I think more of the women - I don't know, it's hard to say. I think you'll find people that prefer one way and another as well.

Facilitator: Yes, of course, it's always going to be different.

Interviewee: Personal preference, yes.

Facilitator: The level of information, did you think it was adequate or anything else you would like us to have talked about?

Interviewee: One thing I did feel that was missing was the fact that muscle weighs a lot more than fat or it takes us less room and don't put all your hopes on the scale.

Facilitator: Of course. Yes, I agree, that's a good point.

Interviewee: Yes. But just the ratio of muscle and fat and maybe go more into the protein and carbohydrates and things like that more than nutrition dietary side.

Facilitator: Yes. Any...

Interviewee: I mean for somebody that doesn't know much about fitness it was probably adequate because there's only so much information you can take in at once.

Facilitator: Yes, I agree and that's why it can be a bit overwhelming sometimes. Is there anything else, yourself being a fitness instructor, that you could recommend that we could change in the future to help motivate women more or any suggestions about how we could improve the program?

Interviewee: No, no more than what I've said. No, I think it was quite good just for women starting out on a fitness journey I suppose.

Facilitator: Yes. Lastly, is there anything else you think we need to know about your community in Wonthaggi that would be important for the evaluation? Any other experiences or anything...

Interviewee: I just think - I mean I've heard some statistics somewhere that said that Wonthaggi is the single mother capital of Australia.

Facilitator: Is it?

Interviewee: Well I'm not sure, it used to be, it could be just hearsay, I don’t know. There's a lot of young mothers down here too that are single. There doesn’t seem to be a lot of - I don’t know. There's lots of single parents down here.

Facilitator: Yes. Obviously that's going to influence...

Interviewee: How often they can get to the gym or how much time...

Facilitator: Food.

Interviewee: ...they have to spend on making their choices and whether they can get to the information sessions and stuff like that as well.

Facilitator: Yes. So do you think if this program was - obviously the program was free but do you think if it did have a cost that would be a problem?

Interviewee: I think so because, as I said, the demographic down here is a lot of people are short of money so I think that would definitely have made a difference.

Facilitator: Yes. Being with your background in fitness, what do you think about be a cost that would be considered reasonable or possible for women?

Interviewee: A lot of my friends don’t join the gym because of the cost and I mean I only pay $8 a week for my gym and for me that's an acceptable cost for the benefits you're going to get out of it. But a lot of people are that short of money they don’t see it that way.

Facilitator: Yes. Do would $5, $10, what do you think would be...

Interviewee: I think $5 or $10. But then again I think any cost would put a lot of people off, especially the single parents. I mean I would have paid it but...

Facilitator: But whether other people would. Yes, we're just interested because we're looking at rolling this program out further and at the moment it's funded but long term...

Interviewee: Yes, well that's it. It's hard for...

Facilitator: But again, it's interesting. So yes, you think sort of $10. What would be your maximum do you think on average?

Interviewee: I don’t think I'd pay any more than $10 or anyone else would.

Facilitator: Yes and that's fair enough.

Interviewee: Most of the people who could afford to pay for it at work, you know what I mean? Maybe an information session would have made it easier to get the working parents in.

Facilitator: Yes and in some towns we did have evening sessions and that definitely did make a difference.

Interviewee: Okay, yes.

Facilitator: But we sort of got a bit more - Wonthaggi was one of the earlier towns that we visited so we started changing our strategies a little bit more but we did have a good response.

Interviewee: Okay, yes.

Facilitator: We did have a good response from Wonthaggi but we started adding a few additional sessions to help boost those numbers a little bit more.

Interviewee: Yes.

Facilitator: But that's a good point about the working mothers.

[Over speaking 0:27:04.5]

Facilitator: Perfect. Well that's pretty much all I wanted to ask from you.

Interviewee: Yes.

Facilitator: Thank you so much for your insight, it's good to hear from different people with different backgrounds so it's great to hear that you're very active and doing so well. We look forward to seeing you in not too long now, in about six months.

Interviewee: All right, not a worry.

Facilitator: Perfect. Thank you so much and I'll send you that voucher now.

Interviewee: All right, thanks. Have a good day.

Facilitator: Thanks for that, Bye now.

Interviewee: Bye.

end of transcript

Participant #BB

full transcript

Facilitator: So I'm not sure, have you got a bit of an understanding about the purpose of the evaluation?

Interviewee: Just what I've been able to glean from reading the manual but it's actually been a couple of months since I stopped using the manual. I've got through it.

Facilitator: Yes, no problem. The purpose of the evaluation, the letter I sent you just described that the purpose of what I'm trying to do is try to work out why some people joined the program and others didn't, to look at any changes you've made to your behaviour and also to look at your satisfaction with the program. Is that okay?

Interviewee: That's fine.

Facilitator: Perfect. So the interview takes about 20 minutes and then as a token of appreciation I'll send you a Coles Myer gift card and if you don’t mind is it okay if I tape record the interview? It's going to be purely privately kept, it's just so that we can make sure that we've accurately recorded everything that you say.

Interviewee: Yes, that's fine.

Facilitator: Perfect. So I'm putting you on speaker but I'm the only one in the room so don’t worry, no one else can hear anything you say and everything - you won't be identified by your name. It's all completely confidential. So to start off with I was just hoping that you could describe a little bit about your town in Yarram just in terms of the size, the age of the people, anything that you think makes your town a bit unique. I'm trying to compare the different towns to each other and just getting a bit of insight into the town you live in.

Interviewee: Okay. Well I'm actually 20 kilometres from Yarram but it is my main town. The population I think it's hovering between 1500 and 2000.

Facilitator: Yes. Do you consider that to be a small town or a larger town, medium?

Interviewee: I consider that to be a small town. The town's economic prospects aren’t that great at the moment so I think that affects people's morale, mentality and lifestyle choices as well. There is an ageing population. At the same time, having said that, you hear a lot about the younger people and their needs. Child care's a huge need in our town. I suspect a lot of the - I'm probably one of the few in your study in this area who don't have children. So there is a healthy population of little ones coming through but I'd say the majority is...

Facilitator: Older.

Interviewee: The township's population are older, yes. A lot of people have come from farming properties and move into town when they need to sell the farm, it's beyond them, they pass it on to the next generation, that type of thing.

Facilitator: Yes. You mentioned child care. Is there access to child care at the moment or not?

Interviewee: I'm probably not the right person to ask. Just from glancing over the local newspaper I know it's a real issue.

Facilitator: Yes. You mentioned...

Interviewee: People wanting to work an hour's drive away where major towns are and there's nowhere for their children to go after school.

Facilitator: Yes. You also mentioned employment. Are there many opportunities for employment?

Interviewee: No.

Facilitator: No.

Interviewee: There was a dairy plant here run by a conglomerate in the Murray Goulburn that basically shut down a few years ago and left a lot of people unemployed and there are a lot of empty, vacant shops in town too.

Facilitator: Yes. What about when you think about the town are there common health problems? I know it's a very generalised question, sorry, but would you consider people to be on average quite healthy or are there any common health problems?

Interviewee: I wouldn't say that people were generally healthy. I'm not from any medical background and I don’t live in town so I'm probably not well equipped. But just having a look around and knowing the people that I know I'd say no the population generally isn't a healthy population.

Facilitator: Is that indicated by their weight or what makes you think that?

Interviewee: It's indicated by not only weight but knowing that so many people have diabetes, so many people have heart troubles, so many people have cancer. In a small town you generally know who's got what and if I took say 100 people that I knew I could list just about everybody's health ailments.

Facilitator: Yes. In terms of obesity per se do you think that - you mentioned that that's a problem, is that a problem?

Interviewee: I don’t think it's more prevalent here than it is anywhere else in Participant. But I do know that it's a problem and I do know a lot of people who are constantly trying to lose weight.

Facilitator: Do you think there's many opportunities for the people that are trying to lose weight to access healthy foods, exercise, recreation facilities, is there much available?

Interviewee: Look I hear people make excuses but if I stand back and analyse there's no excuse. There are gymnasiums, there's a huge sports culture in the district. There are opportunities to exercise. We've got the 90 Mile Beach at our fingertips. We've got walking trails through national parks. There are bike tracks. There are rail trails. The opportunities are there. Community subsidised health and fitness programs, yoga and strength training.

Facilitator: Perfect, lots of opportunities.

Interviewee: So much people can do. There's golf. There's tennis. There's badminton. You name it pretty much it's here except ice skating.

Facilitator: What about good food, healthy sort of fresh fruit, vegetables, meat, diary, is that prevalent?

Interviewee: We do suffer from lack of a good fruit and veg shop. We do suffer from that. But there's Woolworths here. There's another small supermarket that's got really lovely fresh fruit and veg from a nearby town. I know it costs a bit more and that'd probably put people off but people need to analyse will I spend $2 more on a kilogram of apples or buy a bag of chips and I'd say most of them would go for a bag of chips which is really sad.

Facilitator: Do you think the culture is possibly to go for the cheaper options?

Interviewee: Well yes they'd only ever buy something if it appeared cheap to them. But I think a lot of people in this town have not come from prosperous backgrounds. They've been strugglers, battlers and the notion of paying $4.99 for a kilo of apples they just say no, we're not getting them, they're too expensive. When if you buy a kilo of apples at $4.99 you realise an apple's really only costing you less than $1. It's a very affordable, cheap, healthy option.

Facilitator: Yes, of course. But...

Interviewee: People don’t see that.

Facilitator: Do you think it could also be to do with education or more for the cost?

Interviewee: Yes, it's about education and it's about changing the way people think.

Facilitator: Do you think that's difficult to do in a small town?

Interviewee: I think it's probably difficult to do anywhere where people are of a certain age and they won't be told and they won't think outside the box.

Facilitator: Yes. Do you see many groups of women walking together or a lot of walking groups of women? Anything that makes you think that a lot of people are active?

Interviewee: I know personally of quite a few walking groups, women that meet regularly. I know other women that meet on an impromptu basis where are you going, let's go for a walk, that sort of thing. I think you've just really got to put yourself out there and make it known that that's what you want to do. There are some women that walk, that cycle, just informal arrangements that go for a hit of golf.

Facilitator: So on average would you say that most people are active or half, any approximation?

Interviewee: I would say probably a quarter.

Facilitator: Yes, not very active. In terms of socialising we're interested in, again, that kind of notion of social opportunities which sort of encourage people to - do you think there's many places to be social?

Interviewee: I think if you ask most people if you a vox pop of the main street of Yarram opportunities to be social they'd say yes, there's two pubs in town. That's probably it.

Facilitator: What do you think the other opportunities to be social would be?

Interviewee: Well I'm part of the local walking group in town. There is a specific walking group and we go on walks every three weeks and trips and things. I consider that to be social. There's garden clubs. There's book clubs. There's lunch clubs. There's sport clubs. A lot of other social activities revolve around football and netball. That's where a lot of people socialise. But I must admit because I don’t have at school I imagine there are a lot of social activities based around schools.

Facilitator: So on average, if you're willing, there's lot of opportunities.

Interviewee: Yes. There's no reason why anyone needs to sit at home in this little town. There's something on all the time.

Facilitator: That's great. So if we now think back to when you first joined the program can you think of any reasons that motivated you to join the program?

Interviewee: Well it was mentioned to me by a friend of mine who's a pharmacist. Because it was a Monash University study I thought that it would be run well and that it'd be something that wouldn't be too taxing on me and that I could participate in quite easily and it probably wouldn't fizzle out after a few months like most studies do.

Facilitator: Have you had studies come that have sort of collapsed after a few months?

Interviewee: Look I can't think of anything in recent years. Thinking back to more when I was in university myself which was a long time ago and I just know a lot of things never get off the ground.

Facilitator: Yes, course. So you said you came with your friend - I think I remember your friend actually, Katie, right?

Interviewee: Yes.

Facilitator: Yes, I remember Katie, she was lovely. Did most people in that group come together or do you think some people came alone?

Interviewee: Look I got there and there was a large number of women there already and I would say that they were all mothers from - because it was held at a primary school. I'd say the majority of them were mothers from the primary school just judging by those factors and their kids kept running in. So I'm probably the odd one out there.

Facilitator: Yes. Do you think that holding it at the school - because the whole program is not targeted at just mothers - that may off put other women for joining because of the venue?

Interviewee: I think it's probably influenced a lot of women thinking that it's probably something associated with the school.

Facilitator: Yes. So if we were going to run it...

Interviewee: You were aiming for a particular age group weren't you?

Facilitator: Yes, we were aiming for 18 to 50 and that's why we went through the schools. But where would you recommend holding it that would perhaps be a bit more neutral and - because it didn’t matter whether you had kids or not.

Interviewee: No.

Facilitator: Anywhere else you'd recommend holding it so it appeals to more people?

Interviewee: Perhaps the local hall in town, the theatre right in the middle of town or maybe one of the church halls. That would be a cheaper, more affordable option for you. Or even there are cafes in town. We could have met - there's a room upstairs above the café where people would have been happy to congregate and they would have been happy to accommodate you as well.

Facilitator: Yes. Do you think that could have maybe improved - we had quite a good response from Yarram but do you think that that may have improved it a little bit more?

Interviewee: Yes, I think so. I only heard about it through Katie. I didn't hear...

Facilitator: Yes, that was what I was going to ask you. So what about - maybe that was another problem, a lack of awareness perhaps.

Interviewee: Yes, perhaps. Because it appeared to me that you really pretty much got in Yarram I'd say 95 per cent of the people there were school mums...

Facilitator: Yes, there were.

Interviewee: ...at school.

Facilitator: So how do you think we could improve that awareness to reach more women?

Interviewee: Was there anything sent to the local paper?

Facilitator: I think there was. We actually did quite well in Yarram but we went to the whole Participant region and some of the other towns we didn’t do as well. We started changing our strategies to focus more on the newspapers so I think perhaps there was something small in the Yarram paper but I don’t think we had a whole story.

Interviewee: Perhaps distributing flyers at the local health centre, the doctor's surgery and the pharmacy might be an idea as well.

Facilitator: Yes, that's actually where we met Katie.

Interviewee: You're only coming into contact there with people who have health needs so it's very tricky I know. Yes, perhaps even something like an interview done with the local paper.

Facilitator: Yes, that would be good I think.

Interviewee: A story might have grasped more people from different walks of life.

Facilitator: I just spoke to someone from Stawell about getting someone local on board. They thought that for Stawell you really need someone local on board in order to help get more women to a program. Do you think that's the same in Yarram, that you need someone local to promote the program or perhaps not as important?

Interviewee: That's not as important. I think Stawell's got a much larger population than Yarram, hasn't it?

Facilitator: Yes, it's quite big.

Interviewee: Yes. You just prompted me to think there's also a tiny little community newsletter called *The Drum* in Yarram and a lot of people from all age groups are picking that up. It's a free little newsletter that comes out every month. That would be a great place to publicise it.

Facilitator: That would have been a good idea as well. Are there any other reasons why you think that other women in the town didn't come along? So you've mentioned awareness which obviously is going to be a big barrier. Any other barriers?

Interviewee: Yes, I think - this is a generalised opinion and I have no way of backing it.

Facilitator: No, that's completely fine. We are all about hearing your opinions.

Interviewee: Yes, my hypothesis is that a lot of people in town, particularly the younger mums, may not even have finished Year 12. So things like studies run by universities, they'd probably be completely dismissive of it. I have a friend who thinks that I am from a different world because I went to university and she considers that to be lacking on her part. I said to her, look it makes absolutely no difference to me whether my friends have finished primary school, let alone gone to university, it's got nothing to do with who people are. But I think there's just this real social thing about not being good enough.

Facilitator: Yes. So do you think the not being...

Interviewee: It might have also been a language - like a literacy barrier as well.

Facilitator: When you say not being good enough you mean that some people probably didn’t - do you think it's about the comfort of coming to the program or they just don’t feel...

Interviewee: I think a lot of them may have felt that they might have been asked to do things they weren't capable of doing like...

Facilitator: Filling out questionnaires.

Interviewee: Yes, filling out questionnaires. This interview that we're having, if this is in a written format people wouldn't touch it with a barge pole. They just wouldn't. they couldn’t be bothered, wouldn’t want to, would feel inadequate. There's a very low level of literacy I've noticed. Even people who are willing to write their English is poor, their spelling is...

Facilitator: So you think it could be a bit...

Interviewee: …horrendous.

Facilitator: Maybe perhaps the program, people would have been scared because they thought maybe it would be a bit pitched too high.

Interviewee: Yes. Yes, they might have thought that there was too much written involvement. Perhaps they weren't even sure about how much involvement there would be from you over the period.

Facilitator: Yes. So that sort of anxiety about overcommitting perhaps.

Interviewee: Yes.

Facilitator: Did you find that the program was pitched at a level that perhaps was too high?

Interviewee: No, not at all.

Facilitator: Good because it's interesting that you say that because I've only done a few interviews so far. Yarram was one of the first towns that we started on and no one's mentioned that before. But it is important to keep in mind that perhaps the level of education is going to influence our program. Also the timing of the session, we had a day session, do you think that was a problem in terms of people's commitments?

Interviewee: Look now I think of it I know there's another woman, I'm not sure if she's got kids at the school or not, she possibly does. But she was allowed time off work to attend and I don’t think there would have been - from a lot of employers there wouldn't have been a lot of support. I know who her employer was and I'm not surprised by that particular employer. But yes, I'd say there are a lot of women who might have been good for the study that simply couldn't get the time off work.

Facilitator: Yes, so perhaps an evening session would have been ideal.

Interviewee: Yes.

Facilitator: When you said a lot of employers wouldn't have given support, any reasons as to why you think that?

Interviewee: Because business tends to run on a very tight shoestring here and I know parents are always requiring time off to go and see their child at the school or see their child at a concert or go the sport swimming day. Because unemployment is such a big issue here people don’t want to jeopardise their jobs. So taking time off work is just...

Facilitator: Not part of the culture.

Interviewee: …participate in something that's voluntary. It's just not going to be on their radar. They're not going to consider it for a second. It would be no, it's on at that time, I can't go. There wouldn’t be another second's thought.

Facilitator: As we progressed through the towns we actually starting putting an evening session. We found that really did help the response rate as well so that people that were working could come. I now wanted to have a look at what you've changed since being in the program. So I'm specifically interested if anything's changed since you've been in the program. Whether you found at the session whether you learnt anything new or if you remember any specific messages that we talked about in that program, health messages.

Interviewee: Look I haven't got a lot out of the program personally.

Facilitator: That's okay, let's be honest.

Interviewee: There is one thing in the book that I think of and I've mentioned it to a few people and it's so frivolous. But you've got a page - I'm flicking through it now trying to find it - where you say the equivalent of a Tim Tam is five hours' walking or a glass of beer is...

Facilitator: Yes, I know the page you're talking about.

Interviewee: I really quite like the fact that the thing on there that has the least impact was a glass of champagne as I love champagne. That was...

Facilitator: That was sort of something that you found interesting.

Interviewee: Yes.

Facilitator: What about when we were in the session we talked about six key health messages. Don't drink soft drink, having breakfast regularly, getting your exercise in every day. Was all of that familiar to you or was any of that new?

Interviewee: No, nothing was new. I've always been conscientious about my weight and my appearance and having a balanced lifestyle. So to be perfectly honest I haven't learnt anything.

Facilitator: Yes, that's okay. Do you think that's because you've always been quite health conscious?

Interviewee: Yes. Well when I was growing up eating junk food was something that was had very occasionally. We hardly ever had takeaway. Of course now I live 20 kilometres out of town I very rarely have takeaway. I watch what I eat and I always think to myself do I really need that? I'll have a piece of chocolate when I really, really feel like a piece of chocolate but I'll have one mouthful of chocolate, I won't have a bar. I'm just very conscientious of the fact that if I do eat a block of chocolate I'll probably feel sick, I'll probably be sick and I really don’t want to spend 24 hours trying to walk it off.

Facilitator: No, not if you can avoid it.

Interviewee: No.

Facilitator: But the information - so some people have said similar things to you about you're not learning anything new. Did you find it useful as a refresher? Did you find that useful or you already quite...

Interviewee: No, I didn't find it useful as a refresher.

Facilitator: Yes. If you were to describe...

Interviewee: The messages have been engrained in me since I was young, these health messages and healthy living messages from my parents and it's just I won't forget them, they're part of me.

Facilitator: Do you think that would be the same with many other women in the town?

Interviewee: No. I don’t think that at all, no.

Facilitator: So many for them it would be useful?

Interviewee: Yes. If they put the effort in, yes. I hope they've put the effort it but I'm not sure that they all have.

Facilitator: When you say the effort you mean to take on those messages?

Interviewee: Yes. I can't tell you how many times I've stood at the supermarket and watched people I know with young kids stand in front of me and unload a trolley full of chips, chocolate, snacks, frozen food like stuff you shove in the oven full of grease and...

Facilitator: Cheap.

Interviewee: ...polyunsaturated fats. And it's actually not cheap. That crap costs a lot of money. It adds up. It's extraordinary. It's cheaper to eat fruit and vege and make from scratch. But it just makes me shudder when I see what people are putting in their kids' mouths.

Facilitator: If you were to describe this program to somebody else, a friend in the town, how would you describe it? What do you think the main aims of this program were?

Interviewee: It's so broad but it's to promote the message about having a healthier lifestyle and maintaining a healthy weight and body image and incorporating exercise into your lifestyle so that all of these things don’t become overwhelming, it's just a way of living. So you don’t have to worry about becoming obese. So you don’t have to worry that you're lacking in a particular vitamin and you can alleviate any future health issues by adopting a good way of living now. one thing that I have mentioned to a few people from the study was that I think it was from a particular age - I think it might have been 35 or 40 - people commonly put on 600 grams a year. That's a statistic that I think might motivate some people into paying attention now. I thought that was interesting.

Facilitator: Yes. So since starting the program you said that you've spoken - has it encouraged you to speak more to your friends or your social network about health since starting the program?

Interviewee: No, no more than usual. I mean women are obsessed by talking about their weight and their appearance. I was somewhere last night and someone was bemoaning the fact that she tried on four outfits before she could find one that she could fit into and that she's going to start walking an hour every morning starting that day. So instead of doing her 20 minute walk she was going to do an hour.

Facilitator: That's good.

Interviewee: Yes, it is good. But you hear these things all the time. It's sort of that yo-yo diet thing that I hope I never fall into, maybe because my mum's been like that all her life...

Facilitator: And you've...

Interviewee: I'm determined not to let it happen to me.

Facilitator: Yes, of course. So since starting the program - just to confirm - would you say you've influenced anybody else's health or talked to them or helped anyone make any changes to their lifestyle?

Interviewee: No.

Facilitator: No. What about to your family's?

Interviewee: No because I don’t live anywhere near my family. I don’t have much to do with them. And I'm not married and I don’t have kids so if you mean immediate family...

Facilitator: Yes.

Interviewee: ...I don’t have one.

Facilitator: Yes, okay, no problem. What about since starting the program - I know you were already very health conscious - but have you thought more about your weight or your health?

Interviewee: No.

Facilitator: Exercise?

Interviewee: No.

Facilitator: No. That's okay, I'm glad you're being honest. If you look at the specific value of each of the components of the program, so since we saw you we had that one healthy lifestyle group session, we had the program, the manual that you said you've got in your hand, SMS messages, we've got the website and the phone coaching. Did you find any of the individual components more useful than others?

Interviewee: Probably the session was the most helpful. Possibly not for me but for everybody participating in our area the session was probably the most helpful. I've got to say I really doubt that most of the people at that session have more than flicked through the manual.

Facilitator: Yes, you're spot on with that. We've done a bit of an evaluation of that and it seems like people used it at the beginning but then not so much at the end, like not so much now.

Interviewee: I'd be surprised if most people picked it up after seven days of having gone to that session.

Facilitator: Yes. When you said you thought the group session would be most valuable to other people in your town can you explain a little bit further why you think that?

Interviewee: Well that's probably the only way that you're going to get their attention and interact and impart any information to them because, as I say, when they went home I'm pretty sure that nearly 100 per cent of the people there would not have touched their manuals. Would have got your text messages and rolled their eyes like oh, I haven't got time for that kind of thing.

Facilitator: Any reason why you think that? Is it because of something?

Interviewee: I'm very cynical.

Facilitator: No, that's okay. Your insight's really useful to us.

Interviewee: I'm just thinking about the particular women that were there. I know some of them - not very well - and I think life has just been overwhelming them with three or four kids running around. They don’t have time to brush their teeth some days so I'm not sure how they were going to find time to read this manual through.

Facilitator: Yes. Do you think maybe health not being a priority could be one reason why they wouldn’t read the manual?

Interviewee: Yes, I think so. It's just I think that they would think that they'll get round to it. When the kids are at school I'll do this now and when the kids are at high school I'll do this. It never happens. When the kids leave home I'll do this, never happens. I think it's sort of got to be something that you've had built into you from when you were a kid, I really do.

Facilitator: Yes, I agree with you. It's really, really important. You got a phone call a few weeks ago about setting an action plan and setting goals on the phone with one of the research team members.

Interviewee: Yes.

Facilitator: Did you find that helpful or do you think others in the town would have found that helpful to re-motivate them?

Interviewee: It may have helped re-motivate some. It might have made a few people think ooh, I better do something.

Facilitator: What about you?

Interviewee: No. In fact I think I was told that it was going to be about a 20 minute call and I think it lasted five minutes. I didn't have much else to offer because I'm...

Facilitator: Yes, if you're doing it already.

Interviewee: I haven't got anything from - yes.

Facilitator: That's okay. So for you if we took out - maybe I won't ask based on your experiences but I'm just looking into how we can improve the program in the future. Are there any elements of the program you think we could take out that wouldn't affect people's motivation or wouldn't affect people's progress in the program do you think? Be it the text messages, the website, is there anything you think would be less important than others?

Interviewee: No, I think the text messages are probably a good idea because they help remind people that they are participating in a study which otherwise they'd probably forget about so I think the text messages are a good thing.

Facilitator: The phone coaching?

Interviewee: Yes. I wonder if a lot of the women felt a bit stressed by it, that they don’t really have time for it. I don’t know what sort of response you got when you rang around last week and said I'll book you in for a particular time.

Facilitator: Mixed.

Interviewee: I know...

Facilitator: Quite mixed, very varied. You have some people that are willing to help and jump on board immediately and you have other women that are not.

Interviewee: I think you'd probably be able to sort out pretty much from the response from that phone call where you were trying to book in phone calls...

Facilitator: Yes and that's what I've...

Interviewee: ...who's participating and who's not.

Facilitator: Yes and that's what I've done. If I didn’t get much response, two phone calls or not, I just moved on.

Interviewee: Yes.

Facilitator: Because we don’t want to harass people and we found with the phone coaching that some people were very difficult to get onto and we really didn’t want to have to harass them so then we just sent a letter saying if you'd like to speak to us for a chat give us a call.

Interviewee: Yes.

Facilitator: So since you've started the program - just again we already mentioned this - but were there any other specific behaviours that you think you've changed? Any changes to your exercise, diet, self-monitoring, even weighing yourself that maybe you've changed?

Interviewee: No.

Facilitator: No.

Interviewee: No.

Facilitator: Have you accessed any other community services since joining?

Interviewee: No.

Facilitator: No.

Interviewee: I'm part of a lot of community groups and I've very active in our community.

Facilitator: Great, yes. When you say active in your community you mean in terms of on community groups?

Interviewee: Yes, community groups and volunteering to organise things. I run the garden club in town. We've got about 100 people that come from time to time. It's a monthly thing that I run. I'm part of two book groups. I play golf and walk.

Facilitator: So you're very active in the community, that’s great.

Interviewee: Yes.

Facilitator: Sorry, I don’t think this will be applicable to you because you said you haven't made any behaviour changes. But did you find in the manual and at the session talking about goal setting, problem solving, relapse prevention or action plans useful?

Interviewee: No. In fact I think when I had my last phone interview a few weeks ago I've always abhorred goal setting and setting objectives and plans and all that sort of stuff. It just doesn't sit - like I think it's a waste of time. I'm sorry but it's just not part of my philosophy. I come from a setting, you know, where we were trying to encourage our clients to set goals, long term goals, short term goals and I just think it's all a waste of time. I think if you're going to do something you're going to do something whether you write it down on paper I just think it's a waste of time. I'm just a more proactive, if you're going to do it, do it. Don’t talk about it, just do it.

Facilitator: Okay, perfect. So we already touched on this but again just to confirm. Do you have any specific examples of how you've influenced anyone else's food choices or exercise?

Interviewee: No. I think I said before that I don't think I've influenced anybody.

Facilitator: That's okay. The last question, about your satisfaction about the program, based on your experience do you think you'd recommend this program to anyone else in the town?

Interviewee: I'd think very hard about who I'd recommend it to because I think I'd want to sort of analyse that person's personality. And if I was going to recommend it to anyone and perhaps refer them to you about it I'd want to make sure that I wasn't wasting your time or theirs. I'd want to make sure that they really wanted this; that they were looking for some assistance. I know a lot of women who have been to Weight Watchers and I think one of the pharmacies have a Kate Morgan weight loss program or something. There are waves of women trying to lose weight and they'll lose a few kilos and it's all going swimmingly and then you see them two months later and they've put an extra 10 kilos on.

They starved themselves for a month and then all of a sudden they figure that they'll just go back with a vengeance to what they used to. So I'd think really hard about who I'd recommend it to so I wasn't wasting your time or theirs.

Facilitator: I guess the point of our program is to reach anyone of any weight but perhaps it would be more appealing to people that had less health knowledge.

Interviewee: But whether or not they'd be willing to embrace it would be another thing. There are a lot of people around here who just don’t want to be helped quite frankly.

Facilitator: What do you think we could do to reach those people or to motivate them to come to a session like this?

Interviewee: You'd perhaps need something like an article in the paper, a lengthy article, or maybe even like an ad and pay for it to go in the local paper explaining what you're doing and how you think you might be able to help these people. Get your message across in a non-threatening way so it's...

Facilitator: Appealing.

Interviewee: You're not preaching to them, it's something they're invited to attend and perhaps written in a sympathetic tone as well.

Facilitator: Yes. Do you think incentives - the last lady talked about having it at a café and providing coffee. Do you think that would help?

Interviewee: Yes. I think if you even - yes, just coffee and maybe a tray of - I know you're trying to encourage healthy eating.

Facilitator: A fruit platter.

Interviewee: A fruit platter or a small piece of fruit cake, [36:16.2] oats or little oat biscuits or something to have with your coffee. But yes, I'd say definitely a coffee at the café.

Facilitator: You think an incentive would help.

Interviewee: Yeah, a lot of women there, yes. Just make sure it's skinny.

Facilitator: Yes, of course. Or they can have herbal tea. What about in terms of the facilitator? So the facilitator that ran the program was obviously from Monash University. Any comments on whether you think that the program would be taken up if the facilitator was somebody local versus someone from Melbourne?

Interviewee: No, I think - I can't remember the lady.

Facilitator: Her name was Kate.

Interviewee: Yes. I thought she was very good and I thought she was very approachable. But I think I did hear a couple of women say it's all right for her, look how skinny she is, that sort of a thing but you're going to get that. But how can you be encouraging people to...

Facilitator: Be healthy.

Interviewee: ...be healthy and have a good body weight when the person standing at the front of the room is not.

Facilitator: So you think it's more not based on where they're from, more based do you think maybe the facilitator's body shape is more possible it was more threatening?

Interviewee: No, I don’t know it was more threatening. I thought it was good that it was Kate and that she was from Monash University and that she - I think you need somebody with some sort of credibility. And some sort of authority and someone who clearly knew what she was talking about and practiced what she preached as well. So I don’t think somebody local. Honestly, I couldn’t think of anybody.

Facilitator: That would be appropriate.

Interviewee: I mean I couldn't think of anybody.

Facilitator: What about group sessions versus individual, so for you or even your opinion on what other people would have preferred?

Interviewee: I think the group sessions are good as long as you don’t start asking individuals to contribute about their personal habits because I think, particularly in a small town where everybody knows everybody, they're going to start to feel threatened. So group sessions are good. You'll get people there. You've got some camaraderie. A lot of the women are shy. A lot of them aren't educated so they're not confident so they are shy, they like safety in numbers.

Facilitator: Yes. So you think that would be a better uptake than the individual.

Interviewee: Yes. The individual - you know how you took us off and weighed us and that sort of thing. That's all fine. But yes, general things where the girls can go with their girlfriends, like they can get together and have the morning together they'll feel more comfortable there.

Facilitator: Yes. Because we had some women saying...

Interviewee: They don't like being singled out.

Facilitator: Yes. We had some women saying that they wouldn’t want to come to an individual session because they'd find that a bit more intense and a bit more - so that to them makes - they don't want to be with a stranger. They'd prefer to come with their friends.

Interviewee: Yes, they'd find it confronting. I really do think that that's how they would find it.

Facilitator: Yes. Lastly, is there anything else you can recommend that we could change to make the program better?

Interviewee: To be honest I haven't given a lot of thought to it. Off the top of my head, no I can't think of any.

Facilitator: It sounds like the program was probably pitched to someone with less health knowledge than yourself. But what about if you were thinking from the perspective of the women in your town, anything you think we could have done differently?

Interviewee: No, I think it's targeted them quite well. I really do. It's just I would just be - I wouldn't be surprised if they just haven't got around to reading the manual and participating as you would have liked them to. I wouldn’t be surprised if a lot of them bullshit you and pretend that they've done this and done that. I wouldn’t be surprised at all.

Facilitator: Why do you think they'd do - do you think it's because they'll just say what they think we want to hear?

Interviewee: I'd say so, yes. Because you are from Monash University and I think that probably - a lot of people in this town wouldn't have had anything to do with a tertiary institution and they'd be trying to make you think that they have been participating whereas in fact I think a lot of them won't have been. That's just between you and me.

Facilitator: No, of course. So you think that they will just say what you want to hear and what they think.

Interviewee: Yes.

Facilitator: Maybe what they think they should be doing.

Interviewee: Yes. So it's going to be hard for you to get an honest assessment I think.

Facilitator: At the year time?

Interviewee: Sorry?

Facilitator: You said the numbers when we come back in six months?

Interviewee: Yes. I just think even these phone interviews. I wonder how much [unclear 0:41:18.2].

Facilitator: Okay, great. Anything else you want to add about the town or anything else you think's important for the evaluation?

Interviewee: Nothing coming to mind.

Facilitator: Great. Thank you so much again for your time and all your insight. It's been really, really valuable.

Interviewee: I hope I haven't sounded like...

Facilitator: No and it's good that you're honest and it's good to get different perspectives because everyone has something different to add which all forms part of the picture that we're trying to create.

Interviewee: Yes. Every town's full of the same dynamic of people, it's just which ones you discover.

Facilitator: Yes and it's good to hear some of the things you say about the barriers and what to expect when we come back in six months' time.

Interviewee: Yes.

Facilitator: Perfect.

Interviewee: Okay.

Facilitator: Thank you so much for your time and I'll send you that gift voucher in the mail today.

Interviewee: Okay, thank you.

Facilitator: Thank you.

Interviewee: Bye.

Facilitator: Bye.

end of transcript

Participant #SW

Full transcript

Facilitator: So as I mentioned to you, the point of the evaluation is to get your feedback about the program. But the first thing that I wanted to start off with is for you just to describe your community in Wonthaggi to me. So in terms of the size, the age of the community. Anything you think that will be beneficial for us to know.

Interviewee: It’s not overly large. I think it’s – I’m not sure how many people live here. I really don’t know how many people live here. I think there’d be …

Facilitator: But would you consider it a large, a small …? What would consider the town in terms of size?

Interviewee: Probably a small town. But I’ve seen smaller. A lot more young people – well, when the desal was here, a lot more young people moved here.

Facilitator: So a lot of young people there now?

Interviewee: Mainly a lot of a – there was a lot of elderly people. But there are a lot of – they seem to get sent down here for some reason. I don’t know why.

Facilitator: Young people?

Interviewee: Hmmm.

Facilitator: When you say young, do you mean people with families or like more …?

Interviewee: Young teenagers and young parents, yeah. They seem to get sent here. But there’s no employment down this way.

Facilitator: No employment.

Interviewee: No.

Facilitator: So if you were looking for a job, what kind of areas could you find work in?

Interviewee: Well, the did get a lot of people in the new parts when they built the new plaza before [0:01:41 unclear]. But there’s really not much unless – school kids could probably get a job, but it’s really hard for adults.

Facilitator: Do you feel that it’s a health conscious town? Do many people talk about health or there are common health problems that you think – in the town?

Interviewee: No, I don’t think so. Just the usual, what you get, I suppose. The usual ailments that people get. But no, I don’t think so. No.

Facilitator: Would you consider the town to be active? Physically active?

Interviewee: There’s a lot of people that go around – there’s a lot of walking tracks. So there’s a lot of people – and there’s a couple of gyms.

Facilitator: And gyms. And a lot of people are walking?

Interviewee: Oh yes, there’s a lot that jog and walk and, yeah.

Facilitator: Do most of them walk together or more people by themselves or is there walking groups?

Interviewee: I’ve seen elderly walking groups. You know, elderly groups. But a lot of people just go couples or usually on their own or within families. Yeah.

Facilitator: What about access to health services? Would you think that there is adequate health services?

Interviewee: I think we’ve got a health centre for young mums. There’s two doctors’ clinics. But there’s not much for, you know, children with disabilities. There’s a disability school. But it’s nothing to do with – oh, I don’t know. There’s not much up – because it’s a small country town. It’s not like down Melbourne. The kids are [0:01:41 unclear]. There’s not really that up here, unless there’s a seminar.

Facilitator: Any comments on – in terms of obesity, do you think obesity’s a problem for the town?

Interviewee: No. Look, there’s really not that – well, I don’t really take notice of people really. No, I haven’t really seen many overly large people. I mean, you see them everywhere but I don’t think there’s a common problem here.

Facilitator: Do you hear people talking about their health much or about their weight? Is there much talk amongst women about their health?

Interviewee: Well there is with me and my friends. But that’s just more my thing. I think most people talk about themselves, they’re just not happy with something are they?

Facilitator: Yes, no. No-one’s usually very happy with their body. So if you think back to why you decided to join the program, are there any factors that motivated you specifically to join this Healthy Lifestyle program?

Interviewee: Yeah, well I really wanted to get fit and lose some weight. But it didn’t happen because during it I lost my mum and – which sort of I tend to eat a lot for a comfort sort of thing. That’s how I deal with stress. Now I’m waiting for a foot operation, so I really can’t walk so I'm not very active at the moment, so I’ve actually put on weight. Just due to me, nothing else though.

Facilitator: Just due to the circumstances.

Interviewee: Yeah.

Facilitator: Was there anything else that motivated you to come to the program? Did you have friends going that encouraged you to come or anything else?

Interviewee: Yeah, there was a couple of my friends were going and I thought “oh, we’ll give it a try and see what it was like”.

Facilitator: Do you find it easier to go to a program if you’ve got friends going? Or easier to go by yourself?

Interviewee: I’d rather go with friends. But I have been to things on my own.

Facilitator: Now thinking back to the session in Wonthaggi, there was only a small group of women. Can you think of – as you’re living in the town, any reasons that may have stopped other women from attending?

Interviewee: Well I don’t know. Because I think you’d rally up the school. That’s how I joined. I’m not sure if you were anywhere else.

Facilitator: Yep, we did.

Interviewee: But I don’t really know.

Facilitator: Timing of the session? Do you think that could’ve been a problem?

Interviewee: Because at that time when I walked through the gates they might not have been there. I’m not sure.

Facilitator: What about timing of the session? Do you think that may have been a problem? It was during the day.

Interviewee: Well a lot of mums work. Well I found it right, because I wasn’t thinking at the time, but a night session might have been good. Like say – not late, but like a town hall session or something like a club.

Facilitator: What about the venue? Do you think that made a difference to whether people would come or not? Because it was at the school?

Interviewee: It might have put off some. I’m not sure. But that’s where my daughter goes.

Facilitator: Why do you think it could’ve put off some?

Interviewee: Pardon?

Facilitator: How come you think it may have put off some?

Interviewee: Well a lot of parents don't really go to the school, you know? Like they just drop their kids off and that’s it. They don’t put in. Whereas I’ve always been a the school with my kids. I'm always there helping, but some parents just drop in and say – you know what I mean? They might just drop them at the gates.

Facilitator: Yeah, don’t usually go there. Yep. And seasonal factors? Anything that would’ve interrupted in October? Anything? Bigger community events or anything going on?

Interviewee: In October?

Facilitator: Yeah. That’s when the session was last year. Any stand out?

Interviewee: I don’t think there was anything happening in October. Only the motor cycle grand prix thing. That’s about it.

Facilitator: But nothing else. No. We found that we came around Christmas, around harvest time in some of the other towns, and that was a bit of a problem. But I don’t think that would’ve been a problem in your town.

Interviewee: No.

Facilitator: Now as you – sorry. As part of the program, you attended that group session in Wonthaggi. We had the phone call that you got a couple of weeks ago. We gave you the manual and the SMS’. When you think about the program, is there something that stands out to you in terms of what you learnt in the program? Like what information you learnt?

Interviewee: Oh, I don’t know.

Facilitator: So if I was going to ask you – if you were going to talk to someone, a friend, about the program, how would you describe the program? So if you were going to start with “the program was about …”?

Interviewee: Oh, I see, yes, yes. I found it quite interesting actually. The little book. I didn’t read it all when I had the phone call. The phone call was obviously a bit more of a motivator.

Facilitator: But what did you – if you were going to describe the program, what information would you say you learnt about? Or what information was refreshed for you?

Interviewee: I don’t know. I liked – what was the girl’s name? The lady that was doing all the talking. I found her quite interesting to listen to because I liked – she was quite – she was on our path. She’d been there, done that. I’m like one-on-one, like when someone’s actually doing a speech. Yes.

Facilitator: But is there anything that stood out to you? So any key messages about weight or about food? Was there anything that stood out to you, if you could summarise something that you’d learnt?

Interviewee: I don’t know. There probably was, but I’m – I haven’t really done it properly, which is what’s happened.

Facilitator: No, that’s completely understandable. Okay, no problem. What about – since being in the program, have you started thinking a little bit more about what you’re eating? About your exercise and about your weight more? Or would you say it’s been the same?

Interviewee: Oh no, definitely more. I’ve been thinking more about it. But I’m just finding it hard to do it, you know what I mean? Like there’s so much gone – I’ve got to have – I can’t really walk, but I’ve been walking with one of my daughters every night. But the pain in excruciating.

Facilitator: Did you normally walk with your daughter, or has that been since you started the program?

Interviewee: I used to walk with the kids all the time before my feet got sore. But just lately, since I had that last phone call, I’ve been walking with my daughter, yes.

Facilitator: So more since the phone call?

Interviewee: Yes. I mean I have an operation in May, so I need to try and get everything [0:10:20 inaudible] before I have it.

Facilitator: Sorry?

Interviewee: Because I’ve got to have an operation in May. I’m trying to be a little bit fitter before I actually – because I won’t be able to walk after that for a while.

Facilitator: Yes, it would be hard. Have you thought more about what you’ve been eating?

Interviewee: Well I was going through this horrible stage when I was just eating rubbish. Like comfort food. But lately – yes, I’ve just started the last couple of weeks actually, of really eating – I'm just cooking soup now and yeah, I’m really starting to have – I’m cutting out sugar. That’s what I’m doing. And I have flicked through the book again. So I really want to do it. It’s just hard sometimes.

Facilitator: Yes, just finding barriers. So just to clarify – since you started the program, you have thought more about what you’re eating and how much you’re exercising?

Interviewee: Yes.

Facilitator: Great. Sorry, it’s just cutting out a little bit. Can you hear me?

Interviewee: Yes.

Facilitator: Do you have a home number I could call you on?

Interviewee: I’ve got this mobile.

Facilitator: Yes, can you hear me properly?

Interviewee: Yes.

Facilitator: Okay, perfect. Right, sorry. It just cut out for a second. If we look at the different components of the program, what I'm interested is to find out which parts of the program you found most helpful. So we had the Healthy Lifestyle group session, we had the manual, the SMS’ and the website. You also just recently had your phone call. Was anything particularly helpful or most useful to you?

Interviewee: Well after the seminar, and …

Facilitator: Sorry, I just missed that [0:11:58-0:12:06 high pitched noise, vocals inaudible]. Yep, hear you now. Yes, a slight ring. Should I call you back? Sorry. Do you want me to ring you back?

Interviewee: What is that noise?

Facilitator: I don’t know. Is it from …? I might just ring you back, see if that stops the noise.

Interviewee: It’s stopped now.

Facilitator: Yeah, it’s stopped now. Okay, good. There’s a ringing. I’m not sure where it’s from.

Interviewee: No.

Facilitator: Very strange. So you said you loved the seminar?

Interviewee: Yes, and I got more motivated when I had the personal phone call.

Facilitator: And you motivated when you had the phone call. Yep. Did you find the seminar motivating?

Interviewee: Yes, I did. Yes. The lady was lovely, yes.

Facilitator: What about the SMS’? Did they help you?

Interviewee: Well I read them. But I knew what I had to do, but they didn’t really egg me on or anything. But they should’ve. They should’ve, but I just didn’t …

Facilitator: No, that’s okay. Do you think they didn’t egg you on because – were they too long or they were just too generic? Was there something that we could’ve done to make them a bit more useful?

Interviewee: No. It was more – it was just sort of “don't forget to eat your fruit and veggies” or whatever like that. But I don’t know, really. I suppose what more can you really do on an SMS. It all depends on the individual person. See, I just read it and thought yep, okay. But whether you do it or not is up to you, isn’t it?

Facilitator: Yes, of course. So would you say that it helped motivate you? Or it didn’t?

Interviewee: No, I didn’t get motivated from it.

Facilitator: That’s okay. We’ve had a lot of mixed responses.

Interviewee: But the seminar did.

Facilitator: But the seminar did. Did you use the website?

Interviewee: No, I haven’t actually.

Facilitator: Do you often access the website for health information? The web?

Interviewee: Yes, she did give me some [audio cuts out], which I just haven’t. I should’ve.

Facilitator: No that’s okay. Do you normally use the internet to look for health information?

Interviewee: No, I don’t.

Facilitator: No? Some people just don’t use it.

Interviewee: I usually read the body and soul in the Sunday papers every week.

Facilitator: Yes? That’s how some people – everyone’s got their own way of accessing health information. Now I’m just thinking – so thinking about the whole program – oh sorry. I think that – sorry.

Interviewee: That’s all right.

Facilitator: I think it’s the – I think it’s because I’ve you on speaker. If we think about the different components, if we took out those SMS messages, do you think that would’ve affected your motivation today about your diet and your exercise? If you didn’t receive them?

Interviewee: No, I think I’d leave the SMS’ because – that’s just me, but I think it would motivate other people.

Facilitator: So in the context of where you are with your motivation, the SMS’ haven’t had an impact, is that correct?

Interviewee: Not on me, no. I did read them and I do know that I should’ve been doing it. But I didn’t.

Facilitator: No, that’s okay. No problem.

Interviewee: But I think that’s a good idea, though. You sort of forget. Like first the seminar, then the phone call.

Facilitator: So you think they’re a good idea. Yes.

Interviewee: I do, yes.

Facilitator: But the things that have motivated you the most are the phone coaching and the session, yeah?

Interviewee: Yeah.

Facilitator: Now since – I know that you’ve said you’ve had a bit of a tough time with everything going on and sorry to hear that. But is there any changes that you have been able to sustain since being in the program? You mentioned walking earlier, with your daughter.

Interviewee: Yes, I’ve just started walking more now. Even though it kills. But I know I have to do it. Because there’s not really much I can do with exercise because I have fibromyalgia as well. It’s a vicious circle. It sounds silly, but it’s just really hard to get fit. When I have my foot done, I’m just going to be out of action. So I need to do it now.

Facilitator: How much have you increased your walking by, do you think?

Interviewee: Well I walk every night. I never used to walk in some weeks.

Facilitator: Perfect. So you’re walking daily with your daughter.

Interviewee: Every night with my daughter and our dog.

Facilitator: Perfect. It’s great that she’s helping to motivate you.

Interviewee: She’s only 11. She loves it. We try and jog a bit too, you know.

Facilitator: Has that been – that’s a new thing?

Interviewee: Yes. She never used to come at night. We go just after, like six-thirty or something.

Facilitator: And that’s been since the phone call?

Interviewee: Yes.

Facilitator: Great. Glad to hear that’s been helpful for you. Any other changes you’ve made to what you’ve been eating or what you buy for the family?

Interviewee: I cook healthy for the family but I tend to crave sugar so I’m a bit naughty. But I’m cutting out sugar.

Facilitator: You’re doing that now?

Interviewee: Yes.

Facilitator: Has that been any effect from the program? Or just …?

Interviewee: It has. I have read parts of the book and especially that phone call as well, was a bit more motivation. Yeah.

Facilitator: Good. And the phone call. When you say cutting out more sugar, what are you cutting out?

Interviewee: Well, trying not to eat sugar at all.

Facilitator: So that includes any …?

Interviewee: Any rubbish. I buy potato chips or something for the kids. But I’ve just got to stop eating stuff, so I’ll have porridge for breakfast, maybe a shake or a salad sandwich for lunch and I’m just making homemade soups for my tea, but I’ll cook them whatever they – the meat and vegetables stuff.

Facilitator: So in terms of rubbish, you mean more like snack foods?

Interviewee: Yeah. Yes. And that’s what I crave for. If I go out, instead of opting for an apple, I’ll have a doughnut. It’s just ridiculous.

Facilitator: So it’s things like doughnuts. Anything else? You’re cutting out?

Interviewee: Yeah, chocolate. Sugar on cereal. I’m just trying not to eat as much because it’s just – it takes – and I have. I’ve gotten bigger around the abdomen. I don’t like it. It’s just uncomfortable. I don’t feel comfortable going out.

Facilitator: So your motivation is you’re not feeling comfortable?

Interviewee: Yep. I don’t like going out because – I just don’t feel comfortable. Because nothing seems to fit me like it used to. It sort of brings you down.

Facilitator: Of course.

Interviewee: I need to lose weight. Yes.

Facilitator: What about since being in the program – [Sheree’s] the lady who delivered the session – talked about getting health checks and other nutrition problems in women. Has it motivated you to access any other community health services or join any groups in the community?

Interviewee: No. I’m not one to – big on crowds, really. I don’t think I’ve got that confidence. I think if I lost a lot of weight, I’d probably have more confidence to do something like that.

Facilitator: So do you think that even coming to the session was a hurdle for you to come to the session in a crowd?

Interviewee: Yes. Well I thought there’d be a lot of people there. I get a bit nervous. But I wasn’t the biggest one there, so you know what I mean? Yeah.

Facilitator: When you say nervous - sorry, I ‘m just interested because we’ve had – I think what you’re saying is quite a consistent thing that we’re finding, and it’s interesting what you’re saying. So it’s harder to come in a smaller community – would you say it’s hard to come to a Healthy Lifestyle session if you’re feeling a bit self-conscious?

Interviewee: I think any sort of session, if you’re feeling self-conscious. Yeah, I don’t want to go out as much since I’ve put on weight. But if – I think you get more confidence when you lose weight.

Facilitator: Yes, you definitely – I think you definitely do. So that’s great. So you mentioned that you’ve been walking more and you’ve been cutting out that rubbish. Have you been using any of the strategies that were talked about in the session and were, again, in the manual? Like problem solving, goal setting. Have you used any of those strategies to help you do that?

Interviewee: I'm not sure.

Facilitator: So when you’ve been exercising more, can you think of any ways that you’ve problem solved?

Interviewee: No, I don’t think so. I don’t know.

Facilitator: What about the action plans? Did you use the action plans? So when the girl rang you a couple of weeks ago about your health goals, do you remember using an action plan? So what your goal was, how you’re going to achieve it, what the barriers were?

Interviewee: Oh yep. To drink more water, a goal was. I even actually put a note on the pantry. I was. I was. And I’ve cut out soft drink. But I was drinking more water. But I don’t drink enough. I’m still not drinking enough.

Facilitator: But are you doing that sort of thinking when you’re going for your walk? Are you thinking about barriers that could stop you from walking and making up solutions?

Interviewee: No. I just think “we need to do our walk”. I just need to do it.

Facilitator: Good. Sounds like you’re pretty motivated. That’s great. Now what I’m also interested in, and you’ve already touched on this – so the point of the program is to help improve your health and get you to be more active and to start thinking about your lifestyle. But we’re interested in seeing if the program’s – the messages and what we talked about in the program – reached anybody else. So you mentioned that you’ve been walking more with your daughter. Have you talked more to your daughter since being in the program about health and about your weight?

Interviewee: Yes. She’s a sweet tooth, this one.

Facilitator: So you’ve been talking about it with her?

Interviewee: Yes, I have. So she’s – when we get home, she’ll offer an apple instead of something – she’s always looking for something sweet.

Facilitator: So do you think, by you being in the program, you’ve influenced her behaviour?

Interviewee: Yes, I have. Yep.

Facilitator: So together you’re talking about ways to eat healthier? Is that what you said about the apple?

Interviewee: Yep. Because she’s not a big girl. She’s only tiny. But kids like their sweet stuff and I'm not a very good role model when I was eating all that. So I had fruit. I buy a lot of fruit.

Facilitator: Good. So you’re buying more fruit? Yep. Any other examples of how you’ve influenced your daughter?

Interviewee: I always say – like I don’t say horrible things – I’ll just sort of have said that if you eat a lot of shit now – excuse the language – when you get older, you will start to get bigger. It’s because of what you’ve eaten now. They do play sport, my daughters. I’d just like them to be active, you know? I’d say you need to be active now.

Facilitator: So would you say that by you being more active, this has effected your family?

Interviewee: I think so. The girls are pretty active. Tyson is built like a stick insect, so he doesn’t really need to do much. But he’ll just skateboard. But the girls go jogging occasionally together or they’ll go for a walk or they’ll go and play basketball up at the school.

Facilitator: You said one of them is walking with you as well.

Interviewee: Yes. My oldest daughter can’t because she has a lot of homework after school. But yeah, we do that. Yeah. [Overtalking] just that one-on-one. Yeah, it’s nice.

Facilitator: That’s been since you started this program?

Interviewee: Yes. Not every night. It was – sometimes it wasn’t for weeks. But I’ve gotten back into now.

Facilitator: Are there any other – since you said you’ve been cutting out the junk food, has anyone else been affected by this? Have you been buying less junk food for the house or has this affected anyone else in the household?

Interviewee: Yeah, I buy less. Sometimes I do ask for something to eat after tea, but I always say “there’s fruit there”. Because I haven’t bought any rubbish. I might get the odd backchat off my son but he’ll go and find some frozen berries or something and he’s happy then.

Facilitator: So inadvertently, you have influenced your children.

Interviewee: Yeah. That’s what I want them doing. I don’t want to – it’s just unhealthy lifestyle I was living and I don’t want to pass that on to my kids.

Facilitator: So would you agree with me when I say you’re trying to role model your eating behaviours for your children?

Interviewee: And me too, yes. I want them to be healthy. I don’t want them to be obese or have to worry about their weight. I don’t want them to feel like I do. Yeah.

Facilitator: So you’ve been talking more about health and changing things in the household?

Interviewee: Yes.

Facilitator: What about – in terms of, again, that sharing health information and talking about your health, do you talk about your health much with your friends?

Interviewee: They always ask about me and what’s going on with my health problems. But I don’t really bring it up.

Facilitator: Did any of your friends – you said you came with a few of your friends to the program. Have you talked much about the program since? With them? About what changes you’ve made to your lifestyle?

Interviewee: One of my friends who did it – she’s lost over 20 kilos since she joined it.

Facilitator: That’s amazing. That’s fantastic.

Interviewee: I know. I know. She’s motivated me a bit more as well, because of that. Because she was bigger than me.

Facilitator: She’s lost 20 kilos? What has she done, do you think, that’s – or what do you think motivating her? What do you think?

Interviewee: All she’s done is cut out sugar and walked. That’s it.

Facilitator: That’s amazing.

Interviewee: She’s got fantastic willpower, I can say that. Only got one child though, but yeah, so that’s amazing.

Facilitator: Yeah, that is fantastic. It’s great. And it’s great that she’s made such a leap.

Interviewee: I know. I wish I could do that. I think “oh my god!” It’s the willpower. I need that willpower. That’s what I need.

Facilitator: You need the willpower? What do you think would help – is there anything that we could do or what could help give you that willpower, do you think?

Interviewee: I don’t know. I just think walking with my daughter every night will give me more willpower. It was raining last night. I thought she said “oh no, we won’t go”. Then it eased off and I said “no, let’s go”. So we went. Yeah.

Facilitator: So your daughter – so social supports, perhaps?

Interviewee: And she wanted to go too. So we need to just do it whether it’s raining – rain hail or shine, we just have to go.

Facilitator: That’s a good attitude. Did you have – do you think that – sorry to keep going on about this. But do you think that you’ve then influenced any – you’ve mentioned that you’ve influenced your family, which is fantastic. Do you think you’ve influenced any of your friends or anyone in your social network to be a bit healthier since joining the program?

Interviewee: No, I don’t think I have, my friends. I don’t go out with them that often. I’ll probably go out once a week and pop in and see them, but no, not really.

Facilitator: It’s good to honest. Some people think they should – they say things because they think that’s what I want them to say, and I want you to be honest. Because the whole point of this evaluation is to get your opinion, not my opinion. So the last questions I want to ask you is just about your satisfaction with the program. Again, be as honest as you can. So if we were going to come back to Wonthaggi, based on your experience, would you recommend the program to anybody else in your town?

Interviewee: Yeah, I would actually, yes.

Facilitator: Why would you recommend it?

Interviewee: Well there’s nothing like that around here. They need more public awareness for women’s health. There’s really not much, unless there are seminars. So I do, yes.

Facilitator: Are there many seminars? Is that common?

Interviewee: I’ve never seen one. No. I mean, I’ve tried diet after diet and I even joined Jenny Craig and all this. I did lose five kilos on that, but then I got into a rut because it was only for three months. I did put on weight. After I finished. So that’s my fault. But there’s nothing, unless you join a weight loss centre.

Facilitator: Are there many weight loss centres in the community?

Interviewee: No. I had to go to Cranbourne. I think there’s Weight Watchers here. But I’m not sure where it is. Because you don’t really see that advertised.

Facilitator: In terms of the facilitator, you said that you really like Sheree. But attending a program like that, do you have a preference for whether the lady running the program is from your town or is from Melbourne?

Interviewee: Oh no, it doesn’t bother me.

Facilitator: No preference?

Interviewee: As long as she interacts with the women and is just a lovely, fun loving – just down to earth person. That struggles as well, maybe. You know what I mean?

Facilitator: So it’s important that she understands?

Interviewee: Yeah, I think so. Yeah.

Facilitator: When you say struggle as well, as in she’s faced …?

Interviewee: Well, she said she’d had problems with eating and – I mean, most women do. It’s just … I think everyone does whether you’re a bloke or a woman.

Facilitator: What about the venue? Do you think that was convenient? Or do you think that …?

Interviewee: I thought it was. I found it convenient because I was up there. I go there with my daughter, so I thought it was convenient. So did the other mothers that were there, obviously. But it was just a shame that there wasn’t many more.

Facilitator: But there’s no factor – sorry, but there’s no factors that stand out to you as to why other women didn’t attend?

Interviewee: No, I don’t know. Like I said, some mothers just drop the kids off and bang, they’re gone for coffee or something. Yeah, you see, I’m usually up the school.

Facilitator: What would you prefer? You did mention before that you sometimes felt a bit self-conscious in a group. But would you prefer a group based session or an individual?

Interviewee: I think group. Once you sort of get involved in the group, you can all have a bit of a laugh and everyone knows exactly what everyone’s going through …

Facilitator: So that’s, again, that community.

Interviewee: … I think it’s best in groups. But I think it was – you start to relax later on into it. Because everyone’s there for the same reason. One-on-one would probably be a bit too confronting, I reckon.

Facilitator: When you say confronting, what do you mean exactly by that?

Interviewee: I think it’d probably be more nervous one-on-one because – yeah, I don’t know. You’d feel a bit …

Facilitator: Vulnerable maybe?

Interviewee: Pardon?

Facilitator: Like maybe a bit vulnerable?

Interviewee: Yeah, yeah. I don’t think I’d feel comfortable one-on-one. I wasn’t comfortable with a group, but you sort of – because I was with some friends as well, you get to – everyone’s there for the same reason. So you can have a bit of a laugh and everyone knows exactly why we’re here.

Facilitator: So you said you were uncomfortable initially but then you felt better?

Interviewee: Yeah. The lady who was doing the seminar – she’s comfortable too. I think you sort of get more involved. Yeah.

Facilitator: Great. The last question I have for you is if we were going to run this program again, what could we do to make you more motivated? Is there anything you could think of that would help you stay on track and more motivated?

Interviewee: I don’t know.

Facilitator: How could the program be improved?

Interviewee: Maybe that phone call I had. Maybe more of those. Maybe like one-on-one phone call. I thought that was quite interesting. It got me more motivated. Yeah. I don’t really know, really.

Facilitator: Would you like more contact or more sessions? No?

Interviewee: Probably that phone contact was good. Because you probably only need the one seminar and just the phone contact.

Facilitator: And how many? When you say phone contact, once a month? Once every few months? How many, roughly?

Interviewee: Well we’ve only had the one, haven’t we? So …

Facilitator: Yes, but if we were going to run it again, how many would you like to have?

Interviewee: Well I don’t know.

Facilitator: A couple?

Interviewee: Yeah, I reckon. Two or three throughout the year or something like that, I reckon. To keep the motivation going or something. Yeah.

Facilitator: Great. Anything else that you want to add about how we could improve the program?

Interviewee: No, I don’t think so.

Facilitator: Anything else that you think would be interesting for us to capture as part of evaluating the program? About the community that the town you live in?

Interviewee: I can’t think of anything. Not off the top of my head, anyway. No, I don’t know.

Facilitator: That’s no problem. Thank you so much for your time. We really, really appreciate you participating in this interview. It really gives us some valuable feedback that is really important to so that we can just continue improving the program. I’ll send you that gift card in the mail today. So if you don’t get it in the next week, please give us a call back and we’ll make sure that gets sent to you. Thank you so much again.

Interviewee: No worries. Thanks very much for that.

Facilitator: Thank you. Nice to speak to you. Thanks for that.

Interviewee: Thank you. Bye.

Facilitator: Bye.

END OF TRANSCRIPT

Participant #NN

full transcript

facilitator: so as I mentioned to you, the point of the evaluation is to get your feedback about the program. But the first thing that I wanted to start off with is for you just to describe your community in Wonthaggi to me. So in terms of the size, the age of the community. Anything you think that will be beneficial for us to know.

Interviewee: It’s not overly large. I think it’s – I’m not sure how many people live here. I really don’t know how many people live here. I think there’d be …

Facilitator: But would you consider it a large, a small …? What would consider the town in terms of size?

Interviewee: Probably a small town. But I’ve seen smaller. A lot more young people – well, when the desal was here, a lot more young people moved here.

Facilitator: So a lot of young people there now?

Interviewee: Mainly a lot of a – there was a lot of elderly people. But there are a lot of – they seem to get sent down here for some reason. I don’t know why.

Facilitator: Young people?

Interviewee: Hmmm.

Facilitator: When you say young, do you mean people with families or like more …?

Interviewee: Young teenagers and young parents, yeah. They seem to get sent here. But there’s no employment down this way.

Facilitator: No employment.

Interviewee: No.

Facilitator: So if you were looking for a job, what kind of areas could you find work in?

Interviewee: Well, the did get a lot of people in the new parts when they built the new plaza before [0:01:41 unclear]. But there’s really not much unless – school kids could probably get a job, but it’s really hard for adults.

Facilitator: Do you feel that it’s a health conscious town? Do many people talk about health or there are common health problems that you think – in the town?

Interviewee: No, I don’t think so. Just the usual, what you get, I suppose. The usual ailments that people get. But no, I don’t think so. No.

Facilitator: Would you consider the town to be active? Physically active?

Interviewee: There’s a lot of people that go around – there’s a lot of walking tracks. So there’s a lot of people – and there’s a couple of gyms.

Facilitator: And gyms. And a lot of people are walking?

Interviewee: Oh yes, there’s a lot that jog and walk and, yeah.

Facilitator: Do most of them walk together or more people by themselves or is there walking groups?

Interviewee: I’ve seen elderly walking groups. You know, elderly groups. But a lot of people just go couples or usually on their own or within families. Yeah.

Facilitator: What about access to health services? Would you think that there is adequate health services?

Interviewee: I think we’ve got a health centre for young mums. There’s two doctors’ clinics. But there’s not much for, you know, children with disabilities. There’s a disability school. But it’s nothing to do with – oh, I don’t know. There’s not much up – because it’s a small country town. It’s not like down Melbourne. The kids are [0:01:41 unclear]. There’s not really that up here, unless there’s a seminar.

Facilitator: Any comments on – in terms of obesity, do you think obesity’s a problem for the town?

Interviewee: No. Look, there’s really not that – well, I don’t really take notice of people really. No, I haven’t really seen many overly large people. I mean, you see them everywhere but I don’t think there’s a common problem here.

Facilitator: Do you hear people talking about their health much or about their weight? Is there much talk amongst women about their health?

Interviewee: Well there is with me and my friends. But that’s just more my thing. I think most people talk about themselves, they’re just not happy with something are they?

Facilitator: Yes, no. No-one’s usually very happy with their body. So if you think back to why you decided to join the program, are there any factors that motivated you specifically to join this Healthy Lifestyle program?

Interviewee: Yeah, well I really wanted to get fit and lose some weight. But it didn’t happen because during it I lost my mum and – which sort of I tend to eat a lot for a comfort sort of thing. That’s how I deal with stress. Now I’m waiting for a foot operation, so I really can’t walk so I'm not very active at the moment, so I’ve actually put on weight. Just due to me, nothing else though.

Facilitator: Just due to the circumstances.

Interviewee: Yeah.

Facilitator: Was there anything else that motivated you to come to the program? Did you have friends going that encouraged you to come or anything else?

Interviewee: Yeah, there was a couple of my friends were going and I thought “oh, we’ll give it a try and see what it was like”.

Facilitator: Do you find it easier to go to a program if you’ve got friends going? Or easier to go by yourself?

Interviewee: I’d rather go with friends. But I have been to things on my own.

Facilitator: Now thinking back to the session in Wonthaggi, there was only a small group of women. Can you think of – as you’re living in the town, any reasons that may have stopped other women from attending?

Interviewee: Well I don’t know. Because I think you’d rally up the school. That’s how I joined. I’m not sure if you were anywhere else.

Facilitator: Yep, we did.

Interviewee: But I don’t really know.

Facilitator: Timing of the session? Do you think that could’ve been a problem?

Interviewee: Because at that time when I walked through the gates they might not have been there. I’m not sure.

Facilitator: What about timing of the session? Do you think that may have been a problem? It was during the day.

Interviewee: Well a lot of mums work. Well I found it right, because I wasn’t thinking at the time, but a night session might have been good. Like say – not late, but like a town hall session or something like a club.

Facilitator: What about the venue? Do you think that made a difference to whether people would come or not? Because it was at the school?

Interviewee: It might have put off some. I’m not sure. But that’s where my daughter goes.

Facilitator: Why do you think it could’ve put off some?

Interviewee: Pardon?

Facilitator: How come you think it may have put off some?

Interviewee: Well a lot of parents don't really go to the school, you know? Like they just drop their kids off and that’s it. They don’t put in. Whereas I’ve always been a the school with my kids. I'm always there helping, but some parents just drop in and say – you know what I mean? They might just drop them at the gates.

Facilitator: Yeah, don’t usually go there. Yep. And seasonal factors? Anything that would’ve interrupted in October? Anything? Bigger community events or anything going on?

Interviewee: In October?

Facilitator: Yeah. That’s when the session was last year. Any stand out?

Interviewee: I don’t think there was anything happening in October. Only the motor cycle grand prix thing. That’s about it.

Facilitator: But nothing else. No. We found that we came around Christmas, around harvest time in some of the other towns, and that was a bit of a problem. But I don’t think that would’ve been a problem in your town.

Interviewee: No.

Facilitator: Now as you – sorry. As part of the program, you attended that group session in Wonthaggi. We had the phone call that you got a couple of weeks ago. We gave you the manual and the SMS’. When you think about the program, is there something that stands out to you in terms of what you learnt in the program? Like what information you learnt?

Interviewee: Oh, I don’t know.

Facilitator: So if I was going to ask you – if you were going to talk to someone, a friend, about the program, how would you describe the program? So if you were going to start with “the program was about …”?

Interviewee: Oh, I see, yes, yes. I found it quite interesting actually. The little book. I didn’t read it all when I had the phone call. The phone call was obviously a bit more of a motivator.

Facilitator: But what did you – if you were going to describe the program, what information would you say you learnt about? Or what information was refreshed for you?

Interviewee: I don’t know. I liked – what was the girl’s name? The lady that was doing all the talking. I found her quite interesting to listen to because I liked – she was quite – she was on our path. She’d been there, done that. I’m like one-on-one, like when someone’s actually doing a speech. Yes.

Facilitator: But is there anything that stood out to you? So any key messages about weight or about food? Was there anything that stood out to you, if you could summarise something that you’d learnt?

Interviewee: I don’t know. There probably was, but I’m – I haven’t really done it properly, which is what’s happened.

Facilitator: No, that’s completely understandable. Okay, no problem. What about – since being in the program, have you started thinking a little bit more about what you’re eating? About your exercise and about your weight more? Or would you say it’s been the same?

Interviewee: Oh no, definitely more. I’ve been thinking more about it. But I’m just finding it hard to do it, you know what I mean? Like there’s so much gone – I’ve got to have – I can’t really walk, but I’ve been walking with one of my daughters every night. But the pain in excruciating.

Facilitator: Did you normally walk with your daughter, or has that been since you started the program?

Interviewee: I used to walk with the kids all the time before my feet got sore. But just lately, since I had that last phone call, I’ve been walking with my daughter, yes.

Facilitator: So more since the phone call?

Interviewee: Yes. I mean I have an operation in May, so I need to try and get everything [0:10:20 inaudible] before I have it.

Facilitator: Sorry?

Interviewee: Because I’ve got to have an operation in May. I’m trying to be a little bit fitter before I actually – because I won’t be able to walk after that for a while.

Facilitator: Yes, it would be hard. Have you thought more about what you’ve been eating?

Interviewee: Well I was going through this horrible stage when I was just eating rubbish. Like comfort food. But lately – yes, I’ve just started the last couple of weeks actually, of really eating – I'm just cooking soup now and yeah, I’m really starting to have – I’m cutting out sugar. That’s what I’m doing. And I have flicked through the book again. So I really want to do it. It’s just hard sometimes.

Facilitator: Yes, just finding barriers. So just to clarify – since you started the program, you have thought more about what you’re eating and how much you’re exercising?

Interviewee: Yes.

Facilitator: Great. Sorry, it’s just cutting out a little bit. Can you hear me?

Interviewee: Yes.

Facilitator: Do you have a home number I could call you on?

Interviewee: I’ve got this mobile.

Facilitator: Yes, can you hear me properly?

Interviewee: Yes.

Facilitator: Okay, perfect. Right, sorry. It just cut out for a second. If we look at the different components of the program, what I'm interested is to find out which parts of the program you found most helpful. So we had the Healthy Lifestyle group session, we had the manual, the SMS’ and the website. You also just recently had your phone call. Was anything particularly helpful or most useful to you?

Interviewee: Well after the seminar, and …

Facilitator: Sorry, I just missed that [0:11:58-0:12:06 high pitched noise, vocals inaudible]. Yep, hear you now. Yes, a slight ring. Should I call you back? Sorry. Do you want me to ring you back?

Interviewee: What is that noise?

Facilitator: I don’t know. Is it from …? I might just ring you back, see if that stops the noise.

Interviewee: It’s stopped now.

Facilitator: Yeah, it’s stopped now. Okay, good. There’s a ringing. I’m not sure where it’s from.

Interviewee: No.

Facilitator: Very strange. So you said you loved the seminar?

Interviewee: Yes, and I got more motivated when I had the personal phone call.

Facilitator: And you motivated when you had the phone call. Yep. Did you find the seminar motivating?

Interviewee: Yes, I did. Yes. The lady was lovely, yes.

Facilitator: What about the SMS’? Did they help you?

Interviewee: Well I read them. But I knew what I had to do, but they didn’t really egg me on or anything. But they should’ve. They should’ve, but I just didn’t …

Facilitator: No, that’s okay. Do you think they didn’t egg you on because – were they too long or they were just too generic? Was there something that we could’ve done to make them a bit more useful?

Interviewee: No. It was more – it was just sort of “don't forget to eat your fruit and veggies” or whatever like that. But I don’t know, really. I suppose what more can you really do on an SMS. It all depends on the individual person. See, I just read it and thought yep, okay. But whether you do it or not is up to you, isn’t it?

Facilitator: Yes, of course. So would you say that it helped motivate you? Or it didn’t?

Interviewee: No, I didn’t get motivated from it.

Facilitator: That’s okay. We’ve had a lot of mixed responses.

Interviewee: But the seminar did.

Facilitator: But the seminar did. Did you use the website?

Interviewee: No, I haven’t actually.

Facilitator: Do you often access the website for health information? The web?

Interviewee: Yes, she did give me some [audio cuts out], which I just haven’t. I should’ve.

Facilitator: No that’s okay. Do you normally use the internet to look for health information?

Interviewee: No, I don’t.

Facilitator: No? Some people just don’t use it.

Interviewee: I usually read the body and soul in the Sunday papers every week.

Facilitator: Yes? That’s how some people – everyone’s got their own way of accessing health information. Now I’m just thinking – so thinking about the whole program – oh sorry. I think that – sorry.

Interviewee: That’s all right.

Facilitator: I think it’s the – I think it’s because I’ve you on speaker. If we think about the different components, if we took out those SMS messages, do you think that would’ve affected your motivation today about your diet and your exercise? If you didn’t receive them?

Interviewee: No, I think I’d leave the SMS’ because – that’s just me, but I think it would motivate other people.

Facilitator: So in the context of where you are with your motivation, the SMS’ haven’t had an impact, is that correct?

Interviewee: Not on me, no. I did read them and I do know that I should’ve been doing it. But I didn’t.

Facilitator: No, that’s okay. No problem.

Interviewee: But I think that’s a good idea, though. You sort of forget. Like first the seminar, then the phone call.

Facilitator: So you think they’re a good idea. Yes.

Interviewee: I do, yes.

Facilitator: But the things that have motivated you the most are the phone coaching and the session, yeah?

Interviewee: Yeah.

Facilitator: Now since – I know that you’ve said you’ve had a bit of a tough time with everything going on and sorry to hear that. But is there any changes that you have been able to sustain since being in the program? You mentioned walking earlier, with your daughter.

Interviewee: Yes, I’ve just started walking more now. Even though it kills. But I know I have to do it. Because there’s not really much I can do with exercise because I have fibromyalgia as well. It’s a vicious circle. It sounds silly, but it’s just really hard to get fit. When I have my foot done, I’m just going to be out of action. So I need to do it now.

Facilitator: How much have you increased your walking by, do you think?

Interviewee: Well I walk every night. I never used to walk in some weeks.

Facilitator: Perfect. So you’re walking daily with your daughter.

Interviewee: Every night with my daughter and our dog.

Facilitator: Perfect. It’s great that she’s helping to motivate you.

Interviewee: She’s only 11. She loves it. We try and jog a bit too, you know.

Facilitator: Has that been – that’s a new thing?

Interviewee: Yes. She never used to come at night. We go just after, like six-thirty or something.

Facilitator: And that’s been since the phone call?

Interviewee: Yes.

Facilitator: Great. Glad to hear that’s been helpful for you. Any other changes you’ve made to what you’ve been eating or what you buy for the family?

Interviewee: I cook healthy for the family but I tend to crave sugar so I’m a bit naughty. But I’m cutting out sugar.

Facilitator: You’re doing that now?

Interviewee: Yes.

Facilitator: Has that been any effect from the program? Or just …?

Interviewee: It has. I have read parts of the book and especially that phone call as well, was a bit more motivation. Yeah.

Facilitator: Good. And the phone call. When you say cutting out more sugar, what are you cutting out?

Interviewee: Well, trying not to eat sugar at all.

Facilitator: So that includes any …?

Interviewee: Any rubbish. I buy potato chips or something for the kids. But I’ve just got to stop eating stuff, so I’ll have porridge for breakfast, maybe a shake or a salad sandwich for lunch and I’m just making homemade soups for my tea, but I’ll cook them whatever they – the meat and vegetables stuff.

Facilitator: So in terms of rubbish, you mean more like snack foods?

Interviewee: Yeah. Yes. And that’s what I crave for. If I go out, instead of opting for an apple, I’ll have a doughnut. It’s just ridiculous.

Facilitator: So it’s things like doughnuts. Anything else? You’re cutting out?

Interviewee: Yeah, chocolate. Sugar on cereal. I’m just trying not to eat as much because it’s just – it takes – and I have. I’ve gotten bigger around the abdomen. I don’t like it. It’s just uncomfortable. I don’t feel comfortable going out.

Facilitator: So your motivation is you’re not feeling comfortable?

Interviewee: Yep. I don’t like going out because – I just don’t feel comfortable. Because nothing seems to fit me like it used to. It sort of brings you down.

Facilitator: Of course.

Interviewee: I need to lose weight. Yes.

Facilitator: What about since being in the program – [Sheree’s] the lady who delivered the session – talked about getting health checks and other nutrition problems in women. Has it motivated you to access any other community health services or join any groups in the community?

Interviewee: No. I’m not one to – big on crowds, really. I don’t think I’ve got that confidence. I think if I lost a lot of weight, I’d probably have more confidence to do something like that.

Facilitator: So do you think that even coming to the session was a hurdle for you to come to the session in a crowd?

Interviewee: Yes. Well I thought there’d be a lot of people there. I get a bit nervous. But I wasn’t the biggest one there, so you know what I mean? Yeah.

Facilitator: When you say nervous - sorry, I ‘m just interested because we’ve had – I think what you’re saying is quite a consistent thing that we’re finding, and it’s interesting what you’re saying. So it’s harder to come in a smaller community – would you say it’s hard to come to a Healthy Lifestyle session if you’re feeling a bit self-conscious?

Interviewee: I think any sort of session, if you’re feeling self-conscious. Yeah, I don’t want to go out as much since I’ve put on weight. But if – I think you get more confidence when you lose weight.

Facilitator: Yes, you definitely – I think you definitely do. So that’s great. So you mentioned that you’ve been walking more and you’ve been cutting out that rubbish. Have you been using any of the strategies that were talked about in the session and were, again, in the manual? Like problem solving, goal setting. Have you used any of those strategies to help you do that?

Interviewee: I'm not sure.

Facilitator: So when you’ve been exercising more, can you think of any ways that you’ve problem solved?

Interviewee: No, I don’t think so. I don’t know.

Facilitator: What about the action plans? Did you use the action plans? So when the girl rang you a couple of weeks ago about your health goals, do you remember using an action plan? So what your goal was, how you’re going to achieve it, what the barriers were?

Interviewee: Oh yep. To drink more water, a goal was. I even actually put a note on the pantry. I was. I was. And I’ve cut out soft drink. But I was drinking more water. But I don’t drink enough. I’m still not drinking enough.

Facilitator: But are you doing that sort of thinking when you’re going for your walk? Are you thinking about barriers that could stop you from walking and making up solutions?

Interviewee: No. I just think “we need to do our walk”. I just need to do it.

Facilitator: Good. Sounds like you’re pretty motivated. That’s great. Now what I’m also interested in, and you’ve already touched on this – so the point of the program is to help improve your health and get you to be more active and to start thinking about your lifestyle. But we’re interested in seeing if the program’s – the messages and what we talked about in the program – reached anybody else. So you mentioned that you’ve been walking more with your daughter. Have you talked more to your daughter since being in the program about health and about your weight?

Interviewee: Yes. She’s a sweet tooth, this one.

Facilitator: So you’ve been talking about it with her?

Interviewee: Yes, I have. So she’s – when we get home, she’ll offer an apple instead of something – she’s always looking for something sweet.

Facilitator: So do you think, by you being in the program, you’ve influenced her behaviour?

Interviewee: Yes, I have. Yep.

Facilitator: So together you’re talking about ways to eat healthier? Is that what you said about the apple?

Interviewee: Yep. Because she’s not a big girl. She’s only tiny. But kids like their sweet stuff and I'm not a very good role model when I was eating all that. So I had fruit. I buy a lot of fruit.

Facilitator: Good. So you’re buying more fruit? Yep. Any other examples of how you’ve influenced your daughter?

Interviewee: I always say – like I don’t say horrible things – I’ll just sort of have said that if you eat a lot of shit now – excuse the language – when you get older, you will start to get bigger. It’s because of what you’ve eaten now. They do play sport, my daughters. I’d just like them to be active, you know? I’d say you need to be active now.

Facilitator: So would you say that by you being more active, this has effected your family?

Interviewee: I think so. The girls are pretty active. Tyson is built like a stick insect, so he doesn’t really need to do much. But he’ll just skateboard. But the girls go jogging occasionally together or they’ll go for a walk or they’ll go and play basketball up at the school.

Facilitator: You said one of them is walking with you as well.

Interviewee: Yes. My oldest daughter can’t because she has a lot of homework after school. But yeah, we do that. Yeah. [Overtalking] just that one-on-one. Yeah, it’s nice.

Facilitator: That’s been since you started this program?

Interviewee: Yes. Not every night. It was – sometimes it wasn’t for weeks. But I’ve gotten back into now.

Facilitator: Are there any other – since you said you’ve been cutting out the junk food, has anyone else been affected by this? Have you been buying less junk food for the house or has this affected anyone else in the household?

Interviewee: Yeah, I buy less. Sometimes I do ask for something to eat after tea, but I always say “there’s fruit there”. Because I haven’t bought any rubbish. I might get the odd backchat off my son but he’ll go and find some frozen berries or something and he’s happy then.

Facilitator: So inadvertently, you have influenced your children.

Interviewee: Yeah. That’s what I want them doing. I don’t want to – it’s just unhealthy lifestyle I was living and I don’t want to pass that on to my kids.

Facilitator: So would you agree with me when I say you’re trying to role model your eating behaviours for your children?

Interviewee: And me too, yes. I want them to be healthy. I don’t want them to be obese or have to worry about their weight. I don’t want them to feel like I do. Yeah.

Facilitator: So you’ve been talking more about health and changing things in the household?

Interviewee: Yes.

Facilitator: What about – in terms of, again, that sharing health information and talking about your health, do you talk about your health much with your friends?

Interviewee: They always ask about me and what’s going on with my health problems. But I don’t really bring it up.

Facilitator: Did any of your friends – you said you came with a few of your friends to the program. Have you talked much about the program since? With them? About what changes you’ve made to your lifestyle?

Interviewee: One of my friends who did it – she’s lost over 20 kilos since she joined it.

Facilitator: That’s amazing. That’s fantastic.

Interviewee: I know. I know. She’s motivated me a bit more as well, because of that. Because she was bigger than me.

Facilitator: She’s lost 20 kilos? What has she done, do you think, that’s – or what do you think motivating her? What do you think?

Interviewee: All she’s done is cut out sugar and walked. That’s it.

Facilitator: That’s amazing.

Interviewee: She’s got fantastic willpower, I can say that. Only got one child though, but yeah, so that’s amazing.

Facilitator: Yeah, that is fantastic. It’s great. And it’s great that she’s made such a leap.

Interviewee: I know. I wish I could do that. I think “oh my god!” It’s the willpower. I need that willpower. That’s what I need.

Facilitator: You need the willpower? What do you think would help – is there anything that we could do or what could help give you that willpower, do you think?

Interviewee: I don’t know. I just think walking with my daughter every night will give me more willpower. It was raining last night. I thought she said “oh no, we won’t go”. Then it eased off and I said “no, let’s go”. So we went. Yeah.

Facilitator: So your daughter – so social supports, perhaps?

Interviewee: And she wanted to go too. So we need to just do it whether it’s raining – rain hail or shine, we just have to go.

Facilitator: That’s a good attitude. Did you have – do you think that – sorry to keep going on about this. But do you think that you’ve then influenced any – you’ve mentioned that you’ve influenced your family, which is fantastic. Do you think you’ve influenced any of your friends or anyone in your social network to be a bit healthier since joining the program?

Interviewee: No, I don’t think I have, my friends. I don’t go out with them that often. I’ll probably go out once a week and pop in and see them, but no, not really.

Facilitator: It’s good to honest. Some people think they should – they say things because they think that’s what I want them to say, and I want you to be honest. Because the whole point of this evaluation is to get your opinion, not my opinion. So the last questions I want to ask you is just about your satisfaction with the program. Again, be as honest as you can. So if we were going to come back to Wonthaggi, based on your experience, would you recommend the program to anybody else in your town?

Interviewee: Yeah, I would actually, yes.

Facilitator: Why would you recommend it?

Interviewee: Well there’s nothing like that around here. They need more public awareness for women’s health. There’s really not much, unless there are seminars. So I do, yes.

Facilitator: Are there many seminars? Is that common?

Interviewee: I’ve never seen one. No. I mean, I’ve tried diet after diet and I even joined Jenny Craig and all this. I did lose five kilos on that, but then I got into a rut because it was only for three months. I did put on weight. After I finished. So that’s my fault. But there’s nothing, unless you join a weight loss centre.

Facilitator: Are there many weight loss centres in the community?

Interviewee: No. I had to go to Cranbourne. I think there’s Weight Watchers here. But I’m not sure where it is. Because you don’t really see that advertised.

Facilitator: In terms of the facilitator, you said that you really like Sheree. But attending a program like that, do you have a preference for whether the lady running the program is from your town or is from Melbourne?

Interviewee: Oh no, it doesn’t bother me.

Facilitator: No preference?

Interviewee: As long as she interacts with the women and is just a lovely, fun loving – just down to earth person. That struggles as well, maybe. You know what I mean?

Facilitator: So it’s important that she understands?

Interviewee: Yeah, I think so. Yeah.

Facilitator: When you say struggle as well, as in she’s faced …?

Interviewee: Well, she said she’d had problems with eating and – I mean, most women do. It’s just … I think everyone does whether you’re a bloke or a woman.

Facilitator: What about the venue? Do you think that was convenient? Or do you think that …?

Interviewee: I thought it was. I found it convenient because I was up there. I go there with my daughter, so I thought it was convenient. So did the other mothers that were there, obviously. But it was just a shame that there wasn’t many more.

Facilitator: But there’s no factor – sorry, but there’s no factors that stand out to you as to why other women didn’t attend?

Interviewee: No, I don’t know. Like I said, some mothers just drop the kids off and bang, they’re gone for coffee or something. Yeah, you see, I’m usually up the school.

Facilitator: What would you prefer? You did mention before that you sometimes felt a bit self-conscious in a group. But would you prefer a group based session or an individual?

Interviewee: I think group. Once you sort of get involved in the group, you can all have a bit of a laugh and everyone knows exactly what everyone’s going through …

Facilitator: So that’s, again, that community.

Interviewee: … I think it’s best in groups. But I think it was – you start to relax later on into it. Because everyone’s there for the same reason. One-on-one would probably be a bit too confronting, I reckon.

Facilitator: When you say confronting, what do you mean exactly by that?

Interviewee: I think it’d probably be more nervous one-on-one because – yeah, I don’t know. You’d feel a bit …

Facilitator: Vulnerable maybe?

Interviewee: Pardon?

Facilitator: Like maybe a bit vulnerable?

Interviewee: Yeah, yeah. I don’t think I’d feel comfortable one-on-one. I wasn’t comfortable with a group, but you sort of – because I was with some friends as well, you get to – everyone’s there for the same reason. So you can have a bit of a laugh and everyone knows exactly why we’re here.

Facilitator: So you said you were uncomfortable initially but then you felt better?

Interviewee: Yeah. The lady who was doing the seminar – she’s comfortable too. I think you sort of get more involved. Yeah.

Facilitator: Great. The last question I have for you is if we were going to run this program again, what could we do to make you more motivated? Is there anything you could think of that would help you stay on track and more motivated?

Interviewee: I don’t know.

Facilitator: How could the program be improved?

Interviewee: Maybe that phone call I had. Maybe more of those. Maybe like one-on-one phone call. I thought that was quite interesting. It got me more motivated. Yeah. I don’t really know, really.

Facilitator: Would you like more contact or more sessions? No?

Interviewee: Probably that phone contact was good. Because you probably only need the one seminar and just the phone contact.

Facilitator: And how many? When you say phone contact, once a month? Once every few months? How many, roughly?

Interviewee: Well we’ve only had the one, haven’t we? So …

Facilitator: Yes, but if we were going to run it again, how many would you like to have?

Interviewee: Well I don’t know.

Facilitator: A couple?

Interviewee: Yeah, I reckon. Two or three throughout the year or something like that, I reckon. To keep the motivation going or something. Yeah.

Facilitator: Great. Anything else that you want to add about how we could improve the program?

Interviewee: No, I don’t think so.

Facilitator: Anything else that you think would be interesting for us to capture as part of evaluating the program? About the community that the town you live in?

Interviewee: I can’t think of anything. Not off the top of my head, anyway. No, I don’t know.

Facilitator: That’s no problem. Thank you so much for your time. We really, really appreciate you participating in this interview. It really gives us some valuable feedback that is really important to so that we can just continue improving the program. I’ll send you that gift card in the mail today. So if you don’t get it in the next week, please give us a call back and we’ll make sure that gets sent to you. Thank you so much again.

Interviewee: No worries. Thanks very much for that.

Facilitator: Thank you. Nice to speak to you. Thanks for that.

Interviewee: Thank you. Bye.

Facilitator: Bye.

Donald-J

FULL TRANSCRIPT

Facilitator: …work towards improving this program in the future knowing what worked for you and what didn’t.

Interviewee: Yes.

Facilitator: I was also wondering if you minded, so I don’t have to write everything down, do you mind if I tape record the interview?

Interviewee: No, that’s fine.

Facilitator: Great. To start off with, what we’re doing is we’re comparing the different towns we visited. We visited 42 towns, as you might remember, across Victoria. And I was hoping you could tell me just a little bit about Donald in terms of access to fruit and vegetables.

Interviewee: We have a supermarket and that’s our main point of access to fruit and vegetables. The quality varies but, as far as I’m aware, that’s the only place in town where you can purchase fruit and vegies unless you know someone who grows their own.

Facilitator: And in terms of the culture around buying locally, is that something that’s strong in Donald or not so much?

Interviewee: I think it’s quite strong but often if people are out of town, they will shop out of town. There are probably some people that shop out of town deliberately and other people that would shop if they were out of town to maybe get a bit more variety especially in their fruit and vege although sometimes it backfires because when I was in Bendigo last, I thought I’d take advantage of the fruit and vege market and it wasn’t very nice. I would have done better in the supermarket.

Facilitator: And what about exercise opportunities in Donald. Do you think they’re adequate or what’s available?

Interviewee: There probably is quite a lot available. It probably depends on what you’re into really. I know that I could go to, like, netball training or hockey training but it doesn’t really float my boat. I go to Yoga, which is once a week, and I used to have belly dancing and a bit before that they had line dancing. Just things that were sort of active but not necessarily team oriented, which were probably my preference but they’ve sort of folded. They’ve kind of had their run and people have moved on to other things.

Facilitator: Would you consider Donald to be quite a social town or lots of opportunities to socialise or one that’s a bit more isolated?

Interviewee: I think there is lots of opportunities if you know where to look. It’s not always promoted. If you’re new to town you might not instantly know what’s on and where. And it’s not hard to find out the information but you sort of have to know sometimes who to ask or where to look and it can be a little bit tricky if you’re new. If you’ve been here for a long time, most people seem to know what’s on and where but being in the kinder, I do tend to see people who are new to town and don’t know what’s out there.

Facilitator: Are you a local or are you quite new to town?

Interviewee: I’m not local but I’ve lived here for 11 years, maybe, so I’ve probably got a reasonable picture about what’s going on.

Facilitator: I think so, after 11 years.

Interviewee: Mmm. I’m actually a local.

Facilitator: I know, different. And In terms of when you think back to why you joined this program back in about December last year, was there anything that motivated you in particular to sign up for this program?

Interviewee: I was concerned about my health moving forward. I’m getting closer and closer to 40 and probably starting to think about I’ve still got young children so just trying to be more aware about my own health.

Facilitator: And are there any specific concerns you have with your health or your just sort of general health?

Interviewee: Probably some joint issues that I was experiencing and starting to think that weight was playing a bit of a role in some of those aches and pains.

Facilitator: Was there anything that made attending this program easy or made it difficult for you to attend?

Interviewee: No. It was in the evenings so the two sessions that you offered made it easy because I could pick the time that suited me better, that I’d be finished work but not have to have the kids with me.

Facilitator: Are there any other barriers that you think any other women in the town would have faced to coming along to this program that you can think of?

Interviewee: Maybe just the fact that the whole program revolves around you being weighed and measured would be probably the only…

Facilitator: And a lot of people had alluded to that, that that can be a bit confronting in a small town. Was that of any issue or concern to you?

Interviewee: No, because you were in another room so it wasn’t a bit issue. I had a minor concern that the scales were vastly different to my scales at home, which is never nice, but that was probably because they were accurate.

Facilitator: Don’t worry, you weren’t alone with that, we had a lot of people that hadn’t been on the scales in years so you certainly weren’t alone. What about the timing of the year, do you think that would have had any impact? We came around December last year. Is that a particularly busy time for the community?

Interviewee: For the people who are farming, probably it is a very busy time because I think that would have been in the midst of harvest. I’m not a farmer so it didn’t affect me directly but, having been here awhile, I’m starting to get into the…

Facilitator: The rhythm.

Interviewee: …the rhythm of what times are busy. And, at the moment, it’s quite busy because the crops are going in at the moment. So cropping and harvesting…

Facilitator: Yeah, so the busiest times are sort of May and - yeah. And in terms of when you think back to that program again, and I’m probably testing your memory here but in that first initial group session that we had in Donald, we gave a whole lot of information about general health advice that focussed on exercise and nutrition and then some advice that focussed on techniques like goal setting, action plans, relapse prevention, was any of that information new to you or more of a refresher, do you think?

Interviewee: Some of it was new. I think that the goal setting, it was almost like self-counselling, really, where you’re looking at your goal and then looking at the barriers to the goal and kind of rubbing out your excuses before you could make them was probably a bit of a new way of looking at it, if that makes sense.

Facilitator: Yeah, yeah. So that, for you, was a bit new. Any of the health information or not so much? Anything that stood out to you that was a particular focus of importance?

Interviewee: Not that I can remember. I kind of thing I know what to do, I’m just still not that great at doing.

Facilitator: Again, I’ve spoken to quite a lot of women since the program and they said that’s a big problem for them, it’s that activating the knowledge. Is that something you can associate with?

Interviewee: Yes, definitely.

Facilitator: And in terms of your impression about the program, if you were to describe it to somebody else, to say the program was about, what would you describe it as?

Interviewee: I’d say it was a very non-invasive program that offers support to try and improve your own health. So it’s kind of self-directed, self-guided but with support and assistance from outside.

Facilitator: And that was like you said about self-guiding. And, since starting this program, do you think it’s made you think any more or less or about the same about your weight, about your eating or about your exercise?

Interviewee: Yes, it has. It’s made me think about it more.

Facilitator: And what was the next sentence you were going to say?

Interviewee: It hasn’t been entirely effective but I don’t think I’ve gained any weight. I just don’t know that I would have lost any weight. I’m not sure if it was you that I spoke to a couple of weeks ago and I’ve sort of been a bit more conscious about decisions I’ve made. I’ve bought a treadmill, I know I don’t like to walk – it’s a second-hand one so it wasn’t that expensive.

Facilitator: It’s still a big move.

Interviewee: I’ve identified that I don’t really – it’s a small town, I don’t necessarily want to be walking the streets all the time and the hot weather and the cold weather were a bit of a problem for me so I thought I’d take the weather out of the equation and I found a reasonably priced, good quality treadmill and have now just got to commit to using it a bit more regularly.

Facilitator: And that treadmill, when you purchased it, was that as a result of speaking – I mean, obviously not solely but was that prompted by speaking to somebody? It wasn’t me, actually, it was somebody else. Was that as a result of speaking to somebody on the phone that prompted you to do that?

Interviewee: No, it was prompted by the evening that we had and I was thinking about ways of just incorporating more exercise because I bought it back in January, I think.

Facilitator: And so when you said about the first initial session, what we’re looking at is the value of each different component. So we had that Healthy Group session back in Donald, the evening one. We gave you a manual. You’ve had the SMS text messages and that phone coaching that you had with the other girl that that’s when you talked about exercising.

Interviewee: Yes.

Facilitator: Was one more useful to you or more valuable to you than another?

Interviewee: They’ve all had their value. I think the face-to-face session was a really good kind of kick-starter type thing and the manual, I took it around with me a few places in the initial stages and then it sort of got shelved but it’s still around.

Facilitator: And, don’t worry, that’s something I’ve heard a lot.

Interviewee: And the phone coaching, I thought, was a good kick in the bum to kind of keep doing it, going.

Facilitator: What about the text messaging?

Interviewee: Yeah, it was helpful because they’re all still on my phone so I can flick and have a look at them. Sometimes, because they would arrive at odd times, I’d kind of glance at it and go, yeah, I know what that is and then, because I’ve looked at it, it sort of goes read so it doesn’t – yeah, once I’d sort of looked at it, even if I hadn’t fully processed what it said, it would kind of then fade away into the back of my brain instead of the front part.

Facilitator: And a lot of women said that, for them, a text message wasn’t enough, they kind of needed that personal, more individualised support.

Interviewee: That’s probably where the phone coaching session was a bit valuable because you’ve got to ‘fess up and talk to a real person and think about why things have or haven’t been working.

Facilitator: So in terms of, for you, what helps to motivate you, from what I’m hearing, it’s that personal contact and, like you said, that talking to someone and being accountable. Is that correct?

Interviewee: Yes.

Facilitator: What else would help motivate you, do you think?

Interviewee: Things like tonight my dinner is being cooked by someone else, a nice cook who I know is sort of health focussed. So that should be a good meal with – he’ll have lots of vegies and things in it. But obviously that’s not practical for everybody.

Facilitator: So support?

Interviewee: Yes, and talking to other women who were sort of in the same situation is always good, which is a nice thing about the introduction people, it’s with other women so, being a small town, there’s other people there that you know which, for some people, might be a barrier, I guess, but if there’s people there that you know are doing it, it makes it a bit easier to commit to doing it yourself.

Facilitator: And have you spoken to any of those people since about the program or about health or anything like that?

Interviewee: Yeah. I’ve spoken to I think about three or four of the women who were down there I know quite well and I know some of them have started doing things like going to hockey training and just incorporating a bit more of what’s on offer around into their routine.

Facilitator: And. being honest, for you – again, we’re not expecting to see any large changes as the whole point of this program was a low intensity, non-invasive program, as you mentioned before – have you managed to make any changes to your lifestyle?

Interviewee: Probably not big ones just little ones like being more conscious of the vegies. I’m pretty good with vegies already but just trying to be on to my kids with their vegies. If I go away for the weekend, I can come back and find my husband hasn’t served the kids vegies all weekend but that’s probably an easy one that we can sort of keep a check on. And my husband has started playing hockey so he’s incorporating more exercise into this life which sort of has the flow-on effect that when we go somewhere, he’s more inclined to go, “Well, let’s just walk, it’s not that far.” So you kind of incorporate a bit of incidental exercise, more often than, perhaps, we would have 12 months ago.

Facilitator: So is that push coming from you or more from your husband?

Interviewee: I think it’s a bit of a combined thing because he’s aware that I’ve gone and have participated in this study, which, although it doesn’t directly impact him apart from what we may eat, and because some of my friends are doing it and they’re the families that we see a lot, it’s probably indirectly impacting.

Facilitator: And that’s one thing I was about to ask you about. In terms of indirect impact, that’s what we’re kind of looking at as to whether by you being in this program, it’s made any other influence on anyone else. So you’ve mentioned that it’s changed a little bit from what your family are eating. Any specific examples that you have?

Interviewee: My eldest son has sort of become a bit more conscious about what’s healthy and what’s not healthy. Part of that comes from trip to the dentist where he was reading all the posters that were there. And part of that comes from a just general discussions that we have about whether or not what he might want to eat is a good choice at that time of the night when we’re about to have tea.

Facilitator: And has that been more so since you’ve stared the program or no change?

Interviewee: No, most of it’s come about this year so it’s definitely since the program started. He’s not very sporty but he’s decided that he wants to do AusKick.

Facilitator: That’s good, yeah.

Interviewee: And he’s quite keen about that and likes the idea that he’s doing some exercise.

Facilitator: And in terms of support, do you feel like you’ve had enough support from your family to help make a few changes?

Interviewee: Sometimes yes and sometimes no, it really depends on how stressful my week’s been, if I’ve had a stressful week my husband’s inclined to do something like bring home a block of chocolate, which is a bit counter productive. I haven’t quite convinced him that a bunch of flowers would be just as effective.

Facilitator: But it sounds like he’s been helping you to do a bit more exercise sometimes?

Interviewee: Yes.

Facilitator: And going back to the behaviour changes, you mentioned just at the beginning that you’ve made some small changes to your exercise, has that been consistent or something, again being honest, that’s been a bit sort of fluctuating and not overly consistent?

Interviewee: Yeah, it hasn’t been overly consistent. It sort of comes up and goes down and then it comes up. So it’s been fluctuating probably more since the phone coaching a couple of weeks ago. I’ve been a bit more conscious going, you said you’d go for a walk, go for a walk.

Facilitator: So, for you, is it that personal contact will probably help you make more changes perhaps?

Interviewee: Mmm.

Facilitator: What about in terms of your diet? You mentioned that you’d put in a few more vegetables, anything else?

Interviewee: I’m just trying to think. Probably over the summer there was a period of a month or so when I was pretty good about salad but once the weather cools off, that tends to disappear off the radar.

Facilitator: And, again – sorry to be harping on about this but the exercise has that been more so since the phone coaching but prior to that I think you mentioned not as much as you had hoped for?

Interviewee: No, no. I purchased a treadmill and I used it a few times and it didn’t quite get to the stage of being a clothes rack, which I thought was a plus, but it’s been a bit neglected.

Facilitator: And you mentioned before also about the goal setting and stepping things out into small steps, have you been able to do that throughout the program or more so just at the beginning?

Interviewee: More so at the beginning and then in the last couple of weeks, sort of that window of time since I started working this year was very much survival mode. I had a very full-on first term which was quite exhausting mentally and, therefore, the whole physical – moving – didn’t really future. You’d get home and just go, that was a really hard day.

Facilitator: So a bit fluctuating?

Interviewee: Yeah. So things are settling at work now so it’s just a bit easier to then go back and focus on other areas. It was very much a work heavy February, March, April and now that things are sort of settling back down again it becomes a bit more doable to try and squeeze in that time for a walk or that time to actually plan what you’re going to eat that week instead of just grabbing something on the way home.

Facilitator: And it sounds like, you said, with the exercise, that spike in motivation was also a result of being accountable to a person?

Interviewee: Yes. That kind of reminder that, yes, you’re doing this program. [Cross talk] have to do it.

Facilitator: You don’t have to do anything. If we were to alter this program to make it better in the future, how do you think we could better support you?

Interviewee: Maybe if there was more opportunity for talking to somebody. Not too often but maybe just a little bit more often just so that you – because that does kind of make me go, right, yes, remember you were going to do this. More so than a text message probably.

Facilitator: And how often do you think that would be ideal for you?

Interviewee: Maybe, like, after three months instead of – I don’t know when it was, maybe it was only five months. Maybe just a little bit sooner after the initial session.

Facilitator: So, overall, where would you say you are with your satisfaction with how you’ve gone, do you think, again, being completely honest?

Interviewee: About halfway.

Facilitator: So made some changes, correct?

Interviewee: Yes. I’ve sort of explored some other causes. Because I was wondering if the pain I had in my hip and my foot was weight related but I’ve done some other things like finally gone to see a chiropractor and I’ve seen a podiatrist and I’ve just sort of made some other changes that are kind of still moving the right direction - it’ll probably be easier to talk if it’s not painful – that are all kind of baby steps in a way.

Facilitator: And you also mentioned before about activating that knowledge.

Interviewee: Yes, sitting down and going, why is it that you’re having trouble doing it and what can you do to get around it, pretty much, which was useful. But still, yeah, activating that is probably a bit of a sticking point.

Facilitator: In terms of motivating for you, where would you say you’re at with motivation?

Interviewee: My brain would say, yes, I’m motivated. My feet would say not so much. It depends how busy I’ve been that day really and how much time I feel like I’ve not spent with my family, if I want to go and do something else, which would, potentially, take me away from them again.

Facilitator: And in terms of whether you’d recommend this program to anybody else, do you think you would recommend it to anyone else in the community, again, being honest?

Interviewee: Yes, yes, I would.

Facilitator: And is there anyone in particular you’d recommend the program to that you think it would be most useful for?

Interviewee: Well, most of the people I’m friends with are doing it already but women that I see at kinder, I could probably recommend it to them or I’m part of a craft group, which is mainly women. They’d be sort of people that I might feel comfortable.

Facilitator: Do you feel comfortable talking about your health with your friends and weight and food?

Interviewee: Yeah. I talk about it from time to time.

Facilitator: And what about in terms of whether you’d prefer a group or an individual session? What would work best for you?

Interviewee: The group’s fine. I don’t have any problem with a group session.

Facilitator: Any benefits to a group session, do you think?

Interviewee: Often if you’re hearing that other people are struggling with the same thing or have got the same downfalls or have got the same attachment to biscuits or chocolate or the same difficulty in getting moving then it can be kind of encouraging to hear that other people are struggling with the same things or perhaps they’ve come up with a really great solution of how to get over that, which can be helpful.

Facilitator: Were you satisfied with the amount of information you were provided with?

Interviewee: Yeah, that was really good.

Facilitator: And any preference for whether that facilitator was from Melbourne or was a local person?

Interviewee: No, it didn’t really matter. In a way it was nice that was someone different, someone who maybe wasn’t thinking, I’ve given you this message three times already, you’re still here.

Facilitator: And are there any traits that you liked about that facilitator or that you’d recommend that we need to look for when looking at people to run this program?

Interviewee: Not that I can think of. It was fine.

Facilitator: And, last question, besides a bit of additional support to keep you on track, is there anything else that you’d recommend changing?

Interviewee: No, I think it’s pretty good the way it is. I don’t know if you can incorporate a little plug for grant writing to get more – the only thing I’d like to see in town would be some kind of a gym but it’s a bit hard to get those sort of things up and running out here. A little plug for grant writing to get that sort of thing off the ground would be helpful but that’s not really the same area.

Facilitator: That would be helpful for you, more exercise opportunities?

Interviewee: Yeah, that didn’t revolve around the sporting teams.

Facilitator: Anything else that you’d recommend?

Interviewee: No, I think that’s about it really.

Facilitator: One lady suggested like a Facebook group to keep in touch with the other people in the program and to bounce ideas off each other. Would that be something you’d think would be helpful to you?

Interviewee: Yeah, maybe. I do seem to go to Facebook for certain groups that I’m in. So, yeah, it could be.

Facilitator: That was just one idea that we thought was interesting.

Interviewee: Yeah. No, it is an interesting idea because they’re very helpful for some things.

Facilitator: This lady was on the Michelle Bridges diet and she said that’s why she liked it, because it helped give her support and in Port Fairy, then all the women could meet up and go for walks together and get that additional support.

Interviewee: There you go, yeah.

Facilitator: But it might be something we might think about in the future.

Interviewee: Mmm, yeah. No, that’s a good idea.

Facilitator: Well, besides that, unless you have anything else to add that you think is important for us to know about Donald, that was pretty much all I wanted to ask you.

Interviewee: That’s good. No, so far, so good. I just hope the scales will be down by next December and not up.

Facilitator: Yeah, hopefully it all goes well.

Interviewee: Yes.

Facilitator: And if you need any support, please give us a call. We’re always here.

Interviewee: Okay.

Facilitator: And can I just grab your address, Jenny, just to make sure I send that…

Donald-J

FULL TRANSCRIPT

Facilitator: …work towards improving this program in the future knowing what worked for you and what didn’t.

Interviewee: Yes.

Facilitator: I was also wondering if you minded, so I don’t have to write everything down, do you mind if I tape record the interview?

Interviewee: No, that’s fine.

Facilitator: Great. To start off with, what we’re doing is we’re comparing the different towns we visited. We visited 42 towns, as you might remember, across Victoria. And I was hoping you could tell me just a little bit about Donald in terms of access to fruit and vegetables.

Interviewee: We have a supermarket and that’s our main point of access to fruit and vegetables. The quality varies but, as far as I’m aware, that’s the only place in town where you can purchase fruit and vegies unless you know someone who grows their own.

Facilitator: And in terms of the culture around buying locally, is that something that’s strong in Donald or not so much?

Interviewee: I think it’s quite strong but often if people are out of town, they will shop out of town. There are probably some people that shop out of town deliberately and other people that would shop if they were out of town to maybe get a bit more variety especially in their fruit and vege although sometimes it backfires because when I was in Bendigo last, I thought I’d take advantage of the fruit and vege market and it wasn’t very nice. I would have done better in the supermarket.

Facilitator: And what about exercise opportunities in Donald. Do you think they’re adequate or what’s available?

Interviewee: There probably is quite a lot available. It probably depends on what you’re into really. I know that I could go to, like, netball training or hockey training but it doesn’t really float my boat. I go to Yoga, which is once a week, and I used to have belly dancing and a bit before that they had line dancing. Just things that were sort of active but not necessarily team oriented, which were probably my preference but they’ve sort of folded. They’ve kind of had their run and people have moved on to other things.

Facilitator: Would you consider Donald to be quite a social town or lots of opportunities to socialise or one that’s a bit more isolated?

Interviewee: I think there is lots of opportunities if you know where to look. It’s not always promoted. If you’re new to town you might not instantly know what’s on and where. And it’s not hard to find out the information but you sort of have to know sometimes who to ask or where to look and it can be a little bit tricky if you’re new. If you’ve been here for a long time, most people seem to know what’s on and where but being in the kinder, I do tend to see people who are new to town and don’t know what’s out there.

Facilitator: Are you a local or are you quite new to town?

Interviewee: I’m not local but I’ve lived here for 11 years, maybe, so I’ve probably got a reasonable picture about what’s going on.

Facilitator: I think so, after 11 years.

Interviewee: Mmm. I’m actually a local.

Facilitator: I know, different. And In terms of when you think back to why you joined this program back in about December last year, was there anything that motivated you in particular to sign up for this program?

Interviewee: I was concerned about my health moving forward. I’m getting closer and closer to 40 and probably starting to think about I’ve still got young children so just trying to be more aware about my own health.

Facilitator: And are there any specific concerns you have with your health or your just sort of general health?

Interviewee: Probably some joint issues that I was experiencing and starting to think that weight was playing a bit of a role in some of those aches and pains.

Facilitator: Was there anything that made attending this program easy or made it difficult for you to attend?

Interviewee: No. It was in the evenings so the two sessions that you offered made it easy because I could pick the time that suited me better, that I’d be finished work but not have to have the kids with me.

Facilitator: Are there any other barriers that you think any other women in the town would have faced to coming along to this program that you can think of?

Interviewee: Maybe just the fact that the whole program revolves around you being weighed and measured would be probably the only…

Facilitator: And a lot of people had alluded to that, that that can be a bit confronting in a small town. Was that of any issue or concern to you?

Interviewee: No, because you were in another room so it wasn’t a bit issue. I had a minor concern that the scales were vastly different to my scales at home, which is never nice, but that was probably because they were accurate.

Facilitator: Don’t worry, you weren’t alone with that, we had a lot of people that hadn’t been on the scales in years so you certainly weren’t alone. What about the timing of the year, do you think that would have had any impact? We came around December last year. Is that a particularly busy time for the community?

Interviewee: For the people who are farming, probably it is a very busy time because I think that would have been in the midst of harvest. I’m not a farmer so it didn’t affect me directly but, having been here awhile, I’m starting to get into the…

Facilitator: The rhythm.

Interviewee: …the rhythm of what times are busy. And, at the moment, it’s quite busy because the crops are going in at the moment. So cropping and harvesting…

Facilitator: Yeah, so the busiest times are sort of May and - yeah. And in terms of when you think back to that program again, and I’m probably testing your memory here but in that first initial group session that we had in Donald, we gave a whole lot of information about general health advice that focussed on exercise and nutrition and then some advice that focussed on techniques like goal setting, action plans, relapse prevention, was any of that information new to you or more of a refresher, do you think?

Interviewee: Some of it was new. I think that the goal setting, it was almost like self-counselling, really, where you’re looking at your goal and then looking at the barriers to the goal and kind of rubbing out your excuses before you could make them was probably a bit of a new way of looking at it, if that makes sense.

Facilitator: Yeah, yeah. So that, for you, was a bit new. Any of the health information or not so much? Anything that stood out to you that was a particular focus of importance?

Interviewee: Not that I can remember. I kind of thing I know what to do, I’m just still not that great at doing.

Facilitator: Again, I’ve spoken to quite a lot of women since the program and they said that’s a big problem for them, it’s that activating the knowledge. Is that something you can associate with?

Interviewee: Yes, definitely.

Facilitator: And in terms of your impression about the program, if you were to describe it to somebody else, to say the program was about, what would you describe it as?

Interviewee: I’d say it was a very non-invasive program that offers support to try and improve your own health. So it’s kind of self-directed, self-guided but with support and assistance from outside.

Facilitator: And that was like you said about self-guiding. And, since starting this program, do you think it’s made you think any more or less or about the same about your weight, about your eating or about your exercise?

Interviewee: Yes, it has. It’s made me think about it more.

Facilitator: And what was the next sentence you were going to say?

Interviewee: It hasn’t been entirely effective but I don’t think I’ve gained any weight. I just don’t know that I would have lost any weight. I’m not sure if it was you that I spoke to a couple of weeks ago and I’ve sort of been a bit more conscious about decisions I’ve made. I’ve bought a treadmill, I know I don’t like to walk – it’s a second-hand one so it wasn’t that expensive.

Facilitator: It’s still a big move.

Interviewee: I’ve identified that I don’t really – it’s a small town, I don’t necessarily want to be walking the streets all the time and the hot weather and the cold weather were a bit of a problem for me so I thought I’d take the weather out of the equation and I found a reasonably priced, good quality treadmill and have now just got to commit to using it a bit more regularly.

Facilitator: And that treadmill, when you purchased it, was that as a result of speaking – I mean, obviously not solely but was that prompted by speaking to somebody? It wasn’t me, actually, it was somebody else. Was that as a result of speaking to somebody on the phone that prompted you to do that?

Interviewee: No, it was prompted by the evening that we had and I was thinking about ways of just incorporating more exercise because I bought it back in January, I think.

Facilitator: And so when you said about the first initial session, what we’re looking at is the value of each different component. So we had that Healthy Group session back in Donald, the evening one. We gave you a manual. You’ve had the SMS text messages and that phone coaching that you had with the other girl that that’s when you talked about exercising.

Interviewee: Yes.

Facilitator: Was one more useful to you or more valuable to you than another?

Interviewee: They’ve all had their value. I think the face-to-face session was a really good kind of kick-starter type thing and the manual, I took it around with me a few places in the initial stages and then it sort of got shelved but it’s still around.

Facilitator: And, don’t worry, that’s something I’ve heard a lot.

Interviewee: And the phone coaching, I thought, was a good kick in the bum to kind of keep doing it, going.

Facilitator: What about the text messaging?

Interviewee: Yeah, it was helpful because they’re all still on my phone so I can flick and have a look at them. Sometimes, because they would arrive at odd times, I’d kind of glance at it and go, yeah, I know what that is and then, because I’ve looked at it, it sort of goes read so it doesn’t – yeah, once I’d sort of looked at it, even if I hadn’t fully processed what it said, it would kind of then fade away into the back of my brain instead of the front part.

Facilitator: And a lot of women said that, for them, a text message wasn’t enough, they kind of needed that personal, more individualised support.

Interviewee: That’s probably where the phone coaching session was a bit valuable because you’ve got to ‘fess up and talk to a real person and think about why things have or haven’t been working.

Facilitator: So in terms of, for you, what helps to motivate you, from what I’m hearing, it’s that personal contact and, like you said, that talking to someone and being accountable. Is that correct?

Interviewee: Yes.

Facilitator: What else would help motivate you, do you think?

Interviewee: Things like tonight my dinner is being cooked by someone else, a nice cook who I know is sort of health focussed. So that should be a good meal with – he’ll have lots of vegies and things in it. But obviously that’s not practical for everybody.

Facilitator: So support?

Interviewee: Yes, and talking to other women who were sort of in the same situation is always good, which is a nice thing about the introduction people, it’s with other women so, being a small town, there’s other people there that you know which, for some people, might be a barrier, I guess, but if there’s people there that you know are doing it, it makes it a bit easier to commit to doing it yourself.

Facilitator: And have you spoken to any of those people since about the program or about health or anything like that?

Interviewee: Yeah. I’ve spoken to I think about three or four of the women who were down there I know quite well and I know some of them have started doing things like going to hockey training and just incorporating a bit more of what’s on offer around into their routine.

Facilitator: And. being honest, for you – again, we’re not expecting to see any large changes as the whole point of this program was a low intensity, non-invasive program, as you mentioned before – have you managed to make any changes to your lifestyle?

Interviewee: Probably not big ones just little ones like being more conscious of the vegies. I’m pretty good with vegies already but just trying to be on to my kids with their vegies. If I go away for the weekend, I can come back and find my husband hasn’t served the kids vegies all weekend but that’s probably an easy one that we can sort of keep a check on. And my husband has started playing hockey so he’s incorporating more exercise into this life which sort of has the flow-on effect that when we go somewhere, he’s more inclined to go, “Well, let’s just walk, it’s not that far.” So you kind of incorporate a bit of incidental exercise, more often than, perhaps, we would have 12 months ago.

Facilitator: So is that push coming from you or more from your husband?

Interviewee: I think it’s a bit of a combined thing because he’s aware that I’ve gone and have participated in this study, which, although it doesn’t directly impact him apart from what we may eat, and because some of my friends are doing it and they’re the families that we see a lot, it’s probably indirectly impacting.

Facilitator: And that’s one thing I was about to ask you about. In terms of indirect impact, that’s what we’re kind of looking at as to whether by you being in this program, it’s made any other influence on anyone else. So you’ve mentioned that it’s changed a little bit from what your family are eating. Any specific examples that you have?

Interviewee: My eldest son has sort of become a bit more conscious about what’s healthy and what’s not healthy. Part of that comes from trip to the dentist where he was reading all the posters that were there. And part of that comes from a just general discussions that we have about whether or not what he might want to eat is a good choice at that time of the night when we’re about to have tea.

Facilitator: And has that been more so since you’ve stared the program or no change?

Interviewee: No, most of it’s come about this year so it’s definitely since the program started. He’s not very sporty but he’s decided that he wants to do AusKick.

Facilitator: That’s good, yeah.

Interviewee: And he’s quite keen about that and likes the idea that he’s doing some exercise.

Facilitator: And in terms of support, do you feel like you’ve had enough support from your family to help make a few changes?

Interviewee: Sometimes yes and sometimes no, it really depends on how stressful my week’s been, if I’ve had a stressful week my husband’s inclined to do something like bring home a block of chocolate, which is a bit counter productive. I haven’t quite convinced him that a bunch of flowers would be just as effective.

Facilitator: But it sounds like he’s been helping you to do a bit more exercise sometimes?

Interviewee: Yes.

Facilitator: And going back to the behaviour changes, you mentioned just at the beginning that you’ve made some small changes to your exercise, has that been consistent or something, again being honest, that’s been a bit sort of fluctuating and not overly consistent?

Interviewee: Yeah, it hasn’t been overly consistent. It sort of comes up and goes down and then it comes up. So it’s been fluctuating probably more since the phone coaching a couple of weeks ago. I’ve been a bit more conscious going, you said you’d go for a walk, go for a walk.

Facilitator: So, for you, is it that personal contact will probably help you make more changes perhaps?

Interviewee: Mmm.

Facilitator: What about in terms of your diet? You mentioned that you’d put in a few more vegetables, anything else?

Interviewee: I’m just trying to think. Probably over the summer there was a period of a month or so when I was pretty good about salad but once the weather cools off, that tends to disappear off the radar.

Facilitator: And, again – sorry to be harping on about this but the exercise has that been more so since the phone coaching but prior to that I think you mentioned not as much as you had hoped for?

Interviewee: No, no. I purchased a treadmill and I used it a few times and it didn’t quite get to the stage of being a clothes rack, which I thought was a plus, but it’s been a bit neglected.

Facilitator: And you mentioned before also about the goal setting and stepping things out into small steps, have you been able to do that throughout the program or more so just at the beginning?

Interviewee: More so at the beginning and then in the last couple of weeks, sort of that window of time since I started working this year was very much survival mode. I had a very full-on first term which was quite exhausting mentally and, therefore, the whole physical – moving – didn’t really future. You’d get home and just go, that was a really hard day.

Facilitator: So a bit fluctuating?

Interviewee: Yeah. So things are settling at work now so it’s just a bit easier to then go back and focus on other areas. It was very much a work heavy February, March, April and now that things are sort of settling back down again it becomes a bit more doable to try and squeeze in that time for a walk or that time to actually plan what you’re going to eat that week instead of just grabbing something on the way home.

Facilitator: And it sounds like, you said, with the exercise, that spike in motivation was also a result of being accountable to a person?

Interviewee: Yes. That kind of reminder that, yes, you’re doing this program. [Cross talk] have to do it.

Facilitator: You don’t have to do anything. If we were to alter this program to make it better in the future, how do you think we could better support you?

Interviewee: Maybe if there was more opportunity for talking to somebody. Not too often but maybe just a little bit more often just so that you – because that does kind of make me go, right, yes, remember you were going to do this. More so than a text message probably.

Facilitator: And how often do you think that would be ideal for you?

Interviewee: Maybe, like, after three months instead of – I don’t know when it was, maybe it was only five months. Maybe just a little bit sooner after the initial session.

Facilitator: So, overall, where would you say you are with your satisfaction with how you’ve gone, do you think, again, being completely honest?

Interviewee: About halfway.

Facilitator: So made some changes, correct?

Interviewee: Yes. I’ve sort of explored some other causes. Because I was wondering if the pain I had in my hip and my foot was weight related but I’ve done some other things like finally gone to see a chiropractor and I’ve seen a podiatrist and I’ve just sort of made some other changes that are kind of still moving the right direction - it’ll probably be easier to talk if it’s not painful – that are all kind of baby steps in a way.

Facilitator: And you also mentioned before about activating that knowledge.

Interviewee: Yes, sitting down and going, why is it that you’re having trouble doing it and what can you do to get around it, pretty much, which was useful. But still, yeah, activating that is probably a bit of a sticking point.

Facilitator: In terms of motivating for you, where would you say you’re at with motivation?

Interviewee: My brain would say, yes, I’m motivated. My feet would say not so much. It depends how busy I’ve been that day really and how much time I feel like I’ve not spent with my family, if I want to go and do something else, which would, potentially, take me away from them again.

Facilitator: And in terms of whether you’d recommend this program to anybody else, do you think you would recommend it to anyone else in the community, again, being honest?

Interviewee: Yes, yes, I would.

Facilitator: And is there anyone in particular you’d recommend the program to that you think it would be most useful for?

Interviewee: Well, most of the people I’m friends with are doing it already but women that I see at kinder, I could probably recommend it to them or I’m part of a craft group, which is mainly women. They’d be sort of people that I might feel comfortable.

Facilitator: Do you feel comfortable talking about your health with your friends and weight and food?

Interviewee: Yeah. I talk about it from time to time.

Facilitator: And what about in terms of whether you’d prefer a group or an individual session? What would work best for you?

Interviewee: The group’s fine. I don’t have any problem with a group session.

Facilitator: Any benefits to a group session, do you think?

Interviewee: Often if you’re hearing that other people are struggling with the same thing or have got the same downfalls or have got the same attachment to biscuits or chocolate or the same difficulty in getting moving then it can be kind of encouraging to hear that other people are struggling with the same things or perhaps they’ve come up with a really great solution of how to get over that, which can be helpful.

Facilitator: Were you satisfied with the amount of information you were provided with?

Interviewee: Yeah, that was really good.

Facilitator: And any preference for whether that facilitator was from Melbourne or was a local person?

Interviewee: No, it didn’t really matter. In a way it was nice that was someone different, someone who maybe wasn’t thinking, I’ve given you this message three times already, you’re still here.

Facilitator: And are there any traits that you liked about that facilitator or that you’d recommend that we need to look for when looking at people to run this program?

Interviewee: Not that I can think of. It was fine.

Facilitator: And, last question, besides a bit of additional support to keep you on track, is there anything else that you’d recommend changing?

Interviewee: No, I think it’s pretty good the way it is. I don’t know if you can incorporate a little plug for grant writing to get more – the only thing I’d like to see in town would be some kind of a gym but it’s a bit hard to get those sort of things up and running out here. A little plug for grant writing to get that sort of thing off the ground would be helpful but that’s not really the same area.

Facilitator: That would be helpful for you, more exercise opportunities?

Interviewee: Yeah, that didn’t revolve around the sporting teams.

Facilitator: Anything else that you’d recommend?

Interviewee: No, I think that’s about it really.

Facilitator: One lady suggested like a Facebook group to keep in touch with the other people in the program and to bounce ideas off each other. Would that be something you’d think would be helpful to you?

Interviewee: Yeah, maybe. I do seem to go to Facebook for certain groups that I’m in. So, yeah, it could be.

Facilitator: That was just one idea that we thought was interesting.

Interviewee: Yeah. No, it is an interesting idea because they’re very helpful for some things.

Facilitator: This lady was on the Michelle Bridges diet and she said that’s why she liked it, because it helped give her support and in Port Fairy, then all the women could meet up and go for walks together and get that additional support.

Interviewee: There you go, yeah.

Facilitator: But it might be something we might think about in the future.

Interviewee: Mmm, yeah. No, that’s a good idea.

Facilitator: Well, besides that, unless you have anything else to add that you think is important for us to know about Donald, that was pretty much all I wanted to ask you.

Interviewee: That’s good. No, so far, so good. I just hope the scales will be down by next December and not up.

Facilitator: Yeah, hopefully it all goes well.

Interviewee: Yes.

Facilitator: And if you need any support, please give us a call. We’re always here.

Interviewee: Okay.

Facilitator: And can I just grab your address, Jenny, just to make sure I send that…

End of transcript

Participant #FS

Facilitator: To start off with what we’re doing is comparing the different towns that we visited. The 42 towns we’ve been to as part of this program and I was hoping you could tell me a little bit about the access to fruit and vegetables in Donald?

Interviewee: No, it’s not real good. The supermarket is very, very inconsistent with their quality and the price is just generally through the roof.

Facilitator: What about in terms of exercise opportunities?

Interviewee: Look, there are organised things available but in terms of facilities, they’re fairly limited, you know, like a gym that you can go to when it suits you. There’s nothing like that.

Facilitator: Any other major barriers to exercising do you think?

Interviewee: Well I have children and a grumpy husband so they’re my barriers.

Facilitator: Overall do you think the community would be I mean, it’s a bit hard to say but overall, on average, an active one or not so much do you think?

Interviewee: Oh look, they’re fairly active. You sort of you know, it’s like any community you’ve got parts that are really sporty and parts that aren’t.

Facilitator: What about in terms of a social community? Would you consider the community to be quite well connected with each other or one that’s more isolated and cliquey?

Interviewee: Don’t know. I tend to live in my own little world so I find it quite connected but I know other people have complained that it’s cliquey but I don’t really find that.

Facilitator: In terms of programs like this being available before in Donald, has there ever been anything like this before in Donald?

Interviewee: No, look, not that I’m aware of.

Facilitator: If you think back to when you joined this program, back in December last year, almost six months ago now. Do you remember what motivated you to join this program?

Interviewee: Just interest, like you know to see whether it was something that you know maybe a little bit different and look probably – I studied at Monash so there’s probably a little bit of – I like Monash so…

Facilitator: So would it be fair to say you sort of did it to help out with the research and help the evidence?

Interviewee: Yes.

Facilitator: Did you come to the program alone or with friends?

Interviewee: With friends.

Facilitator: A lot of people mentioned that they prefer to come to programs with people they know. Is that better for you or what do you think?

Interviewee: Yes.

Facilitator: If you think back to – we came in December last year, was there any factors that you think may have stopped other people in the community from not coming to that session?

Interviewee: Oh it may have been the time didn’t suit or you know this end is always very busy. Look I really don’t know.

Facilitator: Some people mentioned that some people might not come because being a small town; they don’t like to talk about their health when other people know them in the room. Do you think that could have been a problem?

Interviewee: Oh look maybe.

Facilitator: Yes? Not sure?

Interviewee: No look, maybe.

Facilitator: No problem and when we think back to that first healthy lifestyle session we had, did you come to the school or the evening session?

Interviewee: The evening.

Facilitator: The evening one? At the evening session and at the school, they were both the same content, we talked about general health information related to exercise and nutrition and then we focused on behaviour change techniques like goal setting, developing, action plans and we all worked together to do an action plan. Was any of this information new to you or was it more of a refresher do you think?

Interviewee: No it was actually quite new to me. The idea that, oh look you know I think because you’ve always sort of thought about you either had to go on a health binge all of a sudden. Throw out the Tim Tams, sort of Michelle Bridges type style or the whole breaking it down and just setting little incremental goals. I really liked that. That was achievable.

Facilitator: So that achievable goals?

Interviewee: Yes.

Facilitator: Have you done anything like the Michelle Bridges before or anything like that?

Interviewee: I have actually come off a big failure of Michelle Bridges where a couple of weeks into it I went, I don’t have time for this. You know what I mean? It was like, oh for God’s sake.

Facilitator: So when did you do the Michelle Bridges?

Interviewee: Towards the end. It was in November.

Facilitator: Yes and it didn’t work for you?

Interviewee: Oh in a couple of weeks into it, I went; I have a husband, I have children, I cannot dedicate this amount of time. I’ve got two little boys with special needs, like it was just you know, if I was a single woman, yes maybe that could work, but no.

Facilitator: It’s because of the time commitment?

Interviewee: The time, the access to like some of the ingredients, they’re just – yes, the whole – it had to become your entire focus and it just doesn’t work for me.

Facilitator: So in contrast to the Michelle Bridges program, this program was obviously a lot more, less intensive and a lot more you know, the onus being on yourself. What works better for you do you think?

Interviewee: Obviously the less intensive, small steps, yes and just I really like the kind of I don’t know, the realistic view that you guys had. That you know, it’s – you’re busy but little things can help.

Facilitator: Yes and if you think now about this healthy lifestyle, you’ve already sort of alluded to this quite a lot but if you were to describe this program to somebody else, how would you describe it? If you were to finish my sentence; the program was about…

Interviewee: Common sense.

Facilitator: Yes and in terms of you – in terms of your opinion, do you think the program was more focused on healthy lifestyle or weight or exercise or nutrition? What did you take away? What was the take home message for you?

Interviewee: I took home that it was about choices. That it’s the small choices that make a difference.

Facilitator: Yes a really good point and in terms of the nutrition information that was given, was that, you know, we talked about no soft drink, you know eating breakfast regularly. Was that information new to you or was it more the behaviour change techniques new to you?

Interviewee: I think the nutrition information wasn’t new but how you applied it was.

Facilitator: Yes and have you found that information easy to apply?

Interviewee: Yes I have actually I have. Like you know I won’t say that I’m the fittest woman on the planet now but I certainly have cut out the soft drink. I don’t have soft drink anymore.

Facilitator: That’s great.

Interviewee: I am making an effort when, you know, I go out for lunch to just choose a better choice.

Facilitator: Yes and what we’re interested in doing is trying to work out why this program’s worked for you versus perhaps others in the past.

What’s made it easier for you to do it? You know to make those really important changes this time around do you think?

Interviewee: I think it was the way that it was presented really. That they – that is seems achievable.

Facilitator: Yes and the support provided, has that been adequate to you or do you think more support was needed?

Interviewee: Probably more support. I would have liked to have seen a few more of those sort of group sessions. I think you walk away from those feeling a lot more inspired.

Facilitator: So what’s the motivation been like for you? Some women talked about being quite motivated post that group session. You know, quite motivated once they’d spoken to somebody and then it’s dropped, whereas other women, it’s been pretty consistently high. What’s it like for you?

Interviewee: Yes look, it’s probably a bit spasmodic. I always get a bit more like – I think, you know, when you speak to someone or whatever, it just brings it back to the top of your to do list. Whereas, you know, every day, more things get added to the list, so things get further down. Does that make sense?

Facilitator: Yes of course. Of course, especially with a busy lifestyle and would you say since starting this program it’s made you think more about your weight, what you eat or since you exercise? Or would it be about the same, since you know as before you started?

Interviewee: No it’s probably helped yes, say you know, daily but I think it’s just brought it up in my awareness a little bit more.

Facilitator: Good. Everyone’s got a sort of a different impression of a healthy lifestyle and we’ve heard some interesting ideas from women. What does a healthy lifestyle mean to you do you think?

Interviewee: Interesting, probably about feeling healthy.

Facilitator: What makes you feel healthy?

Interviewee: When you haven’t just eaten a packet of Tim Tams. No, when you sort of you’re carrying less weight, you’re a bit more active, you’re not so tired, you’re a little bit more able to cope. Where you know, when you spot your four year old up on top of the shed, you know, you sort of are able to climb up after him and get him down instead of just going, oh my God, really?

Facilitator: So what motivates you do you think, to be healthy or to try and be healthy?

Interviewee: My children.

Facilitator: Anything else?

Interviewee: Probably the way I feel.

Facilitator: Is that what you were referring to before about the weight and?

Interviewee: Yes.

Facilitator: Yes and if you were to look at the different components of the program, so we had the healthy lifestyle group session, the evening one in Donald. We gave you a program manual. You’ve had the SMS messages. We have a website and a couple of weeks back, you got a phone call to set an action plan and to set some goals with a – with the dietician. What’s been most useful to you do you think?

Interviewee: The group session.

Facilitator: What did you think about the manual? I mean some – we’ve had a lot of mixed responses. Some women have found it really useful, others not so much. How’s it been for you?

Interviewee: It’s been absent for me because my children drew all over it and then cut it up.

Facilitator: Did you do any of the activities initially or read through it initially or that was the children cut it up quite quickly?

Interviewee: They cut it up quite quickly. I did the one at the session and I think I did one more.

Facilitator: Is that a useful way for you to you know, make changes? Some women said it did – they didn’t use it because they don’t like writing things down. They don’t have enough time. Any reason as to why, I mean obviously the children, I mean it’s not available because they cut it up, but, is it something you would have used anyway? Or can you relate to those factors?

Interviewee: Look probably not. I think maybe something like that would be useful if like I said you took it to a group session and then you had allocated time to do it, think about it. I think that would have probably been a bit more useful. But yes, it just kind of gets lost under all of the paperwork otherwise.

Facilitator: What about those SMSs? Do they help you?

Interviewee: Yes, I didn’t mind them.

Facilitator: Yes and some women said that they found them quite motivating. Other women said they just sort of read it then deleted them, so didn’t really have much influence on them. What was it like for you?

Interviewee: I kind of read them. I went, oh yes, that’s right and a couple of times I yes, applied them but yes. So probably a little bit spasmodic.

Facilitator: Yes of course and the phone call, did that help you at all?

Interviewee: Yes it did actually.

Facilitator: Yes and a lot of women talked about that being about the personal support and you know, making individual goals and being a bit accountable. Is that like similar for you?

Interviewee: Yes I would say so.

Facilitator: If we were to take away one of those program components, we also had a website. Have you used that?

Interviewee: No I haven’t.

Facilitator: Do you normally access health information from the web?

Interviewee: Yes, sometimes but no, not a great deal.

Facilitator: So if we were to take away any of those components. Any of them you’d suggest that we could take away that wouldn’t have any influence on your progress.

Interviewee: Oh probably the website.

Facilitator: The website? What about the manual? If you didn’t have the manual?

Interviewee: Yes probably. But like I said, I think if you combine that with a group session I think you would find that that would be more effective.

Facilitator: Yes so keep the manual and you mentioned before, what we are looking at is the behaviours you’ve made as we’ve – as you touched on before, they’re not – we’re not expecting any large changes from this program because it was really about setting small goals. You mentioned the soft drink. Has that been sustainable or has that been something you’ve been able to do for a little bit but then stopped doing and then start it again?

Interviewee: No it’s been sustainable. I’ve had oh, half a carton of diet coke sitting up the top of my cupboard now for about six months.

Facilitator: So yes, you’ve cut that out?

Interviewee: Yes.

Facilitator: That’s great. What about – and then you said choosing healthier options, has that also been sustainable or something that you’ve done a little bit but been less able to sustain?

Interviewee: I’m not saying that you know, I don’t go to a café and have the cake but the main might be a bit more of a healthy choice. Yes, it’s been sustainable.

Facilitator: Anything else you’ve done differently since starting this program?

Interviewee: No. I’m just trying to move around a bit more.

Facilitator: How’s that gone?

Interviewee: Yes not too bad. Not too bad, just trying not to sit for extended periods. Get up and do something.

Facilitator: So a bit more of that incidental exercise perhaps?

Interviewee: Yes.

Facilitator: Is there something that – any strategies that we could have used to make you to be able to be more motivated or to make more changes do you think? You mentioned a bit more contact, anything else?

Interviewee: No, that would probably be about it.

Facilitator: What about in terms of social support? Do you feel well supported by your family or not?

Interviewee: No, not really. They wouldn’t have a clue really.

Facilitator: What about friends?

Interviewee: Yes. Friends are.

Facilitator: When you’ve made these behaviour changes, what we’re looking at is whether you’ve been able to use any of that goal setting, smaller steps, you know, those action plans. So you know, outlining barriers and coming up with your own solutions. Do you think you would have used any of those strategies to reduce the soft drink and choose better meals?

Interviewee: Look, yes probably. I don’t know actually. I don’t know. Yes, I don’t know.

Facilitator: In terms of since starting this program, do you think it’s influenced your family in any way? Have you made any changes that would have affected your family?

Interviewee: Yes probably just trying to introduce like you know a few healthier choices without giving them the actual choice.

Facilitator: Have you talked more about your health with them or just more changed some foods.

Interviewee: Oh probably just changed some foods.

Facilitator: What about anyone outside your family? Have you spoken about this program or about your health with anyone, any of your friends or anyone else that attended that session in Donald?

Interviewee: Yes to my friends who were there.

Facilitator: Do you have any examples of how you’ve – anything you’ve talked about them with or anything that you’ve done together to make a healthy change?

Interviewee: Oh no not really. We kind of go – oh yes, I got a phone call, yeah, and just kind of talked about it. No, yes so…

Facilitator: The last thing I wanted to focus on is just to look at your satisfaction with the program so far. So based on your experience, would you recommend this program to anybody else in your community?

Interviewee: Yes definitely.

Facilitator: Any reasons as to why that would be?

Interviewee: Because it’s practical. Because yes…

Facilitator: Is there anyone that you think this program would work best for? So some people have mentioned mothers, some people have mentioned overweight people. Some people have mentioned you know, men. Anyone that you think this program would work best for?

Interviewee: Probably, yes, mothers really because I am a mother and that’s sort of my focus, yes.

Facilitator: What about the facilitator? Do you have any preference whether that person was from Melbourne or was a local person?

Interviewee: Oh it doesn’t really worry me, no.

Facilitator: Any traits that you think re important for that person that runs that session? You know, knowledgeable, a healthy weight? Anything that stands out to you as being important?

Interviewee: I would say there’s – I would say sense of humour.

Facilitator: Yes and the venue. Was that an okay location for you?

Interviewee: Yes that was fine.

Facilitator: What about a group session versus an individual? Do you have any preference or what would you prefer?

Interviewee: I quite like the group session?

Facilitator: Anything about the group that works for you?

Interviewee: Just you can sort of have a laugh and go, oh, yes. No that make sense, yes I do that to – that kind of you know…

Facilitator: So relating to others?

Interviewee: Yes.

Facilitator: Do you think the information provided was adequate? Or was there anything else that you think would be good to add to this program?

Interviewee: No I think it was fine.

Facilitator: If you were to sort of you know think about how well you’ve done or whether you’ve met some of your goals that you set at the beginning of this program, how satisfied are you with where you’re at?

Interviewee: Out of ten, probably about a six, seven.

Facilitator: Yes so you’re happy with some of the changes you’ve made but perhaps some further ones as well?

Interviewee: Oh yes. You can always improve, can’t you? Yes, if I look at it, I’m probably better off where I am now than I was six months ago.

Facilitator: Great and in terms of the level of support, some people said they were happy with the support, others were disappointed. What’s it like for you? That we’ve provided? Sorry…

Interviewee: Look it was fine probably yes, more of the group or the face to face stuff would have been great.

Facilitator: Some women mentioned like a Facebook group to keep in touch with other members of the program would have helped so they could, you know, shout out ideas or have someone to walk with. Would you use something like that or would that be helpful for you?

Interviewee: Yes, maybe.

Facilitator: Lastly, is there anything else that you think we need to change about this program to make it more successful or anything else that you’d recommend we do to improve it?

Interviewee: Look I would just do a follow-up like on the group session. You know maybe touch base in two months. Have another group. That’s what I would do.

Facilitator: Yes and how many group sessions would be good for you?

Interviewee: Probably three.

Facilitator: Three in the year?

Interviewee: Yes.

Facilitator: More phone calls or anything like that.

Interviewee: Oh, I’m not great on the phone.

Facilitator: So, yes just three group sessions?

Interviewee: Yes.

Facilitator: Okay perfect and lastly is there anything else you think we need to know about Donald as part of our valuation that make it a unique or different town from others?

Interviewee: No. Not really, I think it’s fine. It’s a country town.

Facilitator: Okay perfect. Well that was all I wanted to ask you. Thank you so much again for your time and I just wanted to double check your address, so I can send you that gift card. What was your address again, sorry?

Interviewee: Post office box three, Donald, three, four, eight, ‘O’.

Facilitator: Perfect. Well I’ll send you that gift card later today and if there’s anything else that you think we need to know, please feel free to give us a call, but otherwise, thank you again so much for you time and we look forward to seeing you in six months in Donald.

Interviewee: Okay no problems.

Facilitator: Thanks so much for that.

Interviewee: Okay.

Facilitator: Bye now.

Participant #CD

Facilitator: So to start off with, what we’re doing is I’m comparing the different towns that we visited. We loved Donald but I wanted you to talk me a through a little bit about your thoughts about your town in terms of access to fruit and vegetables in the town.

Interviewee: We have very poor quality fruit and vege in our town so, yeah, I would say poor quality.

Facilitator: Where would you buy them from? Is there a fruit shop?

Interviewee: No, there’s only a supermarket.

Facilitator: So you have to travel outside?

Interviewee: Yeah. There’s a supermarket down in St Arnaud. That’s owned by the same people that own Donald but their quality of fruit is so much higher than up here because they’ve got to compete with Peaches down there so, yeah.

Facilitator: One woman I spoke to when we were in Donald said that, in terms of social norms, it’s the culture to buy from within the town and it’s not really acceptable to buy from other towns. Is that what you think or what’s it like?

Interviewee: I agree that’s sort of, yeah, the social norm but I’m one that shops for price and if I’m over in Horsham, I buy up big. But I’m not in Horsham very much so I have to buy locally mainly.

Facilitator: So is it about supporting the locals? Is that the mentality?

Interviewee: Yeah, it is. Yeah.

Facilitator: And what about in terms of exercise opportunities. Is there are a gym or any places to exercise?

Interviewee: I think there used to be a gym at the school but the last time I heard they were talking about closing it down due to insurance reasons because it was sort of held at the school.

Facilitator: So if you were going to be active, what kind of things can you do?

Interviewee: Walk. There’s a riding club, like, cycling club. There is a casual netball club not just competition. So that started up recently, I think since you guys were here last. We’d only just started that one up. There’s walking tracks.

Facilitator: So, overall, are you satisfied with the amount of opportunities there are to exercise?

Interviewee: Well, for me, yes, because I’m very busy so I don’t have a lot of chance to. They do have exercises and stuff but, for me, it’s the getting someone to look after my three kids so I have time to do that. Yeah, there’s not a lot of childcare around and that makes it hard.

Facilitator: And, overall, would you consider the community to be quite active or not particularly?

Interviewee: I think, in the last 12 months, it has become very active.

Facilitator: Has something changed?

Interviewee: I think just because a few more people see a few more people out and they think I could be doing that. And they see the effects of getting out and how much weight people have lost and how healthy they’ve become that, yeah, they get out and…

Facilitator: They’re getting out and being more active.

Interviewee: Yeah.

Facilitator: That’s great. When you think back to when you started this healthy lifestyle program almost six months ago now, do you remember what motivated you to join this program?

Interviewee: I’d like to lose weight, yeah.

Facilitator: Anything else? Did you come in with any other expectations to learn or to get support or was there any other…

Interviewee: Yeah, mainly just to get motivation, just bounce off other people.

Facilitator: As you remember, we had a pretty good response in that. Were you at the day time session or the evening?

Interviewee: The day time.

Facilitator: The day one. We had a pretty good response from Donald but do you think there were any factors that may have stopped other women in the town from coming along?

Interviewee: The unknown. Being in a small town, if – yeah. Usually work of mouth works really well but I hadn’t said to anyone, “Are you going? Are you going?” I just sort of rocked up.

Facilitator: Some women talked about coming to the session as being a problem because in a small town you want privacy and you don’t want to come along to a health session. And some people said it was a barrier to admit that they wanted to change. Do you think that was an issue?

Interviewee: Not for me but my personality is fairly out there and I don’t care what other people think. It could be a barrier for some people, yes.

Facilitator: And the timing of the year. We had it around December last year. Is that a busy time?

Interviewee: For us in this community, I would say yes because it’s harvest time. And usually any farmer’s wives are out there working alongside their husbands so I was just lucky that I wasn’t.

Facilitator: We did have a lot of kids at the session but childcare could have, potentially, also been a barrier.

Interviewee: Yes, definitely.

Facilitator: What about cost? Do you think that would stop a lot of people from attending if there wasn’t one but if there was one?

Interviewee: It may impact the amount of people, yeah. It probably would.

Facilitator: And in terms of when you think back to that first group session that we had in Donald, in that session we gave you that manual and we talked about healthy lifestyle in general, gave a bit of advice about that and then we also talked about behaviour strategies to change lifestyle like goal setting and we all wrote an action plan together, was any of that information new to you, do you think?

Interviewee: I think how you guys mentioned about just starting slow and change one thing first then establish that change and then go on to establish another goal once that goal had been achieved. I thought that was really good. I hadn’t really thought of doing it that way. That helped change the way I’ve looked at losing weight and stuff like that.

Facilitator: What about the information that was presented about the different – we had the six messages about soft drink, exercise, was all that familiar to you or a refresher or more new, do you think?

Interviewee: No, it was fairly familiar to me.

Facilitator: Some people said they found that quite useful and others said not so much. How was it like for you?

Interviewee: It just ingrained in me that I need to change my patterns, I suppose, yeah. Like, I knew it all but it’s just actually adopting it.

Facilitator: Yes, of course. So I guess that’s always the challenge, isn’t it?

Interviewee: Yeah, especially when you have – like my partner, his habits stem back from his childhood and he still – yeah, it’s hard for him to change and if he’s not changing then it’s hard for the whole family to change. So that makes it…

Facilitator: Yeah, that makes sense. Which habits are the ones that you think are hard for him to change?

Interviewee: Always having desert, things like that. Yeah, he has to have desert because that’s what you do and things like that. He always has.

Facilitator: So I guess it comes down to that sort of mentality.

Interviewee: Yeah, the mentality of it all.

Facilitator: And in your opinion, what do you think you’d consider a healthy lifestyle?

Interviewee: One where you’re active either playing a sport or you’re out there going for walks most days and, yeah, eating a lot of fruit and vege. That’s what I would consider a healthy lifestyle.

Facilitator: Yeah, good.

Interviewee: Not someone who exercises every single day. That, I don’t think, is a healthy lifestyle. I don’t think it’s good for the body to rigorously…

Facilitator: But just being well-rounded?

Interviewee: Yeah.

Facilitator: And in terms of your opinion about what the program was about, if you were to finish my sentence, “This healthy lifestyle program was about”, how would you finish that sentence, do you think?

Interviewee: I would say it was about encouraging women to get out there and start making goals and achieving them and getting – moving forward.

Facilitator: Had you ever done anything like this before or was anything ever like this available in Donald?

Interviewee: No.

Facilitator: No?

Interviewee: Not that I know of.

Facilitator: We’re the first to come.

Interviewee: Yeah, or the first one that was actually advertised that I knew of.

Facilitator: Do you think that’s why we probably got a positive response, because it was sort of a unique opportunity?

Interviewee: Yeah. And it was well advertised. Like, we had it almost every week in the school newsletter and things like that. It was also advertised in the paper.

Facilitator: It was great. The town, really, were very supportive and sort of took the program on board.

Interviewee: Yeah. And we now have quite a few personal trainers in town that also encourage healthier living so that sort of – yeah.

Facilitator: Is that new, those personal trainers or been there for a while?

Interviewee: There’s been, I know, two for a while but they’ve been getting more active in the community as in they’re involved in the netball and then they do other things, yeah.

Facilitator: And since being involved in this program, again, being completely honest, do you think it’s made you think a bit more about your weight, about how much you exercise or what you’ve been eating or no change?

Interviewee: Definitely a change. Every time I got a text message I’m like, have I been – it was good. It made me stop and think, well, how have I been going because especially when you slide off the bandwagon a bit and you think – but those regular text messages were good, like, yeah, I need to keep reminding myself, yeah, keep on with it, it does make change.

Facilitator: And when you mentioned sliding off the bandwagon, a lot of women have said that since starting the program, they’ve had sort of ups and downs with how they’ve gone in terms of changes they’ve made. Do you feel the same/?

Interviewee: I know, for myself, that I’ve had my son in hospital with surgeries and stuff so whilst I was in hospital, I did have trouble because I was only able to get takeaway food and things like that. Start small. If you can’t change one thing, then try to change something else. So I was making sure that I was walking up and down stairs instead of catching the lift if I’m going to be eating things like this.

Facilitator: I guess it sounds like you’re sort of doing a bit of problem solving yourself. So I can’t change this but I’ll do that instead.

Interviewee: Yeah, yeah, and it was just thinking start with something small. And that was just, well, I can walk up and down the stairs rather than – because I couldn’t go out for a run or a walk every day because I had to be beside my son. So if I did got down to get food, I could walk up and down the stairs.

Facilitator: That’s a really good strategy. In terms of the specific components of the program, you mentioned before that the text messages were useful. If we think back to all the components, so we had the text messages, the manual, not long ago you got called by someone to a bit of a phone coaching session and you set an action plan with her. And we also had the program manual. Which did you find the most useful, did you think?

Interviewee: I would say the text messages. The manual was good at the start but then it was easier to put that down and forget about it. I liked what was in the manual when I did read it and thought it was fantastic but the possibility of me picking them up and doing it every day or every week was hard.

Facilitator: A lot of women have said this as well that you used the manual possibly at the beginning but then not so much later on.

Interviewee: Yes.

Facilitator: And you said the text messages were good. What did you like about the text messages?

Interviewee: That every now and then they offered a little bit of advice but also it was just a reminder that someone was there wanting to support us if need be if we were struggling.

Facilitator: What about the phone coaching? Did you find that useful?

Interviewee: I did actually. I did enjoy that.

Facilitator: And based on all the support that’s been provided, do you think you’ve been able to make any – we’re not expecting anyone to have made any major changes but do you think you’ve been able to make any changes to your lifestyle?

Interviewee: Certainly. I eat breakfast now where I never used to. That’s a big change.

Facilitator: And how did you do that because what we’re interested in knowing is how you’ve been able to do that now versus in the past so that whatever you’ve done, we can help give advice to somebody else?

Interviewee: Well, I don’t really know how I’d do it. That was what my first goal was, to start eating breakfast. So I suppose it was just thinking, when I was getting all the kids’ breakfast, I thought, look, even if you can’t sit down and eat breakfast, put on a piece of toast and just eat that or grab a banana on your way out the door. But it was just trying to remember that I hadn’t eaten breakfast. That was a big thing.

Facilitator: Has that been sustainable or something that you did at the beginning and then lost?

Interviewee: No, I’m sustaining that.

Facilitator: So it sounds like you’ve just done it through being more aware and you said you set that as your goal and you managed to achieve it.

Interviewee: Yeah/

Facilitator: That’s great. Anything else that you’ve changed? You mentioned before about doing a bit more incidental activity by walking up the stairs.

Interviewee: I have tried making sure that if I have a spare hour to go for a walk, and for the last four weeks I’ve been able to successfully do that at least two or three times a week, and also been riding my bike for 20 minutes at night. Only 20 minutes but, yeah.

Facilitator: It’s all good.

Interviewee: I’m an emotional eater and I know I am and if I start eating more I don’t exercise because I’m usually feeling down so that’s sort of a lose – lose situation for me.

Facilitator: But it sounds like you’ve been doing quite a bit more exercise. Is that correct?

Interviewee: Yes, that’s definitely correct.

Facilitator: Has that been since starting this program or something you were already doing before starting the program?

Interviewee: No, since starting the program.

Facilitator: That’s great. And, again, I don’t mean to pry but what do you think has made you be able to do more exercise now than previous attempts in the past?

Interviewee: I suppose it would be motivation. And also knowing some of the other ladies in the program have lost weight, a lot of weight, very noticeable weight that, yeah, I think that’s been a bit of motivation, knowing that you can do it but you’ve got to do a little bit at a time.

Facilitator: So would you say it was fair to say that you’ve been able to make these changes through the support of the other women in the program and the support of this program?

Interviewee: Yes, definitely.

Facilitator: That’s excellent to hear. And in terms of motivation, you mentioned before that you’ve had some problems with emotional eating and it has sometimes fluctuated but, overall, would you say that motivation to be healthy has been pretty sustainable or fluctuated?

Interviewee: Sorry, say that again?

Facilitator: Sorry, the motivation to change, to eat breakfast, to exercise and to eat better, has that been sustainable or has that gone up and down, do you think?

Interviewee: It has gone up and down but, generally, a downward trend. A downward trend but there has been ups and downs.

Facilitator: So, overall, motivation has…

Interviewee: Picked up.

Facilitator: Picked up?

Interviewee: Yeah. The motivation has picked up but my weight, I’m meaning it was a downward trend.

Facilitator: The downward trend as in it’s gone up and then gone back down?

Interviewee: Yeah.

Facilitator: Do you feel happy with how you’ve done so far in this program or are you satisfied?

Interviewee: A little bit. I would have hoped that I could have maintained a more, I suppose, what’s the word, keep on to it more every day, thinking about it, but I haven’t.

Facilitator: And don’t feel alone, a lot of women have said that. And some women suggested that in order for them to keep their motivation up, they need a bit more support. Do you think that would have been useful for you?

Interviewee: Yeah, probably it would have, yeah/

Facilitator: And some women said they liked text messages or they wanted more personal contact. What would have worked best for you do you think?

Interviewee: Probably a personal contact probably would have worked out a lot more, yeah.

Facilitator: And when you say a lot more – sorry, again – is that sort of once a fortnight, once a month, once a week? What’s good for you, do you think?

Interviewee: Probably a once a week would have, yeah, really, really helped. Once a fortnight would have been okay. Yeah.

Facilitator: And also one lady mentioned that she thought a face-to-face at a midway point, like, six months, would have been useful. Would that have been useful to you, do you think?

Interviewee: Yeah, I think it would have been probably good. Like, even just have another – like a session like you guys did have and just where someone would say, “How have you been going? What have you been struggling with and how do you think you could help that?” sort of thing, “How do you think you could change that?” would have probably been really good, yeah.

Facilitator: And in terms of social support, I know you mentioned before that it’s a bit difficult with your partner’s mentality around desert and things but do you feel adequately supported by your family?

Interviewee: Yes, adequately. Yeah. I don’t eat desert, I never have and he offers me desert but he knows that I’m most likely not going to eat it but he encourages, if I want to, like, go for a bike ride, he encourages me. But he hasn’t been home for a while. He hasn’t even been home to look after the kids for me to do that.

Facilitator: So in terms of barriers to making changes, would you say your major barriers would be – it sounds like time could be one?

Interviewee: Yes.

Facilitator: What are the other things that stand out to you, do you think?

Interviewee: It’d be child care as I’ve tried picking up during the day going for walks, even though that’s hard because my husband always wants me to be doing work, being a farmer’s wife, during the day. It makes it hard/

Facilitator: So work commitments?

Interviewee: Yeah.

Facilitator: Would you say support could be a potential barrier or not?

Interviewee: No, I don’t think it would be a barrier.

Facilitator: And you also mentioned before motivation?

Interviewee: Yeah, that’s a big barrier.

Facilitator: So additional support could help motivate you. Is there anything else that you think would help motivate you more, do you think?

Interviewee: I don’t know. I was going to say, if I had access to a cheap, relatively cheap, personal trainer but a personal trainer that’s not stick thin. A person that’s healthy living and someone to just walk along side you once a week and just see how you’re going sort of thing. Being a slightly bigger person, it makes it frustrating to see someone so skinny but because they work out every single day and that, to me, is impossible, at the moment, to do that.

Facilitator: And, again, in terms of things that would motivate you to live a healthy lifestyle, what are the major things that stand out to you?

Interviewee: Health would be a big thing. Me, I’ve been suffering lower back and stuff like that so that’s been a big thing for me to actually go out and walk and stuff because it’s better for me. So health would be one of them.

Facilitator: Anything else? Someone mentioned to be role modelling for their kids and helping set examples for their children and being able to keep up with them would be something that’s important to them. Is that something that motivates you?

Interviewee: No, I think it would be being a role model for my husband more than anything. My children are very active so I’m not worried about them and they are very healthy eaters, always have been.

Facilitator: Another thing they are looking at is about whether this program had any influence on anyone outside the people that were in that room, in that Donald session at the school. Do you think you’ve talked more about healthy eating or changed anything that you’ve done that’s affected your family?

Interviewee: Yes, definitely. Well, my husband started seeing me going for walks and losing a little bit of weight. He’s started bike riding with the bike riding club because he loves bike riding.

Facilitator: So that’s motivated him, do you think?

Interviewee: Yeah, it has to a point. He knows he needs to lose weight and he wants to lose weight. Of course, his factor is time again and work commitments so it makes it hard.

Facilitator: And what your children? Have you changed anything arhat you’ve bought or cooked in the house or that’s been pretty consistent?

Interviewee: No, we’ve bought a heap more fruit and expected them to eat more fruit than maybe having a bickie and cheese or something as in a more processed sort of food, your whole fresh fruit and vege. And, yeah, probably also less soft drink. We don’t drink as much soft drink as we used to.

Facilitator: It sounds like you’ve made some really good changes. And the next thing I was just wanting to look at is – it sounds like you’ve had a big influence on your family but do you think you’ve had any influence on anyone outside your family like any friends or anyone that you work with or anyone outside?

Interviewee: No, I wouldn’t think I have.

Facilitator: And some people have said that it’s because they don’t usually talk about health with their friends or with anyone outside of their family. Is that similar for you?

Interviewee: I do talk about health with my friends but most of my friends are very active so that’s why, it’s because they’re more active than me, I think. They’re more motivating me to get active.

Facilitator: And have they been a good support for you do you think?

Interviewee: Definitely.

Facilitator: Do you go walking with them or do exercise with them?

Interviewee: No, I don’t.

Facilitator: But it’s still good that they’re encouraging you.

Interviewee: Yeah. I think they’ve encouraged me to come along to weigh training and things like that but, of course, I haven’t been able to get there.

Facilitator: And the childcare and issues like that are something that’s been a consistent theme that a lot of women from Donald have said so it does sound like it’s a big barrier.

Interviewee: It is.

Facilitator: And the last question I have for you is based on your experience, do you think you’d recommend this program to anyone else in your community?

Interviewee: Yes, I would. I think it’s been great. Even if you just get you heading in the right direction, yeah, fantastic.

Facilitator: And that’s what a lot of people have said, it’s been beneficial to start your motivation.

Interviewee: Yeah, it’s a kick start without – like, people do the whole silly soup diets and that to try and kick start their motivation and they always seem to pile the weight back on but I think this has been great because even though I may not have lost a lot of weight, I have lost weight and I am continuing to lose weight only through the slight changes that we’ve made. So just like you slowly put on weight, if you slowly lose weight, you’re more likely to keep it off.

Facilitator: Yes, I agree.

Interviewee: That’s fantastic.

Facilitator: Some people said they’d recommend this program to people that have had less experience with health or some people said they’d recommend this program to people that were more overweight or less overweight. Anyone in particular you’d recommend it to?

Interviewee: People who I know have been yo-yo dieters, yeah, I would definitely recommend.

Facilitator: And sorry to keep going on about this but you mentioned before about the facilitator, you like to have someone that has - not necessarily a stick thin person or is on the same path as you.

Interviewee: Yeah.

Facilitator: Does it make any difference to you whether that facilitator is from Melbourne or is a local person?

Interviewee: I don’t know. It depends on the personality, I suppose, of the person. If they’re from Melbourne and have a personality that I agree with, yeah, that wouldn’t bother me but, yeah, probably a local person might be a bit evasive for some people in the community. They might think, this person might be judging me whereas someone who comes and goes, they’re not too worried about.

Facilitator: Is there any traits that you’d recommend that you liked about the facilitator or you’d recommend that we need to look for to facilitate this program?

Interviewee: Just, I think, a great communicator and someone who’s very encouraging would be a good thing.

Facilitator: Were you happy with the facilitator?

Interviewee: Yes.

Facilitator: What about the venue?

Interviewee: I think it was very appropriate, having it at the school because the school is a big part of our community. I think it may have hindered some other people from the other primary school. They may have thought it was just for the Donald Primary School, the meeting, so they may not have come because of that.

Facilitator: Is that the Catholic school?

Interviewee: Yeah.

Facilitator: And some women said, not in Donald but in another community, that the Catholic school are less likely to come to the primary school but the primary school might go to the Catholic school.

Interviewee: Yeah/

Facilitator: Is that similar in Donald?

Interviewee: I would say that’s probably fairly true.

Facilitator: And one woman recommended, yeah, if you want to get more women, have them at the different schools.

Interviewee: Yeah. When you think about it, a lot of people send their kids to the Catholic schools because they’re a bit toffy. They think it’s a better education. Not necessarily but they think it is.

Facilitator: The primary school looked fantastic and the principal was amazing.

Interviewee: I’ve done teaching round at both the Catholic and the primary and I would go to the primary any day.

Facilitator: And what about in terms of the level of information provided? Was that enough for you or did you need further or more specific information, do you think?

Interviewee: No, I think the level of information was great, yeah.

Facilitator: And we already touched on this before but you said in terms of the support provided by the research team that, ideally, it would be best to have a bit more contact.

Interviewee: Yeah, I’d agree with that.

Facilitator: So would you say if I asked you if you were satisfied with the contact, would you say yes or no, do you think?

Interviewee: Probably in-between.

Facilitator: So in-between but in order for you to make more changes and be more motivated, more contact?

Interviewee: More contact, yes.

Facilitator: And the last question, I swear, is there anything that you’d recommend to us that you think we need to change in the future to make this program more successful?

Interviewee: No, I don’t think so.

Facilitator: No?

Interviewee: No.

Facilitator: Nothing else we need to know about Donald?

Interviewee: No, I don’t think so. Sorry, mind’s had a blank.

Facilitator: No, sorry, I’ve talked your ear off.

Interviewee: That’s all right. I’ll probably get off the phone and think, oh, yeah, you could do this.

Facilitator: Yeah. And if you do think of anything, please, you’ve got our number so give us a call. We’re happy to hear any suggestions, that’s the whole point of the evaluation is making it better for people in the future. So, please, if you have anything, send us a message, an email, however best that you like to contact us.

End of transcript

Participant_#HS

Facilitator: And just to let you know in the letter that we sent you a while back I’m just wondering if it’s okay if we tape record the interview just to make sure that we accurately get down everything you say?

Interviewee: Yes, no worries.

Facilitator: Okay perfect. So to start off with I’m starting easy. I just want you to tell me a little bit about the access to fruit and vegetables in Beechworth?

Interviewee: Well it’s reasonably good. You’ve got the major supermarket. Quality can be another thing I suppose and there also is the little fruit and veggie shop which is sort of like a specialised – well not specialised but you know they probably sell maybe a bit better quality or more local produce. Then of course you’ve got options of you know Wodonga, Myrtleford, Wang, those sort of things. So I’d say I mean it’s pretty good.

Facilitator: What about in terms of exercise opportunities? Is there many options available?

Interviewee: Oh it’s limited only by your inspiration I suppose or your desire.

Facilitator: So there’s lots of things to do if you’re willing?

Interviewee: Absolutely yes. Yes and probably more so if you’re in town a bit but then again you know you can get other people you know to do it with you or it depends yes how driven you are I think also yes.

Facilitator: Yes and would you consider the town to be one that you know there’s lots of social connections and the community are quite – you know are quite a friendly …

Interviewee: Yes I think so. Yes.

Facilitator: And has there ever been a healthy lifestyle program like this available before that you’ve been aware of or that you’ve been involved in?

Interviewee: Not that I know of no except your – you know your advertised programs that cost lots of money that people don’t have.

Facilitator: Yes. Have you been in a program before?

Interviewee: No.

Facilitator: No and if you think back to when we came to Beechworth in about March earlier this year, do you remember what motivated you to come along to this program?

Interviewee: Well to see basically what sort of resources or what sort of things would inspire and help me to keep going along my path I suppose.

Facilitator: Yes and what’s your path?

Interviewee: Just you know to be a bit fitter, lose a bit of weight and activity I suppose yes and eating like healthy meals and all that sort of – having that sort of dietary – appropriate dietary. Not necessarily inappropriate.

Facilitator: Yes and do you think there were many factors that may have stopped other people from coming along on that day to that session in Beechworth?

Interviewee: Look there probably was you know a lot of mums work and some might not have heard about it. Yes I mean yes.

Facilitator: If we think back to that first session, we had that big group session when we gave a bit of healthy lifestyle advice. In that session we talked about sort of general healthy life advice. We had those six messages you know about reducing soft drink, eating breakfast and then we talked about problem solving and goal setting and you know strategies to help achieve behaviour change …

Interviewee: Yes.

Facilitator: Was any of that information new to you or mainly quite familiar do you think?

Interviewee: No. I think it’s just some motivation and desire to put it into practice or the you know the constant reminder or you know the group support the you know let’s keep it going or have you been doing this sort of thing so …

Facilitator: And you’ve mentioned motivation, what’s that like for you?

Interviewee: Pretty high when we’ve got you know we’ve got our family. We’re not having any more and you know there’s no more excuses not to you know just sort of lounge around well not that you lounge around but you know not to be more physically active and fitter because the kids need fit, well reasonably fit parents, if they want to be involved with you know we’ve always – the kids – the youngest one’s just learning to ride bikes so you know we go for bike rides and go for walks or go for a few other things yes.

Facilitator: So I guess is it fair to say that what’s driving you is to be there for your children and to improve your health? Is that what – is that why being fit’s important to you?

Interviewee: And for my own – and for me you know it was before when the girls were little you didn’t have time to dedicate to yourself and I enjoy you know my bike riding, my walking, running and my circuit stuff and that’s me time if you know what I mean. Yes so …

Facilitator: And what’s …

Interviewee: Yes.

Facilitator: Are there any obvious things that are obstacles that you’ve found that make it hard to be as active or to eat as well as you’d like to?

Interviewee: Time and money. The old – the same old same old really. Time and money. Yes.

Facilitator: Yes and just to, sorry, just to clarify it sounds like a lot of what we talked about at the session was quite familiar to you. Is that correct?

Interviewee: I think so yes. We’re all pretty much in the same boat.

Facilitator: Yes so that group support was useful in learning that you were all in the same boat?

Interviewee: Yes and I believe that for me, something like the group thing, an ongoing sort of program like that would be far more beneficial support wise and just community wise and all that sort of thing so I think yes rather than we sort of just disbanded and it hadn’t really gone anywhere and I suppose it’s probably up to us as well to sort of I don’t know let’s get together blah, blah, blah but yes it just didn’t translate to that sort of way so …

Facilitator: Yes and you’re not alone in saying that. A lot of people have said that. Would that be helpful for you to have that ongoing regular group meeting?

Interviewee: I think so or you know it doesn’t have to be a meeting it can just be a catch up oh you know, let’s go you know I don’t know we can do something or somewhere all the kids can go because most of us have all got kids. Somewhere that doesn’t have to be during the week because most of us work and we’re you know on short timelines, that sort of thing. So yes I mean there’s so many factors to consider. You can’t have them all you know covered but yes.

Facilitator: Yes so to have a bit of that support from the whole – from the other women in the program?

Interviewee: Absolutely yes. Yes.

Facilitator: Yes and if you were to describe the program or if you were to think about what the take-home message of sorry – was from this program what do you think that was?

Interviewee: Aim to be healthy. Aim – yes. Aim for longevity of life and sort of be conscious about your choices.

Facilitator: Yes and when you mentioned being conscious of your choices, do you think by again by being completely honest because we want as much feedback as we can get, but do you think by being involved in this program it’s made you think more or less or no change in what you’re eating, about your weight, about your exercising and has it had any influence?

Interviewee: It’s probably like a nagging thought if you know what I mean. It’s not necessarily you’ve actually done – made any changes but it’s there that you know you should or that sort of thing so I’d say that’s probably the main impact it’s had on me and you can say that that sort of nagging thought will eventually sink in and help – up on the bench sweetheart – might actually on that particular day you’ll go no, no I’m going to follow that nagging thought or you know I’m going to start no it’s right sort of thing.

Facilitator: Yes so would it be fair to say, a couple of women I’ve spoken to said that the program’s made them more aware of what they should do but they’ve sort of struggled potentially with that activating the change? Is that something that you can associate with?

Interviewee: Yes because one of my goals was to eat more fruit and I can’t necessarily say that I’ve done that but I guess – mummy’s on the phone sweetheart so if you go over there and have a look. Thanks babe - ask dad. Where was I going with that? Yes.

Facilitator: You were talking about fruit so you said you’ve been thinking about it?

Interviewee: Yes what my goal was and yes you know it’s probably just been in the last couple of weeks that I probably have actually increase and reduced – you know increased the fruit or whatever and reduced the crap so to speak so I mean it’s probably been what was it, March you said? So but that nagging little thought you know obviously it being there and it’s taken a while to root itself in my brain but yes you know yes.

Facilitator: Yes but now it’s kicked in?

Interviewee: Yes it’s slowly – it’s still a gradual process. A slow, gradual process so yes any change I suppose is better than none.

Facilitator: Yes and in terms of the program itself, we had as – we had different components. We obviously had that group session back in Beechworth at the school then we had – we gave you a program manual – you’ve had some SMS text messages …

Interviewee: Yes.

Facilitator: The website and also someone called you not so long ago just to touch base and to help set some goals with you on the phone so sort of a bit of a phone coaching session.

Interviewee: Yes.

Facilitator: Did anything stand out as being more useful to you or the most useful?

Interviewee: The most useful? I suppose the text messages were good because it was just like a small prompt but the manual, I think I flicked through it the afternoon I come home and haven’t touched it since. Yes but I think yes the regular interaction with the people that are going through it as well would definitely be far more motivating and inspiring than yes – but again the text messages sort of kept it in your memory I suppose and you sort of let it fade into the background into like it never happens.

Facilitator: Yes so it sounds like the face to face is the best source of contact for you. Is that correct?

Interviewee: Yes I think so definitely.

Facilitator: What about the phone call? Did you find that useful or is that something that if …

Interviewee: Oh I think it just seemed to reiterate what we’ve been over and you know I didn’t really find it very helpful at all. It just yes it didn’t change anything anyway.

Facilitator: Yes so what’s – I know this can be a little bit of a hard question, but what’s made you change in the last couple of weeks versus back in March or previously do you think?

Interviewee: Drive and desire. I think you just get to a stage where you go that is it. I’ve had enough and I’m just doing it.

Facilitator: Yes and I know – I don’t mean to pry …

Interviewee: No that’s okay.

Facilitator: But did something happen? I mean – what’s – why now? Like what’s it …

Interviewee: I don’t know. Don’t know oh well I suppose I’ve been you know increased physical activity. I haven’t necessarily changed dietary habits and I guess I’ve – oh probably a while ago come to the realisation that if you don’t change - like I’ve come so far with physical activity so if I want to improve say weight or whatever any more then it’s going to have to incorporate dietary components so okay so it’s like suck it up princess, here we go.

Facilitator: Yes so it sounds like you – the exercise has been something you’ve always been in – you’ve been in control of for a while is that correct?

Interviewee: Yes well that’s been – it’s always been but then the kids came along and then you don’t get a chance and probably this is my third year so I’ve been you know it was every second day, now it’s three days a week sort of thing so we try to maintain that but to – you can – the results to go further it needs to be complimented by dietary changes and that sort of thing so …

Facilitator: And has that motivation – so is it coming from then you or is it something else?

Interviewee: Yes. Well no one else is going to do it for me.

Facilitator: Yes and just to – I mean you touched on this a minute ago but based on – since attending this program are there any specific changes that you’ve made? You mentioned that you’ve increased your fruit and you’ve reduced the junk. Is that a result of the program or is that just a result of you?

Interviewee: Oh it probably can be attributed to that. I think it’s just you know you get to the stage where you know you think I don’t know but yes. Well if you want to achieve something, these are the steps you’ve got to take and are you prepared to do it? Yes, okay. When – okay now. So …

Facilitator: Yes so it’s yes. From …

Interviewee: We all know what we need to do but it’s just a matter of doing it. Being driven enough to you know want it and all that sort of thing and have others who are in similar situations go yes we’re doing it and it’s like yes, cool, okay I’m in. So it’s like a bit of group therapy.

Facilitator: Yes and have you got others, sorry, at the moment now trying to do something as well?

Interviewee: Oh well when I first started say three years ago, I was doing my exercise program I suppose you’d call it, we’ll call it a program, by myself and then I’ve had friends come and go but I’ve had a friend who’s and been doing it probably this is her second year I think and then there’s a group of us that do once a week and then anyone you know in between. They do whatever in between sort of thing so yes.

Facilitator: Is it an exercise program you’re talking about?

Interviewee: No it’s just well no not really. It’s just being active and in the Herald Sun they used to have the Body and Soul section on Sundays and they’d have like a circuit program so we just go to the stage where we’d take bits and pieces of that and we’d do that three or four days a week and now we’ve just got to a certain stage where now it’s just three so yes but that does – there’s not just circuit it’s walking or interval, running, that sort of stuff so …

Facilitator: Yes and I’ve touched on this and I keep repeating myself but …

Interviewee: Yes.

Facilitator: So since being in the program do you think you’ve made any changes that have been sustainable or not so much?

Interviewee: Well I hope to sustain the fruit intake.

Facilitator: From now?

Interviewee: Oh yes and you know you I guess you hear people that once they make up their mind then you know, they’re quite happy to stick to it. They might sort of waiver here and there but you know they’re – essentially that’s what they want to do so that’s what they’ll do.

Facilitator: Yes and the three types of – and sorry, the exercise three times a week, that was happening since before you were in the program?

Interviewee: Yes.

Facilitator: Okay so mainly the biggest change sorry is the increase of fruit and the reduced junk food?

Interviewee: Yes.

Facilitator: Great and is there any other factors? You mentioned that your friends have helped you know with the exercise. You mentioned that your drive has helped to get that fruit in. Anything else?

Interviewee: Yes. No that’s pretty much the core elements I think yes.

Facilitator: And in terms of those behaviour change strategies like we talked at the session about goal settings, problem solving, have you used any of that to help get that fruit in or is it just something that you just did?

Interviewee: It’s probably just the goals you know and yes you know what steps you need to take so yes.

Facilitator:	Yes and in terms of what we’ve been looking at with everyone is about the reach of this program so we’re trying to work out by if you – by you being involved in this program, whether it’s had any influence on your family, your children, your partner. Do you think that by you being in this program has had any flow-on effects?

Interviewee:	I think it would probably most definitely because if you don’t – if you’re changing your dietary then you’re also I mean being the shopper and cooker and all that sort of stuff, then if it’s yes you direct or guide I suppose what is consumed and what isn’t consumed; quantities and all that sort of thing. So yes I think to make you more conscious of yes what you’re bringing in and what you’re putting out on the table and that sort of thing. So what you’re allowing them to consume.

Facilitator:	Yes so any …

Interviewee:	And increasing their activity as well.

Facilitator:	Any examples of how you’ve influenced your family since being in this program?

Interviewee:	Well I suppose the increased activity I mean we’ve always been a fairly active sort of family like the bike riding and the walking and you know that sort of sporty type things and the kids are involved in and that sort of stuff and I suppose shopping wise you focus – you don’t tend to buy so much of the – I mean I tend to cook at home a lot anyway because all the stuff’s too expensive but you know being aware that okay well this is allocated for this week. It’s not just for the next two days, it’s got to do the week sort of thing you know. Yes.

Facilitator:	Yes but sorry because it sounds like that most of the changes you are doing in terms of the exercise that was something that hasn’t really been influenced by the program? Correct?

Interviewee:	Oh but it can be more inspiring to know that you know that and that’s what it’s all about too I think you know you’ve got the idea and the concept but without the drive and the reminder and the you know that sort of thing that can sort of just sort of disappear as quickly as it came if you know what I mean.

Facilitator:	Yes so it’s helped sustain it for you perhaps?

Interviewee:	Yes. Every little bit helps.

Facilitator:	Yes and have you spoken to anybody else in the program about how you’ve been going or have you found any support from anybody else in the program?

Interviewee:	Oh look there’s the odd conversation as you’re walking past, in between dropping off and doing whatever and that sort of thing but as to you know a full on conversation or you know a D and M about it, no. .

Facilitator:	No? That’s okay and do you feel supported by your family or have they helped to make you make changes?

Interviewee:	Yes. They like going for bike rides.

Facilitator:	Good so they’re all – everyone’s getting in together?

Interviewee:	Oh yes, yes.

Facilitator:	Yes and do you think by you being in this program, again, it’s just a low intensity program so we’re not expecting any large changes but do you think you’ve had any influence on any of your other friends outside the program or in the program?

Interviewee:	Maybe a marginal but yes I mean yes we just do what we do so yes activity wise and that I mean we’re always encouraging people with you know friends or whoever to come along and do it with us. We’re sort of not skill based if you know what I mean. You’re not going to have a certain level of fitness to come along with us like you’ve got to yes …

Facilitator:	Yes and since being in this program how do you feel about your progress? Do you feel you’ve done well, you’d like to do more, how are you feeling about …

Interviewee:	I’d like to have done better. I don’t think I’ve done – I haven’t been a scholar student that’s for sure. Probably the grade that could use more effort that’s what I’d put myself at.

Facilitator:	Yes. What about your weight, have you been weighing yourself?

Interviewee:	But in saying that you know …

Facilitator:	Have you been weighing yourself?

Interviewee:	You know, hey?

Facilitator:	Sorry I missed what you just said.

Interviewee:	Oh I just I yes put in some more effort, just saying that you know towards the last couple of weeks it has been increased consciousness about it all and it’s like right. Roll up your sleeves, let’s go.

Facilitator:	That’s good to hear.

Interviewee:	Yes.

Facilitator:	And have you been weighing yourself or have you found that helpful or not helpful?

Interviewee:	No. Well I don’t really have access to a set of scales and I’d probably be on there every second day if I did so I usually just go off to the doctor’s surgery and jump on when I’m out there or around and that sort of thing. So I probably am keen to do that next week or so only because yes, yes that’s where I’m at but you know you feel you know – and it doesn’t necessarily – I think that can be sort of a negative thing, whether you see a change or you don’t see a change and I think you can tell when you’re putting your clothes on or you know that because you haven’t been having that you’re doing all right anyway.

Facilitator:	Yes and in terms of the last thing I wanted to ask you was about your satisfaction with this program so based on being involved in this program do you think that it’s something that you’d recommend to other people in the community or to any other – any of your other friends?

Interviewee:	I think if there were maybe some changes like a group you know catching up and I think the text messages are pretty good. You sort of go, oh God, them again but you know like I say, it does prompt you into keeping that in your memory bank if you know what I mean. Yes the booklet a complete and utter waste of time for me because I didn’t read it.

Facilitator:	Is it …

Interviewee:	Yes I mean I think a more person to person approach and it won’t I mean if you guys were the ones to get the ball rolling and then let the group like generate the momentum if you know what I mean, we can be the you know like SMS support and then maybe coming up every three months or something and address the group or you know. I don’t know do something like that but yes it yes.

Facilitator:	One lady mentioned that she thought like a group Facebook would be ideal because then she could keep in touch with other women in the program and organise a few you know like you mentioned that you do your program in the park. You know you could get people …

Interviewee:	Yes.

Facilitator:	Other people to come along. Is that something you think would be useful?

Interviewee:	Not for me because I’m not on Facebook.

Facilitator:	No.

Interviewee:	A complete an utter waste of time. I mean it’s good – it’s a good medium don’t get me wrong but yes, I just – I don’t have time and yes I’ve got better things to do then sit on the computer and …

Facilitator:	Yes but that kind of idea of keeping in touch with everyone and meeting up as a group would appeal to you?

Interviewee:	Definitely.

Facilitator:	Okay perfect and in terms of that facilitator, do you have any preference whether she’s a local lady or a lady from Melbourne? Any …

Interviewee:	Well I guess if it was a local person you’d have to sort of maybe see who it was. Yes it depends who it was. Yes.

Facilitator:	What about the venue? Any – was that an okay venue at the school or …

Interviewee:	Outdoor, indoors, either, either. It could be catching up before a walk around the gorge or you know blah, blah, blah.

Facilitator:	That could have been a good idea. A group walk prior – before the session and what about group versus individual? Do you have any preference of how you’d like to receive your support?

Interviewee:	What do you mean by that sorry?

Facilitator:	Would you prefer if that session was a one on one or did you like the group or …

Interviewee:	Oh no, no one in all in, I say yes.

Facilitator:	So the group?

Interviewee:	Yes.

Facilitator:	Yes and in terms of the information provided, was that okay or did you want more information?

Interviewee:	I don’t know. I was up at the kinder today and they’ve got this program going and it’s all about healthy eating and stuff like that and it’s just - it’s visualising what it is that you should be doing. You know like we’re talking about healthy eating and stuff like that and it’s like yes that’s good and you can read a chart until the cows come home but until you can visually quantify that okay there’s x amount of fruit there on the table, there’s and you can distribute that for this time and this time during the day and there’s that particular fruit or you know it’s all about visually quantifying it for me as well, to help improve the – and yes and that was funny that you – yes you’re doing this questionnaire today because it was yes quite a valuable exercise up there for the kinder so yes.

Facilitator:	Yes so it sounds like you’re a bit of a visual learner?

Interviewee:	Absolutely yes, yes and you know visual quantifying.

Facilitator:	So you want – you know eat three pieces of fruit or eat two so that kind of learning’s good for you?

Interviewee:	Yes and varied combination as well you know it’s like it’s all yes. It’s all about going back to grass roots for us mums because you know we learnt from you know our parents and they’ve learnt from their parents and it’s a hard nut to crack to try and you know alter learnt, engrained habits shall we say yes.

Facilitator:	Are there some of those engrained habits that you want to change?

Interviewee:	Yes, yes but it’s all about knowledge I think and you know like I said, being able to visually quantify it and say okay, well this is good, this is appropriate, this is adequate, anything over and above that is you know, is not necessary and not that good for you blah, blah, blah so …

Facilitator:	Yes and in terms of you mentioned that you wanted more support, how – and you said that you prefer it sort of face to face in terms of the group. How often would you have liked that group to meet up?

Interviewee:	Oh once a month?

Facilitator:	Once a month? Yes.

Interviewee:	Yes minimum. I mean you don’t want to get sick of each other.

Facilitator:	Of course and in terms of your expectations, again, being honest, do you think by being involved in this were your expectations met or did you have any other different …

Interviewee:	No.

Facilitator:	Expectations of what you wanted to achieve or …

Interviewee:	Yes I’d say they probably weren’t met but they were probably different but as to what exactly it was I don’t know. Yes because I just wanted to participate and see where – I guess where it took me I suppose so …

Facilitator:	Yes but you’re not exactly sure what you were looking for?

Interviewee:	Whatever it was I didn’t necessarily get it but you know in saying that, I got that constant reminder to be more conscious of being healthy and you know it’s taken a few months but you know something has clicked and it has sort of had an impact and you know it’s all interlinked. You know you’ve got to have you know a sort of component of each of it going around to sort of yes get you going.

Facilitator:	Yes and last question, is there anything else that you would recommend that we do to help improve this program in the future?

Interviewee:	I think just encourage them to – the participants to sort of interact. You know and you might find that you have okay you’ve got your once a month meeting sort of thing but then you know some might be friends so they catch up once a week or some might become friends and catch up you know here, there, more frequently, that sort of thing so yes I think that yes that sort of thing would be far more beneficial for a lot of people I think yes.

Facilitator:	Yes and one person said that yes, they were quite happy for the group to catch up without the university you know, without the facilitators.

Interviewee:	Yes.

Facilitator:	Would you be happy with that or would you prefer the facilitators?

Interviewee:	Oh yes because it would be something that would develop itself you know. You’re sort of given the drive and the tools or whatever or you know the resource could contact you or for you to email or do whatever, SMS, but yes so it’s you know you can hold your hands – hold people’s hands but sometimes it’s to a certain extent. But you know ultimately it’s up to the individual or whatever their reasons are or whatever.

Facilitator:	Yes so maybe yes get the – just get the participants to meet up themselves and one person also suggested you know having someone from the local health centre involved. Is that something that you think would be okay or …

Interviewee:	Yes absolutely yes.

Facilitator:	Okay perfect. Is there anything else that you think we should capture in our evaluation or anything else you wanted to add?

Interviewee:	No I think that’s pretty much – yes. Pretty much covers it. Yes I think the group thing’s very important, the SMSs are good, yes and yes I think it’s a worthwhile thing to be doing but yes as you do more and as you get more feedback in sort of the – improved and streamlined and all that sort of thing so it does have more of an impact. Yes because there are lots of people out there that aren’t necessarily involved but you know maybe next time round would be and or wished they had have been and that sort of thing so yes you constantly sort of being around gives people food for thought as to like, maybe I should do it this time. Or you know maybe it would be a good idea or you know …

Facilitator:	So maybe holding it again could be useful at another time to get people involved?

Interviewee:	Yes, yes absolutely and then promoting the fact that you have been here and I mean probably like varied results like I’d probably say, yes have a go but someone who really went the full nine yards with it who has dramatically changed and has proved out of sight would absolutely positively rave about it so your intake would be higher again so …

Facilitator:	So it sounds like that word of mouth is very important?

Interviewee:	Oh in a rural setting absolutely yes, absolutely.

Facilitator:	Any other ways to get more people involved?

Interviewee:	If you were to advertise like if you were to – in school newsletters, kinder newsletters, localise it you know because yes that’s a very important medium of communication. Parents who drop off and pick up and don’t get to read the newsletter on the email or just pick it out of the kid’s bag and just flick through it while they’re having a cup of tea before they start the next round of jobs to do and you know when they get home from work or you know they get to catch up on at the weekend sort of thing yes.

Facilitator:	Okay and this is actually the last question. I just wanted you to confirm your address if you could please, just so that we could – can send you that gift card. What was your address again sorry?

Interviewee:	Oh okay yes.

Facilitator:	Is it 18 Mellish …

Interviewee:	18 - 18 Mellish.

Facilitator:	It’s double L sorry?

Interviewee:	M-E-L-L-I-S-H.

Facilitator:	Okay perfect. Well thank you …

Interviewee:	Oh Brian I can’t hear you. Sorry, my husband’s having his input now.

Facilitator:	Sorry. Any feedback’s welcome.

Interviewee:	No. He wants to know if you want him – if you want to talk to him. She doesn’t want to talk to you. Go away.

Facilitator:	We’re happy to talk to him if he’s got something he wants to add but …

Interviewee:	Yes. No. Nothing constructive I don’t think.

Facilitator:	But thank you again so much for your time …

Interviewee:	No worries.

Facilitator:	And I’m really glad to hear that your motivation’s picked up a bit because that’s really good to hear but if you’re requiring any more support or you want more tips or help, please just give us a call and we’re happy to talk to you. We’re always here.

Interviewee:	Yes. No worries. Well you guys are coming up …

Facilitator:	We’re coming in March, back again at the end of the year.

Interviewee:	Oh but in 12 months from the date yes okay cool.

Facilitator:	Yes from the date and …

Interviewee:	We’ll …

Facilitator:	Yes besides that …

Interviewee:	…especially with all your SMSs and things and yes we’ll probably catch you in March then.

Facilitator:	Yes. We look forward to seeing you in March and thank you again for your time and if you don’t receive that gift card in the next week or so, please let us know.

Interviewee:	No worries.

Facilitator:	Perfect. Thank you so much again.

Facilitator:	Thank you.

Interviewee:	Okay bye.

Facilitator:	Bye.

End of transcript

Participant #JG

Facilitator:	Thank you so much for sending back the form and we really appreciate your commitment and participation, it’s much appreciated.

Interviewee:	Yes, no that’s fine.

Facilitator:	So the point of the evaluation is really just to have an informal chat about how you’ve gone about the program. What we’re doing is we’re trying to look to the future to work out how we can improve this program in the future and what needs to change.

Interviewee:	Okay.

Facilitator:	So to start off with, what we’re doing is we’re comparing the different communities that we went to. We went to 42 communities as you may remember and I was hoping you could tell me a little bit about access to fruit and vegetables in Beechworth.

Interviewee:	It’s okay. We have an IGA which is very limited in their fruit and veggies and we usually shop at Coles in Myrtleford, which is further away, only because they’ve got a greater range and their often a lot cheaper. We also have little green grocers in Beechworth but they’re very limited and it’s often organic but it’s very small amounts and quite expensive too.

Facilitator:	What about exercise opportunities? Are you satisfied with what’s available?

Interviewee:	Well we’re in Stanley which we’re 10 minutes out of Beechworth so I used to be a member of the gym and I found that when – as actually my membership was finishing up and they had just introduced, it’s not a 24 hour gym, but longer hours, like unsupervised which I thought was great but I still have to drive into Beechworth. I know it’s not far but it’s a dark road, it’s a 10 minutes of you know country road, no lights, so I just found that the 10 minutes does make a big difference to get into town and to go to the gym. So out here it’s really any exercise that I do myself.

Facilitator:	Yes and in terms of whether you think the town’s quite social, would you think that Beechworth and Stanley are quite sort of socially connected towns or one that there’s a lot of isolation? How would you describe the sort of social opportunities?

Interviewee:	I’d say those two were connected yes. Very good in that way.

Facilitator:	Yes and if you think back to when we came to the town in Beechworth and we obviously promoted the program to people living outside Beechworth as well, back in March, do you remember what motivated you to join this program?

Interviewee:	Yes it was just to, I think from – I can’t remember what I said but I think it was just to get a bit of a kick-start to knowing that I did need to get some exercise and kick-start a bit of healthy eating, health and wellbeing just as I was getting older.

Facilitator:	Yes and kick-start’s a word that a lot of people have mentioned to us. It’s a common word that we’ve heard. What do you mean by kick-start? Are you talking about motivation, commitment, what’s it to you?

Interviewee:	Sorry, what do I need?

Facilitator:	Kick-start?

Interviewee:	Oh just a bit of motivation and I suppose just to get something started.

Facilitator:	Yes and if you think back as well when we came, we had an actually really good response from Beechworth but are there any factors that you think that may have prevented other women from coming along?

Interviewee:	Perhaps. Are you saying you did have a good response or you didn’t?

Facilitator:	Yes it was pretty good actually. It was actually better than we thought.

Interviewee:	Yes. I would say – I work in Myrtleford. I know there was a meeting in Myrtleford and of the women who I worked with, nobody knew about the one – because I was tossing up which just workwise, you know which would have been easier to go to and none of the women that I worked with had heard about it and even in Beechworth, there weren’t a lot of people who knew about it so I’m not – I’m not even sure where I found out about it. I know I got an email from – no. I did, I got an email from school. So people at school found out about it. Yes I don’t know. I don’t know but there weren’t a lot of people I knew who talked about it, apart from those women that I – most of the women that were at the one that I went to I knew. You know, I don’t know them really well but I know them.

Facilitator:	So maybe a lack of awareness was a problem?

Interviewee:	Yes maybe, maybe and just I suppose just that little bit of an extra reminder to say that it’s on. I think maybe people knew it was on but you know probably need that extra, come on it’s on, get a move on and go up.

Facilitator:	Yes and you might be able to answer this. We actually had a much better response from Beechworth and Myrtleford.

Interviewee:	Yes.

Facilitator:	Being someone who works in Myrtleford, any idea why that would be or any thoughts?

Interviewee:	I don’t know. I just think the – I know that – I think that Myrtleford – Myrtleford’s very much very big families. Not big families but it’s got a lot of families are there and have been there for generations and Beechworth is lots – the people I know they’re all people who have come to live in Beechworth have family. There’s not many – there’s a few families I know who are sort of long term families, got generations but most people are not new to town …

Facilitator:	Newer?

Interviewee:	But have relocated to Beechworth and I think there is a further motivation and you know people wanting to do things and get involved in things and I think that’s a big part of it.

Facilitator:	Yes and thinking back to the session, you had obviously attended the group session and then in the session we talked about healthy eating advice, we gave a few nutrition tips and then we moved on and we talked about techniques; we set goals together and we looked at problem solving and planning, was any information presented to you knew or was it was familiar? What was…

Interviewee:	Yes it was stuff that I knew. One thing I don’t think I was aware of was that regardless of what we do, as we age, we do put on weight, like every year we put on a small amount of weight which I suppose I’m noticing. I’m 46 now and I’m noticing that and part of it is I suppose it’s my eating as well but it certainly is a different weight spread of you know my body. My body has changed and I’ve noticed, yes. It’s probably been in the last two years that I’ve noticed just changes in my body that I didn’t notice at 40, 41.

Facilitator:	So in terms of the take home message for you, was there any sort of key message that you sort of took home? Is that the message that you took home or was there anything else?

Interviewee:	I think what it was that if you don’t do something, you know it’s up to us to motivate ourselves. Like we know all the information, for anything to happen we’ve got to actually do it.

Facilitator:	Yes.

Interviewee:	Like I know a lot of the push seemed to be that it was you know you were going to give us the text messages and we’ve got the booklet and there’s a website but it’s pretty much up to us to really, you know to make the long term and something that we’re going to do, it’s got to be self-motivated.

Facilitator:	Yes and in terms of the other information that was provided was most of that familiar to you?

Interviewee:	Yes, yes but it’s always good to hear it. I’m just trying to – I know I took some notes down. Yes, I think it was the – I can’t remember. I know the word, Optifast. But I don’t know what Optifast – I’ve got Optifast written down in my notes. I don’t remember what that was.

Facilitator:	I think someone asked about diet shakes. That’s just a diet shake.

Interviewee:	Oh okay, yes. No. Yes so there was stuff that I did know but it’s you know I think regardless of what you know it’s always good to hear it and it just keeps you alert and aware of – just because you know doesn’t mean you’re acting on it but to keep hearing it you know I could hear it again and go oh yes, that’s right. Just keep hearing those things.

Facilitator:	Yes and if you were to describe this program to somebody else, to a friend, somebody else in the community, if you were to finish sentence; the program was about – what would you say do you think?

Interviewee:	About making changes towards your whole – your healthy wellbeing I think and yes.

Facilitator:	Yes and since being in the program, again, we want you to be completely honest because the whole point of this evaluation is to get a – some insights in – insights from people involved in the program but do you think since starting this program it’s made you think more about your weight, what you’re eating, your exercise? Has it changed your awareness at all do you think?

Interviewee:	It hasn’t changed my awareness. I suppose I’ve always been aware. I – probably you know maybe 20 years ago I went to Weight Watchers. I was probably eight, nine kilos overweight. I went to Weight Watchers which I found really – and it was over a long – it was over about eight months so I lost about 10 , 11 kilos which was really good but that was a good learning like I got lots of knowledge about eating in that time and since that I’ve pretty much kept the weight off except for say the last two years where, as I said, it’s probably an age things as well. So I’ve always had that in my awareness in my mind. What it has done you know with those little text messages is it’s made me alert and just to keep on track and not so much the exercise. I can be completely honest here I’ve done no exercise and that’s one of my things that I really wanted to get on track of.

With my eating it’s been up and down and at the moment, like right today, it’s a bit of a down day but I’ve had spurts I suppose where I’ve been really good and drinking my water and writing down what I eat and just keeping tabs and even you know just starting off to write down what I had eaten and after I’ve done that for a couple of days then I seem to be on track and just be in a healthier state of mind and eating healthy without you know just doing it. And then, yes like actually the last couple of days we’ve had cakes at school and even for a while there when I’m being good I don’t even touch the cakes at school or the biscuit bin or but when they’re there it’s like I just go in a cycle. So I’ll have some after school, I’ll have some during school and you know to the point where I think it’s almost like well I’m being bad so I just keep being bad.

But the other thing I think you mentioned was if something goes wrong that you just start fresh the next day. It’s not like well I’m being bad so I’ll just continue being bad I’ll just – yes it’s a new day.

Facilitator:	Yes and has that been – have you been able to apply that do you think?

Interviewee:	Yes I – but it takes – I think it takes a few days just to kick in because I think when I’ve – in the times that I’ve had a bad day, then I’ve probably had a few bad days. It’s almost been like well like you know I’m sort of going downhill and then I think no and I can just be – because you know I went, as I say, I go up and down and I think it’s got a lot to do with me mentally as well, how I’m feeling, what’s you know what’s happening and yes but I certainly like at the moment I’ve probably since I finished it – when was it? What time of the year was it?

Facilitator:	March.

Interviewee:	March. I’d say I’d put on a few kilos since then but I’m not concerned about it. Like my weight goes up and down a few kilos all the time so it’s – I don’t put on great amounts of weight and I don’t lose it. I just sort of go up one or two kilos but I’m probably the heaviest I’ve been for a while.

Facilitator:	And you mentioned motivation. So just to confirm. It sounds like for you motivation fluctuates a bit, is that correct?

Interviewee:	Yes, that’s the same yes. I think one of the things that I probably would have liked with and with the people that were there as I said, I knew one or two sort of well enough to say hello to and the others I knew their faces. But even if there’d been something – some way that even we could connect, and that’s something we could have done ourselves but we sort of were there and then we were gone. There’s one that I’ve seen you know over the last few months I’ve probably seen her two or three times and as soon as we see each other we mention the program. That’s our common thing and it would have been good actually even to meet up with those people because I know there was one lady who I didn’t know but I know she was really keen to get something happening. So I think that could have been a possibility like at the meeting to say you know, maybe you people might want to get in contact. Yes, more of a chance for us to even talk and say well you know what are you keen to do and what are others keen to do and there’s a few now because there was another one – I think there was another one in Beechworth the next day? Was there another gathering? See I don’t know …

Facilitator:	We had a day and a night.

Interviewee:	A day yes. I went to the night one so the people who were in the day one, like I don’t even know who they are but I’m sure there’d be people in there who I know so even to find out that yes that were involved in the day one. As I said this lady that I see who was in the night one that I went too, I see her, we talk about it like yes, yes, you know what are you doing but there’s probably people I know who went to the day one, who you know we had met even worked out that we were both at the same sort of meeting.

Facilitator:	Yes and you’re not the only one that said that and I think that’s a really, really good idea. One lady suggested that like a Facebook group to keep in touch with everyone and to organise some sort of group activities involving physical activity would be a good idea. Would you think that would …

Interviewee:	Yes, no I do only because I think everyone’s on – most people are on Facebook now and even if it was just to say okay, next Sunday there’s a bike ride on do you want to come or we’re going for a walk. I’m still in that yes, I took up a bit of running for a while and I’m not a runner but I took it up thinking yes, I’ll do it and even to – I think I’m in the walking stage at this point but even to do the – just to get out and yes, that would be a good idea.

Facilitator:	Yes because I think you’re not the only one that said that. A lot of people have said that they felt that they lacked support from the group.

Interviewee:	Yes only because there wasn’t really a chance for us to get together and to talk about it and I know we had a bit of a chat over the night just of bits and pieces but yes. No, that’s a good idea.

Facilitator:	Do you think another group meeting would be ideal? That’s what someone else suggested as well, that another group session would have been beneficial?

Interviewee:	As in to follow-up? Yes. I think even if it was like you know two months down the track or even if it meant that people from the day and the night got together or you know even if it was because I don’t know how many people did the day one but I think there were only five of us who did the night one, or four of us.

Facilitator:	The day one was quite a lot bigger, about 15.

Interviewee:	Yes and I suppose that it’s probably yes, it’s about the size of it it’s just a matter of yes meeting people who are in the same area and have got similar and because and what I notice is you know the good thing is they were a similar age and similar needs as in we’re all busy, we’re working, we’ve got kids, it’s you know finding that time. Yes.

Facilitator:	Yes that’s yes and that’s a good idea, they’re really good ideas and do you think any of the components, you mentioned all of the components of the program before; you said the group session, the manual, the website, you had a bit of a phone coaching session and the text messages, was anything more useful? What was the most useful do you think?

Interviewee:	The booklet I haven’t really – I’ve got it and I’ve you know I’ve sort of flicked through it but I haven’t used it as it’s meant to be used and I know like obviously a lot of work has been put into it but it’s probably something I just need to revisit and again, that might have been something that could have, I don’t know, been tracked to say you know well look at this area and then go on and you know read this part this week. Even if it was a Facebook thing where we’re you know have you read – like a book club. Have you read section one and what do you reckon about that and just to get us on track I think because it’s here and it’s all in bits and pieces but it’s – I know that there were the sections but it wasn’t really like do we follow it through? Yes, it wasn’t really clear how to use the booklet which was sort of given to us. It wasn’t really clear as to how – I know we did our goals but yes, it wasn’t quite clear about the how to use it.

Facilitator:	Yes. And what did you think about the other components? The SMSs and the phone coaching?

Interviewee:	Yes, the SMSs were good because they were just a reminder that we’re still part of it and they weren’t intrusive at all, they were just a little quick reminder which was good and the phone coaching, yes, that was good because that was just – hang on, I’ve just got to say goodnight to my daughter.

Yes the first phone coaching was good. I had a goal. Was it with you I made the goal?

Facilitator:	I think it was with me actually.

Interviewee:	To drink water.

Facilitator:	Yes.

Interviewee:	And to drink some water and it was good it was like yes, so I did do that and I’m probably not doing as regularly as I was but I’m still doing it and yes, it was just a little goal and just keeping on track.

Facilitator:	Yes and that personal contact, some people have found quite helpful because they said that they felt more accountable if they had some sort of – a bit of individualised personal contact. Can you relate to that or is that something that you don’t necessarily do?

Interviewee:	Yes I can because a little bit like when I was at Weight Watchers like I used to go every week and that’s what kept me accountable. The fact that I was being good because I knew that the next week I was going and not that I had to lose weight but when I was losing weight, yes, that I did have to lose like a couple of pounds, a couple of kilos or a kilo. That I knew that I had a goal for that next week so it certainly kept me very accountable and you know even now I still try and do things like I – even if it’s like well I’ll weigh my, I haven’t done it for a while but when I’m being you know watching what I’m eating and doing stuff it’s like I’ll way myself in a week and you know just make sure that I’m still okay and my exercise is my big worry. But I just I find the time, like I get home late, especially in winter it’s dark, it’s cold, and yeah, I just need to do it. So I just, yeah, I find the time the biggest problem.

Facilitator:	Yes and what do you think motivates you? You mentioned that what could motivate you was accountability when you’re in Weight Watchers but what else motivates you do you think? Or what else could motivate you to get active and do more do you think?

Interviewee:	Other people and again that accountability for you know if I was meeting up with someone and I was exercising with them, I was keeping you know I keep – I suppose keeping a diary and that’s what the booklet and I know it allows the you know the walking and then doing the exercise in there but yes I don’t know. I don’t know.

Facilitator:	Is there something that we could have done do you think to help motivate you more?

Interviewee:	Unless it was something like a – not so much a Facebook but something where we could log in. You know you could log into something and say well even like on a weekly thing and just to log in and see how the week’s gone, like do it week by week. I mean that’s a lot of work for someone but that could be an accountability thing where if I am doing it by week by week and then I’ve got something to really strive for.

Facilitator:	Yes and you also mentioned before that it’s additional contact would – might help. You said a two month session is that correct?

Interviewee:	Yes with the group?

Facilitator:	Yes.

Interviewee:	Yes I think just as a – 12 months is a long time and already like what is it then …

Facilitator:	It would be about …

Interviewee:	Six months have gone.

Facilitator:	Yes about five yes.

Interviewee:	Five or six months have gone and so suddenly we’re halfway through it and I think as far as if we’re looking for things to be happening, that’s a long time and you know it’s halfway through and as far as changes, they haven’t really happened. Like I was really enthusiastic, really excited about it but I was also aware it’s not a program it’s just letting us be aware and we’re the ones who really need to take control and do it. So it’s almost like well yes, we’ve got to do it, unless you want to join a Jenny Craig or a Weight Watchers or a gym where they’re going to be onto you – you know not onto us but they’re going to be – we’re going to be accountable to that. Yes, we’ve got to be the ones who’ve really got to make the changes. But the support certainly does help.

Facilitator:	Yes and again being honest, you mentioned some changes that you’ve made before that lasted for some time. Do you think you’ve made any – can you discuss what changes you’ve made since being in the program or if you’ve made any? Again it’s low intensity so we’re not expecting anything massive but has there been anything you’ve been able to do, do you think?

Interviewee:	Yes well one of my – the water. I’ve been drinking the water and the other thing is, I know it wasn’t – one of my goals was I wouldn’t drink Diet Coke and I know Diet Coke hasn’t got sugar in it but it was almost like a – you know it’s quite addictive, the Diet Coke and it puts me in a – I don’t know what it does but it’s just – it’s almost like, it doesn’t make me feel crazy if I don’t have it which concerns me but when I’m drinking water I feel healthier. So that’s one thing I really try not to drink the Diet Coke because when I’m not drinking that I’m actually eating better as well. So yes the water’s been a big part of it and I suppose the other thing that I yes and again that fluctuates but when I’m being really good I don’t touch – my biggest problem is when I’m at school because at home I’m fine and we just have dinners and you know we don’t have sweets in the house. But at school we have morning teas probably once a week we have huge morning teas because it’s someone’s birthday and there’s always a biscuit barrel full of chocolate biscuits and that’s my biggest downfall. But when I’m being and I think that’s the thing. When I’m being good, I don’t go near them and if I’m not being good and I know that’s a terrible thing, good, being good and you know bad but then yes I do tend to attack them.

So I suppose I do, I am aware of what I’m eating and I do take my lunch to school and that was one of the things that I was often missing out, if I forgot my lunches I thought well I’ve forgotten my lunch and sometimes I’d just get a lunch order from school like a salad roll but often times I’d forget my lunch and I’d think oh well, I’d be right and then I’d just you know just eat whatever I could through the day. Just grab a couple of biscuits and then have nothing and then at the end of the school day coming home I you know called in and grabbed something to eat. So I don’t do that anymore as far as the pigging out on chocolate and you know sort of those really bad choices, I’m not doing those.

Facilitator:	Yes and did you stop those bad choices since you’ve been in the program or is that something you stopped before?

Interviewee:	It’s probably been – no I think it’s been – it’s probably been the last six months or so because like I really wanted it to – I wanted to go back in six months and at the end of the program and I want to have made some changes, so certainly I stopped doing that and I just have to I suppose my only – my advice is the you know at school just getting that under control because that is – that’s probably a big thing.

At school it’s busy, I’m hungry at different times it’s so easily accessible. Like it’s not a matter of calling into a shop it’s simply right there. I’m in the staff room every day. You know today there was you know an incredible amount of – not even a birthday it was just someone who had done a good job in the office or something and there’s all these cakes and biscuits and slices and oh you know. So that’s where I need to really and I went – can do it like I can stop it when I want to but I just have to get it under control.

Facilitator:	Yes and what’s making you want to stop that is it your weight is it – what sort of drives your motivation do you think?

Interviewee:	Probably like at the moment as I said, I’m probably the heaviest I’ve been for probably my 20 years, I’m probably the heaviest I’ve been and it’s only probably only a couple of kilos. I think I’m 66 or 67 kilos and I know normally my weight’s about 62. I don’t know what I was when I first saw you. Have you got the record of what I was?

Facilitator:	I’ve got – I’m at my house but I can text it to you.

Interviewee:	Okay yes no that’s all right. I think I was fairly – I think I was okay but I certainly feel I just need to get back into just back in control of what I’m doing so that’s my motivation like right now. But in the long term my motivation is for the fact that I’m getting older. I also, I think I told you I’ve got MS and I have a few health issues going on with that which is making things a bit more difficult and that’s where I really need to make some choices about not the food size but the exercise side because I do want to have my physical fitness. Even talking to you now is like well yes I’m serious about it. I’ve got to do it because yes I’m 47 in the next week or so and yes, I’m yes and you know as I’m getting older I’m getting longer between exercise.

Like I always used to do exercise like even two years ago I was at boot camp at the gym and I was doing stuff and I used to do that, like I’d do it for three or four months now. I wouldn’t do anything for a few months then I’d do something again and I’d sort of go up and down up and down and do different exercises but this is a long time since I haven’t done anything. So it’s probably about 18 months, two years since I’ve done anything decently.

Facilitator:	Yes and if you think about you know about motivation and what’s motivated you, some women found you know the small steps and breaking things up is quite motivational. Some women have used problem solving and goal setting to help boost their motivation. Have you used anything like that?

Interviewee:	No. No but I’m willing to try. I know the goal setting – I know like the small goal setting like when we first met and with the phone coaching, that certainly a really achievable way to do it but I think it’s just time to do it and yes, I just need to do it. So there’s no excuses, I just need to actually set some goals and work out my priorities. As I said I’m the one who’s going to benefit. Long term, as I said, the short term is probably yes the weight and just keeping myself at a good weight but the health is the biggest yes the long term benefit that I need to look forward to.

Facilitator:	And is social support – how’s that for you? Is that something that you think is helpful or a barrier? What’s that like for you?

Interviewee:	Well I’ve got my husband and kids. We do lots of stuff but we don’t do a lot – and the kids play lots of sport and netball and football and you know dance and lots of things but as a family, we don’t do a lot. We don’t do any exercise together. I mean occasionally I’ll go for a bike ride with my daughter but my husband’s health isn’t very good so we don’t even seem to walking as he’s not very able to do that so he’s got emphysema. So for us to do stuff as a family you know sometimes I’ve just got to say come on Pete let’s go for a walk because you know he can’t go very far. Not that that’s an excuse but we don’t do stuff as a family.

As I said I go for a bike ride with my daughter because you know she’s 10 and we can keep up with each other but because he doesn’t do anything that’s not much of a motivation for me. So it’d be me doing it by myself whereas 10 years ago, we used to and this is before kids, probably 15 years ago, we used to do lots of exercise together and we’d be great motivators for each other and also I don’t have – I have friends – a lot of my friends are my girlfriends from school which are in Melbourne and I still keep in contact with them but as far as social, like I don’t really have – there’s lots of people that I know and I’m friends with but I don’t really socialise with people in Beechworth. Like I see them, I say hello and yes I’m friends with them but I don’t really don’t do anything with any friends in Beechworth so I don’t you know and there’s not really a group I go out and go walking with or meet up with really much at all. So that’s probably a bit of a barrier.

Facilitator:	Yes so I guess it sounds like a yes a bit of lack of support could potentially be a barrier.

Interviewee:	Yes.

Facilitator:	And this is a hard question but what we’re interested in finding out is by you being involved in this program, whether you think it’s had any influence or any effect on any of your family or any of your friends. What do you think?

Interviewee:	Well my husband certainly knows you know I spoke to him about it and I you know when I first did it he actually encouraged me to – he got notice about it around about the same time I did and he encouraged me and said you know, this is something you might be interested in and I was and yes, he’s certainly supportive but supportive as in if there’s anything I want to do. But yes, it’s really – ultimately it’s me who’s got to do it. I spoke to some people at school about it because no one in the school has done it. So even if at school somebody had done it that would be a good thing too because it would be like well come on guys, we’ve got to do stuff and I mean the kids yes I don’t think they know. They probably knew I went to a meeting but and Isabelle always knows she knows that you know I’m conscious about my health and just making good choices because we don’t have junky food in the house and you know we do eat healthily as a family.

Facilitator:	Yes, so do you think…

Interviewee:	Disregarding of course my time at school.

Facilitator:	Yes but that’s not what – that’s in the staff room right?

Interviewee:	That’s different yes.

Facilitator:	Yes. So do you think you’ve changed anything that you’ve purchased or bought for your family or not really?

Interviewee:	No. We’ve always been pretty good. We eat really well. We don’t have sweet biscuits in the house my husband does most of the cooking and does the kids lunches and we’ve always been pretty good with that. Like on the weekend we’ll go into town we’ll buy some doughnuts or a pie you know buy some sweet things like that and occasionally like the kids have a little packet of lollies that last them three months and you know they have one every – they’re really good like that and all they drink is water and milk and they have juice in the mornings but they don’t drink soft drinks and they don’t do cordial so …

Facilitator:	That’s good.

Interviewee:	Yes that’s always been pretty good and so there’s nothing that we’ve really changed of that.

Facilitator:	Yes and a lot of people have said the same thing. If you’re already doing everything pretty well, then there’s less room for change.

Interviewee:	Yes.

Facilitator:	And in terms of your satisfaction with the program, based on your experience in this program, do you think it’s something that you’d recommend to other people in the community or to any other friends of yours?

Interviewee:	Yes I do. I think because obviously so much work has gone into it and I don’t know how the other women are going with it but I think there’s so much work you know there’s a great little booklet, there’s obviously the support that’s there. I think it’s not getting the full benefit if we’re not really able to monitor it more closely. As in you monitor what we’re doing and how we’re going. I think it’s yes I think maybe we need more accountability to what we’re doing or more meeting up because I think if we met up more regularly and I know that was – that wasn’t one of the aims of the program but I think by meeting up regularly, it just keeps us on track from you know in a short amount of time.

Facilitator:	Yes. So would it be fair to say that in terms of support you wouldn’t think it was adequate?

Interviewee:	For it to be effective I’d say no.

Facilitator:	Yes. What about in terms of delivering the information? Obviously you came to the group session, do you have a preference whether that information was delivered in a group setting or individual?

Interviewee:	I think a group’s more positive.

Facilitator:	Some people said they …

Interviewee:	Not more positive I just think it’s you know – there’s a chance for people to get to know other people in the group and to share ideas and you know to realise I think also that we’ve got the commonality there’s you know we’ve got the same concerns and we’re certainly all aware and wanting to do things so yes just that commonality is really good.

Facilitator:	Some people said that they felt they liked the group but then they felt that they didn’t have an opportunity to ask any questions. A lady I spoke to yesterday said she was a little bit embarrassed and wanted to ask some questions but felt embarrassed in the group setting. Did you have any feelings like that or did you think we needed some– she wanted a bit of one on one time so she could ask some personal questions. Is that something you think would be useful?

Interviewee:	No, not for me. I’m quite happy to yes, if I had questions with that group as I said because it was a small group. If it had of been a bigger group I’d probably be reluctant to be asking but I that group of five, that’s fine for me. I didn’t have any problems asking and yes if there was something personal that wouldn’t concern me.

Facilitator:	And sorry, back to that support, in an ideal world, how often do you think the group should re-meet?

Interviewee:	Maybe two months?

Facilitator:	Yes and after the two months? Like every two months or how often over the year?

Interviewee:	I think maybe two months and two months and then after four months and then maybe just review it after then like you’d have to see obviously if that’s – the two months if that was helpful or if people felt they need it or even it might even mean that – so are you talking about the group with you guys or just as us?

Facilitator:	Yes, either way?

Interviewee:	Yes because I think three – like two, maybe two months with you and then two months with you again and then after that see like even if some of us could meet together just as ourselves and because I think that’s what it is. It’s just that support from each other that is going to keep us on track and keep us thinking about what we’re doing so and maybe again you know, maybe another four months later with you guys again. Yes I think that would be good.

Facilitator:	Yes and any preference whether that facilitator was from Melbourne or was from a local lady?

Interviewee:	That didn’t really matter. I think yes it didn’t – wouldn’t concern me. It’s just I think if there’s someone facilitating it then it wouldn’t matter where they’re from.

Facilitator:	Yes and was there enough information?

Interviewee:	Yes, the information was good it was you know we didn’t want too much information I think as I said earlier, a lot of the stuff it’s stuff that we all are aware of and I think what really came through to me was because what was the age group that the…?

Facilitator:	18 to 50.

Interviewee:	Oh was it? 18 to 50 okay because I thinking it was older. No that’s good because yes because I think all the women were about my age. What was the question? I’ve lost track.

Facilitator:	Sorry, whether yes whether you thought there was more information that you wanted provided.

Interviewee:	No not at that stage because it was it’s early on and I don’t think we needed it. If certainly had we met two months after, there might have been other questions but we certainly had the chance to bring those up but there wasn’t anything. No.

Facilitator:	Yes, and last question. Is there any other tips for us that you think we could include to make this program better in the future?

Interviewee:	No. I feel I haven’t been on the website and I will get onto that because – no I think the follow-up the support might be but I you know I don’t know how possible that is. But even if that wasn’t available, for you to facilitate that we, the people in Beechworth get to meet up regularly and to do something. Like to still have it but for us to be facilitating it like it’s not really you know you guys are in Melbourne, it’s not really going to have to work if you guys have to travel up and down. But if it was facilitated as I said, through Facebook or even with someone getting us to get together or whether that be us getting together or but yes, somehow being facilitated that we could meet more often. Just to keep each other’s motivations up.

Facilitator:	Yes and one lady also said that she suggested, yes, getting the local community you know service involved so that then there was a familiar face involved.

Interviewee:	Yes, and that’s always a good thing. I’m sure there’s just people in health promotion and there seems to be plenty of people around in that area, in this area, you know and not too far away as in Wangaratta or Wodonga. There’d be people who are available in that way and even if it meant that you know one time when we got together we did some exercise and another time we shared some healthy recipes or you know you could focus it so you’ve got different topics that we’re looking at or you know different reasons to be gathering.

Facilitator:	Yes that’s a really good idea. Yes so give some topics to you guys. Okay perfect. Is there anything …

Interviewee:	Sorry.

Facilitator:	Anything else you think we should know or anything else that you want to add?

Interviewee:	No. I just think it’s you know I think it’s a great opportunity and there’s as I said, there’s obviously, there’s so much work that’s been put into it. It would be really good for yeah a lot more people that I know. As I said there are people who yes weren’t involved at all because they didn’t know about it so yes. Maybe if it was – if there were a lot more people doing it, again, there’s a lot more people likely to be on track. I know there’s people at school, not at school, at my kid’s school who meet and they go you know they go running and they go jogging, they do different things because I work I don’t but I know that they do that as a regular thing but I imagine they would have been interested in this sort of thing but yes. As far as I know they didn’t go.

Facilitator:	Yes and how do you think we could get them to go? Because we did advertise through the school newsletter but any other avenues that you think we could use?

Interviewee:	Yes. No because I – yes I know it took me a few – I did get the email. I got an email from work and then I think there was a flyer and then my husband got an email I think. So it was probably a few kicks before I got into actually doing anything so that’s probably a fault of people. They see something once, doesn’t mean they take it on board. It probably takes a few hits to think oh yes, yes I’ll think about that. The local paper, I’m not sure if there was – no there was something in the local paper I think, from what I remember.

Facilitator:	I think there was as well yes but that …

Interviewee:	Actually I just had a look through my book and my weight was 68.2 which and I’ve got my waist and my height which I haven’t done but that’s interesting because that’s about what I am now. Whereas I thought I was a lot less, so I’m probably around the same weight.

Facilitator:	That’s good so …

Interviewee:	And that’s the other thing. The tracking I haven’t kept up with the tracking but that’s a good thing but I haven’t used it so looking at it now, six months down the track, that’s a good thing.

Facilitator:	But you mentioned that tracking online could be another good one.

Interviewee:	Yes.

Facilitator:	I think that’s a good idea. Okay perfect, anything else you want to add?

Interviewee:	No. Just keep up the good work and it just the 12 months? How long does the project go for?

Facilitator:	The project goes for two years …

Interviewee:	Two years yes.

Facilitator:	But it’s just the 12 month one coming up in March.

Interviewee:	That’s the first year is it, the end of the first year?

Facilitator:	Yes.

Interviewee:	Yes okay, so after that?

Facilitator:	There’ll be one more the following year.

Interviewee:	Yes the same one or a different one?

Facilitator:	I think it’ll just be …

Interviewee:	Continuing on or …

Facilitator:	I think it’ll just be a continuation.

Interviewee:	Yes.

Facilitator:	The two year sort of timeline is still a bit vague and needs a little bit more thought put into it. But at the moment we actually, Beechworth was one of the last towns we visited so we actually start going back to the towns in September for the earlier towns.

Interviewee:	Okay, yes.

Facilitator:	So the year trips start in September.

Interviewee:	Okay yes.

Facilitator:	But I agree. I think we do need to maximise and provide a bit more support.

Interviewee:	Yes, yes no because there’s – and it’s – I don’t – we know what to do, we’ve just got to do it.

Facilitator:	Yes. One lady told me she’s looking for the motivation pill.

Interviewee:	That’d be good. I’d like that.

Facilitator:	Yes so I think that’s a - something that’s come up a lot so that’s interesting.

Interviewee:	Yes and I you know I don’t know if it’s an age thing or a health thing but I’m just finding it a lot harder now just to be motivated to do anything and it might be the winter I don’t know. It certainly doesn’t help but there’s been other winters where I’ve you know I’ve been exercising and doing things. So – but it’s just going to get harder as I get older so it’s time to you know I need to be doing things. You know I used to think how good I was that I was in my you know when I was 40, 41 that I was still doing exercise and still fairly active and then in such a short time I’ve really gone from doing stuff to not doing anything which is not good.

Facilitator:	Yes, and I think it’s easy to fall into the trap but it’s just about trying to work out strategies to motivate yourself.

Interviewee:	Yes.

Facilitator:	Yes okay well I hope that your motivation picks up and …

Interviewee:	Thank you.

Facilitator:	If you need any …

Interviewee:	And it has even by talking to you. Like it has I’m thinking yes it’s you know it’s probably time to get it happening again and just getting the exercise in. As I said even if it’s just a getting a walking thing happening or just starting on some small goal.

Facilitator:	Yes and if you need further support please feel free to contact us. There’s always someone in the office.

Interviewee:	Yes lovely okay thank you very much.

Facilitator:	Perfect thanks again so much for your time …

Interviewee:	Not a problem.

Facilitator:	And I’ll send you that gift card in the post when I get back to the office.

Interviewee:	Oh lovely.

Facilitator:	You’re welcome.

Interviewee:	Okay thanks very much.

Facilitator:	Thank you so much again. All the best.

Interviewee:	Okay no worries bye.

Facilitator:	Bye.

Interviewee:	Bye.

End of transcript

Participant# SS

Interviewee:	No problem.

Facilitator:	So I think you got the letter but the point of our evaluation is just to get your opinion about what you thought about the program. We want you to be completely honest so that we can improve the program in the future and then as a token of appreciation we’ll give you a gift card. The interview takes about 20 minutes or so.

Interviewee:	Okay no worries.

Facilitator:	Does that sound okay?

Interviewee:	Yes no problems.

Facilitator:	Great to start off with we’re looking at comparing the different towns we visited. I was hoping you could talk to me about the access to fruit and vegetables and exercise opportunities in Koroit?

Interviewee:	Okay there’s not much really available especially I guess for stay at home mums because you’ve got no real options where they include childcare but the fruit and veg is fine. Just up at the supermarket they usually have a good range.

Facilitator:	What about in terms of exercise? Is there much to – gyms and things like that?

Interviewee:	There’s really nothing in Koroit. You have to go to Warrnambool or Port Fairy.

Facilitator:	In terms of social activities some women have said that in their town there’s lots of opportunities to socialise and others have said, not so much. What’s Koroit like?

Interviewee:	I guess it sort of depends on who you know like I find now that my oldest is sort of going into the kinder there’s more opportunities there – you get to know more of the mums and more social that way but before that there was nothing really.

Facilitator:	Would you say the town overall a lot of the women seem sort of connected to each other or not so much?

Interviewee:	No not so much.

Facilitator:	If you think back to when we came to visit Koroit back in about October last year, do you remember what motivated you to join this program?

Interviewee:	Well yes I had just had – I had my second child in January and I had to have a knee operation after that and I had put on some baby weight and I just thought the support would be really good in helping me to stay focused and lose the baby weight.

Facilitator:	Do you think that there were any factors to make it difficult for you to attend or did you come alone or with friends?

Interviewee:	My friend, Kaye from Murray Goulburn. I went with her.

Facilitator:	Do you think there would be any barriers in the town or any factors that may have stopped other women from attending?

Interviewee:	Yes probably. It’s who you know and who you would have heard about it from.

Facilitator:	How did you – did you hear about it through the school or the kinder?

Interviewee:	No it was actually through Kaye at Murray Goulburn.

Facilitator:	Oh at the Murray Goulburn. Yes and sorry – if you think back to that session, we had – we talked about and I remember that session was quite brief but we talked about general health information and then we focused on behaviour change techniques like goal setting and we all walked through an action plan together – was any of the information new to you?

Interviewee:	No not really.

Facilitator:	What about – so is there anything in terms of key messages that struck – stood out to you that you learnt?

Interviewee:	Probably the main one was even if you have a bad day not to give up. You know, just pick yourself up again the next day and just start again instead of letting it get you down and thinking that you failed.

Facilitator:	Yes good and was that more do you think more from the manual or more from the group session?

Interviewee:	Probably more from the group session. Also from what you guys were saying and yes and what I read in the book.

Facilitator:	Yes and sorry to keep focusing on this, but in terms of that behaviour change strategy - so looking at barriers to achieving a healthy lifestyle – setting goals and making action plans, did you do that previously before you started the program? Sort of – I know you probably wouldn’t have written it down as such but is that something that’s…?

Interviewee:	Yes it definitely was because I’ve always struggled with my weight growing up – yes. I’ve always – since I was a – about 18 I’ve always sort of followed that kind of strategy.

Facilitator:	Yes so you’ve been doing that for a while so not so new to you?

Interviewee:	No.

Facilitator:	Since joining the program have you been able to make any changes to your lifestyle? We’re not expecting to see any big changes but have you made any – been able to change anything slightly?

Interviewee:	Yes – definitely have. I’ve definitely tried to have time to myself so that I can get my exercise in. I’ve tried to make that a priority. Beforehand that was the first thing that I would let slide.

Facilitator:	Yes and I guess is that through – yes just sort of setting time aside and thinking through ways to get more exercise?

Interviewee:	Yes and just planning ahead the week before. Trying to organise either my mum or my husband’s mum to have the girls for a set time so that I can make it to the gym or to an exercise class.

Facilitator:	Yes so it’s that sort of yes – planning and thinking about it that way. How much more do you think you’ve been able to exercise - once more, twice more?

Interviewee:	Twice more.

Facilitator:	Oh that’s great. That’s great and we spoke to a lot of – I’ve spoken to a couple of women now and a lot of women talked about that – when they left that session, initially they were really motivated but then they found that motivation sort of starting to decrease. Have you had a similar problem or for you it’s been pretty sustainable?

Interviewee:	It’s been sustainable but at the moment because I’ve just had back surgery so at the moment I’m not sort of allowed to do anything but up until that stage I – my motivation has been very high.

Facilitator:	Is there any reason again – what we’re trying to do is some women we’ve spoken to have sort of lacked quite a bit of motivation and we’re just trying to work out what motivates women. What do you think’s helped motivate you to – I mean obviously the surgery means that you can’t exercise as much, but what’s motivated you to keep this up do you think?

Interviewee:	Just my children. I want to have an active lifestyle so that I can keep up with my children and enjoy them as they grow up. So for me it’s not really a question. It’s something I have to do because there’s a certain type of life I want to have with my girls and I need to be fit and active to do that, so that keeps me motivated every day.

Facilitator:	That’s fantastic. You mentioned that you came to the session sort of to get that additional support. Again, being completely honest, have you been satisfied with the amount of support that we’ve provided?

Interviewee:	Oh definitely. Sometimes, you know, you’re having a bad day and you get through one of your texts and it just helps you stay focused.

Facilitator:	Yes so in terms of – again, what we’re trying to assess sorry is why – it’s fantastic that you’ve made these changes now but do you think that’s been because of the program or just because it was where you were at with your life?

Interviewee:	Probably where I was at with my life more so than the program.

Facilitator:	Just to clarify, what’s motivated you the most is being there for your children?

Interviewee:	Yes.

Facilitator:	Is there any reason why – that you’ve decided you’ve been able to make changes successfully now versus in the past?

Interviewee:	Well no sort of – even beforehand I lost a lot of weight and I maintained it. It was just the pregnancies that made me put the weight back on so yes. It’s just a constant motivation through my life really and this has just helped me refocus and get to where I want to go.

Facilitator:	So do you think that the program’s made you a bit more aware of your behaviour – about what you’re eating, your exercise and your weight?

Interviewee:	Yes definitely and it’s also made me realise that there’s a lot of other women, even if they’re not seen by me to be large, that feel the same way that I do, which is something I guess I never really thought about much before. That’s helped me as well to think that I’m not the only person who struggles.

Facilitator:	Yes definitely so that sort of support is useful for you?

Interviewee:	Yes definitely.

Facilitator:	What about support from your family? Some women have said – felt quite supported by their families and others not so much. Is that important of you or are…?

Interviewee:	Yes I don’t feel I get enough support but I try to do by with what I get.

Facilitator:	Yes and has that been potentially a barrier for you to be as healthy as…?

Interviewee:	Sometimes, you know, you can’t take the girls out when it’s raining – to try and do a run down the road or anything like that, you sort of need someone who can sit with the girls while you go off and do something. That to me is a barrier.

Facilitator:	Yes of course and in terms of your overall impressions of the program, if you were to describe this program to a friend or to a family member, how would you describe this program?

Interviewee:	Oh it’s a tough one. I like the easy going nature of it where it’s just setting your own goals. So for someone who doesn’t want the restrictions of like a Jenny Craig program or anything like that, I think it’s wonderful. You get to set your own pace and your own goals and I find for myself, that once I start seeing a result that it pushes me harder and I think that if you’re really serious about losing weight, that’s the first thing. Once you start seeing that result that it just kicks in even harder and you try harder to get to where you want to be.

So for people that are serious about losing the weight I think this is a wonderful program because it’s your own motivation and when you get to where you want to be you can feel proud that it’s you that has done it and it hasn’t been someone telling you what to eat, what to do, how much to eat. So it just feels like a bigger victory.

Facilitator:	Yes of course – so yes and I think that’s what a lot of women have said that – you know, sort of that feeling of achievement and pride that’s motivated them to keep going?

Interviewee:	Yes.

Facilitator:	In terms of – has the program sort of made you – helped make you feel a bit more in control or – of your health or of your choices?

Interviewee:	Yes definitely. It’s definitely made me realise that I’m in control of what goes into my body.

Facilitator:	Yes and what about in terms of – have you been monitoring your weight or has that been useful for you?

Interviewee:	Yes I weigh myself once a week.

Facilitator:	Oh good so - yes that’s great. That’s a perfect amount so not too much but that’s great and in terms of – if we look at the specific components of the program, so we obviously came to Koroit to the factory, then we gave a program manual, we had the SMSs, the website and the phone coaching – you had a phone call a few weeks ago. Were any parts more useful or more valuable to you than others?

Interviewee:	The manual’s always nice to have there so if you’re having a bit of a downer day you can flick back through and find the motivation again through the manual. So just having that, you know, right there all the time when you need it. I – that’s very helpful.

Facilitator:	Yes so have you been – a lot of women have said they sort of used it quite a bit at the beginning and then haven’t used it as much lately. Have you – has yours been consistent or…?

Interviewee:	Yes. Sort of – whenever I felt like I needed it – whenever I’d weigh myself maybe and didn’t have the results that I felt I should have got for that week, I would go through and flick through the manual and reset my goals and start again and try not to let the fact that I didn’t get as far as I wanted to put me down.

Facilitator:	Great. So it sounds like you’ve really done a good job also with identifying, you know, that perhaps – if a relapse happens just moving forward?

Interviewee:	Yes that’s right.

Facilitator:	What about the text messages? Some people found them – like you mentioned before, quite motivating. Was that good for you?

Interviewee:	Yes it was, definitely.

Facilitator:	In terms of the website, not many people have used that what about you?

Interviewee:	No I don’t think I’ve actually – apart from maybe the first couple of days after the initial [inaudible] it.

Facilitator:	Sorry one moment – and the phone coaching. Did you find that useful to have someone to call you and talk you through goals again or is it something you were already doing so you didn’t really need that extra support?

Interviewee:	No it was good. It sort of – and all the stuff she talked through and just remember what your aim and your goals are.

Facilitator:	That’s great. So what I’m hearing from you it seems that all components except for the website - sorry. All of the components were quite useful?

Interviewee:	Yes definitely.

Facilitator:	So what we were looking at is whether - if we took one away would it affect your motivation do you think?

Interviewee:	No probably not.

Facilitator:	If we were to take one away what would be the most – what would you take away first that would…?

Interviewee:	For me it would be the website. I just don’t find the time to get on the computer.

Facilitator:	No of course yes with your girls – busy lifestyle. In terms of since we came you mentioned that exercise has been one thing that you’ve focused on. Have you made any other changes to what you’ve been eating or anything like that or mainly just the exercise?

Interviewee:	Mainly just the exercise. I always to try to keep what I eat pretty similar to my fruit and my vegetables - I find it’s the exercise I really need to get to where I need to be.

Facilitator:	That great. It’s great that you seem very focused on one thing rather than changing everything. The next thing I wanted to have a quick look at is – what we’re interested in is the reach of the program. So whether this program has influenced anyone outside the program or just the people that were involved in the program – again don’t feel obligated to say it has because we’ve had very mixed answers for this but in terms of the reach of the program, do you think by you being involved in this program it’s had any influence on your family’s food or exercise?

Interviewee:	No not at all.

Facilitator:	No? Have you talked to your family about – how – I think your girls are quite – I think I remember you have quite young - your children, correct?

Interviewee:	Yes one and four.

Facilitator:	One? No not going to talk to them about health. What about your husband? Have you spoken to him much about health or about the program at all?

Interviewee:	Not a great deal. He sort of just follows my lead so what I eat he eats so yes.

Facilitator:	What about in terms of friends?

Some women have said, you know, since being in the program they’ve started to talk a little bit more about their weight, about what they’ve been eating and exercise with their friends and others, not so much – what about you?

Interviewee:	No not so much. I find that none of my girlfriends are really into the exercise side of a healthy lifestyle. They can sort of maintain the good eating for a while but none of them have the motivation for the exercise and the exercise is what really gives me an adrenalin rush so I – yes we don’t really talk about it because what I get out of it they don’t.

Facilitator:	Yes and some people also said they don’t – yes, either their friends are not on the same page, that’s why they’re not talking about it or if some people – I mean it doesn’t sound like it’s your case but some people also said for them their health was quite private. So that’s another reason.

Sorry – so just to confirm - would it be fair to say that you probably haven’t influenced anyone else’s behaviours except your own since starting this program?

Interviewee:	Probably. Me and Kaye that started the program together, before I had to have my operation we were getting together once a week to do an hour exercise and stuff.

Facilitator:	That’s great.

Interviewee:	Yes apart from that no one outside of the program.

Facilitator:	Yes no problem. Did you – have you had support from anyone else within the program besides Kaye or no?

Interviewee:	No – oh my friend, Ann-Marie, she was doing it as well. Yes.

Facilitator:	Yes oh good. The last thing we wanted to look at is – actually sorry, one more thing – you mentioned before motivation and about your friends not having any motivation. It sounds like you’ve done a fantastic job at increasing your motivation and sustaining that increase in exercise but do you have any tips for anybody else who might be struggling with motivation that we could perhaps relay to them?

Interviewee:	Probably the biggest tip is like when you first start, the first few weeks are hard. You have to push through probably for a month or more to start feeling better and see the benefits. A lot of people I think do it for a couple of weeks and you know, don’t feel like they’re getting anything out of it and they give up. It’s – you’ve got to yes really commit for at least six weeks to just – to start to feel better and notice the change.

That would be me advice – give it, you know, at least six weeks before giving in and pay ahead. I find that works for me. For ten sessions so you’re out of pocket so then you feel obliged to turn up rather than pay for – if it’s when you decide to turn up because yes I find that if I pay for a bulk lot of exercises I’ll stick it out until I’ve got my value for it and then I’m not feeling like I’m wasting the money.

Facilitator:	Yes because you’ve committed?

Interviewee:	Yes and that just keeps me motivated and keeps me going.

Facilitator:	Yes and what about – so some people find it – yes like you mentioned before with Kaye or finding a friend to exercise with. Do you think that’s…?

Interviewee:	Oh yes definitely. I found myself through the years - I haven’t had many friends that have – as I said have been into the exercise but I found a wonderful personal trainer who I get along with really, really well who pushes me and motivates me and that’s been a key for me because he’s more like a friend than a personal trainer. So even though he’s pushing me to get where I want to go it’s a fun environment. So finding that fun environment that makes you want to keep turning up each week makes all the different.

Facilitator:	Yes – making it enjoyable? Good that’s really interesting. Definitely we’ll pass that onto the next woman who asks me how she can increase her motivation. Based on – the last thing I wanted to look at is your satisfaction with the program but based on your experience, do you think you’d recommend this program to anyone else in your town?

Interviewee:	Yes definitely yes. Especially being isolated out here I think just knowing, you know, the person who’s two doors down from you that you might not really know could be going through the same thing and yes there are options out there for you. You’re not alone.

Facilitator:	Yes that’s really interesting and I think you said you also liked this program because it wasn’t too overbearing. Is that correct?

Interviewee:	Yes that’s correct yes. You feel free to have your lifestyle. You work it around your lifestyle.

Facilitator:	So is there any recommendations you’d make then to improve the program - any suggestions? Some people have suggested maybe as you’re probably aware we’re coming back in about six months but prior to that there’s no other contact. Any other suggestions of how we could improve this program? Would more contact help you?

Interviewee:	Not me personally but I could see from other people’s perspective that probably would yes. Maybe a six monthly revision or you know, a weigh in, so people can see maybe more than in a year where they’ve got to so, you know, if they’re half way to where they want to go then for the next six months, you know, they can focus again and realise that they can achieve where they want to go.

Facilitator:	Yes and in terms of the facilitator, do you have any preference whether that person is a local person or from Melbourne?

Interviewee:	No not at all.

Facilitator:	Do you think anyone else would? Some people talked about feeling a bit nervous to see somebody local because then they feel a bit more – a bit guilty. Do you think that could be an issue for some people or not so much?

Interviewee:	Yes I guess it could. It probably wouldn’t bother me so much but yes I guess in a small town the gossip factor I guess would be high. You’d have to make sure it was someone that you could trust with your information.

Facilitator:	Yes and in terms of the facilitator, are there any traits that you liked about her or didn’t like that you think make a good facilitator?

Interviewee:	The confidence – I like the confidence I just felt like they were sharing it with us. That they – even though they didn’t really know us but they just felt like if we really wanted it we could achieve what we wanted. I really liked that. I went away with confidence. I could achieve what I wanted to achieve.

Facilitator:	Any other important traits that you think a facilitator of this program would need?

Interviewee:	Empathy as well because sometimes you don’t get to where you want and that can be really disappointing. Yes they need to be able empathise with you but then be able to still steer you back on track.

Facilitator:	Yes good and in terms of the venue – I know we had it at the factor, was that an ideal location for you or not so much? Do you the think the fact that…

Interviewee:	I used to work at the factory that was fine for me. I guess maybe people that didn’t work at the factory may have felt a little bit uncomfortable being in that environment.

Facilitator:	Do you think it would have made people feel more or less comfortable that – I mean yes – as you said we targeted the women that were working in the factory – do you think it would have been – sorry – positive or negative that the all knew each other – everyone knew each other in the room?

Interviewee:	No probably positive. There would have been more of a comfort factor there to speak freely.

Facilitator:	Yes and that support?

Interviewee:	Yes.

Facilitator:	If you had a preference what would you prefer a group or an individual education session?

Interviewee:	I like the group.

Facilitator:	What do you see as a benefit of a group?

Interviewee:	You get ideas from other people, from hearing their stories and their input which you can then take and apply to your own life if it works in with your lifestyle.

Facilitator:	Yes I agree and I think it gives you good strategies so people are actually doing that so you know it can work. I think that’s what a lot of other women have said as well. In terms of the level of information provided, was that adequate for you or did you need more information, more…?

Interviewee:	No I found it adequate.

Facilitator:	Yes and you already touched on this but I think you said in terms of support, you were quite happy with the amount of contact provided?

Interviewee:	Yes definitely.

Facilitator:	Well that’s pretty much all I wanted to ask you. Is there anything else you think we need to know about Koroit or about – we didn’t actually do many workplace sessions – or anything else you think we should include in our evaluation of this program that would be useful?

Interviewee:	No not really. Not that I could think of off the top of my head no.

End of transcript

Participant #EJ

Facilitator:	Okay so to start off with we’re just looking at the different towns that we’ve been to. We – I was hoping you’d talk me through a little bit about Koroit’s access to fruit and vegetables and healthy foods?

Interviewee:	Oh okay well we’re only sort of 15 minutes away from Warrnambool anyway which has all the main shopping centres and fruit and veg anyway so I find that getting fresh fruit and veg isn’t a problem. We do have a local supermarket that also has fruit and veg but I just find they’re a bit pricey compared to yes - going to Warrnambool although you like to try and support locally. So yes, getting fresh fruit and veg isn’t a problem.

Facilitator:	What about in terms of opportunities to exercise? Would you consider them good, not good?

Interviewee:	Well in Koroit itself they’ve got a few different programs running now that they never used to in the past. Like I’ve actually just of recent times joined a yoga group and for the first time they’re doing that at the state school here in Koroit, that was never offered in the past but for the likes of Pilates and things like that – like I’ve started doing Pilates as well. I have to go to Warrnambool for that but as for general walking and all that yes - we’ve got the beautiful Vale Trail and things like that so we’re lucky.

Facilitator:	Yes and in terms of sort of social opportunities, would you consider the town quite social or not particularly social?

Interviewee:	Depends on your interpretation because I’ve got a young – my son plays in the Koroit football side so that side of it – I find Koroit yes a fairly tightknit community. Yes I suppose I’ve been here 20 years so it might be different for somebody that’s just come in but yes. No but as for yes going out and walking with groups and things like that, they haven’t got any of that. They’ve got a bike riding group that just started up but yes.

Facilitator:	Overall would you consider the town to be quite active or not so much?

Interviewee:	Depending what age bracket you’re looking at because we’ve got an older generation here in Koroit too but even some of the older ones tend to go up to the Senior Citizens and they’ve got exercise programs and that available to them so yes it’s hard to say overall but look there’s – we’ve got a guy that runs like - not personal training but that sort of thing like has a gym type set up and things so I think overall yes, Koroit would be fairly active.

Facilitator:	Has any sort of healthy…

Interviewee:	Sorry?

Facilitator:	Has any healthy lifestyle programs like our one been available before?

Interviewee:	No. Not that I know of.

Facilitator:	Yes and I think most women have said that as well and if you think back to when you joined this program, I know it was a while ago now, it was almost pack in end of October last year, do you remember what motivated you to participate?

Interviewee:	Well one of the mums that I do yoga with, she belongs to the state school and she was saying that it was on and at that stage I was looking at, yes, getting my health and everything on track so I thought I wouldn’t mind going – just having a listen to yes, what was being what said. Like I went with an open mind I didn’t really know what was going to be involved and all that but yes – no. I thought it was really good.

Facilitator:	Yes good and we actually had a – I mean a very good response from the women in Koroit but is there any factors that you think may have stopped more women from attending?

Interviewee:	I’ve got no idea how well it was advertised. I know it was done through the state school but other than talking to this mum and yes, something went home in their newsletter, I’m not involved in the other primary school newsletter anymore so I don’t know if anything went in that? I thought they actually had a fairly good turn up?

Facilitator:	Yes we did. I agree I thought it was pretty good.

Interviewee:	Much more it would have been yes not much room for where we had it.

Facilitator:	Yes I know it was a bit small wasn’t it?

Interviewee:	Yes in the library but no, I thought it was a great turn up actually.

Facilitator:	Yes and we were really, really pleased with that response. Some women though said that they thought that maybe some women may not have attended because they may have felt a bit self-conscious about coming to a program like that in front of a lot of people they knew. Do you think that could have been a problem?

Interviewee:	Possibly yes. I know yes – like – yes. No I’d agree with that. Maybe they don’t want to – like I’ve actually gone to Weight Watchers but I’d never do it in my local town because I wouldn’t want people to know what I was doing. Yes.

Facilitator:	But you felt comfortable coming to this program?

Interviewee:	Yes – no I didn’t have a problem with it at all.

Facilitator:	Okay good so it sounds like maybe – is that because – how come you felt comfortable to this program but not Weight Watchers?

Interviewee:	Well I didn’t know what this one was on. I thought it was more just talking about things you could do to improve your lifestyle and all that I didn’t realise it was – yes. I didn’t know sort of much about it but because one of the other mums asked me to go I looked it as a bit of a social outing.

Facilitator:	Yes and that’s what the whole point of it was to be social and just to be informal.

Interviewee:	Yes.

Facilitator:	In your opinion, now that you’ve been to the program if you were to describe it to somebody else, how would you describe this program? It was about?

Interviewee:	Well not necessarily about weight loss but maintaining your weight or looking at ways to improve in your lifestyle to making you a healthier person yes.

Facilitator:	What we’re interested in finding out is what different women’s’ interpretation of what healthy means. What does it mean to you to have a healthy lifestyle do you think?

Interviewee:	Well I think, you know, eating your fruit and veg and exercising and not overindulging and look it's not just about the food as well it’s the mental side as well so it’s just trying to get all that balance right.

Facilitator:	Yes of course and when you were – at that initial session in Koroit at the school. The program – the session was sort of split up into two major ideas. There was a whole lot of health information given about, you know, ways to eat better and exercise more and then there was also information given about – sorry – strategies to change your behaviour. So we talked about goal setting, we set action plans together and we talked about problem solving. Was any of this information new to you?

Interviewee:	The healthy eating side of it – a lot of it wasn’t but it was just interesting to have a diet – was it you that did the talk?

Facilitator:	Yes.

Interviewee:	Yes.

Facilitator:	You’re the first person to realise.

Interviewee:	I can’t picture why I said that, but I just thought yes – I mean as I said I’ve done Weight Watchers and I’m back on it again now but just to – yes some of your ideas and all that – I was really inspired for the first eight weeks and I actually lost weight and then I sort of fell off the bandwagon again but no – I found it really good. I thought it was yes, it was interesting and like just talking about having cashew nuts and something in your bag and all those sorts of things yes. No I’m a great one for it. I thought that side of it was great. I suppose I’m more of a person that needs to probably – if they had the meetings face on face more regularly it would be easier yes to follow it but I know that’s hard with yes – the logistics of it all.

Facilitator:	So for someone like yourself, it sounds like - and don’t think that you’re not alone. A lot of people have said that – that they were motivated, you know, for a month, two months and then slowly they just lost that motivation. Is that what you’re saying as well?

Interviewee:	Yes but that’s me full stop.

Facilitator:	That’s no problem and…

Interviewee:	It’s nothing against the program you sort of go – but it was funny because even the text messages you’d be having a bad day and you’d get a text message and you’d think, oh yes okay. Yes but anyway…

Facilitator:	Is there something that you think – you did mention this a second ago that more support would have helped you. Can you talk me through that a little bit more? What strategies could we have helped you – we just want to know how we could make this program better for someone like yourself?

Interviewee:	Well I would like maybe – because I know we don’t go back – I don’t – I think we don’t have a meeting face to face again until 12 months so even more regularly. Like I – and I’m not saying once a week or once a month but even once a quarter or something to – just talk – how things are going and more face on but that’s just me because that’s how I…

Facilitator:	No, no that’s okay.

Interviewee:	Yes some people don’t like the one on one they prefer to do it all online or whatever but I like that one on one.

Facilitator:	Yes and that personal contact it sounds like?

Interviewee:	Yes.

Facilitator:	Yes and do you think – one lady, assume – another – I’ve spoken to quite a lot of women and some women said that they like the idea of a bit more support because it makes them feel a bit more sort of accountable for their behaviour. Is that something that you agree with or not so much?

Interviewee:	Well to start off with I thought it’d be that but I knew a few that went but it’s not something that we sort of talk about or anything but at the start like because you gave us the red drink bottle. I had that at work because I work in Koroit and a few people would comment because they’d recognise the red drink bottle but yes…

Facilitator:	So perhaps a bit more support would have helped you a little bit more?

Interviewee:	Yes.

Facilitator:	Yes and in terms of – we talked about those behaviour change techniques like problem solving, goal setting, we did the action plans – was that a new concept to you or was that already familiar to you?

Interviewee:	No. That wasn’t so – I mean like with any weight loss you’ve got to sort of set your goals but I – what did I concentrate? Two things you changed me with is I definitely have the two pieces of fruit now. I was just eating maybe one but I always make sure I have the two a day and not weighing myself every day.

Facilitator:	So you’ve been doing that a bit less now?

Interviewee:	I’d break that habit. Yes.

Facilitator:	How much are you weighing yourself now do you think?

Interviewee:	I know you’re only meant to do it once a week but I might do it say, twice a week.

Facilitator:	Yes and no – that’s good. Is that – do you find that motivating or?

Interviewee:	I’m trying to get – I did to start off with but it’s trying to get my head around it – yes and then the walking as well. I think when I last spoke – when I last had the conversation I try to walk at least four times a week.

Facilitator:	Yes so what I’m hearing from you, it sounds like you have made some good changes is that correct?

Interviewee:	Yes.

Facilitator:	So what have you changed? You mentioned you’ve done a bit more walking?

Interviewee:	Yes so I’m back walking regularly. Fruit and yes not weighing myself as much but I have gone back to Weight Watchers as well so I’m doing all that as well.

Facilitator:	When did you start the Weight Watchers do you think?

Interviewee:	That was – I’m about into my fifth week so in April.

Facilitator:	Yes and has this program – did this program encourage you to do Weight Watchers or it was just something that you wanted to do?

Interviewee:	No did it or didn’t it? I didn’t want to come back and weigh in and be heavier than what I was which was the way it was going but you know, there was a number of factors that yes. I mean I’m a high flyer at Weight Watchers always do it, stop and then go back but yes, I’m back and motivated at the moment with it all.

Facilitator:	That’s good. So this program, sort of both of them together have helped?

Interviewee:	Probably worked yes they work together.

Facilitator:	Yes good and sorry – since starting this program do you think you’ve thought more about your weight, more about your exercise, more about what you’ve eaten or no change?

Interviewee:	Oh that’s hard because you can’t – with Weight Watchers anyway you’re looking at what you’re eating all the time. I think I’m more conscious of my walking and exercise yes.

Facilitator:	If we think now about the different components of the program, we looked at – in the program you’ve – you were given the manual. You had – we had the one session in Koroit, we have been giving you the SMS messages, there was a phone call recently like you said that you focused on exercise and there was also a website, what do you think has been most useful for you?

Interviewee:	Look I found the one on one session brilliant.

I got probably the most out of that and then it’s probably a combination of the – I mean I haven’t used the webpage as much but probably a combination of just the text messages to know that you’re still on the program and the phone calls yes, all sort of work together.

Facilitator:	Yes good. What about that manual? Some women have reported that they found it really helpful and others said that they haven’t opened it since the first session?

Interviewee:	Oh I completed it but I haven’t looked at it since other than last phone call. Yes.

Facilitator:	Did you do the activities in there?

Interviewee:	I did yes.

Facilitator:	Good so I know this is sometimes hard to think about but you mentioned before that you’ve been exercising more and increased your fruit, was there anything else that you’ve changed? That’s already quite a look so that’s…

Interviewee:	No probably not.

Facilitator:	When you did them, do you think that you used any of those behaviour change strategies like goal setting or the action plans? Did that help you do it or is it something you just did?

Interviewee:	I think it made me more aware of it like more conscious of it to try and make an effort like yes. Like I knew you shouldn’t be weighing yourself daily and just to have that talked about and all that I think well, I’m hearing it from too many places not for it to be right. Yes.

Facilitator:	It’s great that you’ve been able to exercise more. What I’m interested – is finding how come you’ve been able to do it this time round versus, you know a previous attempt do you think?

Interviewee:	Well I have done it before as well but this time round I’ve joined forces with the girl that I work with too so we’re actually walking before we work.

Facilitator:	Great.

Interviewee:	So we work three days together and then we sort of made it to an extra day so those four days, everything going well if no one’s sick or whatever, we try to walk. So to be accountable for that other person, yes that’s been good.

Facilitator:	Has the support from both the programs helped you as well?

Interviewee:	Yes probably your program – I mean, yes well - yes, no probably both programs yes because I was going to say I promised to improve that with my last phone call so I’ve been conscious of that.

Facilitator:	About the exercise?

Interviewee:	Yes.

Facilitator:	How have you – So if we were going to tell – give some advice from you to another woman that’s struggling with exercise, how have you managed to put that in? You said you’re walking with your friend. Anything else you’ve had to reorganise or do to achieve that?

Interviewee:	Oh look I think it’s more about your headspace and all that but it’s taken me a long time to realise that you’ve got to make time for yourself. You feel guilty doing it but I think it makes you a better mother. Especially out walking and all that too and involve the kids like so they know what you’re doing. I used to be pretty secretive with what I was all doing but this time round I’ve actually told them what I’m doing and I look at them say, oh do I really want to go for a walk? Put on your coat go. Yes so to get the support of your family as well.

Facilitator:	So – and have you found that your family’s been quite supportive?

Interviewee:	Yes.

Facilitator:	That’s great because the next thing I was going to ask you about was whether – what another thing that we were looking at is whether this program, by you being involved in this program, has it had any influence on your family or on your friends or anyone else? Has this resulted in them making any changes to their lifestyle?

Interviewee:	Well a lot of my friends and all that I probably – I haven’t spoken to them about being on the program and I – and as I said, I’m a fairly private person so this time round I’ve told family with the weight loss but I certainly haven’t told friends.

Facilitator:	Since joining this program do you think you’ve influenced what your family’s eating at all?

Interviewee:	Well I think it just – you probably don’t think you do but I think it’s a roll off – roll on effect because if you’re eating healthy yourself and doing healthy meals it follows on.

Facilitator:	Yes so maybe a bit more healthy meals in the house?

Interviewee:	Yes.

Facilitator:	Are the kids coming – you mentioned that they’re supporting you to exercise. Do they come with you or you just told them about it?

Interviewee:	No I’d like them to come with me but I can’t them to walk but my son’s a – in the local football anyway so his fitness levels and everything is unbelievable anyway and my daughter’s into dancing so she’s forever dancing around the house so…

Facilitator:	But they know at least? That’s good.

Interviewee:	Yes.

Facilitator:	Sorry – I don’t mean to pry but just…

Interviewee:	I put them on the bus, wave them and go for my walk. Yes they see me heading off each time.

Facilitator:	So they’re supporting you? That’s great.

Interviewee:	Yes.

Facilitator:	Sorry to ask you this again but just to clarify…

Interviewee:	No, you’re right.

Facilitator:	The behaviour – so you mentioned you’ve done more walking, you’re weighing yourself less and you’re eating more fruit. Has that been sustained since starting the program or that’s changed a bit? Like fluctuated? Sorry…

Interviewee:	Oh no probably fluctuated. I was pretty good there for a while and then I fell off the bandwagon but I’m back on again.

Facilitator:	Yes so it’s been mixed?

Interviewee:	Yes.

Facilitator:	In terms of – is there anything we could do to help you keep that motivation consistent?

Interviewee:	No I think at the moment just what you’re doing – the text messages and the phone calls and yes, all of that.

Facilitator:	Again, just to clarify, do you think you’ve used any of that goal – I mean you may not have written it down as such and you said you did finish the manual but do you think that goal setting and that sort of problem solving in your head has been helpful?

Interviewee:	Yes I think it just gave me a different view – a different take on things so yes, no I think it has. It’s going through your program that I’ve changed those things is not because of Weight Watchers with the weigh-in weekly and things like that, yes.

Facilitator:	Do you use – you said you found it useful when the girl called you on the phone but do you ever use any of those sort of problem solving strategies yourself?

Interviewee:	Look, I couldn’t even remember what – I knew they were doing – setting things small and trying to look at the obstacles and all that. You probably do it without really thinking about it.

Facilitator:	You probably do. It’s like when you said, you know, the rain – it was raining but, you know, you still – you know, that’s a barrier but you still went ahead and you did it anyway and the last thing I want to ask you was about your satisfaction with the program. So based on your experiences, would you recommend this program to anyone else in town? I won’t be offended even though I was the one who ran the session?

Interviewee:	Look at the start and all that I found it highly motivating. Even just to go and get ideas and think of ways you can do it and just realise there’s so many in the same boat. Like you’re not alone like, yes.

Facilitator:	No you definitely are not and that’s what a lot of people have found really encouraging from the experience is that they feel like everyone’s in this same situation.

Interviewee:	Yes.

Facilitator:	So you’d be likely or not likely to recommend it?

Interviewee:	No – likely. Yes definitely.

Facilitator:	In terms of the facilitator – I know sorry it was me but would you prefer if that person was from Melbourne or if it was a local person?

Interviewee:	I don’t think that matters. I thought – and I’m not just saying this because I know it was you I just thought you were very good and very motivating and spoke very well and – no, I found it great, the actual start up session.

Facilitator:	You said this before but would you prefer a group or an individual session do you think?

Interviewee:	No I think it’s better to have a group because you’re bouncing ideas and when you’ve sort of got people talking and things they were saying and some of the comments that come out, you didn’t have to say anything if you didn’t want to but you were sitting there thinking I relate so much to that yes.

Facilitator:	Yes and in terms of support, were you happy with the support provided or would you have preferred a few more phone calls or a few more text messages? Was there something else you would have liked?

Interviewee:	Probably a few more phone calls maybe.

Facilitator:	Yes and you – yes I think that’s what you said. You said that the motivation’s fluctuated but am I correct in saying that it seems that it got a bit better once you got that phone call from, Kelly, the other girl involved?

Interviewee:	Yes because when was that? What date was that?

Facilitator:	You got your phone call…

Interviewee:	I think that was about the time that I – everything sort of happened at…

Facilitator:	It was in April.

Interviewee:	Do you know what day?

Facilitator:	You were just about to start Weight Watchers, the 14^th^ of April.

Interviewee:	Oh well I started Weight Watchers on the 17^th^.

Facilitator:	Yes you’d just started.

Interviewee:	So it was all perfect timing around that time and I saw that as an omen I think and that’s a sign.

Facilitator:	Yes so the personal contact helped you?

Interviewee:	Yes.

Facilitator:	But it sounds like you’ve still made some good changes but the motivation has varied?

Interviewee:	Yes.

Facilitator:	Was there any other information that you thought would have been helpful to be included in this program?

Interviewee:	Not that I can think of off the top of my head.

Facilitator:	No? Anything else you would suggest to change about this program to make it better in the future?

Interviewee:	No other than, as I said all along, more contact – no.

Facilitator:	No? Yes. Okay good and is there anything else you think we need to know about Koroit that makes it an interesting or unique town that would be interesting for us to factor into the evaluation of this program?

Interviewee:	Not that I can think of off the top of my head. No.

Facilitator:	No?

Interviewee:	Other than some people said yes, people might be – because we’re – everybody sort of knows everybody or knows somebody so just the privacy time if people – yes - are not wanting other people to know that they’re part of it.

Facilitator:	Yes of course – so…

Interviewee:	Did you give – but then people would have had the opportunity to go to neighbouring towns anyway if they didn’t want to do it in their own town.

Facilitator:	You could've gone to Port Fairy if you wanted to but I mean at the same time it was a travel so it would have been a bit more difficult but we did have…

Interviewee:	But the travel for us is nothing. Like we’re used to travelling so to get in the car and go 15 minutes we’re used to having to go to other places to do things so I don’t think that would have been a real – would stop somebody from doing it if they really wanted to do it.

Facilitator:	We didn’t have many women from Koroit go to Port Fairy no.

Interviewee:	Oh right.

Facilitator:	I mean Koroit was one of the towns where we did have somewhere close by but some of the towns we went to didn’t have a close option but it seems like from, you know, what you – as you said, we had a really – very good response so it…

Interviewee:	Because I can’t – we had about 20 or so didn’t we?

Facilitator:	Yes we had about 20-25 yes.

Interviewee:	Yes see I reckon that’s huge.

Facilitator:	Yes and more women in the factory came along – like we did a factory session as well.

Interviewee:	Oh okay.

Facilitator:	So we really – in the end we had close to 30 so that was pretty good. One of the best towns we went to actually.

Interviewee:	Okay well you’re doing something right.

Facilitator:	We’re doing something right. Yes we loved Koroit. We’re looking forward to coming back it was a beautiful town so yes.

Interviewee:	Good.

Facilitator:	Is there anything else you want to add?

Interviewee:	No I don’t think at this stage.

Facilitator:	Okay no problem. So thank you so much for your time

End of transcript

Participant # TP

Facilitator:	Perfect. So I think you got the letter but the point of the evaluation is just to get your feedback. It’s not quite the same as the other phone call we gave you. This is more just to find out what you liked about the program and what you’ve learnt and how you think we can improve the program in the future.

Interviewee:	Yes.

Facilitator:	Does that sound okay?

Interviewee:	Yes that sounds good.

Facilitator:	Okay perfect. To start off with part of the evaluation is we’re trying to compare the different towns that we visited. We came to Koroit, as you’d remember, last October but I was hoping to get your opinion about what you thought in terms of access to fruit and vegetables and opportunities to exercise in Koroit?

Interviewee:	I thought the – I think they’re pretty good. Yes. I mean there’s access to fruit and vegetables, there’s the decent supermarket yes which stocks all that. It’s generally, you know, pretty good quality. I suppose the takeaway – there’s sort of fast food but there’s another sort of café thing which has healthier options so yes that’s pretty good and as far as exercise I mean it’s pretty quiet so – and there’s parks and you know, walking paths and things and like the bike path so yes. I think it’s pretty good.

Facilitator:	Yes so if you were willing to be active there’s plenty of opportunities?

Interviewee:	There are yes.

Facilitator:	What about in terms of social opportunities? So some towns we’ve visited there’s been like a lot of connection between the women in the town and others not so much. What’s it like in Koroit?

Interviewee:	I fit in – you know generally there seems to be a good connection. Yes like there’s a few women who run – well I know of one woman that runs like a fitness thing for women and there’s a guy that runs like a boxing thing. Yes. So I think – yes there seems to be a pretty good connection. Like lots of – I suppose there’s playgroups and then there’s, you know, things where mums get together, yes.

Facilitator:	So you can be – you can…

Interviewee:	Then with the school you could, you know, easily get involved like at the school level yes with other parents.

Facilitator:	Yes okay good and if you think back to when we came to Koroit, do you remember what motivated you to join this program?

Interviewee:	I suppose for myself it was more eating – wanting to eat more vegetables. Yes that was mostly it and yes exercise a bit more.

Facilitator:	Yes so was it to – I guess to find a bit more extra support or learn more?

Interviewee:	Oh yes. Probably learn more yes.

Facilitator:	In terms of barriers to recruitment we found that – we had actually a really good response in Koroit which was fantastic but are there any – do you think there was any factors that would have prevented more women from attending in the town?

Interviewee:	I mean maybe some were working.

Facilitator:	Yes we had that a lot. Yes.

Interviewee:	Yes, that’s the only thing I could think of and maybe those with young children, although, yes I think – did they offer childcare? I can’t remember.

Facilitator:	Yes we had a lot of kids in the room that were there but not – there was no childcare as such but children – it was a – everyone was welcome. In terms of your knowledge, you said you came to learn new things. Do you think you learnt anything new? Again, being completely honest is fine.

Interviewee:	Yes I think I’ve – I’m trying to think what I learnt. Probably just to be a bit more conscious like think about things more daily and yes that preplanning sort of aspect, thinking ahead yes.

Facilitator:	So were those behaviour strategies - we talked a little bit about – in the session we set some action plans together and about problem solving and goal setting. Was that a little bit more new to you rather than the information that was presented?

Interviewee:	So problems – yes. So how could you overcome that? Yes. That was good.

Facilitator:	Have you been able to utilise any of that since being involved in the program do you think?

Interviewee:	Yes I have.

Facilitator:	Yes? What have you been able to change since starting the program?

Interviewee:	I suppose I’ve been exercising a bit more. That could be having a child in – having two children in school now.

Facilitator:	Yes so that’s been…

Interviewee:	You know, so it could be…

Facilitator:	A mixture?

Interviewee:	You know, yes and I suppose the other thing is just being more conscious of making sure they have vegetables – like we all have vegetables every day and trying to incorporate as much as I can into the meals. Yes so just a greater awareness and it’s got me thinking about it more…

Facilitator:	Yes so you’ve started to think a bit more about your…?

Interviewee:	Conscious yes.

Facilitator:	That’s great and has that been – I spoke to a woman just earlier, from Koroit, who said that she found that her motivation had sort of fluctuated. So at the beginning she came back – after she attended the first session she was really motivated but now, sort of her motivation had sort of dwindled a little bit. Have you found a similar situation or yours has…?

Interviewee:	No. I wasn’t really – I’ve just sort of kept it at a level I think, you know, like I haven’t been over enthusiastic or under, I’ve just sort of yes. Kept it at an even – just sort of maintained – tried to maintain it.

Facilitator:	Yes that’s great and you said – so you said a bit more exercise. How much more exercise do you think roughly?

Interviewee:	Probably, you know, at least two more sessions a week.

Facilitator:	Great and then you said about planning as well?

Interviewee:	Yes.

Facilitator:	Anything else with specific planning you’ve made to your food intake?

Interviewee:	Not – I think I’ve cut back actually on like processed – having like a processed food after lunch. Like I’ll have the savoury part for lunch and then I might just have something small instead of a bit carbohydrate. Do you know what I mean? Yes.

Facilitator:	Yes and is there any reason – what we’re interested in is a lot of people like yourself, it’s fantastic, seem to have done really well. But is there anything about, you know, this program that you – has helped you make these changes successfully versus in the past when you haven’t been able to sustain them? Do you get what I mean? Sorry.

Interviewee:	Yes. I mean maybe it was it – you know, just me doing it with the other women.

Facilitator:	Yes so that support?

Interviewee:	Yes doing it as a group. Well I mean it’s not sort of like a group thing but maybe it was just that group – that initial yes sort of – and the discussions, you know and just explaining like everyone’s sort of in the same situation. Do you know what I mean?

Facilitator:	Yes of course. Yes so that sort of…

Interviewee:	Finding solutions together.

Facilitator:	Yes and working it out together that helped?

Interviewee:	Yes.

Facilitator:	So would it be – so that sort of strategizing and that problem solving together?

Interviewee:	Yes.

Facilitator:	That made it easier?

Interviewee:	Yes.

Facilitator:	Do you think that – again, don’t feel obligated just to say yes but do you think the support through the text messages or the support that – I know we haven’t provided a lot but the support we’ve provided has helped keep that…?

Interviewee:	Yes that was good. Yes text messages were good. Yes.

Facilitator:	Is there anything – so with the program we’ve had, the program included obviously the initial session in Koroit, the text messages, the manual and you recently also had a phone coaching session by a lady that just talked to you about setting goals and I think you said you’d – we’d talked about vegetables was there any specific component that you found more useful than another?

Interviewee:	Probably the group one, the initial yes. So probably that initial one.

Facilitator:	Yes so that face to face? Yes.

Interviewee:	Yes because honestly I haven’t really looked at the manual.

Facilitator:	Yes and don’t worry, you’re not alone and that’s what I was interested in asking you because most women actually haven’t…

Interviewee:	School work and…

Facilitator:	Is it just because you’re busy? Time?

Interviewee:	That’s it. You know, you don’t have time to sort of…

Facilitator:	Sit down?

Interviewee:	You know, yes but I mean the goals I certainly remembered the goals, you know, yes.

Facilitator:	So would it be fair to say that you’ve sort of been doing that goal setting in your head but not really writing down in the manual as such?

Interviewee:	Yes exactly.

Facilitator:	I think a lot of people said they flicked through it and then with life it gets busy so then they stop. What about family support or support? Do you find that – is that important for you in order to make changes to your lifestyle?

Interviewee:	Oh not really because I’m sort of the one that does it so yes.

Facilitator:	So you’re in charge of the?

Interviewee:	Yes pretty much. Yes.

Facilitator:	Sorry going back to this for a second – the text messages. Some women have thought they’ve been really used and helped remind them to do something whereas others found that it sort of didn’t create enough accountability. They could just skim it and you know, put the phone down. Did you find that useful or what was your reaction when you go the message?

Interviewee:	Yes I did. A good reminder yes like you know you expect something from a friend say or someone you know but no they were good yes.

Facilitator:	So it sort of made you think about it or reminded you to go?

Interviewee:	Yes.

Facilitator:	Would you go as far as to say it was motivating or more just a reminder?

Interviewee:	Probably a reminder.

Facilitator:	I think that’s been a consistent thing that we’ve found and if we were going to alter the program, say we were to take out one component of it or – is there anything of those text messaging, manual, that initial session or the phone coaching that you think we could take out that wouldn’t affect your motivation or wouldn’t affect where you’re up to?

Interviewee:	I think the manual yes.

Facilitator:	The manual yes? So that was the least useful?

Interviewee:	I think it’s important to write stuff down but maybe the manual it was just a bit – it was just a bit too much information.

Facilitator:	Yes so - and that’s…

Interviewee:	You know what I mean?

Facilitator:	Yes so maybe something a little bit more simple or less time consuming?

Interviewee:	Yes, small yes.

Facilitator:	In your opinion, what was your impressions about the aims of the program? If you were to describe it to a friend, how would you describe this program?

Interviewee:	I think just to generally improve – to me it’s like a bit of a health promotion program so generally improving lifestyle choices. Yes.

Facilitator:	Sorry to keep prying but what does healthy lifestyle mean to you? A lot of women have said a few different things. It’s been really interesting to hear. What’s your impression of a healthy lifestyle?

Interviewee:	Well for me it means having a balanced diet and exercise – being active as much as I can yes and moderation I guess.

Facilitator:	Yes of course and in terms of achieving a healthy lifestyle, for motivation, what do you think motivates – it sounds like you’re – seem to be living a pretty healthy lifestyle but what motivates you most do you think?

Interviewee:	I suppose well for my own health and wellbeing. I mean I’m a nurse so I sort of see the, you know, the effects of bad lifestyle choices so for, you know - the future health. I guess it’s maintaining health and also influencing my children…

Facilitator:	Yes of course.

Interviewee:	To hopefully take up yes healthier choices.

Facilitator:	Being a nurse, maybe you’ll be able to give us some insight. We’ve had a lot of women talk about sort of lacking motivation. Is there any strategies that you could suggest to them or anything that you think might be useful for them?

Interviewee:	Oh…

Facilitator:	Sorry.

Interviewee:	It’s – no, no, no I wasn’t…

Facilitator:	Yes, no, no…

Interviewee:	Maybe getting involved with another person. Doing it with a friend if you can and I think it’s – once you start, it’s just that initial starting because you miss it when you stop. Do you know what I mean?

Facilitator:	Yes.

Interviewee:	I think it’s that yes…

Facilitator:	That support and…

Interviewee:	Yes.

Facilitator:	Momentum? Yes. The next thing I was hoping to look at is what we’re trying to look at is whether this program influenced or had any impact on anybody outside the program so be it your friends or family.

Do you think since being involved in this program it’s changed – even I mean it’s only going to – the whole program is very low intensity but – so we’re not expecting any drastic changes but has it changed anything in your household in terms of food choices or exercise?

Interviewee:	No I wouldn’t say so. Well I suppose it has with the, you know, with the children. So there’s more, yes, there’s more, you know, vegetables. More awareness I think that yes we have to have them every day.

Facilitator:	Yes that’s great and do you talk to them about it or it’s more just – it’s the vegetables are on the plate?

Interviewee:	Oh we talk about it yes.

Facilitator:	That’s great and has that been more so since the program or something you’ve always talked about with them?

Interviewee:	I think more so since the program.

Facilitator:	Yes and I think it’s – as you said earlier, because you’re sort of thinking – it sounded like you were thinking about it a bit more and what about your friends? Do you talk about your health much with your friends or about food choices with them?

Interviewee:	Yes I mean I’ve got a friend who’s trying, you know, to lose weight and you know, exercise more so I guess, you know, we’ve been talking about it a bit yes but that’s as far as it goes really. I mean my friends are pretty healthy like they eat pretty healthily anyway so and have an awareness anyway.

Facilitator:	Yes so no particular examples of how you’ve sort of given them any extra information or anything?

Interviewee:	No.

Facilitator:	No? That’s fair enough. A lot of people said that in terms of this program they’ve sort of changed more about what they’ve done rather than influencing others because a lot of people said they felt uncomfortable or as you said, if you’re active, often your friends are active so there’s no need.

Interviewee:	Yes.

Facilitator:	The last thing I wanted to look at is just your satisfaction with the program and whether, based on your experience, you’d recommend this program to anybody else in the town?

Interviewee:	Oh yes I would.

Facilitator:	Any reasons as to why you would?

Interviewee:	I just think it’s beneficial and it’s a good program.

Facilitator:	Yes. Has there been anything like this before in Koroit?

Interviewee:	Not that I know of, no.

Facilitator:	In terms of the facilitator, do you have any preference if that person is from Melbourne or is somebody local?

Interviewee:	Doesn’t matter no.

Facilitator:	Any traits that you think make a good facilitator?

Interviewee:	I guess just, you know, not being – not dictating. You know, yes and then just encouraging any little efforts

Facilitator:	Do you think that the venue was a good choice of venue? Or – some women without children mentioned they would have preferred it somewhere else, do you think that would have made a difference?

Interviewee:	Yes oh it was fine. I thought it was fine yes.

Facilitator:	In terms of group versus individual?

Interviewee:	Group.

Facilitator:	Group? Is that again – I think you mentioned earlier because of getting to hear what other people are going through and working at it together.

Interviewee:	Yes.

Facilitator:	Did you think that the information was adequate that we provided?

Interviewee:	Yes very, yes.

Facilitator:	Our last question is – was there enough support provided from the research team do you think or is there ways that we could improve this program to make it better in the future?

Interviewee:	No I didn’t – I mean maybe – was there recipes in the manual? I didn’t…

Facilitator:	No on the website there’s some recipes.

Interviewee:	Is there? Yes.

Facilitator:	Yes.

Interviewee:	You know, yes, or even like tips on just getting extra vegetables in meals.

Facilitator:	Yes so some more…?

Interviewee:	You know maybe yes…

Facilitator:	Practical strategies?

Interviewee:	Yes.

Facilitator:	But for you it was enough contact? Some women wanted more face to face where other…?

Interviewee:	That was fine for me.

Facilitator:	Fine for you? I guess it probably depends on where you’re…

Interviewee:	Where you’re at yes.

Facilitator:	On the path of your – how much work you need.

Interviewee:	Yes.

Facilitator:	Anything else that you can think of that could make this program a little bit better or more effective for other women?

Interviewee:	No I think, no. I can’t think of anything.

Facilitator:	No problem.

Interviewee:	Yes.

Facilitator:	Anything else we need to know about Koroit for our evaluation that makes it a unique or different town to other towns?

Interviewee:	Oh I wouldn’t say anything I think – pretty much yes representative of, you know, most places. There’s – I mean just, you know, being a mum at the school you do see there’s a lot of junk food that gets dished out to the kids like it’s, you know, lunchtime and stuff yes so – which I think is pretty standard around this area. Yes unfortunately.

Facilitator:	Yes and I think as you’d probably know being a nurse that the people that need help are always going to be harder to reach.

Interviewee:	Exactly.

Facilitator:	So I think our sample will be quite representative of a more healthy population.

Interviewee:	Yes.

Facilitator:	But I guess there’s not too much you can do about that?

Interviewee:	Well no I mean – just got to keep trying. They’re doing – at the school they do healthy lunchbox awards.

Facilitator:	Oh that’s great.

Interviewee:	Which is trying to reduce, yes – they give out a healthy lunchbox award to each grade every week.

Facilitator:	Oh wow.

Interviewee:	They’re promoting like no packaging.

Facilitator:	Oh good yes.

Interviewee:	To really – you know, we don’t want packaged foods sort of thing so that’s good yes.

Facilitator:	That’s great because that works in with all your – all the healthy lifestyle and hopefully the kids will – make the kids eat better.

Interviewee:	There’s chips and bits and stuff yes.

Facilitator:	That’s great. Well I don’t actually have any other questions to ask you but thank you so much for taking the time to speak to us.

End of transcript

Participant #AR

Facilitator:	So the whole point of this is it’s meant to be just an informal chat just to get you talking about what you thought about the program, and to start off with we’ve just been asking women about the town that you live in. What we want to do is compare – we’ve got 42 towns enrolled in the program and just to be able to compare in terms of access to fruit and vegetables, and exercise opportunities between the towns. So on average we’re just getting your opinion about what you thought about that for Port Fairy.

Interviewee:	Okay well we’ve got one supermarket that’s open very good hours, from 8:00 in the morning til 8:00 at night. It does have reasonably good fruit and vegetables but limited, I still have to go 20 minutes to Warrnambool to get some fruit and vegetables that they don’t stock in Port Fairy.

Facilitator:	Yeah, and what about exercise opportunities?

Interviewee:	So there’s a swimming pool, indoor swimming pool and the beach in Port Fairy. There’s also a couple of private gyms which are accessible to everybody but quite expensive, like one gym session is $12 – ranging between $8 and $12 a session, and you can’t be a member and just go whenever you want. So for me I was exercising six days a week, using a gym six days a week, so six at $12 is $72 a week that I was paying to exercise.

Facilitator:	Yeah, so a bit expensive.

Interviewee:	So it’s expensive, so out of reach of a lot of people.

Facilitator:	Of course, and do you consider on average – I know it’s a difficult question – but to be an active community or not so active?

Interviewee:	Relatively active I guess because there’s the surf lifesaving club so there’s a lot of people that do that sort of stuff, and do surfing, and there’s the basketball association, and there’s soccer, and netball, and football, and stuff like that. But there’s a lot of people who don’t do anything.

Facilitator:	Yeah of course, I guess that’s the same in every town.

Interviewee:	Yeah.

Facilitator:	And what about in terms of social opportunities? We went to some towns and we found that people seemed very connected with each other and others not so much. What’s your thoughts on that about Port Fairy?

Interviewee:	Well I don’t socialise much in the evenings because I work in the evenings, but it is – it’s very – I would have to say it is a very social town and a very connected town because Port Fairy won the award for the most liveable community in the world. So we have quite a good community atmosphere, not necessarily activity orientated though but it is a good place to live definitely.

Facilitator:	Yeah it’s beautiful, and now if you think back to when you joined the program back – I think it was around October last year, do you remember what motivated you to join this healthy lifestyle program?

Interviewee:	Well I just started losing weight using Michelle Bridges’ weight loss program, and so I wanted this as another resource for me to be able to look back on to try and help me lose weight.

Facilitator:	And how have you been going on the Michelle Bridges program?

Interviewee:	I’ve lost 20 kilos.

Facilitator:	Wow that’s fantastic.

Interviewee:	Yeah, so I’m happy.

Facilitator:	That’s great, and did you find – we had in some towns some people talked about different barriers for other women attending, in Port Fairy we actually had a pretty good response if you remember but can you think of any factors that stand out to you as to why some women in the town wouldn’t be able to attend, or it wouldn’t appeal to them?

Interviewee:	Really probably only laziness, but maybe because it is a small community they’re nervous about doing something that everybody is going to be watching.

Facilitator:	Yeah we’ve heard that a lot.

Interviewee:	Maybe, I mean for me one of my friends had lost a lot of weight using Michelle Bridges as well and it was like a real motivator for me, but then other people would use it – because it’s a public thing and everybody knows you’re doing it other people would feel very nervous about doing it. That would be my only understanding.

Facilitator:	Yeah and I think that’s a really good point, and that’s what we’ve been hearing a lot, that a lot of people feel it’s a bit of a barrier to come forward and to say I want to do something about my health.

Interviewee:	Yeah, which was one of the things – the very first thing with Michelle Bridges – I hate to be preaching her, but when you join up before you start, and the starting day for the next round is like today – so for the month beforehand she sends you messages each week, okay this week I want you to get rid of the crap out of your kitchen, and chuck out those Coco Pops, and chuck out this, and buy fruit and veggies, and so she’s getting into your head before you start. Then another one she says is don’t make this a secret, tell everybody you’re doing it, post it on Facebook that you’re doing this and you’re going to make your life better. So it makes everybody join Facebook groups, so like in Warrnambool and Port Fairy there is a massive Facebook group of people doing or that have done Michelle Bridges. We’re always posting each other – I’ve done this, I’ve done that, I need help with this, can somebody help me, my shins are hurting what shall I do – so there’s a heap of support.

Facilitator:	Yeah, so do you think for you that in order – it sounds like you’ve been able to make these changes because of that support provided, is that correct?

Interviewee:	Yeah, masses and masses of support, girls I’ve never met – we all went to Melbourne, there was 30 of us last week, we all went to Melbourne. We roll in accommodation together, none of us knew each other really apart from chatting on Facebook and meeting and going and doing walks in the Grampians together, or we went and did stuff in the Otways together. We’d just post on Facebook okay who wants to come, we’re going to do this, and there’s every time out of maybe 200 people in our little Facebook group we might get five, we might get ten, and we’d all go and do something together and we’re all very positive because we’re all making that change. If you could get something like that going it would be amazing because everybody’s then got that support.

Facilitator:	Yeah, so do you think that this program – again we want you to bed completely honest because the whole point of this evaluation is to improve the program – do you think that this program had adequate support or not enough?

Interviewee:	Yeah I think it did, I really liked getting the text all the time just to keep you on track. The only negative thing I could say is that the very first time when we sat down with the girls in Port Fairy and we were looking at the book I didn’t get a chance to look at the book before we did it. So I had things filled in halfway through the book and not stuff filled in at the start of the book, and it took me a long time to get that all sorted out, and that’s all. But it was a good thing and I liked the texts, and I liked all those little reminders and little positive things, they were great.

Facilitator:	Good, and what did you think – one of the things that we’re interested in looking at is whether this program helped to improve your health knowledge or whether you learnt anything from the program. As you might remember in the first session we talked about general healthy eating and general lifestyle advice, and then we also talked about strategies to help make behaviour change like goal setting and developing action plans. Was any of this information new to you or was most of it more of a refresher for you?

Interviewee:	For me it was probably a refresher because I was doing Michelle Bridges at the same time, but if I hadn’t been doing Michelle Bridges it would have all been new and it was all very good and useful.

Facilitator:	Yeah, anything that stood out to you as being most useful as a refresher?

Interviewee:	No, the thing I remember the most that I quote a lot to my friends is that she told us that women naturally will gain 2 kilos a year and just by eating one or two less biscuits that will stop that extra weight gain. I found that quite a remarkable concept, so just that one less biscuit every day can make that much difference.

Facilitator:	Yeah, so for you that concept of making a small change was new to you?

Interviewee:	Small change, yeah.

Facilitator:	That’s great.

Interviewee:	And a lasting thing really because you just think it doesn’t have to be a big change, it has to be a continuous change though probably.

Facilitator:	Yeah and that’s really interesting, and that was the whole point of the program, just to make those small changes to help benefit your health, so that’s great. So that’s what you found most useful?

Interviewee:	Yeah.

Facilitator:	And if you were going to describe this program to somebody else how would you describe it? So if you were to finish my sentence “the program was about...” what was your impressions?

Interviewee:	Making small changes that make a big difference in your life really I guess.

Facilitator:	Yeah, and as you eluded to before we focussed on healthy lifestyle, but what we’re interested in is trying to get what you consider a healthy lifestyle. So in your opinion what does a healthy lifestyle mean do you think?

Interviewee:	A healthy lifestyle is being fit enough to be able to enjoy your life, imparting that on your kids and not doing anything that will damage your body, limiting the bad things I guess.

Facilitator:	Yeah, so it sounds like a big motivation for you is your children, is that correct?

Interviewee:	Yes.

Facilitator:	See it’s fantastic that you’ve...

Interviewee:	See I’ve got to play and keep up with the kids.

Facilitator:	Yes, it’s great that you’ve done such a good job. Is the motivation for you mainly about being healthy, or what’s motivated – I mean it’s great that you’ve set an example for everybody else but what we’re interested in is finding out what’s made you change now versus in the past, is anything – can you think of anything significant?

Interviewee:	The main thing that made me change and made me start losing weight was I saw a photo of me, because I’m usually behind a camera, not in front of the camera. I saw a photo of me and decided that’s it, that’s ridiculous, I’m so fat it’s out of control. I’d say okay kids you go for a bike ride and I will drive the car to spot A and we’ll meet there rather than me riding the bike with the kids.

Facilitator:	Yeah, so self-consciousness, is that fair?

Interviewee:	I just don’t think I could do it because I was too unfit.

Facilitator:	Yeah, so what motivated you to change was your being unhappy with your past weight, and as you mentioned earlier keeping up with your children.

Interviewee:	Keeping up with the children, yeah.

Facilitator:	That’s great, I’m glad to hear that you’ve done so well. Since starting the program – I know it’s a bit difficult to separate Michelle Bridges and this program but if you think about just this program for the moment do you think since starting the program you’ve thought more about your weight, more about what you’re eating or about how much you’ve been exercising?

Interviewee:	Definitely yeah, because I’m always thinking of that little biscuit story.

Facilitator:	Is there any one thing you’ve thought about more than the other or all of them?

Interviewee:	No not really any one thing, probably all of them – yeah all of them equally I would think.

Facilitator:	Good, and have you been weighing yourself or have you found that useful?

Interviewee:	Yeah I do weigh myself but that’s also part of the Michelle Bridges thing as well, but I would have been doing that, if I wasn’t doing Michelle Bridges I would have been doing that, a weigh in as well. But that’s also very motivating.

Facilitator:	Yeah why do you think that’s motivating?

Interviewee:	Because you can see the difference it’s making in a physical way whereas I think you can’t actually tell by looking at yourself if you’ve lost weight, or if you’ve lost measurement, not just weight, but if you put a tape measure around your hip you can’t – if you look at your hips you can’t see that they’re much smaller than they are until you put tape measure around them and things like that.

Facilitator:	Yeah really good point.

Interviewee:	It’s a way of gauging it I guess.

Facilitator:	Yeah so that constant feedback.

Interviewee:	Yeah, I mean I can see it now when I walk past a window and I look in the mirror I go god that’s me, but when I’d only lost a couple of kilos at the start I wouldn’t have thought there was any difference.

Facilitator:	Yeah, but now you’re feeling much better?

Interviewee:	Yeah but because I’d measured myself and weighted myself I knew there was a difference but I didn’t see it, but I see it now definitely because 20 kilos is a lot, but at the start it was a really good motivating way to lose weight.

Facilitator:	Yeah, and now you touched on this before about the different parts of the program, as we said we’ve had the text messaging, we’ve had the group session, the manual, and you also had a phone call not that long ago. Did you find any particular part of this program more motivating than another?

Interviewee:	More motivating? No I think the most motivating thing would have been the text.

Facilitator:	Yeah, and with the texts – sorry to keep prying but some people talk about the texts as being a reminder and others are talking about it being more of a motivator, what was – once you got that text message what did it do for you?

Interviewee:	Well both, it did remind me, definitely reminded me to look at the book, but it also did motivate me because you felt yeah they’re really interested in what we’re doing and we’re really going to make that change. So they were both reminding – both...

Facilitator:	And again it sounds like that support was important to you.

Interviewee:	Yes, just like when I go to the gym I need her yelling at me to keep going and not stop.

Facilitator:	Yeah that constant motivation and...

Interviewee:	Come on do two more, don’t stop now.

Facilitator:	Yeah, so it’s kind of that – would you say – one woman said she liked the text messages because she sort of found that it made her a bit more accountable.

Interviewee:	Yes, that’s right.

Facilitator:	Yeah, and is there any – we’re just thinking about in the future altering the program slightly, if we took away any of those program components is there any ones that we could take away that would leave you as motivated as you are presently? So if we took away the phone coaching, or if we took away the manual, is there anything that you think could be left behind?

Interviewee:	I like the manual because it was something to look back on and I’d read it before I went to bed – I’m comparing to Michelle Bridges again, you couldn’t do that because hers is on a computer and I don’t take my computer to bed. So I like the book and I’d sit there and read it and think yes I need to do this, and yes that makes a difference. So I like the book although I understand the book is being a part that might go cost wise. But I don’t think so, I like how it was.

Facilitator:	Good, what about the phone coaching, did you find that motivating or valuable?

Interviewee:	I don’t remember the phone coaching, I remember the interview sort of like a long interview like this but I don’t remember any other phone thing.

Facilitator:	Yeah that was the phone coaching, so she just rang you and sort of helped set goals with you and focus you. I think I’ve got your file in front of me, you said that your overall goal was to fit into a cocktail dress.

Interviewee:	Yeah and I fit into it.

Facilitator:	That’s fantastic, that’s amazing.

Interviewee:	Okay I must have had it then.

Facilitator:	Yeah you had it sorry, it was a while ago now though, it was back in the beginning of April.

Interviewee:	Yeah okay then, and I’d just had an operation then so that’s probably why I don’t remember it. So yeah I fit into it, it was all good.

Facilitator:	That’s fantastic, so it sounds like you’ve made significant changes to your lifestyle, is there anything in particular that you’ve changed that you can sort of discuss with us? How have you lost all this weight, what have you actually done?

Interviewee:	What I have done is eaten less food, portion sizes were my main killer. I mean I did exercise as well but since I’ve had my operation I haven’t exercised at all except to walk down to the shop for a coffee though I’ve still lost weight. Over the last three months I haven’t been exercising and I’ve still lost three kilos.

Facilitator:	That’s great.

Interviewee:	Yeah so I was doing a lot of exercise but I’ve still been able to keep losing weight by just watching what I’m eating. I’m not totally crazy but I’ve been really watching my portion sizes and that made the major difference for me. The food I ate before was never bad food it was just too much of it.

Facilitator:	Yeah so you’ve just been cutting down the size?

Interviewee:	Yeah portion size, portion control which is my main [inaudible], and really – this is Michelle Bridges again, sorry – the other thing she sort of taught me I guess was it was called lay by system. So let’s say you wanted that Tim Tam I said I’m not going to have it today but if I still really want it tomorrow I could have it. So you’re sort of put off the urge and that worked a lot, I mean that was nothing I don’t think mentioned anything like that in the booklet but that worked because I’d go no I’m not going to have it today and if the next day I still really needed some chocolate I’d have a little square or something and really enjoy it, but I didn’t go crazy – well I did last night.

Facilitator:	Yeah everyone’s allowed to have a lapse.

Interviewee:	Yeah.

Facilitator:	And do you think that that portion control, and like you mentioned that Tim Tam was a result of Michelle Bridges, but do you think that portion control was more a result of being in Michelle Bridges or being in this program?

Interviewee:	Well it was mentioned in both so it’s hard to...

Facilitator:	Differentiate?

Interviewee:	Yeah, we can say it was definitely part of your program because it was in there as well but the lay by system was definitely Michelle Bridges, but...

Facilitator:	Yeah so it’s a mixture.

Interviewee:	Yeah.

Facilitator:	And you said before that the thing that’s been motivating you and helping you is social support, is that correct?

Interviewee:	Yes definitely.

Facilitator:	And what kind of support do you think is most important to you, is it support from someone like Michelle Bridges, or us – so an outsider, or support from family, or friends, or is it a combination do you think?

Interviewee:	Well definitely support from you guys and any program like Michele Bridges or whatever, that support was very useful. The social network support that I got from Michelle Bridges was massive. My actual personal friends – two or three of my friends that are really overweight that need to do this have gone absolute opposite and haven’t given me any support, and basically don’t talk to me now, don’t even mention it – wow you look great, you’ve done really good – they don’t mention it, it’s like I’m still 20 kilos heavier. So my actual close friends have gone the opposite way and not supported me at all, but I’ve found new friends in the meantime that are supportive, so it’s working better than having non-supportive friends.

Facilitator:	Of course, what about your family?

Interviewee:	Yeah they’ve been really good, my children have been a little bit too good – mum stop eating that chocolate – but yeah they’ve been really good. My family have all been eating the food that I’ve been serving them, I haven’t been eating different food than them so that – we’re all embracing that healthier eating.

Facilitator:	That’s fantastic because one of the things that I was just about to ask you about is what we’re interested in is finding out whether this program has only influenced your behaviour or whether it’s caused changes in your household. So it sounds like as a result of being in this program and Michelle Bridges is it fair to say you’ve influenced your families food choices?

Interviewee:	Yes definitely.

Facilitator:	And what have you – you mentioned portion sizes, what else have you changed in terms of what you’re buying for the family?

Interviewee:	Well I still have to feed my children as much as I fed them before but basically I never ate bad food so it was just not cooking the whole packet of pasta instead of half a pack of pasta. So I think that I haven’t bought anything different because we always ate lots of fruit and veggies and we ate lots of food. The main difference I guess for one of my sons who is a bit podgy is that I made him – instead of just walking up to the fridge as soon as he’s bored, because that’s when he eats, is I say you can’t eat anything until you’ve had a big glass of water so then he’s not so hungry and doesn’t scoff so much. Food wise they ate well anyway, they didn’t eat crappy food.

Facilitator:	So it was just the portions that you’ve changed for the family?

Interviewee:	It was just the portions – yeah for my husband, I didn’t really change it for the kids that much because they’re only 10 and 12.

Facilitator:	Yeah of course, and you talk about – since being in this program would you say you’ve talked more or less about your health, or about weight and food?

Interviewee:	More, heaps more.

Facilitator:	Heaps more, and is that – I know again it’s a bit difficult – is that because of both programs, or this program, or...

Interviewee:	Yeah both programs.

Facilitator:	Both, and you...

Interviewee:	I have definitely spoken about your program to my friends who – like about two different ways that you can lose weight. Some people can’t afford to pay Michelle Bridges $200 for three months so it was a great way for people to have that option to make a difference.

Facilitator:	Yeah, did you come with friends to the program or by yourself?

Interviewee:	I came by myself, I tried very hard to get one of my friends who was 160 kilos to join your program but she wouldn’t do it.

Facilitator:	Yeah, and that’s no problem. So you said that your friends haven’t been supportive but it sounds like you have actually talked to them about the program.

Interviewee:	Yes definitely.

Facilitator:	Any examples of how as a result they’ve changed their behaviour or they haven’t really been influenced by it?

Interviewee:	No they haven’t at all.

Facilitator:	Yeah, and that’s okay.

Interviewee:	Maybe one day they will.

Facilitator:	Yes hopefully when they’re ready, and I guess it’s about when they’re ready.

Interviewee:	When they’re ready, yeah exactly.

Facilitator:	And going back to about all the amazing changes you’ve made did you find that when you were making these changes you’ve used anything like goal setting or problem solving? Has any of that been helpful for you or it’s more just about doing it?

Interviewee:	I just did it, I did set goals like fitting into the cocktail dress, but also I set – initially I set myself a goal of losing ten kilos over six months, but I conquered that well and truly, I did nine kilos in four months I think. So my main goal once I started losing weight and I knew that I could do it was to keep it up and to get into the dress. So now I have to set myself a new goal because I’ve done the dress.

Facilitator:	You can get into another dress perhaps.

Interviewee:	No that was – I’m not a person like that normally but I had a function to go to and I wanted to wear that dress, so now I will set a different goal that will be activity orientated, but I just haven’t decided what it is yet.

Facilitator:	That’s great, and the last thing that I just wanted to look at is about your satisfaction with the program. So based on your experience in this program do you think you’d recommend it to any of your other friends or anyone else in your social network?

Interviewee:	Yeah I’d definitely try and get some of my friends, I know a lot of my friends eat pretty well anyway but for some of them that don’t I would definitely recommend it because it was a self-pace thing as well. I thought it was great, it felt very personalised which was nice.

Facilitator:	Yeah that’s great, and in terms of the facilitator that ran the program do you have any preference whether that person was a local person from Port Fairy or whether they were somebody from Melbourne?

Interviewee:	I don’t have any preference but I think maybe if it’s not a local it might be easier for people who aren’t really confident.

Facilitator:	Yeah I agree.

Interviewee:	I don’t know, I think if it’s a local person in such a small community it’s a little bit awkward.

Facilitator:	Yeah of course, and again you sort of lack that privacy.

Interviewee:	Yeah.

Facilitator:	And is there anything – if you think about what makes a good facilitator, so the person that ran that first session, what traits do you think are important for somebody coming in – so this program is going to be rolled out across Victoria hopefully, what personality traits or factors do you think are important to make a good facilitator?

Interviewee:	I think they need to be very positive and very – a good speaker but also a very good listener, but very encouraging is a good thing they need to be.

Facilitator:	Yeah, someone else also mentioned about being sort of knowledgeable and practicing what they preach, what do you think about that?

Interviewee:	Yes definitely, yeah.

Facilitator:	Because we’ve had some mixed responses.

Interviewee:	You wouldn’t want somebody that’s 160 kilos to tell you to lose weight, definitely.

Facilitator:	No, it’s been interesting though, we’ve had some mixed responses. Some women found that it was off putting to have someone a healthy weight and some women found it very positive, it’s been interesting though.

Interviewee:	Yeah, yeah I mean you certainly probably wouldn’t feel as comfortable if it was some super athletes, but just a normal person not an overweight person I think is the main thing I think because I find that quite odd when you go to a doctor or the hospital and you see all these overweight medical staff, you think you shouldn’t be like that.

Facilitator:	No, you should practice what you preach.

Interviewee:	Yeah.

Facilitator:	And what about the venue? I think you came to the session at the school, correct?

Interviewee:	Yes I did.

Facilitator:	What did you think about that? We’ve had a lot of mixed responses about whether they thought it was an ideal venue, what are your thoughts?

Interviewee:	I didn’t find it a problem, it was just – we did it in the library, it was not a problem at all for me.

Facilitator:	Because some women just said they prefer it somewhere a bit more neutral, at a health centre or somewhere, if they didn’t have kids at the school they felt a bit uncomfortable.

Interviewee:	Yeah well I’ve got kids at the school so it was not a problem at all for me, but the people who – even people that didn’t go to that school, the other school, people would be going why should it be at this school – that’s true. But I mean I go to that school so it wasn’t a problem for me at all, it’s just easy.

Facilitator:	Yeah, what about group versus individual, do you have any preference of what you’d prefer?

Interviewee:	When I was first starting I think definitely individual, but now as more of a motivating thing it wouldn’t me being a group thing because that was all sort of very – it gives people a lot of encouragement, that if you go there and everybody has done well we can all support each other and become a little gang of people that have succeeded. But initially I think having that very private thing was a good thing.

Facilitator:	Yeah because some people said that they liked the group because it was a bit less threatening so it made them feel like other people were going through the same thing.

Interviewee:	Yeah, I don’t know about at the start though but...

Facilitator:	Yeah, but now that you’ve done well?

Interviewee:	Because of fear of failure that you mightn’t do well...

Facilitator:	Yeah.

Interviewee:	But certainly if you could get the people together that were doing it, which would be very hard, but even if it was just through social media that you got a little group of people that were all like a private group, like our Michelle Bridges Facebook group – we’re a little private group that can all talk to each other and go out and do stuff together, that’s very motivating.

Facilitator:	Yeah, so you think that would have been – if we could improve the program in the future you think maybe a bit more support between participants would have been useful?

Interviewee:	Yeah, and it can be very private like a Facebook private group that nobody else can see into or join into, then you’re all feeling confident that you’re all talking to each other but not everybody else in the world knows about it.

Facilitator:	Yeah and that’s a good thing, that’s something we should definitely think about.

Interviewee:	Yeah.

Facilitator:	Any other improvements or suggestions about how we could make this program better?

Interviewee:	No I think you’re doing a fantastic job. No I don’t think so, I think the only other thing that I can think of right at the very start – when I went up to the school and the lady was there handing out the envelopes I didn’t really know what I was grabbing, I just grabbed it because she was talking about being healthy and I grabbed it. I didn’t really know what it involved or anything like that, if you can get some information into like school newsletters or into the local papers before they came so everyone had more of an idea of what it entailed you’d probably get a bigger intake of people doing it.

Facilitator:	Yeah so you think maybe we needed more media or more...

Interviewee:	Yeah it was very sudden and I hadn’t heard anything beforehand, I don’t know if there was and I missed it but it was all very sudden and last minute – it felt very last minute and there wasn’t time for people to go I might do that.

Facilitator:	So maybe more time.

Interviewee:	It was just there one day gone the next sort of thing.

Facilitator:	Yeah so maybe more times or even more sessions. A lot of people have said that you often need some people to go and then tell their friends and then that would sort of increase numbers as well, so different sessions on different days.

Interviewee:	Yeah different sessions on different days and just having maybe before you came – because you’re coming from Melbourne down here, before you come down get something – ring up the school or wherever and just say we’re going to come down, we’re going to try and do this, put it in your newsletter or whatever at school so people know that that’s what we’re coming to do and then they’ll look out for you when you come. Maybe if they organise okay somebody else can pick the kids up from school so I can go and find out about it or whatever.

Facilitator:	Yeah, so how did you find out about the program?

Interviewee:	Well when I walked into school either one morning or one afternoon it might have been to pick up the kids there was a lady standing there handing out envelopes going this is what we’re doing.

Facilitator:	Yeah we must have seen you at the school grounds, yeah.

Interviewee:	Yeah.

Facilitator:	Okay, that’s a really good strategy, and were you satisfied with the information provided or is there anything else you would have liked – you mentioned a couple of things about the Michelle Bridges that you found useful, was there anything you’d recommend for us to put in our program?

Interviewee:	No, maybe the only thing I think is that sort of maybe communication between the people doing it so we’ve all got support within each other. I have no idea – still no idea of anybody else from our school that are doing it, and we’ve been doing it that long – I have no idea of the other people that are doing it. So to have that little support group would be nice I think.

Facilitator:	Yeah I agree.

Interviewee:	As long as everybody agreed to be part of it because some people want to be private about it but the people who didn’t – it’d be really good to have a little support group between us all where you all knew everybody was doing it and where you can go how are you going, what’s your goal, and blah, blah – just to make it a bit more of a challenge really even.

Facilitator:	And a bit more social as well.

Interviewee:	Yeah and definitely more social, and we can say okay let’s all go out and do something together on a Wednesday or whatever.

Facilitator:	Yeah that’s a really good idea. That’s great, that was pretty much all I wanted to ask you, everything you’ve provided is incredibly valuable and thank you so much for your time, is there anything else you think we need to know as part of our evaluation or anything else that you think is important for us to know?

Interviewee:	No I just hope you can get more people on board and get it going everywhere, it’s a really good idea.

Facilitator:	Perfect, and sorry the last thing I actually wanted to ask you – what about cost, do you think that would have been a barrier if we didn’t – if it cost to join the program?

Interviewee:	Well that would very much depend on the cost I think, and I think when you’ve got an unknown – like everyone would have to know what they got for their money, that’s all, I don’t think it’d be a problem as long as people had an idea of how much it was costing and what they were going to get for their money.

Facilitator:	And what do you think would be a reasonable fair cost?

Interviewee:	I have no idea.

Facilitator:	$10, $5?

Interviewee:	I don’t think anybody would balk at $10 or $5, I don’t think at all. If they knew what they were going to get for that then I don’t think that would be a problem, that sort of money. I mean the Michelle Bridges thing is $200 for a 12 week program.

Facilitator:	Yeah, so maybe...

Interviewee:	If it was that sort of money you’d have to really have it nice and organised, and really – that’s a lot of money compared with $5, $20, whatever. I think it would be harder to get people to take that envelope and agree to do it if they had to pay without actually knowing what they were going to get, whereas I just grabbed the envelope, went for the information and it wasn’t a problem. But if you wanted people to give you $5 before you got the envelope you probably wouldn’t get many people doing it.

Facilitator:	Yeah I agree, we need to make sure it’s up front and...

Interviewee:	Yeah so they know okay this is the information I’m going to get for my money, then it wouldn’t be a problem. You’d have to have a big information session and get people to sign up and pay their $5 then, it wouldn’t be a problem as long as you had the nice sessions beforehand.

Facilitator:	Yeah okay, perfect, thank you again so much for your time, everything you said is really useful to us, and we look forward to seeing you in a few months, the year is rolling around quite quickly...

Interviewee:	Very good.

End of transcript

Participant #AC

Facilitator:	And just to let you know in the letter that we sent you a while back I’m just wondering if it’s okay if we tape record the interview just to make sure that we accurately get down everything you say?

Interviewee:	Yes, no worries.

Facilitator:	Okay perfect. So to start off with I’m starting easy. I just want you to tell me a little bit about the access to fruit and vegetables in Beechworth?

Interviewee:	Well it’s reasonably good. You’ve got the major supermarket. Quality can be another thing I suppose and there also is the little fruit and veggie shop which is sort of like a specialised – well not specialised but you know they probably sell maybe a bit better quality or more local produce. Then of course you’ve got options of you know Wodonga, Myrtleford, Wang, those sort of things. So I’d say I mean it’s pretty good.

Facilitator:	What about in terms of exercise opportunities? Is there many options available?

Interviewee:	Oh it’s limited only by your inspiration I suppose or your desire.

Facilitator:	So there’s lots of things to do if you’re willing?

Interviewee:	Absolutely yes. Yes and probably more so if you’re in town a bit but then again you know you can get other people you know to do it with you or it depends yes how driven you are I think also yes.

Facilitator:	Yes and would you consider the town to be one that you know there’s lots of social connections and the community are quite – you know are quite a friendly …

Interviewee:	Yes I think so. Yes.

Facilitator:	And has there ever been a healthy lifestyle program like this available before that you’ve been aware of or that you’ve been involved in?

Interviewee:	Not that I know of no except your – you know your advertised programs that cost lots of money that people don’t have.

Facilitator:	Yes. Have you been in a program before?

Interviewee:	No.

Facilitator:	No and if you think back to when we came to Beechworth in about March earlier this year, do you remember what motivated you to come along to this program?

Interviewee:	Well to see basically what sort of resources or what sort of things would inspire and help me to keep going along my path I suppose.

Facilitator:	Yes and what’s your path?

Interviewee:	Just you know to be a bit fitter, lose a bit of weight and activity I suppose yes and eating like healthy meals and all that sort of – having that sort of dietary – appropriate dietary. Not necessarily inappropriate.

Facilitator:	Yes and do you think there were many factors that may have stopped other people from coming along on that day to that session in Beechworth?

Interviewee:	Look there probably was you know a lot of mums work and some might not have heard about it. Yes I mean yes.

Facilitator:	If we think back to that first session, we had that big group session when we gave a bit of healthy lifestyle advice. In that session we talked about sort of general healthy life advice. We had those six messages you know about reducing soft drink, eating breakfast and then we talked about problem solving and goal setting and you know strategies to help achieve behaviour change …

Interviewee:	Yes.

Facilitator:	Was any of that information new to you or mainly quite familiar do you think?

Interviewee:	No. I think it’s just some motivation and desire to put it into practice or the you know the constant reminder or you know the group support the you know let’s keep it going or have you been doing this sort of thing so …

Facilitator:	And you’ve mentioned motivation, what’s that like for you?

Interviewee:	Pretty high when we’ve got you know we’ve got our family. We’re not having any more and you know there’s no more excuses not to you know just sort of lounge around well not that you lounge around but you know not to be more physically active and fitter because the kids need fit, well reasonably fit parents, if they want to be involved with you know we’ve always – the kids – the youngest one’s just learning to ride bikes so you know we go for bike rides and go for walks or go for a few other things yes.

Facilitator:	So I guess is it fair to say that what’s driving you is to be there for your children and to improve your health? Is that what – is that why being fit’s important to you?

Interviewee:	And for my own – and for me you know it was before when the girls were little you didn’t have time to dedicate to yourself and I enjoy you know my bike riding, my walking, running and my circuit stuff and that’s me time if you know what I mean. Yes so …

Facilitator:	And what’s …

Interviewee:	Yes.

Facilitator:	Are there any obvious things that are obstacles that you’ve found that make it hard to be as active or to eat as well as you’d like to?

Interviewee:	Time and money. The old – the same old same old really. Time and money. Yes.

Facilitator:	Yes and just to, sorry, just to clarify it sounds like a lot of what we talked about at the session was quite familiar to you. Is that correct?

Interviewee:	I think so yes. We’re all pretty much in the same boat.

Facilitator:	Yes so that group support was useful in learning that you were all in the same boat?

Interviewee:	Yes and I believe that for me, something like the group thing, an ongoing sort of program like that would be far more beneficial support wise and just community wise and all that sort of thing so I think yes rather than we sort of just disbanded and it hadn’t really gone anywhere and I suppose it’s probably up to us as well to sort of I don’t know let’s get together blah, blah, blah but yes it just didn’t translate to that sort of way so …

Facilitator:	Yes and you’re not alone in saying that. A lot of people have said that. Would that be helpful for you to have that ongoing regular group meeting?

Interviewee:	I think so or you know it doesn’t have to be a meeting it can just be a catch up oh you know, let’s go you know I don’t know we can do something or somewhere all the kids can go because most of us have all got kids. Somewhere that doesn’t have to be during the week because most of us work and we’re you know on short timelines, that sort of thing. So yes I mean there’s so many factors to consider. You can’t have them all you know covered but yes.

Facilitator:	Yes so to have a bit of that support from the whole – from the other women in the program?

Interviewee:	Absolutely yes. Yes.

Facilitator:	Yes and if you were to describe the program or if you were to think about what the take-home message of sorry – was from this program what do you think that was?

Interviewee:	Aim to be healthy. Aim – yes. Aim for longevity of life and sort of be conscious about your choices.

Facilitator:	Yes and when you mentioned being conscious of your choices, do you think by again by being completely honest because we want as much feedback as we can get, but do you think by being involved in this program it’s made you think more or less or no change in what you’re eating, about your weight, about your exercising and has it had any influence?

Interviewee:	It’s probably like a nagging thought if you know what I mean. It’s not necessarily you’ve actually done – made any changes but it’s there that you know you should or that sort of thing so I’d say that’s probably the main impact it’s had on me and you can say that that sort of nagging thought will eventually sink in and help – up on the bench sweetheart – might actually on that particular day you’ll go no, no I’m going to follow that nagging thought or you know I’m going to start no it’s right sort of thing.

Facilitator:	Yes so would it be fair to say, a couple of women I’ve spoken to said that the program’s made them more aware of what they should do but they’ve sort of struggled potentially with that activating the change? Is that something that you can associate with?

Interviewee:	Yes because one of my goals was to eat more fruit and I can’t necessarily say that I’ve done that but I guess – mummy’s on the phone sweetheart so if you go over there and have a look. Thanks babe - ask dad. Where was I going with that? Yes.

Facilitator:	You were talking about fruit so you said you’ve been thinking about it?

Interviewee:	Yes what my goal was and yes you know it’s probably just been in the last couple of weeks that I probably have actually increase and reduced – you know increased the fruit or whatever and reduced the crap so to speak so I mean it’s probably been what was it, March you said? So but that nagging little thought you know obviously it being there and it’s taken a while to root itself in my brain but yes you know yes.

Facilitator:	Yes but now it’s kicked in?

Interviewee:	Yes it’s slowly – it’s still a gradual process. A slow, gradual process so yes any change I suppose is better than none.

Facilitator:	Yes and in terms of the program itself, we had as – we had different components. We obviously had that group session back in Beechworth at the school then we had – we gave you a program manual – you’ve had some SMS text messages …

Interviewee:	Yes.

Facilitator:	The website and also someone called you not so long ago just to touch base and to help set some goals with you on the phone so sort of a bit of a phone coaching session.

Interviewee:	Yes.

Facilitator:	Did anything stand out as being more useful to you or the most useful?

Interviewee:	The most useful? I suppose the text messages were good because it was just like a small prompt but the manual, I think I flicked through it the afternoon I come home and haven’t touched it since. Yes but I think yes the regular interaction with the people that are going through it as well would definitely be far more motivating and inspiring than yes – but again the text messages sort of kept it in your memory I suppose and you sort of let it fade into the background into like it never happens.

Facilitator:	Yes so it sounds like the face to face is the best source of contact for you. Is that correct?

Interviewee:	Yes I think so definitely.

Facilitator:	What about the phone call? Did you find that useful or is that something that if …

Interviewee:	Oh I think it just seemed to reiterate what we’ve been over and you know I didn’t really find it very helpful at all. It just yes it didn’t change anything anyway.

Facilitator:	Yes so what’s – I know this can be a little bit of a hard question, but what’s made you change in the last couple of weeks versus back in March or previously do you think?

Interviewee:	Drive and desire. I think you just get to a stage where you go that is it. I’ve had enough and I’m just doing it.

Facilitator:	Yes and I know – I don’t mean to pry …

Interviewee:	No that’s okay.

Facilitator:	But did something happen? I mean – what’s – why now? Like what’s it …

Interviewee:	I don’t know. Don’t know oh well I suppose I’ve been you know increased physical activity. I haven’t necessarily changed dietary habits and I guess I’ve – oh probably a while ago come to the realisation that if you don’t change - like I’ve come so far with physical activity so if I want to improve say weight or whatever any more then it’s going to have to incorporate dietary components so okay so it’s like suck it up princess, here we go.

Facilitator:	Yes so it sounds like you – the exercise has been something you’ve always been in – you’ve been in control of for a while is that correct?

Interviewee:	Yes well that’s been – it’s always been but then the kids came along and then you don’t get a chance and probably this is my third year so I’ve been you know it was every second day, now it’s three days a week sort of thing so we try to maintain that but to – you can – the results to go further it needs to be complimented by dietary changes and that sort of thing so …

Facilitator:	And has that motivation – so is it coming from then you or is it something else?

Interviewee:	Yes. Well no one else is going to do it for me.

Facilitator:	Yes and just to – I mean you touched on this a minute ago but based on – since attending this program are there any specific changes that you’ve made? You mentioned that you’ve increased your fruit and you’ve reduced the junk. Is that a result of the program or is that just a result of you?

Interviewee:	Oh it probably can be attributed to that. I think it’s just you know you get to the stage where you know you think I don’t know but yes. Well if you want to achieve something, these are the steps you’ve got to take and are you prepared to do it? Yes, okay. When – okay now. So …

Facilitator:	Yes so it’s yes. From …

Interviewee:	We all know what we need to do but it’s just a matter of doing it. Being driven enough to you know want it and all that sort of thing and have others who are in similar situations go yes we’re doing it and it’s like yes, cool, okay I’m in. So it’s like a bit of group therapy.

Facilitator:	Yes and have you got others, sorry, at the moment now trying to do something as well?

Interviewee:	Oh well when I first started say three years ago, I was doing my exercise program I suppose you’d call it, we’ll call it a program, by myself and then I’ve had friends come and go but I’ve had a friend who’s and been doing it probably this is her second year I think and then there’s a group of us that do once a week and then anyone you know in between. They do whatever in between sort of thing so yes.

Facilitator:	Is it an exercise program you’re talking about?

Interviewee:	No it’s just well no not really. It’s just being active and in the Herald Sun they used to have the Body and Soul section on Sundays and they’d have like a circuit program so we just go to the stage where we’d take bits and pieces of that and we’d do that three or four days a week and now we’ve just got to a certain stage where now it’s just three so yes but that does – there’s not just circuit it’s walking or interval, running, that sort of stuff so …

Facilitator:	Yes and I’ve touched on this and I keep repeating myself but …

Interviewee:	Yes.

Facilitator:	So since being in the program do you think you’ve made any changes that have been sustainable or not so much?

Interviewee:	Well I hope to sustain the fruit intake.

Facilitator:	From now?

Interviewee:	Oh yes and you know you I guess you hear people that once they make up their mind then you know, they’re quite happy to stick to it. They might sort of waiver here and there but you know they’re – essentially that’s what they want to do so that’s what they’ll do.

Facilitator:	Yes and the three types of – and sorry, the exercise three times a week, that was happening since before you were in the program?

Interviewee:	Yes.

Facilitator:	Okay so mainly the biggest change sorry is the increase of fruit and the reduced junk food?

Interviewee:	Yes.

Facilitator:	Great and is there any other factors? You mentioned that your friends have helped you know with the exercise. You mentioned that your drive has helped to get that fruit in. Anything else?

Interviewee:	Yes. No that’s pretty much the core elements I think yes.

Facilitator:	And in terms of those behaviour change strategies like we talked at the session about goal settings, problem solving, have you used any of that to help get that fruit in or is it just something that you just did?

Interviewee:	It’s probably just the goals you know and yes you know what steps you need to take so yes.

Facilitator:	Yes and in terms of what we’ve been looking at with everyone is about the reach of this program so we’re trying to work out by if you – by you being involved in this program, whether it’s had any influence on your family, your children, your partner. Do you think that by you being in this program has had any flow-on effects?

Interviewee:	I think it would probably most definitely because if you don’t – if you’re changing your dietary then you’re also I mean being the shopper and cooker and all that sort of stuff, then if it’s yes you direct or guide I suppose what is consumed and what isn’t consumed; quantities and all that sort of thing. So yes I think to make you more conscious of yes what you’re bringing in and what you’re putting out on the table and that sort of thing. So what you’re allowing them to consume.

Facilitator:	Yes so any …

Interviewee:	And increasing their activity as well.

Facilitator:	Any examples of how you’ve influenced your family since being in this program?

Interviewee:	Well I suppose the increased activity I mean we’ve always been a fairly active sort of family like the bike riding and the walking and you know that sort of sporty type things and the kids are involved in and that sort of stuff and I suppose shopping wise you focus – you don’t tend to buy so much of the – I mean I tend to cook at home a lot anyway because all the stuff’s too expensive but you know being aware that okay well this is allocated for this week. It’s not just for the next two days, it’s got to do the week sort of thing you know. Yes.

Facilitator:	Yes but sorry because it sounds like that most of the changes you are doing in terms of the exercise that was something that hasn’t really been influenced by the program? Correct?

Interviewee:	Oh but it can be more inspiring to know that you know that and that’s what it’s all about too I think you know you’ve got the idea and the concept but without the drive and the reminder and the you know that sort of thing that can sort of just sort of disappear as quickly as it came if you know what I mean.

Facilitator:	Yes so it’s helped sustain it for you perhaps?

Interviewee:	Yes. Every little bit helps.

Facilitator:	Yes and have you spoken to anybody else in the program about how you’ve been going or have you found any support from anybody else in the program?

Interviewee:	Oh look there’s the odd conversation as you’re walking past, in between dropping off and doing whatever and that sort of thing but as to you know a full on conversation or you know a D and M about it, no. .

Facilitator:	No? That’s okay and do you feel supported by your family or have they helped to make you make changes?

Interviewee:	Yes. They like going for bike rides.

Facilitator:	Good so they’re all – everyone’s getting in together?

Interviewee:	Oh yes, yes.

Facilitator:	Yes and do you think by you being in this program, again, it’s just a low intensity program so we’re not expecting any large changes but do you think you’ve had any influence on any of your other friends outside the program or in the program?

Interviewee:	Maybe a marginal but yes I mean yes we just do what we do so yes activity wise and that I mean we’re always encouraging people with you know friends or whoever to come along and do it with us. We’re sort of not skill based if you know what I mean. You’re not going to have a certain level of fitness to come along with us like you’ve got to yes …

Facilitator:	Yes and since being in this program how do you feel about your progress? Do you feel you’ve done well, you’d like to do more, how are you feeling about …

Interviewee:	I’d like to have done better. I don’t think I’ve done – I haven’t been a scholar student that’s for sure. Probably the grade that could use more effort that’s what I’d put myself at.

Facilitator:	Yes. What about your weight, have you been weighing yourself?

Interviewee:	But in saying that you know …

Facilitator:	Have you been weighing yourself?

Interviewee:	You know, hey?

Facilitator:	Sorry I missed what you just said.

Interviewee:	Oh I just I yes put in some more effort, just saying that you know towards the last couple of weeks it has been increased consciousness about it all and it’s like right. Roll up your sleeves, let’s go.

Facilitator:	That’s good to hear.

Interviewee:	Yes.

Facilitator:	And have you been weighing yourself or have you found that helpful or not helpful?

Interviewee:	No. Well I don’t really have access to a set of scales and I’d probably be on there every second day if I did so I usually just go off to the doctor’s surgery and jump on when I’m out there or around and that sort of thing. So I probably am keen to do that next week or so only because yes, yes that’s where I’m at but you know you feel you know – and it doesn’t necessarily – I think that can be sort of a negative thing, whether you see a change or you don’t see a change and I think you can tell when you’re putting your clothes on or you know that because you haven’t been having that you’re doing all right anyway.

Facilitator:	Yes and in terms of the last thing I wanted to ask you was about your satisfaction with this program so based on being involved in this program do you think that it’s something that you’d recommend to other people in the community or to any other – any of your other friends?

Interviewee:	I think if there were maybe some changes like a group you know catching up and I think the text messages are pretty good. You sort of go, oh God, them again but you know like I say, it does prompt you into keeping that in your memory bank if you know what I mean. Yes the booklet a complete and utter waste of time for me because I didn’t read it.

Facilitator:	Is it …

Interviewee:	Yes I mean I think a more person to person approach and it won’t I mean if you guys were the ones to get the ball rolling and then let the group like generate the momentum if you know what I mean, we can be the you know like SMS support and then maybe coming up every three months or something and address the group or you know. I don’t know do something like that but yes it yes.

Facilitator:	One lady mentioned that she thought like a group Facebook would be ideal because then she could keep in touch with other women in the program and organise a few you know like you mentioned that you do your program in the park. You know you could get people …

Interviewee:	Yes.

Facilitator:	Other people to come along. Is that something you think would be useful?

Interviewee:	Not for me because I’m not on Facebook.

Facilitator:	No.

Interviewee:	A complete an utter waste of time. I mean it’s good – it’s a good medium don’t get me wrong but yes, I just – I don’t have time and yes I’ve got better things to do then sit on the computer and …

Facilitator:	Yes but that kind of idea of keeping in touch with everyone and meeting up as a group would appeal to you?

Interviewee:	Definitely.

Facilitator:	Okay perfect and in terms of that facilitator, do you have any preference whether she’s a local lady or a lady from Melbourne? Any …

Interviewee:	Well I guess if it was a local person you’d have to sort of maybe see who it was. Yes it depends who it was. Yes.

Facilitator:	What about the venue? Any – was that an okay venue at the school or …

Interviewee:	Outdoor, indoors, either, either. It could be catching up before a walk around the gorge or you know blah, blah, blah.

Facilitator:	That could have been a good idea. A group walk prior – before the session and what about group versus individual? Do you have any preference of how you’d like to receive your support?

Interviewee:	What do you mean by that sorry?

Facilitator:	Would you prefer if that session was a one on one or did you like the group or …

Interviewee:	Oh no, no one in all in, I say yes.

Facilitator:	So the group?

Interviewee:	Yes.

Facilitator:	Yes and in terms of the information provided, was that okay or did you want more information?

Interviewee:	I don’t know. I was up at the kinder today and they’ve got this program going and it’s all about healthy eating and stuff like that and it’s just - it’s visualising what it is that you should be doing. You know like we’re talking about healthy eating and stuff like that and it’s like yes that’s good and you can read a chart until the cows come home but until you can visually quantify that okay there’s x amount of fruit there on the table, there’s and you can distribute that for this time and this time during the day and there’s that particular fruit or you know it’s all about visually quantifying it for me as well, to help improve the – and yes and that was funny that you – yes you’re doing this questionnaire today because it was yes quite a valuable exercise up there for the kinder so yes.

Facilitator:	Yes so it sounds like you’re a bit of a visual learner?

Interviewee:	Absolutely yes, yes and you know visual quantifying.

Facilitator:	So you want – you know eat three pieces of fruit or eat two so that kind of learning’s good for you?

Interviewee:	Yes and varied combination as well you know it’s like it’s all yes. It’s all about going back to grass roots for us mums because you know we learnt from you know our parents and they’ve learnt from their parents and it’s a hard nut to crack to try and you know alter learnt, engrained habits shall we say yes.

Facilitator:	Are there some of those engrained habits that you want to change?

Interviewee:	Yes, yes but it’s all about knowledge I think and you know like I said, being able to visually quantify it and say okay, well this is good, this is appropriate, this is adequate, anything over and above that is you know, is not necessary and not that good for you blah, blah, blah so …

Facilitator:	Yes and in terms of you mentioned that you wanted more support, how – and you said that you prefer it sort of face to face in terms of the group. How often would you have liked that group to meet up?

Interviewee:	Oh once a month?

Facilitator:	Once a month? Yes.

Interviewee:	Yes minimum. I mean you don’t want to get sick of each other.

Facilitator:	Of course and in terms of your expectations, again, being honest, do you think by being involved in this were your expectations met or did you have any other different …

Interviewee:	No.

Facilitator:	Expectations of what you wanted to achieve or …

Interviewee:	Yes I’d say they probably weren’t met but they were probably different but as to what exactly it was I don’t know. Yes because I just wanted to participate and see where – I guess where it took me I suppose so …

Facilitator:	Yes but you’re not exactly sure what you were looking for?

Interviewee:	Whatever it was I didn’t necessarily get it but you know in saying that, I got that constant reminder to be more conscious of being healthy and you know it’s taken a few months but you know something has clicked and it has sort of had an impact and you know it’s all interlinked. You know you’ve got to have you know a sort of component of each of it going around to sort of yes get you going.

Facilitator:	Yes and last question, is there anything else that you would recommend that we do to help improve this program in the future?

Interviewee:	I think just encourage them to – the participants to sort of interact. You know and you might find that you have okay you’ve got your once a month meeting sort of thing but then you know some might be friends so they catch up once a week or some might become friends and catch up you know here, there, more frequently, that sort of thing so yes I think that yes that sort of thing would be far more beneficial for a lot of people I think yes.

Facilitator:	Yes and one person said that yes, they were quite happy for the group to catch up without the university you know, without the facilitators.

Interviewee:	Yes.

Facilitator:	Would you be happy with that or would you prefer the facilitators?

Interviewee:	Oh yes because it would be something that would develop itself you know. You’re sort of given the drive and the tools or whatever or you know the resource could contact you or for you to email or do whatever, SMS, but yes so it’s you know you can hold your hands – hold people’s hands but sometimes it’s to a certain extent. But you know ultimately it’s up to the individual or whatever their reasons are or whatever.

Facilitator:	Yes so maybe yes get the – just get the participants to meet up themselves and one person also suggested you know having someone from the local health centre involved. Is that something that you think would be okay or …

Interviewee:	Yes absolutely yes.

Facilitator:	Okay perfect. Is there anything else that you think we should capture in our evaluation or anything else you wanted to add?

Interviewee:	No I think that’s pretty much – yes. Pretty much covers it. Yes I think the group thing’s very important, the SMSs are good, yes and yes I think it’s a worthwhile thing to be doing but yes as you do more and as you get more feedback in sort of the – improved and streamlined and all that sort of thing so it does have more of an impact. Yes because there are lots of people out there that aren’t necessarily involved but you know maybe next time round would be and or wished they had have been and that sort of thing so yes you constantly sort of being around gives people food for thought as to like, maybe I should do it this time. Or you know maybe it would be a good idea or you know …

Facilitator:	So maybe holding it again could be useful at another time to get people involved?

Interviewee:	Yes, yes absolutely and then promoting the fact that you have been here and I mean probably like varied results like I’d probably say, yes have a go but someone who really went the full nine yards with it who has dramatically changed and has proved out of sight would absolutely positively rave about it so your intake would be higher again so …

Facilitator:	So it sounds like that word of mouth is very important?

Interviewee:	Oh in a rural setting absolutely yes, absolutely.

Facilitator:	Any other ways to get more people involved?

Interviewee:	If you were to advertise like if you were to – in school newsletters, kinder newsletters, localise it you know because yes that’s a very important medium of communication. Parents who drop off and pick up and don’t get to read the newsletter on the email or just pick it out of the kid’s bag and just flick through it while they’re having a cup of tea before they start the next round of jobs to do and you know when they get home from work or you know they get to catch up on at the weekend sort of thing yes.

Facilitator:	Okay and this is actually the last question. I just wanted you to confirm your address if you could please, just so that we could – can send you that gift card. What was your address again sorry?

Interviewee:	Oh okay yes.

Facilitator:	Is it 18 Mellish …

Interviewee:	18 - 18 Mellish.

Facilitator:	It’s double L sorry?

Interviewee:	M-E-L-L-I-S-H.

Facilitator:	Okay perfect. Well thank you …

Interviewee:	Oh Brian I can’t hear you. Sorry, my husband’s having his input now.

Facilitator:	Sorry. Any feedback’s welcome.

Interviewee:	No. He wants to know if you want him – if you want to talk to him. She doesn’t want to talk to you. Go away.

Facilitator:	We’re happy to talk to him if he’s got something he wants to add but …

Interviewee:	Yes. No. Nothing constructive I don’t think.

Facilitator:	But thank you again so much for your time …

Interviewee:	No worries.

Facilitator:	And I’m really glad to hear that your motivation’s picked up a bit because that’s really good to hear but if you’re requiring any more support or you want more tips or help, please just give us a call and we’re happy to talk to you. We’re always here.

Interviewee:	Yes. No worries. Well you guys are coming up …

Facilitator:	We’re coming in March, back again at the end of the year.

Interviewee:	Oh but in 12 months from the date yes okay cool.

Facilitator:	Yes from the date and …

Interviewee:	We’ll …

Facilitator:	Yes besides that …

Interviewee:	…especially with all your SMSs and things and yes we’ll probably catch you in March then.

Facilitator:	Yes. We look forward to seeing you in March and thank you again for your time and if you don’t receive that gift card in the next week or so, please let us know.

Interviewee:	No worries.

Facilitator:	Perfect. Thank you so much again.

Interviewee:	No worries

Facilitator:	Thank you.

Interviewee:	Okay bye.

Facilitator:	Bye.

End of transcript

Participant #NJ

Facilitator:	I think I mentioned to you earlier the point of the evaluation is just to get your insight about the program and what you found useful and how we could improve it in the future. Everything you say will be confidential and the whole point is that we want you to be as honest as possible because we want to know what you liked and what you didn’t like about the program, yes?

To start off with we’re comparing the different towns to each other in terms of fruit and vegetable access and opportunities to exercise. Do you think that Port Fairy has good access to fruit and vegetables and opportunities to exercise?

Interviewee:	Yes I do.

Facilitator:	Overall do you think – do you view it as an active community?

Interviewee:	It’s quite an ageing community but you know, I’m – I’d probably say, yes. I mean I certainly know there’s heaps of people do swimming, like kids particularly and that kind of stuff and I would say it’s reasonably active - average.

Facilitator:	What about in terms of opportunities to socialise? Some communities we’ve been to seem very connected and others not so much. What’s your thoughts on that?

Interviewee:	I mean I think there are opportunities to socialise. I think like anywhere it’s what you make of things as well. You can either get involved or not but I do think there are things that you can do and I think you – I mean even if – I’ve got young children so obviously you get to know other parents and those sorts of things, but you know, they have things like friendship nights for people who do feel socially isolated and things about, you know, three or four times a year and that kind of stuff.

Facilitator:	Oh that’s good.

Interviewee:	Yeah.

Facilitator:	Has a program like this ever been available in Port Fairy before - a healthy lifestyle research program?

Interviewee:	Not to my knowledge.

Facilitator:	If you think back to when we started the program back in October, November last year do you remember what motivated you to join this program?

Interviewee:	Well I think that I honestly just thought that it was probably hard to get people to do it and I thought – I also thought that, you know, it would be good for me and, you know…

Facilitator:	Come along?

Interviewee:	Improve my life. Yes that’s probably it.

Facilitator:	When you say improve your life, what do you mean by that exactly?

Interviewee:	Well I mean, you know, more health and, you know, if you’re healthy you manage things better and cope with things better and all that kind of stuff. Yes.

Facilitator:	As you said it was a bit difficult to get people to come along to the program. Is there anything that stood out to you as a potential barrier to join this program?

Interviewee:	Look I don’t think so. I just think that we – it can be a bit apathetic and you know, those sorts of things – in terms of – I mean – oh, we couldn’t be bothered and blah blah. I don’t think it was this specific program I just…

Facilitator:	Any other factors that you think would stop other people from attending?

Interviewee:	I mean I can’t remember. Was there a day and a night time thing offered?

Facilitator:	Yes there was a night and a day.

Interviewee:	Yes – no – well if there was both of them then well I sort of think well – so…

Facilitator:	One woman mentioned that they thought the reason why people wouldn’t attend is because they felt a bit self-conscious in a smaller community sort of coming forward and joining a program that was about weight. Do you think that could have been a problem?

Interviewee:	Oh look it wasn’t a problem for me. I could see that it could be a disadvantage to – for, you know – because everybody knows everybody and those sorts of things but, you know, I think that would have been an individual kind of thing.

Facilitator:	In terms of since we’ve seen you - what we’re looking at is to find out whether this program helped improve your – if you learnt anything from the program? The program covered general health information about nutrition and exercise and then focused on behaviour change strategies so goal setting. We developed action plans with everyone. Was any of this information new to you?

Interviewee:	Look I think some of it was new or presented slightly differently or things like that and you know, there – I mean I have to say I didn’t take very good advantage of the program at all. I’m a big failure…

Facilitator:	No don’t…

Interviewee:	You know, but they did say, you know, you needed to know about your failures as well. So yes I think there was some new information even things like some of the apps you can get for your phone and that kind of stuff and you know, I’m not saying I got any of those but I thought that there was a lot of interest shown in that sort of thing and that other people that are more onto it may have done that, you know. So I do think there was, you know, some new information provided and it was simple and well-structured and that kind of stuff so yes.

Facilitator:	Was any of the information a bit of a refresher for you or mainly new?

Interviewee:	Oh yes. No there was - refresher as well so.

Facilitator:	Did you find that valuable?

Interviewee:	Yes I always think it’s good to be reinforced - so.

Facilitator:	In terms of your overall impressions about the program - about the program aims, what did you view as the aim of this program do you think?

Interviewee:	I – well I think it’s about improving – reducing obesity and improving general health and lifestyle. I mean that’s what - you know, the impression that I got was that, you know, people got healthier habits and healthier eating and more regular exercise and those sorts of things.

Facilitator:	Yes I agree and I guess that was the whole point. Just to – yes, take charge and improve your lifestyle. In terms of healthy lifestyle, what do you think a healthy lifestyle means to you?

Interviewee:	It means eating enough fruit and vegetables. Not having too much junk. Getting regular exercise - you know, managing stress, that kind of thing.

Facilitator:	What would motivate you to achieve a healthy lifestyle? So some people have talked about role modelling for their family. Others have talked about, you know, keeping up with their children. What motivates you do you think?

Interviewee:	I think that for me, I have to get to such a point of self-disgust that I all of a sudden think, you know, get – kick myself into gear so I don’t know – I’m trying to motivate myself. I’m not getting very far. I’m buying healthy magazines and things like that and – got, you know, subscribed to Healthy Mind and Healthy Body on Facebook and things like that and I sit there and I read the things and think, right. I’m going to do stuff – I’m going to do stuff, but it’s not happening very often so.

Facilitator:	Is there something that we could do to help improve your motivation? Is it – would more support help you or is there something that we could provide?

Interviewee:	I really don’t know. I mean I do think that, - you know, when I’ve had – when a text has come through I’ve thought, oh shit I should do something about that and those sorts of things but, you know, I sort of - I let life get in the way and I mean – you know one of the things that you talked about in the thing was about planning and that kind of stuff and I’m not there – haven’t been doing that – been doing that well at all. So yes - and I’m not quite sure what – yes. I know I need to but I’m not. You know what I mean? I don’t know what would motivate me to actually do it.

Facilitator:	But since starting about – since starting the program do you think it’s made you think a little bit more about your weight or about exercise about your health?

Interviewee:	Look I definitely think it has made me think a lot more about it because even though I haven’t – I’ve lost like about three kilos in all that time.

Facilitator:	That’s still great.

Interviewee:	Nothing - so you know, it’s – I don’t know. It – I certainly think about it a lot more as I said I started buying these magazines, I got on to these things and that kind of stuff and you know and – I’ve started seeing an osteopath and so – started doing yoga. So I mean there are things that I have…

Facilitator:	Done?

Interviewee:	Changed but it’s not – I haven’t done what I’ve wanted to do if that makes sense.

Facilitator:	Yes that makes perfect sense and in terms of value of specific components – the components of the program so we’ve had, as you’ve mentioned the text messages. We had that one group session and we’ve had – we gave you the manual and also a couple of weeks ago you were called just to set some goals with a person on the phone. Have you found any parts of the program more motivating or more valuable than others?

Interviewee:	I would say that initially I responded more to the texts and things than I did as time went on. So, you know, I mean maybe even another face to face…

Facilitator:	Yes would have helped?

Interviewee:	Halfway through might have made a difference because, you know, it sort of, you know – and like I mean - I don’t think it’s the program’s fault I’m unmotivated but I do think it has made me make some changes in my life but not really enough so yes. So I don’t know if another face to face or something – and I know when you do Weight Watchers it’s that having to go weigh-in that motivates you. So perhaps if there was a bit of that - having to go front with other people there and confess might make a difference but then I don’t know - yes.

Facilitator:	Did you find the program manual useful?

Interviewee:	Look I mean – I have to say I use the manual very little. When I first got it I thought, that will be great, I’m going to go through that and be motivated and set – finish setting my goals and this that and the other and I never got around to doing it. I think that if I did that it probably would have made a difference because I do think it was very simply set out and also in the sessions they were saying what a difference it made when people, you know, really did that so yes.

Facilitator:	if we were to take away any of the program components, is there anything that would – that would lead you – at a similar motivation level to you are now? That if we didn’t have one component it would make a difference? So if we didn’t have the manual do you think you’d be where you are or if we didn’t have the text messages - if you understand what I mean?

Interviewee:	No I don’t think you should take away any of the program. I think that it’s those little things that at least – getting you going that tiny little bit and if they weren’t those sort of drop-ins and reminders that, you know, really what they do is make you feel guilty. You think oh crap! I should be doing something more with that and I don’t think that’s a bad thing - you know, so I wouldn’t be, - I don’t think there’s anything in there that I’d be saying, oh you’d be better off without that, so…

Facilitator:	Okay and you did mention that you have changed some of your – you have made some changes to your behaviour. What have you actually changed did you say?

Interviewee:	I’m doing yoga once a week now.

Facilitator:	Great.

Interviewee:	I’m seeing an osteopath for, you know, general health – I’ve got a bad back and things like that. I’ve started buying Prevention Magazines which – I don’t know if you’re aware of that but it’s a health and lifestyle magazine aimed – it’s aimed at people around my age, you know, thirties up. I’m in my mid-forties but I mean it’s not like you get some magazines and they’re all these young sexy girls and you think that’s got nothing to do with me. This is showing like, you know, woman in my age group and you know, people with kids and jobs and that kind of stuff are managing to do it and so it’s sort of realistic I think and so really it’s to try and encourage myself and I have sort of linked onto a couple of you know, healthy sites on Facebook and I’m – I thought I was really good with my fruit but something that the thing did show me was that I’m not always as good. I tend to eat lots of fruit or hardly any. So I’m trying to make sure – I’m trying to do that apple a day kind of thing so at least I get – I usually get some fruit occasionally – I can’t believe it’s hailing when it was like nearly 30 degrees two days ago!

Facilitator:	Looks bleak.

Interviewee:	Yes - so I don’t know.

Facilitator:	More changes to your physical activity it sounds like?

Interviewee:	Small changes to my physical activity. What I am doing more of is what I call incidental exercise.

Facilitator:	Great.

Interviewee:	So when I’m taking the kids to swimming instead of sitting and you know, yapping to the other mums I’m getting in the pool and you know, I’m not like doing laps and things as I’m carting a baby with me but I’m walking up and down with her in my arms and one of the ladies I talk to on the Monday night where my daughter swims she’s been getting in the pool with me now and so – and we just walk up and down the pool talking to each other instead of sitting doing nothing talking to each other. So I figure I’m trying to do more of that sort of, you know, things that – it’s not like I’m going out and going to the gym but taking more advantage of, you know, yes - that incidental exercise stuff.

Facilitator:	That’s great and is that – are these changes as a result of being in the program or since you started the program?

Interviewee:	They’re since I started the program. I don’t know if they’re just a result of being in the program because, you know, as I said I’ve, you know, started reading the magazine, I’ve started – you know, but I suppose doing those things have been since I started in the program anyhow so yes.

Facilitator:	Do you find that social support is important for you to make these changes?

Interviewee:	I probably haven’t take as much advantage of social support as I should and I probably don’t feel that my husband’s as supportive – he’s supportive enough about me getting healthy and whatever but as long as it doesn’t impact on his – you know what I mean. You know, I probably need to – I do need to sit down and say well listen here, I need, you know, you to get up earlier and have the kids so I can do this or in case [unclear 0:16:00.6].

Facilitator:	Yes and that would help. Have you used any of those behaviour strategies that we talked about? So we talked about problem solving, goal setting – is any of that – have you used any of that information?

Interviewee:	Not really no.

Facilitator:	That’s okay, no problem and a lot of people find that for them they rather just do something rather than, you know, really think about it. The next thing I wanted to have a quick look at is about the program reach. So what we’re interested in is finding out whether this program as a result of you being in this program whether you’ve influenced anyone else’s behaviour? Whether that’s part of you know, your family, your friends – have you talked much about this program with your family or with your friends?

Interviewee:	I would say very, very little. Yes – I’m not…

Facilitator:	So would you say you would have influenced your family or friends behaviour since being in the program?

Interviewee:	Not really no.

Facilitator:	That’s no problem. Do you feel comfortable talking…?

Interviewee:	I’d like to say yes but I probably wouldn’t be being honest.

Facilitator:	No, no and the whole point is to be honest so there’s no right or wrong answer. A lot of people say things because they think that’s what we want to hear but that’s absolutely no problem. Do you feel comfortable talking about your health with your friends, family?

Interviewee:	Oh look probably with most of them, you know, I – it’s – I – it probably depends on the friends to some extent too. You know, I mean I’ve got some friends I can tell absolutely everything to and others that yes I – yes wouldn’t.

Facilitator:	Yes of course and some people said, you know, they haven’t spoken to their friends because they’re quite private or they felt a bit self-conscious so there’ll be a lot of different reasons as to why people do or don’t.

The last thing I wanted to look at is about your satisfaction with the program. So again, being completely honest based on your experience in this healthy lifestyle program would you recommend this to anybody else in your town?

Interviewee:	Look I would recommend it to other people. I think that the, you know, not having done a lot has been me. I don’t think that it’s the program and look I mean – and you know, I think lots of people – you know, different things work better for different people and that kind of stuff you know and I still think if you sort of sat down and gone through the book and done all the things you should of – I mean I probably would’ve been further along than I am and so I would recommend it to the others. Yes because I think if they did that it would make a difference.

Facilitator:	Yes so if we were to change anything about the program – you mentioned that more contact might help motivate you. Is there anything else?

Interviewee	Not that I can think of off the top of my head.

Facilitator:	What about the facilitator? Would you have a preference on whether the facilitator is from someone – from a local person or someone from Melbourne?

Interviewee:	I only – I don’t think it can make any difference unless there’s further contact. So, you know – and in some ways if there’s not going to be further contact and whatever I…

Facilitator:	Doesn’t matter?

Interviewee:	Yes I don’t think it would really make – well it might be even – it might be worse to have somebody from the town because you might be looking at them feeling guilty and that kind of stuff whereas I think that if, you know, sort of seeing somebody more often there can be an advantage of someone being local because you might feel that they might [unclear 0:19:40.5] something like that.

Facilitator:	Yes and is there any – in terms of facilitator – any traits that you think are important that make up – make a good facilitator?

Interviewee:	You need to be outgoing, bright – yes I mean I thought the facilitators were good. I thought they showed enthusiasm for what they’re promise was and those sorts of things so I can’t – I certainly don’t think there was anything wrong with the people who did it and but I – yes just I think that sort of non-judgemental – yes and fun stuff’s important too.

Facilitator:	Was the information adequate for you or did you think you needed further – more information, less information?

Interviewee:	I think it was probably adequate, you know, I don’t think you probably – I think if people were there too long it becomes an issue but yes I think it was probably adequate. Yes.

Facilitator:	Last question. What would you prefer – group or individual session?

Interviewee:	Look I think certainly the group session – I think a group session’s fine. You know, I mean it may – perhaps an individual follow-up but I think the group session’s fine and yes.

Facilitator:	Is there anything else you want to add or anything else you think’s important for us to know about the program that could make it better in the future or?

Interviewee:	Not that I can think of. You know, as I said, perhaps just another, you know, another group session halfway through or something like that yes.

Facilitator:	Okay great. Thank you again Jackie so much for your time it’s really important to us.

Interviewee:	No worries.

Facilitator:	It’s great to hear your feedback and I’ll send you that gift card in the mail now. Is there anything else you want to ask me or any other questions or concerns that you have?

End of transcript
